# The anatomy of a personal health agent

**DOI:** 10.1038/s41586-026-11161-8

**Published:** 2026-10-21

**Authors:** A. Ali Heydari, Ken Gu, Vidya Srinivas, Hong Yu, Akshay Paruchuri, Zhihan Zhang, Yuwei Zhang, Qian He, Hamid Palangi, Nova Hammerquist, Ahmed A. Metwally, Brent Winslow, Yubin Kim, Kumar Ayush, Yuzhe Yang, Girish Narayanswamy, Maxwell A. Xu, Jake Garrison, Samuel Schmidgall, Amy Armento Lee, Jenny Vafeiadou, Ben Graef, Isaac R. Galatzer-Levy, Erik Schenck, Andrew Barakat, Javier Perez, Jacqueline Shreibati, John Hernandez, Anthony Z. Faranesh, Naghmeh Rezaei, Javier L. Prieto, Conor Heneghan, Jiening Zhan, Mark Malhotra, Shwetak Patel, Yun Liu, Tim Althoff, Xin Liu, Daniel McDuff, Xuhai Orson Xu

**Affiliations:** 1 Google Research, Mountain View, CA USA; 2 Google DeepMind, Mountain View, CA USA; 3 Vituity, Emeryville, CA USA; 4 Columbia University, New York, NY USA

**Keywords:** Health care, Signs and symptoms, Patient education

## Abstract

Large language models are enabling a new generation of health agents, yet their ability to address the diverse needs of individuals in everyday health settings remains underexplored^1,2^. Here we developed a comprehensive personal health agent that reasons over multimodal data from consumer wellness devices and medical records to provide personalized health support. Through analysis of web search and health forum queries, together with user-centred research involving end users and health experts, we identified four key user journeys: general health knowledge, personal data interpretation, wellness advice, and symptom assessment. These journeys informed three specialist sub-agents: (1) a data science agent that analyses longitudinal wearable and medical data, incorporating population-level statistics to contextualize numerical insights; (2) a health domain expert agent that integrates personal and contextual data with health knowledge to generate personalized insights; and (3) a health coach agent that synthesizes insights, supports multi-turn interaction and goal setting, applies specified psychological strategies and tracks progress. We integrated these capabilities in the personal health agent, an open-source multi-agent framework for dynamic, personalized health interactions. We evaluated the sub-agents and full system across 11 benchmark tasks using automated and human evaluation, with more than 7,000 annotations and 1,350 h of effort from health experts and end users. This represents the most comprehensive evaluation of a personal health agent thus far and provides a concrete foundation for improving access to high-quality personalized health support more broadly.

## Abstract (web summary)

A multi-agent framework that coordinates data science, medical reasoning and health coaching over longitudinal wearable and clinical data to provide holistic personal health support is described.

## Main

Rapid advancements in large language models (LLMs) have driven transformative changes in health. Current LLMs can support various clinical tasks, such as medical documentation^3^, diagnosis^4^, decision support^5^ and drug discovery^6,7^, as well as consumer health tools, including virtual health assistants^8^, symptom checkers^9^, digital coaches and advisors^10,11^, and clinical examination dialogues^12^.

Wearables add continuous activity, sleep, heart rate variability and other physiological signals^13,14^, providing longitudinal context for health support. Recent systems connect these data to LLMs^1,2,8,15–17^, but numerical time-series reasoning remains unreliable^18^. Previous systems either cover narrow fitness or sleep questions without deep numerical analysis^8^, or address broader numerical queries without evaluating health expertise and interactive coaching^2^. A comprehensive system must combine these capabilities while maintaining a coherent interaction^19^. Building on these multifaceted developments and the integrated nature of personal health journeys, we envision that the era of intelligent personal health agents is arriving.

Individual needs for daily health and wellness support are highly diverse^2,20,21^. We used a user-centred design process spanning end-user queries and discussions, surveys and an expert workshop to cover general wellness, fitness and medical topics. The analysis identified four categories of needs: (1) general health knowledge, (2) personal-data interpretation, (3) wellness advice, and (4) health symptoms (Supplementary Table 1). These categories require different capabilities. Personal data questions demand numerical precision and code execution; coaching requires open-ended, empathetic dialogue; and knowledge or symptom questions require factual grounding. Our experiments confirmed that a monolithic LLM architecture struggles to balance these competing objectives. We therefore adopted a modular multi-agent architecture that decouples these technical requirements while preserving a cohesive user experience through orchestration and synthesis of individual sub-agent processes.

**Fig. 1:**
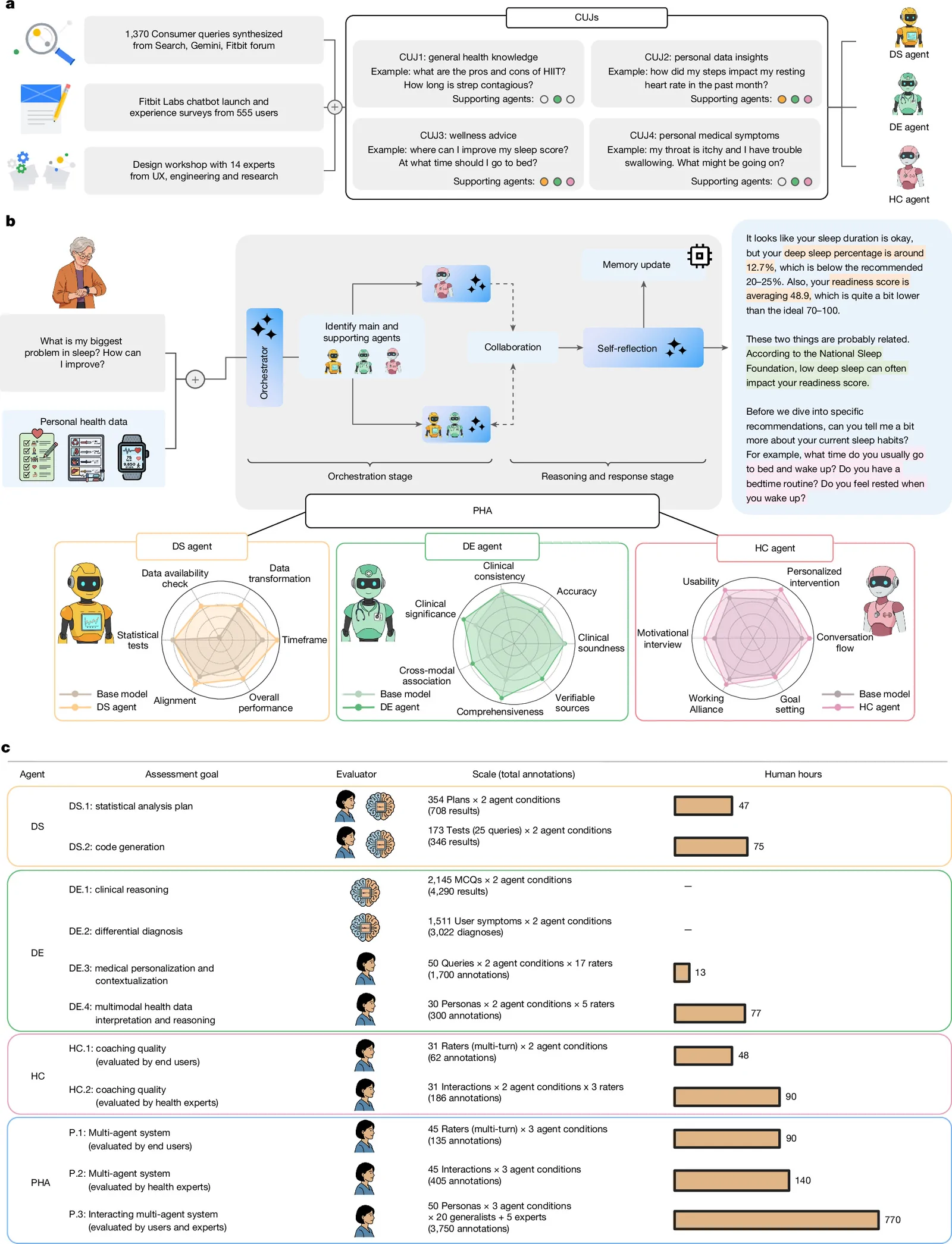
Overview of the PHA. **a**, The anatomy of personal health needs with a user-centred design process. **b**, Design a multi-agent system, powered by state-of-the-art LLMs, consisting of specialist DS, health DE and HC agents to address diverse consumer health needs. ‘Base model’ refers to the Gemini 2.0 family. **c**, Our work represents the most comprehensive evaluation of a health agent thus far (more than 7,000 annotations and 1,350 h of effort on 11 benchmarks). MCQ, multiple-choice question.

Three specialists address these requirements. The data science agent (DS agent) converts personal and population-level time series into quantitative analyses, such as estimating changes in running performance. The domain expert agent (DE agent) interprets wearable data, health knowledge and available medical records (in this study, medical records were limited to blood test results because of protocol-level data access constraints; future work could integrate electronic health records with other modalities), for example, by contextualizing a biomarker value. The health coach agent (HC agent) applies evidence-based strategies such as motivational interviewing^22^ to support goal setting, identify barriers and guide behavioural change. Because many requests span these roles, we drew inspiration from multi-agent collaboration strategies, such as voting^23,24^, iteration^25^, debate^26^ and hierarchical team formation^27^, and built the multi-agent personal health agent (PHA) with an orchestrator to assign work and synthesize the outputs of the agents (Fig. 1a). Of note, PHA is not intended to supplant human health providers, but to explore the technological frontier of consumer empowerment, envisioning how a unified agent can provide more holistic, actionable health insights.

We developed a holistic evaluation framework (Fig. 1b) that maps to the capabilities identified through the user-centred analysis. We benchmarked the DS agent on its ability to generate robust analysis plans and codes, the DE agent on its capacity for factuality and evidence-based reasoning, and the HC agent on its coaching effectiveness through user studies with both end users and experts. We then evaluated the integrated PHA system through open-ended, multi-modal conversations that reflect diverse health scenarios. Across 11 benchmark tasks, the evaluation included more than 7,000 human annotations and 1,350 h of effort (651 end-user hours and 699 expert hours), validating the advantage of each specialized sub-agent and demonstrating the promising real-world applicability of our integrated multi-agent health agent.

By combining a modular multi-agent architecture, a capability-matched evaluation framework and an open-source agent implementation, this work establishes a reproducible foundation for developing, comparing and testing PHAs for everyday health and well-being of individuals.

## User-centred analysis of health needs

We first performed a systematic, user-centred analysis to define the landscape of personal daily health needs. We synthesized 1,370 consumer health queries from search engines and health forums, health goal surveys from 555 users, and a design workshop with 14 UX and engineering experts (see Methods). This analysis identified four critical user journeys (CUJs; Fig. 1a):

1. General health knowledge: users seek factual information about broad health topics, medical conditions and wellness concepts; for example, ‘what are the pros and cons of HIIT?’.
2. Personal data insights: users want to understand and interpret their own data from wearables or medical records to identify trends and relationships; for example, ‘how did my steps impact my resting heart rate in the past month?’.
3. Wellness advice: users look for actionable and personalized recommendations to improve their well-being or achieve specific health goals; for example, ‘to improve my sleep score, what time should I go to bed?’.
4. Personal medical symptoms: users describe their symptoms to explore potential causes and understand what might be happening; for example, ‘my throat is itchy and I have trouble swallowing. What might be going on?’.

Supplementary Table 1 provides a detailed breakdown of these categories. Because CUJs often overlap within one conversation, they motivated the production of a modular architecture spanning data analysis, health-domain reasoning and coaching.

## Modular multi-agent health architecture

We developed the PHA as a modular system of three specialist agents, each addressing a complementary user need (Fig. 1b).

**Extended Data Fig. 1:**
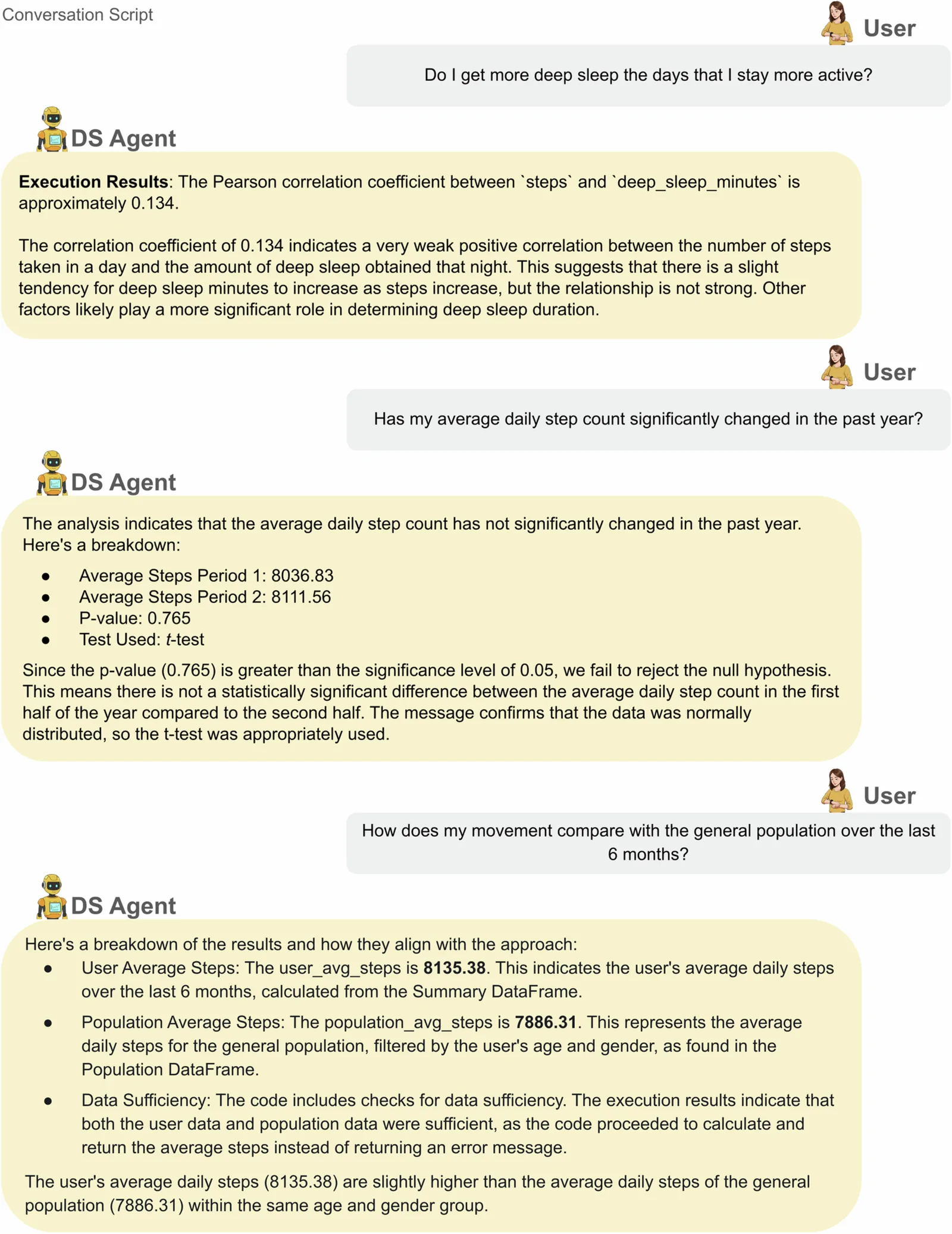
Example Conversation with the DS Agent.

The DS agent analyses personal time-series data to derive quantitative insights. Although LLMs have been explored for data science tasks^2,28–35^, their capacity to interpret ambiguous, open-ended personal health inquiries and perform statistically sound analyses remains a key gap. Our DS agent is designed to translate such queries into robust analysis plans and accurate computation code. Example conversations between a user and the DS agent are shown in Extended Data Fig. 1. More technical details can be found in the Methods.

**Extended Data Fig. 2:**
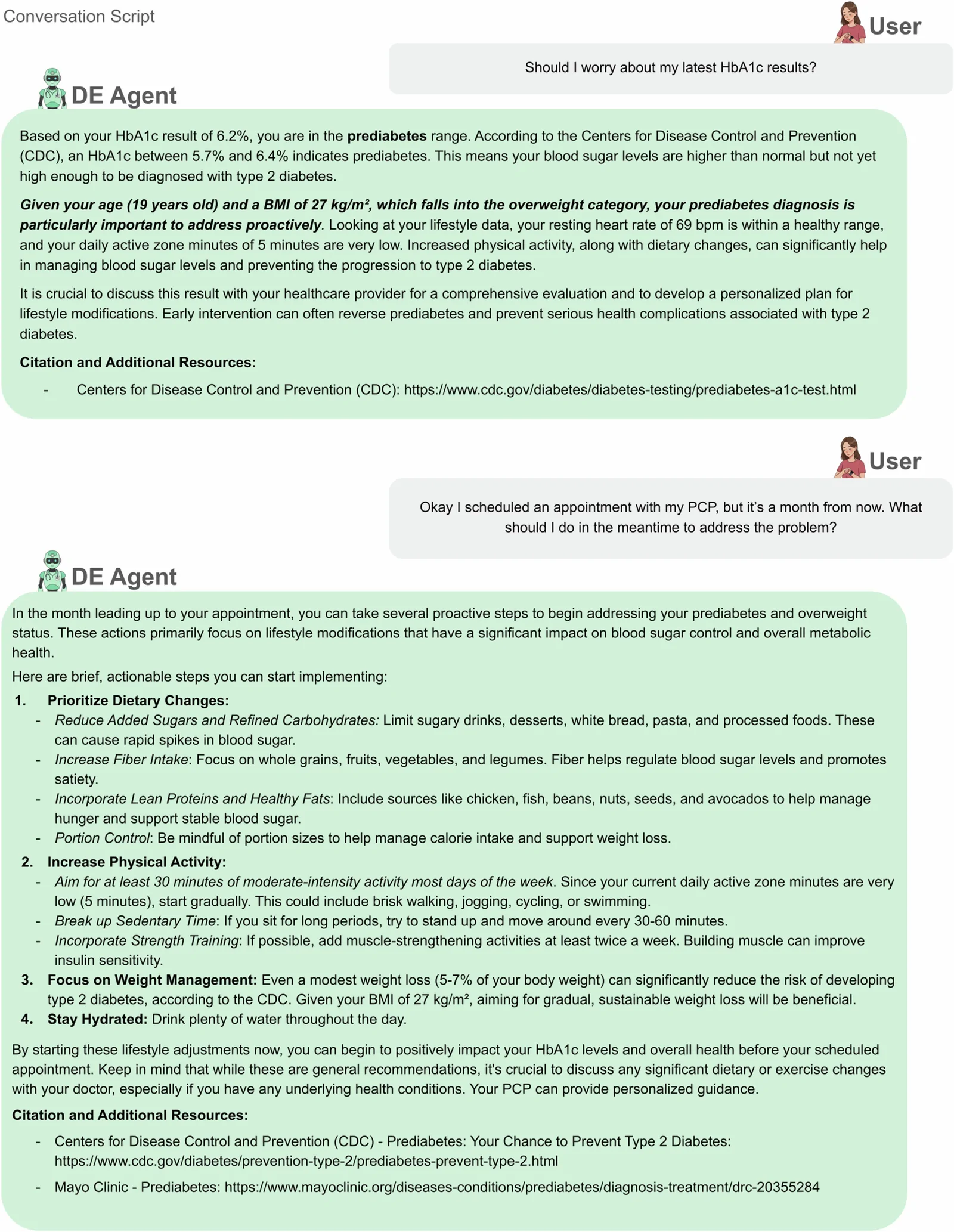
Example Multi-Turn Conversation with the DE Agent.

The DE agent provides evidence-based, clinical context for user data. Previous work has benchmarked the medical knowledge of LLMs^36–38^, but the essential skill of reasoning and synthesizing across multimodal personal data (for example, wearables, laboratory results and user context) is underexplored. Our DE agent is equipped with a suite of tools to integrate these disparate data streams and generate accurate, personalized interpretations. Example conversations with the DE agent are shown in Extended Data Fig. 2. More technical details are in the Methods.

**Extended Data Fig. 3:**
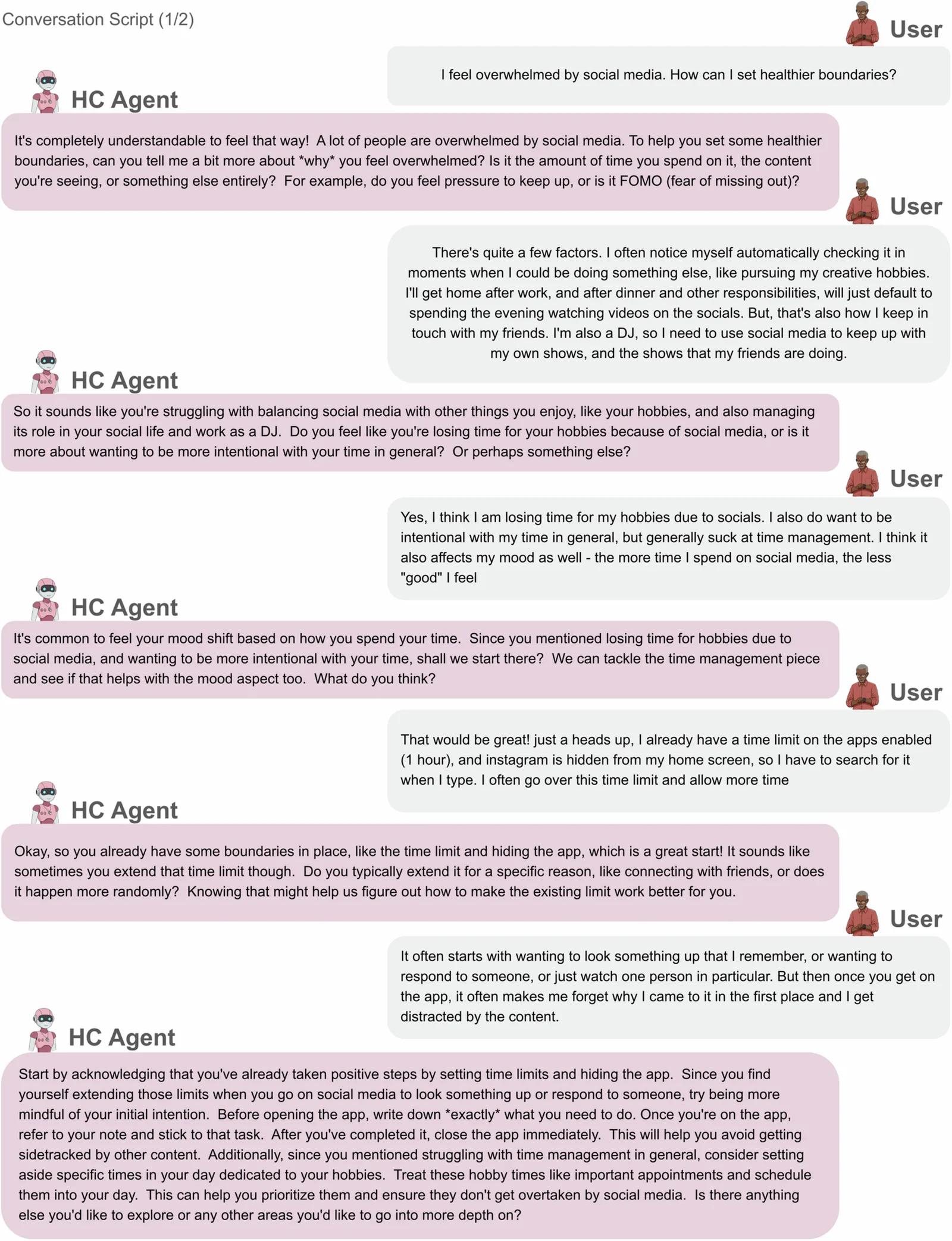
Example Multi-Turn Conversation with the HC Agent (Part 1/2).

**Extended Data Fig. 4:**
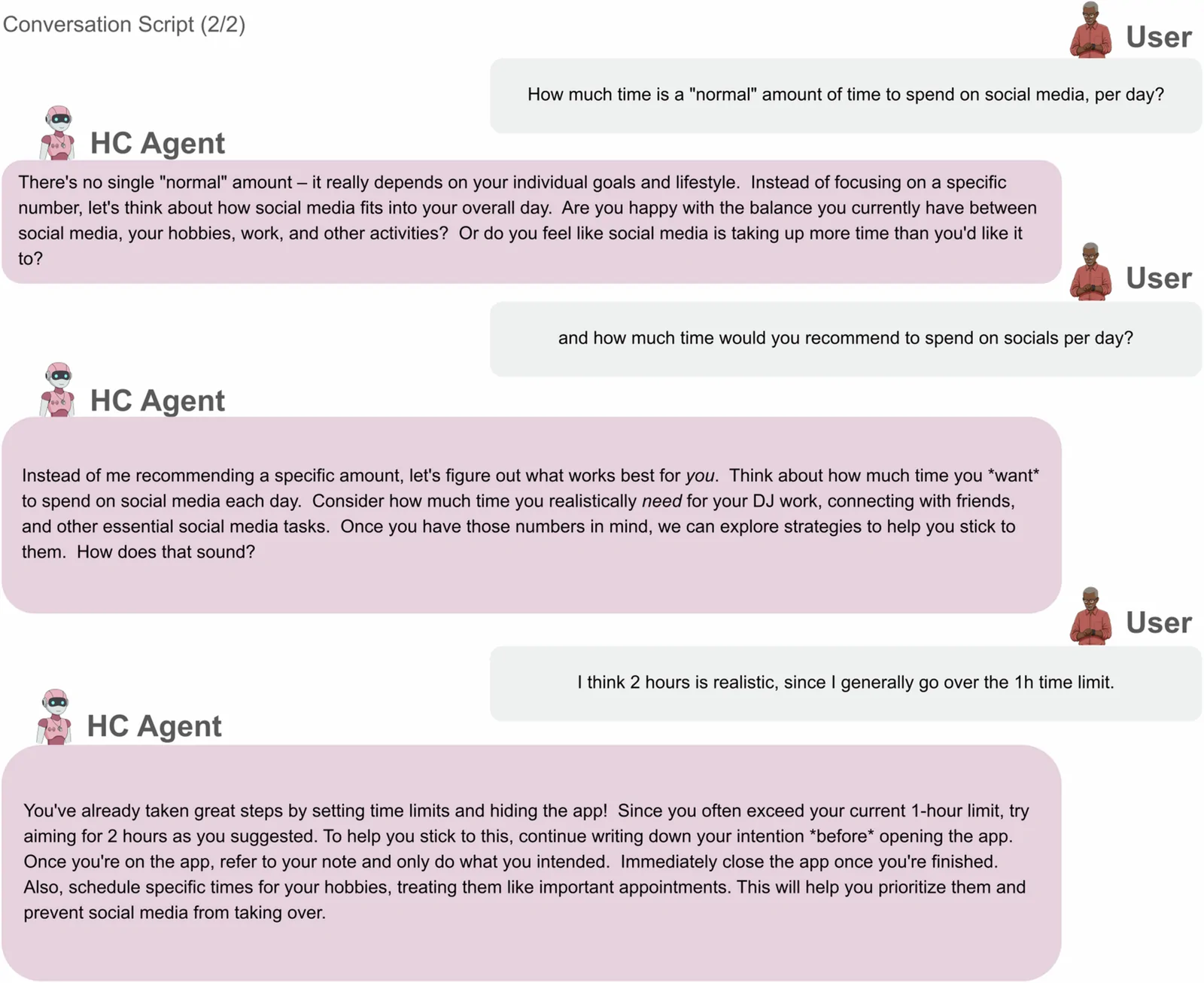
Example Multi-Turn Conversation with the HC Agent (Part 2/2).

The HC agent guides users through multi-turn conversations on goal setting and behavioural change. Although artificial intelligence coaching is an emerging area^39–41^, support for open-ended dialogue across a wide range of wellness topics is limited. We designed our agent with a modular architecture to provide an expert-aligned, personalized coaching experience that effectively balances information gathering with actionable advice. Example conversations between a user and the HC agent are shown in Extended Data Figs. 3 and 4, with more details in the Methods.

**Extended Data Fig. 5:**
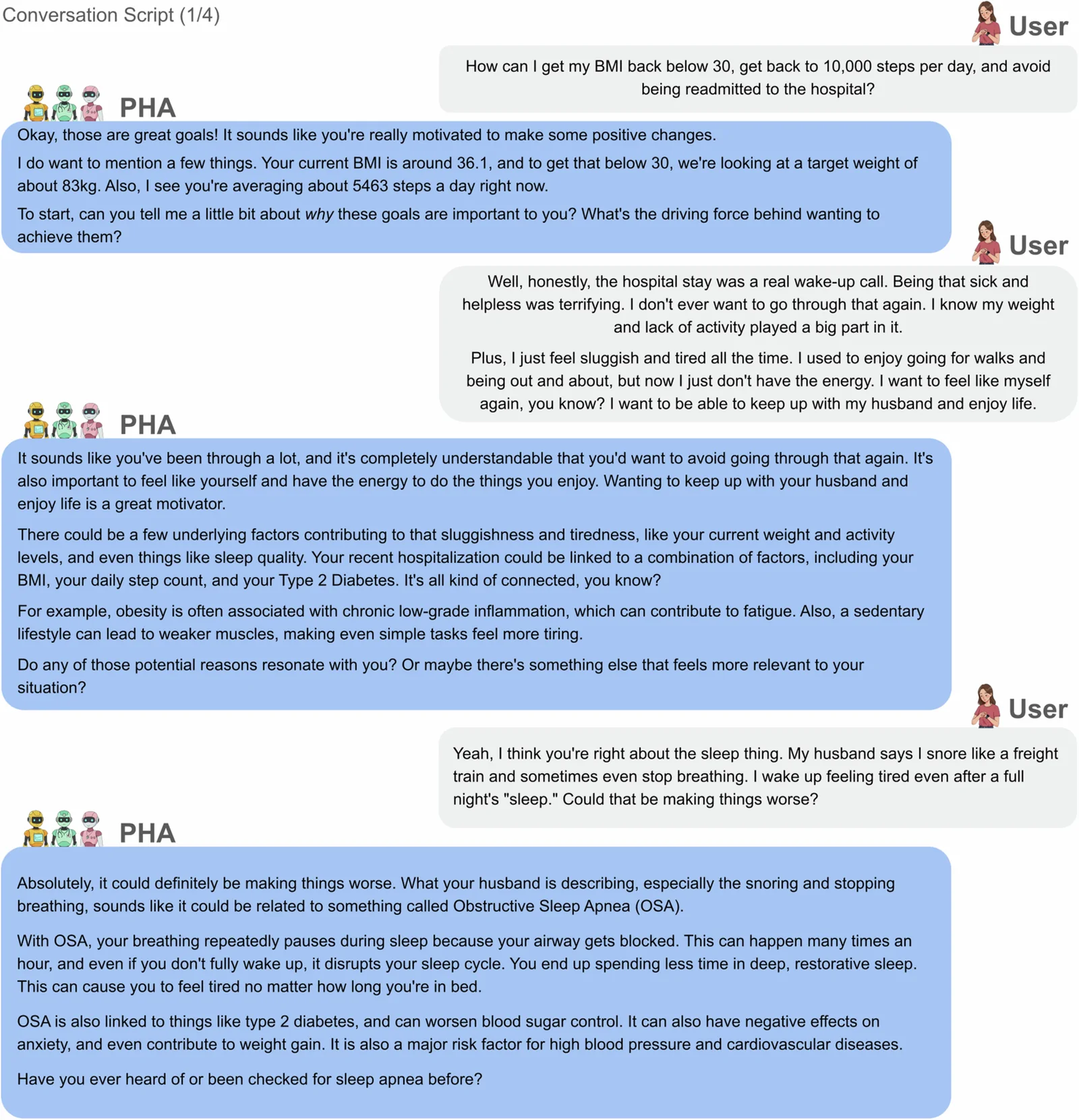
Example Multi-Turn Conversation with the PHA (Part 1/4).

**Extended Data Fig. 6:**
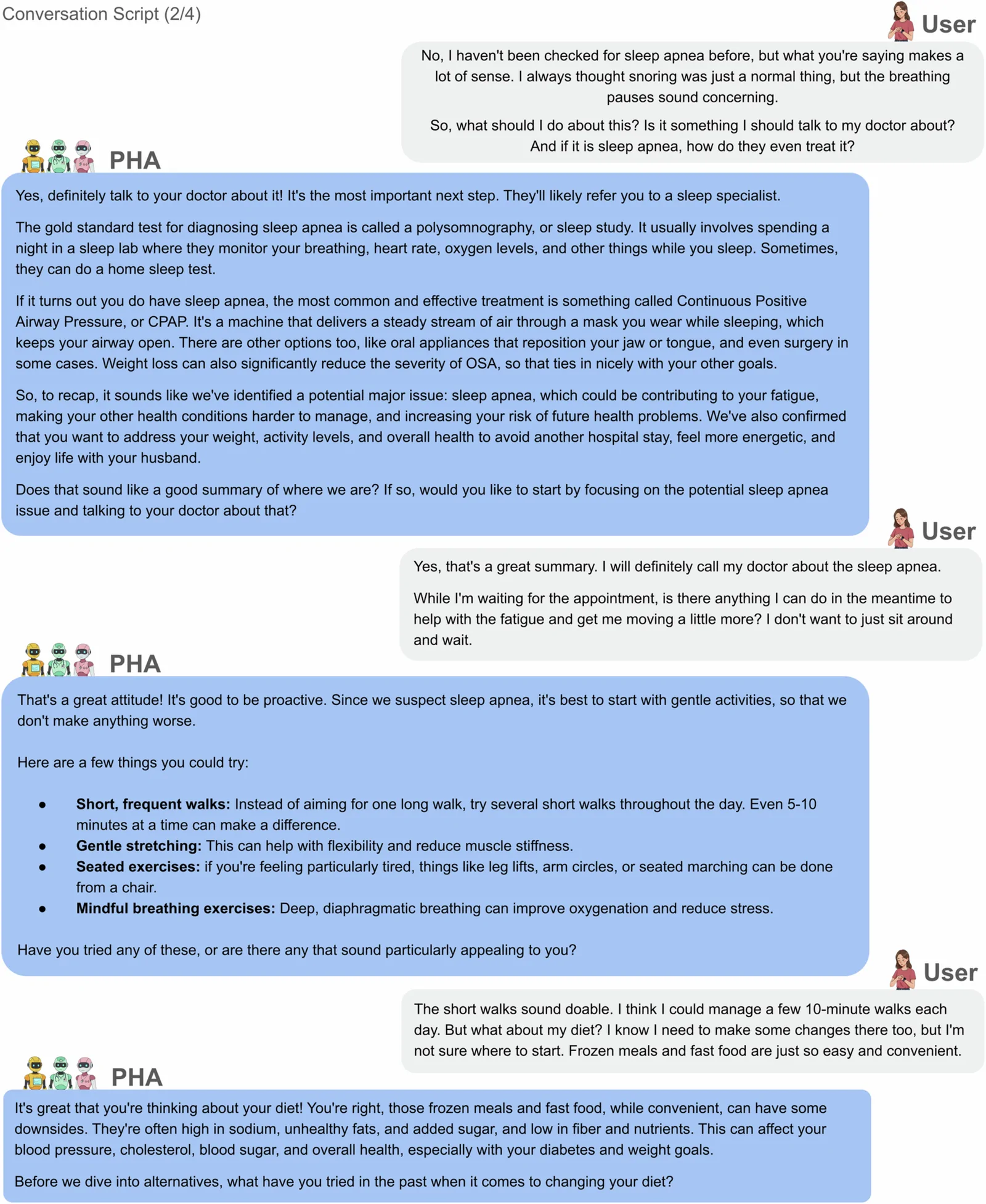
Example Multi-Turn Conversation with the PHA (Part 2/4).

**Extended Data Fig. 7:**
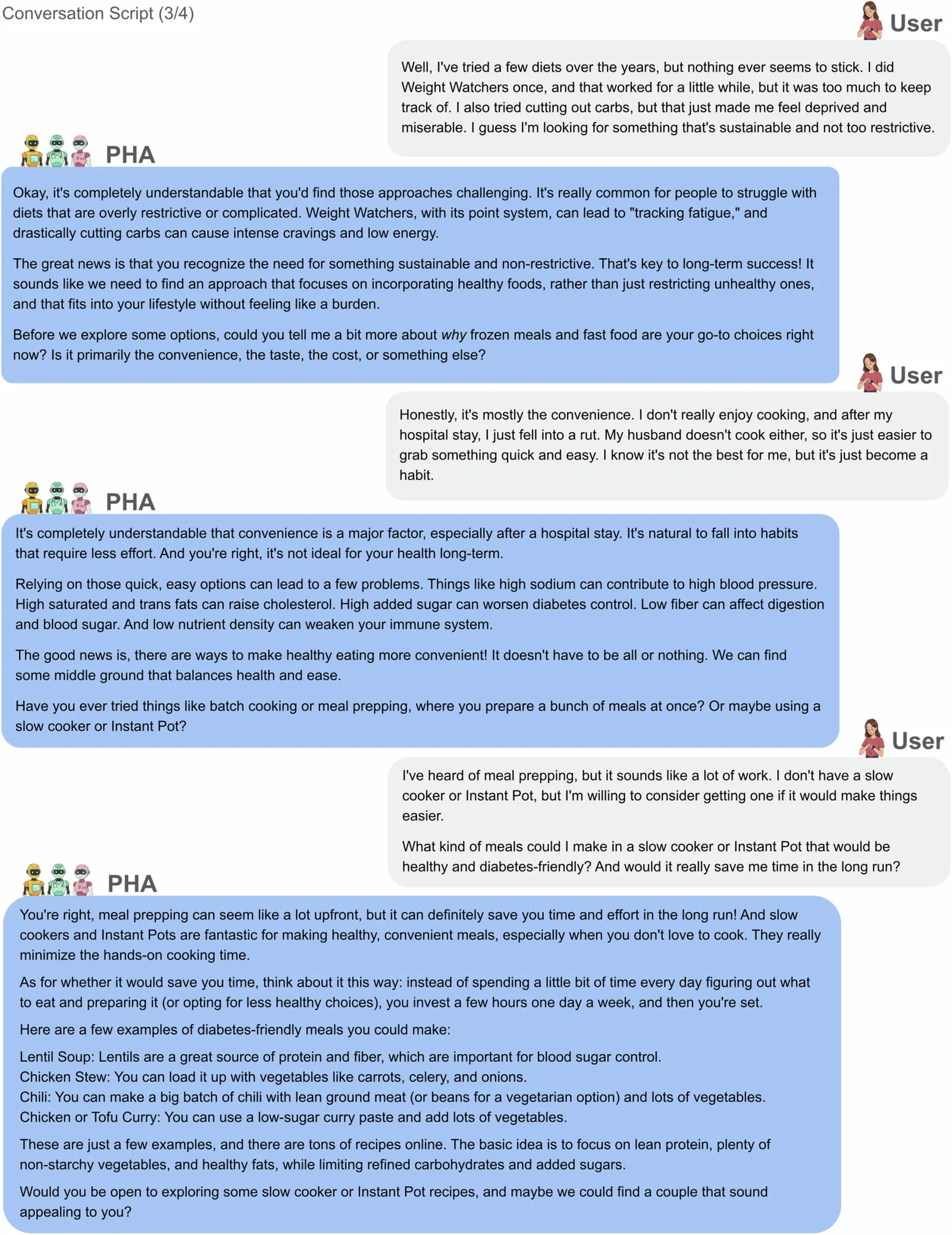
Example Multi-Turn Conversation with the PHA (Part 3/4).

**Extended Data Fig. 8:**
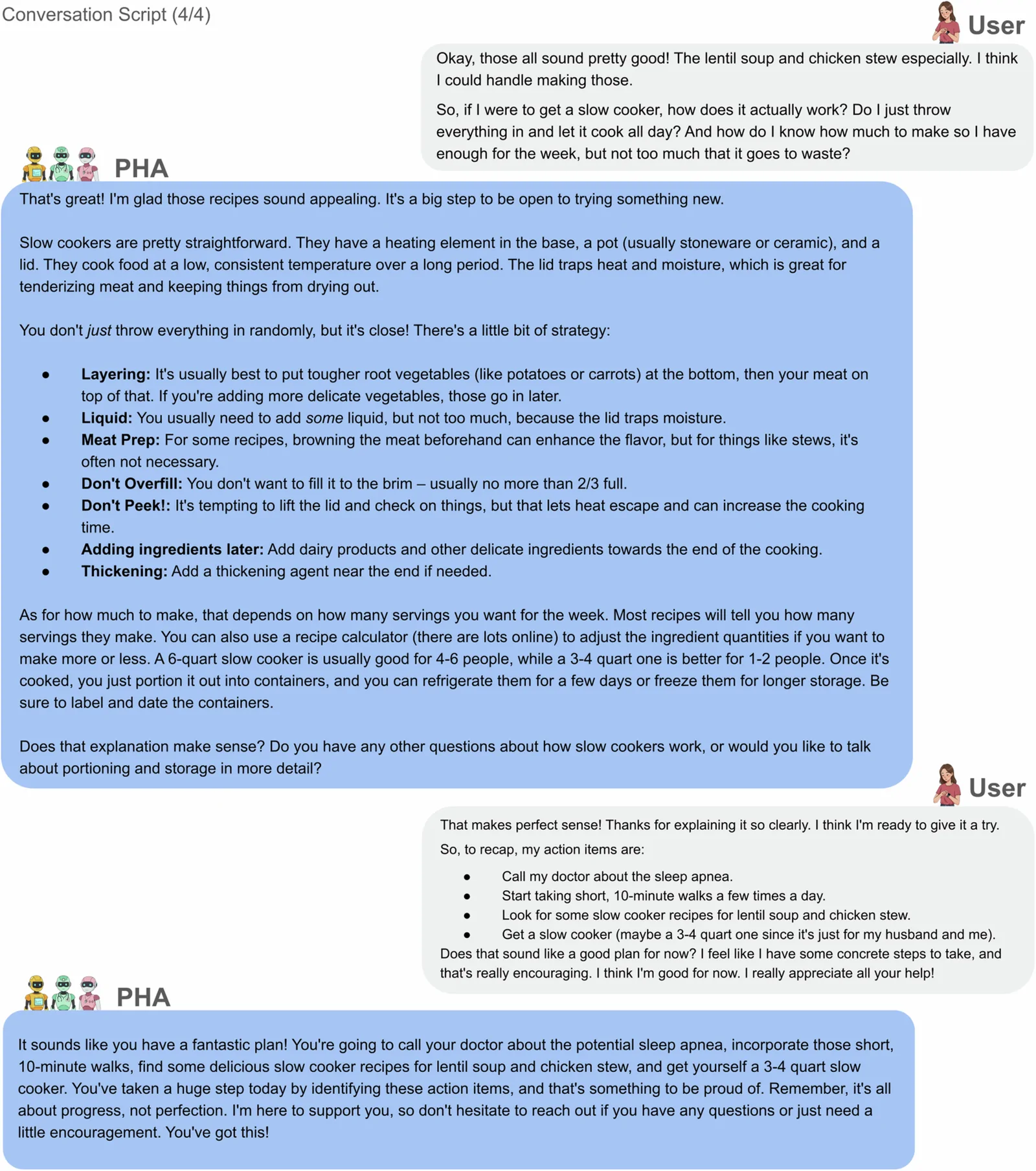
Example Multi-Turn Conversation with the PHA (Part 4/4).

Finally, the PHA is the fully integrated system. A central orchestrator dynamically manages the workflow, decomposing user queries, assigning tasks to the appropriate specialist agents and synthesizing their outputs through an iterative reflection process to provide a single, cohesive and context-aware response to the user^42^. Example multi-turn conversations with PHA are shown in Extended Data Figs. 5–8, with more technical details in the Methods.

Figure 1a maps CUJs to specialist agents. For example, the query ‘I want to understand my sleep data last week and know how I can improve my sleep’ (CUJ2 and CUJ3) recruits the DS agent to analyse wearable data, the DE agent to provide evidence-based sleep knowledge and the HC agent to personalize advice. Supplementary Table 1 enumerates the 26 query sub-types and their primary and supporting agents.

To rigorously validate this framework, we designed the most comprehensive evaluation of a personal health agent thus far. Our evaluations were grounded in data from the large-scale Wearables for Metabolic Health (WEAR-ME) study (*n* = 1,165) to ensure real-world validity^43^.

**Extended Data Fig. 9:**
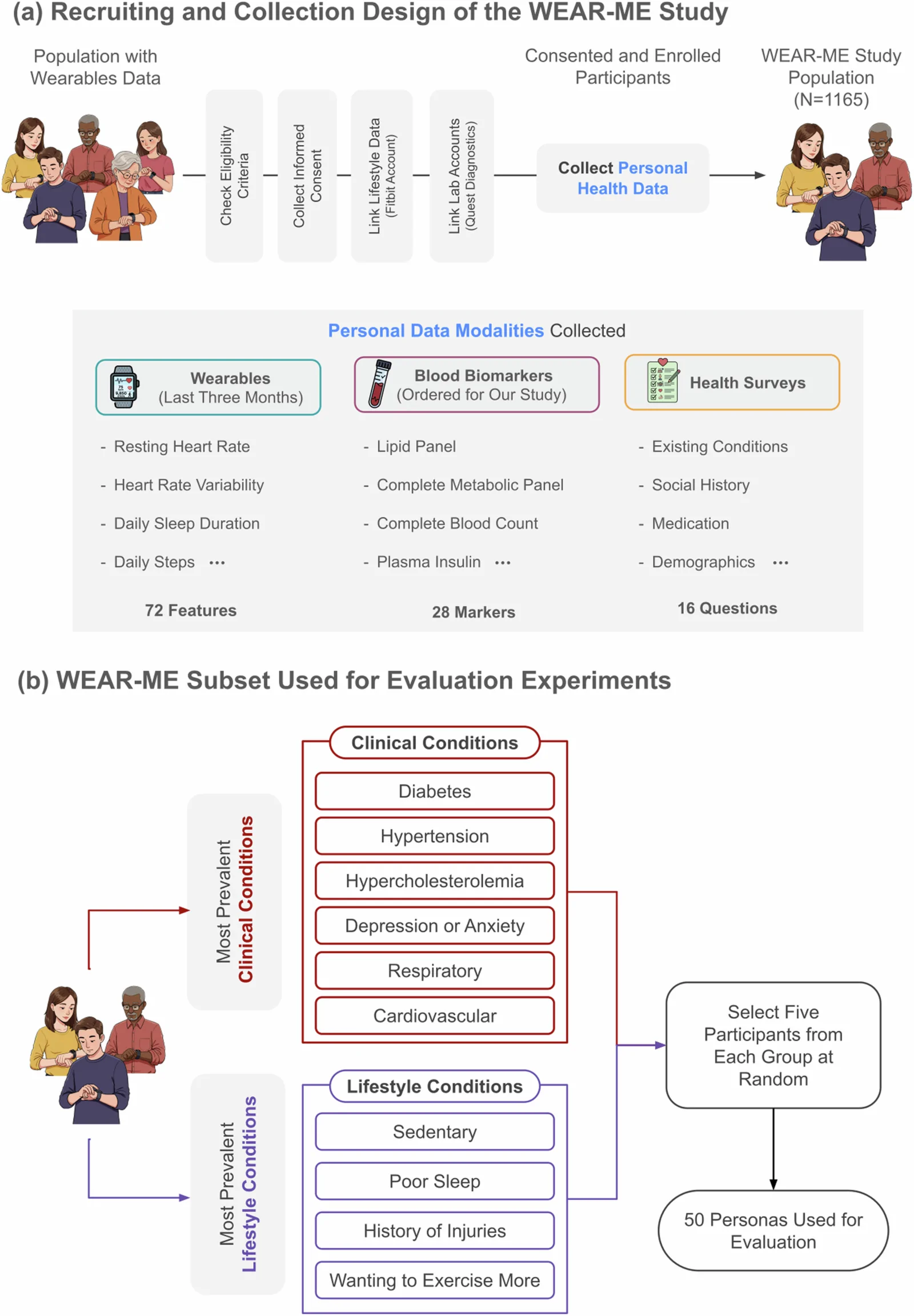
Overview of the wearables and metabolic health study (WEAR-ME) study. (**a**) The WEAR-ME study consists of Fitbit users who opted-in to participate in our study. Those who consented and enrolled in the WEAR-ME study were then asked to visit one of the Quest centers for blood biochemistry data collection. Each participant contains multimodal data from Fitbit, blood test results, and questionnaires. (**b**) For our human evaluations, we selected a subset of the WEAR-ME data based on prevalent health conditions present among the WEAR-ME participants. We identified 10 common profiles, and selected five individuals at random from those profiles. More details are introduced in Supplementary Section B.

This dataset provides rich, multimodal data for each participant with data-quality validation (for example, filtering users with short or unreliable wearing periods, and high missing data rates), including longitudinal wearable data, clinical blood test results and detailed health questionnaires (see Extended Data Fig. 9). The evaluation assesses both the standalone performance of each sub-agent and the synergistic efficacy of the integrated PHA system across 11 benchmark tasks. We used the Gemini 2 family (Flash/Pro) of models^44^ for our evaluation, which has demonstrated strong performance on medical tasks^8,45,46^. In this work, our focus is not to train a new LLM but to design and construct the agentic system around a given base LLM. This multi-faceted approach involved both automated and human evaluations, drawing on over 7,000 annotations and 1,350 h of effort from health experts and end users. The detailed justification of the choice of base LLM, evaluation protocols and a full description of the WEAR-ME dataset are provided in the Methods.

## The DS agent improves analysis and coding

**Fig. 2:**
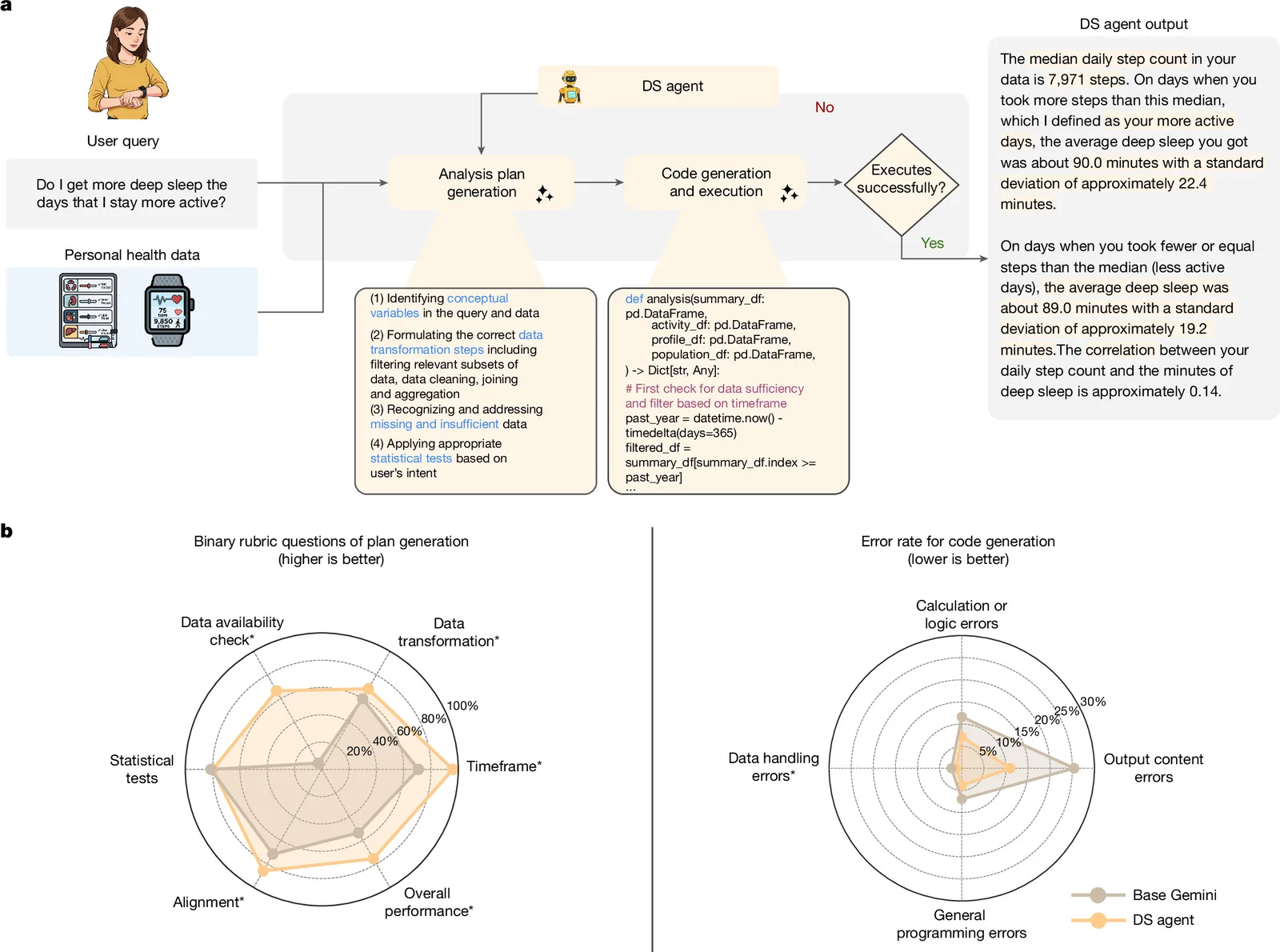
Schematic and evaluation results of the DS agent. **a**, The architecture of the DS agent, which first generates a detailed, natural language statistical analysis plan from a user’s query and a data schema summary (see Supplementary Section C.2 for prompting details). Subsequently, it produces and executes code to implement this plan. An iterative self-correction loop allows the agent to revise the code on the basis of execution errors, the original query and the analysis plan. **b**, Results of the performance evaluation of the DS agent, demonstrating significant improvements over the base Gemini model in both analysis plan quality and code generation pass rates. Supplementary Section C.3 summarizes the evaluation details and rubrics. Statistical indicator: *P < 0.05.

The DS agent is designed to address a critical gap in existing systems: the ability to interpret ambiguous, open-ended user health queries (for example, ‘am I getting more fit?’) and translate them into statistically sound, executable analyses. The agent uses a two-stage architecture that first generates a structured, natural language analysis plan and then translates that plan into an executable Python code with an iterative self-correction loop (Fig. 2a). To validate this design, we independently evaluated the performance of the agent on these two core tasks: analysis plan generation and code generation.

### Analysis plan generation

We assessed the analysis plan quality by the DS agent using an expert-defined rubric covering six pre-specified dimensions of methodological soundness (timeframe, data transforms, data sufficiency, summary statistics, statistical tests and overall alignment; full definitions in the Methods, DS agent task 1). To enable scalable evaluation, we developed an LLM-based autorater validated against 354 annotations from 10 expert data scientists (intraclass correlation coefficient(3,1) = 0.838; details in Supplementary Section C.3.1).

As shown in the left panel of Fig. 2b, the DS agent significantly outperformed the baseline overall (average score of 75.6 ± 1.4% versus 53.7 ± 1.8%, *P* < 0.001, *r* = 0.925, Wilcoxon signed-rank test). The most notable gain was on data availability, in which the near-failure baseline rate of 5.3 ± 1.6% rose to 66.5 ± 3.7%, reflecting a well-documented LLM weakness in detecting missingness and artefacts^47^. Major gains were also observed on timeframe, data transforms and alignment, whereas statistical tests showed no significant difference (a category in which the baseline already performed strongly). Per-dimension statistics are reported in Supplementary Table 5.

### Code generation and execution

We evaluated code generation against a benchmark of 173 unit tests created by 7 data science experts across 25 challenging query–plan pairs (Methods; Supplementary Section C.3.2). The DS agent achieved a 75.5 ± 3.3% first-attempt pass rate, significantly outperforming the base Gemini’s 58.4 ± 3.7% (*χ*^2^ = 26.3, *P* < 0.001, McNemar’s test), and rose to 79.0% after up to five iterative self-correction trials. The largest improvement was on data-handling errors (which typically render the entire pipeline unusable) dropping from 25.4 ± 3.3% to 11.0 ± 2.4% (*χ*^2^ = 12.3, *P* < 0.001). Per-error-category breakdowns are in Supplementary Table 6. We further verified that these gains generalized to other SOTA LLMs, indicating that the DS agent architecture is model agnostic (see Supplementary Section C.5.3).

## The DE agent synthesizes multimodal insights

**Fig. 3:**
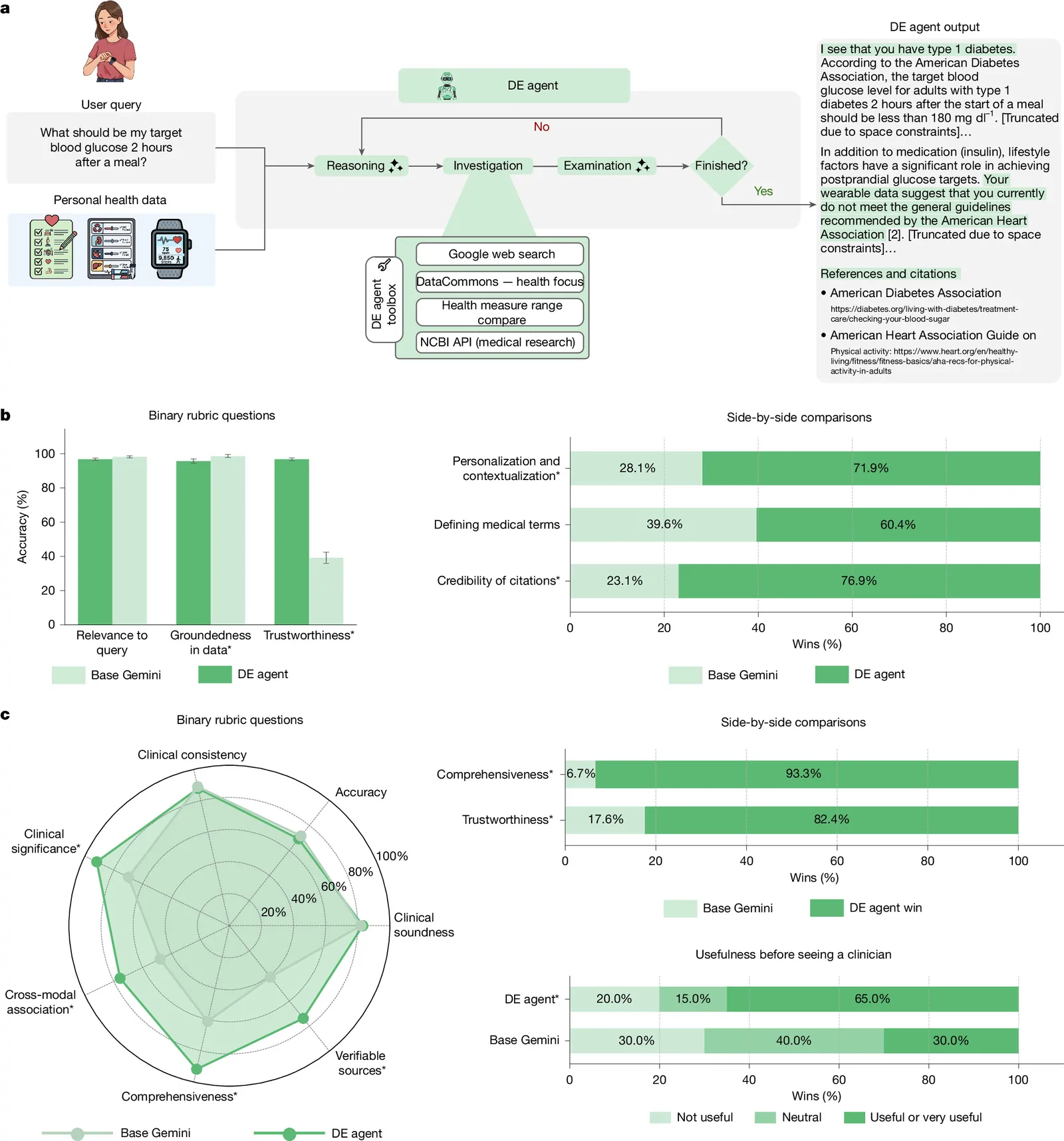
Schematic and evaluation results of the DE agent. **a**, Overview of the workflow for the DE agent, which takes the query and personal health data of a user as input. The agent uses an iterative process of reasoning, investigation and examination, using a toolbox with access to specialized resources (see Supplementary Section D.1 for prompting details). **b**, End-user evaluation (*n* = 17) of the DE agent significantly outperforms a base Gemini model in multimodal dimensions in single-turn, contextualized question and answer (Q&A). **c**, Clinician evaluation (*n* = 5) of multimodal health summaries shows the same conclusions. Supplementary Section D.2 summarizes the evaluation details and rubrics. The error bars indicate the standard error of the mean; statistical indicator: *P < 0.05.

The DE agent functions as a specialized cognitive layer designed to synthesize, contextualize and validate health information against authoritative sources and the personal data of a user. It uses a multi-step reasoning architecture that uses a suite of tools, including web search, the National Center for Biotechnology Information (NCBI) API and the DataCommons API, to iteratively investigate user queries and ground its responses in verifiable evidence (Fig. 3a). We systematically evaluated the performance of the DE agent across its four foundational capabilities: factuality of health information, diagnostic reasoning through multi-turn conversations, contextualization and personalization, and multimodal data synthesis.

### Factuality of health information

We benchmarked factuality on more than 2,000 expert-level multiple-choice questions from board certification and coaching examinations across endocrinology, cardiology, fitness and sleep medicine. The DE agent improves the accuracy of the base Gemini across nearly all domains, with overall 83.6% versus 81.8% (*χ*^2^ = 9.51, *P* = 0.002, McNemar’s test, OR = 1.67; specific results are in the Methods, DE agent task 1; Supplementary Table 11).

### Accuracy of diagnostic conversations

We evaluated the ability of the agent to generate a differential diagnosis from common, self-reported symptoms. On a retrospective dataset of 2,000 real-world consumer health cases, the DE agent significantly outperformed a Gemini 2.0 Pro-based state-of-the-art DDx agent^4^ (*P* < 0.001, *r* = 0.156, Wilcoxon signed-rank test), reaching a top one accuracy of 46.1% (∆ = 4.7% over the previous SOTA), with consistent gains at the top five and top ten (Supplementary Table 12 and Supplementary Fig. 4). Similar to the DS agent, we further verified that these gains generalized to other SOTA LLMs, indicating that the DE agent architecture is model agnostic (see Supplementary Section D.3.3).

### Personalized answers to health queries

We curated a set of 50 personalized health-related questions based on the query set in the previous user-centred design analysis across diverse demographics and health conditions. In a blinded study, 17 end users compared DE agent and baseline Gemini responses on these contextualized health queries using Boolean rubrics and side-by-side rankings (Methods, DE agent task 3; Supplementary Table 9). On binary rubrics (Fleiss’ *K* ≥ 0.684; Fig. 3b), the most consequential gap was on trustworthiness (96.9 ± 0.8% versus 38.7 ± 3.3%, *P* < 0.001, *β* = 6.873, binomial generalized linear mixed model (GLMM)); relevance to query and groundedness in data were comparable across systems (Supplementary Table 15). For side-by-side, end users preferred the DE agent on personalization and contextualization (71.9 ± 1.5%, *P* < 0.001) and credibility of citations (76.9 ± 1.5%, *P* < 0.001), see Supplementary Table 16 for details.

### Multimodal clinical health summaries

We assessed the ability of the DE agent to synthesize unstructured text, structured wearable data and PDF laboratory results from 30 WEAR-ME participants into comprehensive health summaries. A panel of five expert clinicians independently evaluated summary quality in a blinded side-by-side comparison (Supplementary Table 10). On binary rubrics, the DE agent significantly outperformed the base model on clinical significance (96.4% versus 73.8%), cross-modal association (79.4% versus 50.0%), comprehensiveness (96.4% versus 64.2%) and citation credibility (77.6% versus 42.7%; all *P* < 0.001, binomial GLMM; Supplementary Table 17). In direct comparisons, clinicians preferred DE agent summaries on comprehensiveness (93.3%, *P* < 0.001) and trustworthiness (82.4%, *P* < 0.001; Supplementary Table 18). On overall clinical usefulness, 65% of DE agent summaries were rated ‘useful’ or ‘very useful’ versus 30% for the base model (*P* < 0.001; Supplementary Table 19). The expert safety review of the summaries by the DE agent showed that they largely have no or minimal potential harm (83%), and although they are non-trivial, 15% have mild harm and 2% have moderate-to-severe harm (Supplementary Figs. 5 and 6). We highlight this concern in the ‘Discussion’ section.

## The HC agent for expert-aligned coaching

**Fig. 4:**
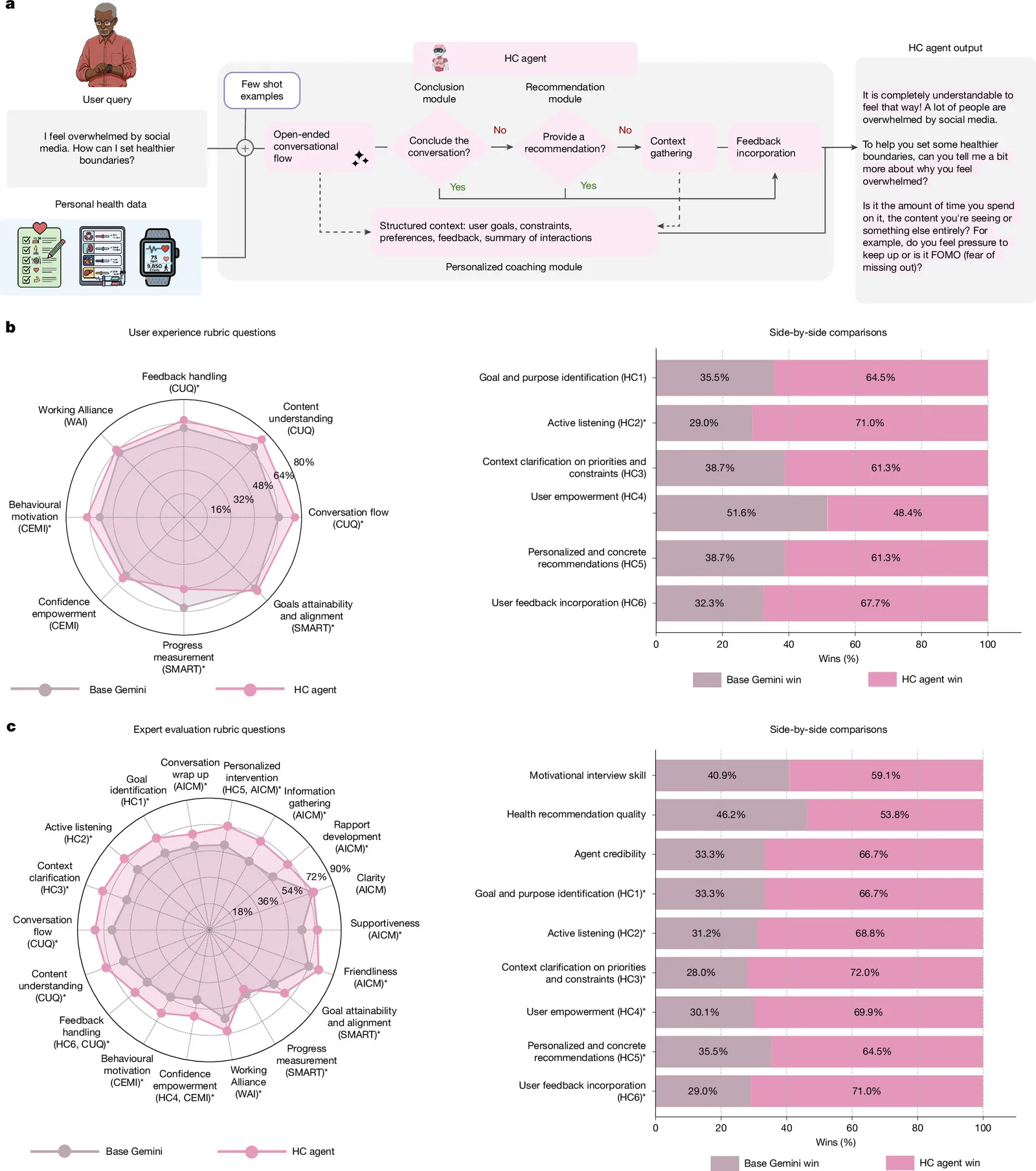
Schematic and evaluation results of the HC agent. **a**, The HC agent architecture uses a modular process with a core personalized coaching module and conversation flow modules (recommendation and conclusion) to adapt the dialogue and strategically time recommendations (Supplementary Section E.1 shows prompting details). **b**, End-user evaluation (*n* = 31) shows that the HC agent significantly outperforms a base model in multi-turn coaching conversations, leading to higher user engagement and preference. CEMI, client evaluation of motivational interviewing; CUQ, chatbot usability questionnaire; SMART, specific, measurable, attainable, relevant and time-bound; WAI, Working Alliance inventory. **c**, Coach expert evaluation (*n* = 3) of conversation transcripts confirms these conclusions, revealing an even more pronounced advantage for the HC agent from the perspectives of experts (more details provided in Supplementary Section E.2). AICM, artificial intelligence coaching metrics. Statistical indicator: *P < 0.05.

The HC agent is designed for robust coaching on goal setting and wellness planning, navigating multi-turn, mixed-initiative dialogue. Six expert-derived design themes emerged from a formative interview study with 11 health experts: (1) goal and purpose identification, (2) active listening, (3) context clarification, (4) user empowerment, (5) personalized and concrete coaching recommendations, and (6) user feedback incorporation (Methods). The agent uses a modular architecture grounded in motivational interviewing (Fig. 4a), separating a core personalized coaching module from specialized conversation flow modules. We evaluated the agent through a dual-perspective study with both end users and health coaching experts.

### End-user evaluation of coaching

We recruited 31 participants for blinded, counterbalanced sessions comparing our HC agent with a baseline built on the same base Gemini model (Methods, HC agent task 1). Across the chatbot usability questionnaire, the Working Alliance inventory, the client evaluation of motivational interviewing and SMART goal-setting instruments (Fig. 4b), the HC agent showed significant advantages on conversation flow, motivational interviewing and feedback handling (all *P* ≤ 0.05, GLMM with log link function), and was preferred over the baseline on five of six themes (win rates of 61–71%; significant advantage on active listening, *P* = 0.025; Supplementary Table 27 perspectives and Supplementary Table 28). The HC agent also produced more natural conversation endings (83.9% versus 74.2%). The one exception was progress measurement, for which the baseline scored higher. We explicitly address this in the integrated PHA design.

### Expert validation of coaching

Three health experts independently re-evaluated the end-user conversation transcripts using the end-user rubrics plus a specialized artificial intelligence coaching metrics instrument (Methods, HC agent task 2; Supplementary Section E.2.3). The HC agent outperformed the baseline on nearly every coaching dimension (∆ = 7–16%, all *P* < 0.001 except clarity; see Fig. 4c, Supplementary Tables 29 and 30). The expert-perceived advantage was substantially larger than the end-user-perceived advantage (relative ∆ = 10.2 ± 1.2% versus 3.5 ± 2.5%), and experts preferred the HC agent across all six HC themes (win rates of 64.5–72.0%, all *P* < 0.05). As with end users, experts gave the baseline a slight edge on progress measurement, motivating the modular refinement that we apply in the integrated PHA system. A notable rater discrepancy emerged on user empowerment, for which experts preferred the HC agent (69.9% win rate), whereas end users ranked the two systems similarly (48.4%). This observation reinforces the value of incorporating both perspectives in the evaluation.

## PHA delivers holistic health support

**Fig. 5:**
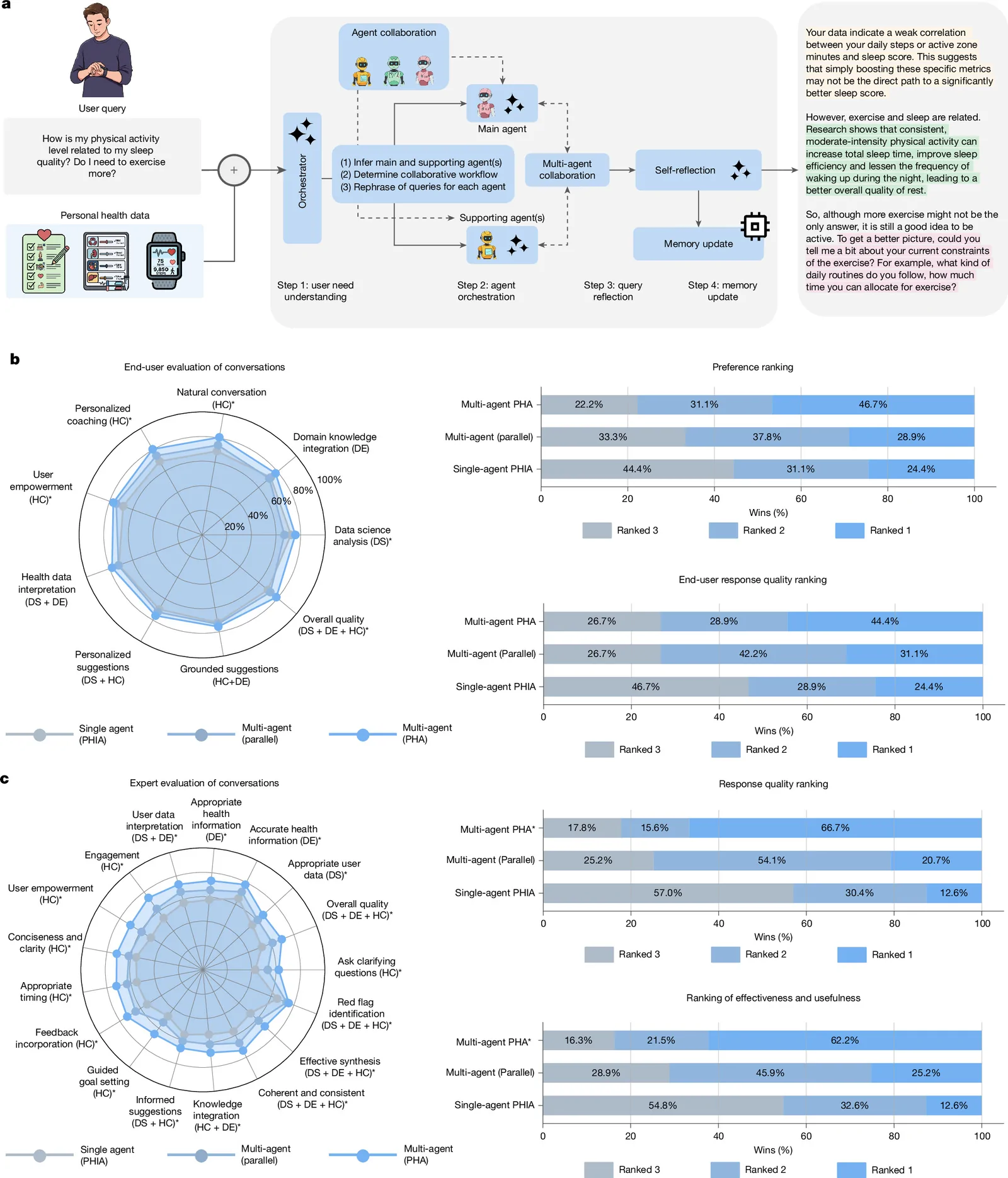
Schematic and evaluation results of the PHA. **a**, Overview of the collaborative workflow for the PHA. An orchestrator dynamically assigns tasks to the DS, DE and HC agents, managing a multi-step process of collaboration, reflection and memory update to synthesize a cohesive response (see Supplementary Section F.1 for prompting details). **b**, End-user evaluation (*n* = 45, matched with WEAR-ME-derived personas) of live multi-turn conversations shows the PHA as the modal top choice over both single-agent and parallel multi-agent baselines for overall quality and helpfulness. **c**, Health expert evaluation (*n* = 3) of these live conversation transcripts enhances these conclusions, revealing a substantially more pronounced preference for the PHA system over the baselines. Statistical indicator: *P < 0.05. Supplementary Section F.2 summarizes more evaluation details.

To address the multifaceted nature of personal health, we hypothesized that a synergistic collaboration between our specialized agents would deliver a superior user experience than monolithic or non-collaborative architectures. We therefore developed the PHA, an integrated system guided by four core design principles: address comprehensive user needs, adaptive support, low user burden and simplicity. The framework by the PHA features a central orchestrator that manages a sequential, iterative workflow involving user need understanding, dynamic agent task assignment, query reflection and a persistent memory module to maintain conversational context (Fig. 5a).

We evaluated this collaborative architecture against two strong baselines: a capable single-agent system (personal health insights agent (PHIA)) based on recent previous work^2^, and a parallel multi-agent system in which the three specialist agents respond independently. To compare the three systems, we recruited 45 end users with diverse health conditions, covering all WEAR-ME persona profiles (see Supplementary Table 35), as well as 3 health experts (Methods, PHA tasks).

### End users prefer collaborative PHA

Figure 5b summarizes the results from our end-user evaluation. The findings indicate that although all three systems were perceived as generally competent, our proposed PHA architecture was consistently favoured for providing a better overall experience. End users ranked PHA as their top choice nearly half the time (46.7% on overall preference and 44.4% on response quality), more frequently than the parallel multi-agent (28.9% and 31.1%, respectively) and single-agent PHIA (24.4% and 24.4%, respectively) baselines. The detailed rubric breakdown shows that PHA numerically outperforms both baselines on every rubric, with the advantage reaching post-hoc statistical significance on personalized coaching (*P*_PHA–PHIA_ < 0.001), natural conversation (*P* = 0.002), user empowerment (*P* = 0.032) and overall quality (*P* = 0.044, all GLMM with log link function and post-hoc Tukey’s honest significance test), as well as a PHA-versus-parallel advantage on data science analysis (*P* = 0.016). Per-rubric statistics are reported in Supplementary Tables 36 and 37. Beyond the ranking and scores, end users qualitatively praised the coherent and personalized conversation flow of the PHA. For instance, one participant stated that the PHA “did a really stellar job giving me personalized advice based off of what I was asking and off of my health scores and info, and it really honed in on a routine change that was unique to me and simple to fit into my current lifestyle and evening routine”. Another participant noted the PHA as “personable and knowledgeable and knew your history and dove right into it”. Although the baseline agents may possess the skills on domain knowledge and coaching, the iterative framework of the PHA allows the HC and DE agents to reflect on and contextualize the specific findings from the DS agent, providing a more deeply synthesized and insightful response.

### Experts favour the PHA framework

The evaluation from health experts, summarized in Fig. 5c, reveals a more pronounced advantage for the PHA system than the results derived from end users. Coach experts identified major quality differences that, although not immediately apparent to end users, are fundamental to providing safe and robust guidance and critical for the long-term effectiveness and user success of the system. Experts gave PHA the highest overall quality rating (72.8 ± 1.4% versus 64.3 ± 1.3% for parallel multi-agent and 54.4 ± 1.4% for PHIA, all pairwise *P* < 0.001, GLMM with log link function), with this advantage extending across nearly all 17 individual rubrics (Supplementary Table 38). Experts also ranked PHA as the best system in 66.7% of conversations on overall quality and 62.2% on effectiveness, more than twice the preference for the parallel multi-agent (20.7% and 25.2%, respectively) and roughly five times that of the PHIA (both 12.6%, all pairwise *P* < 0.001, Friedman test followed by post-hoc Wilcoxon signed-rank test with Holm–Bonferroni; Supplementary Table 39). The qualitative comments by experts echoed these results across coaching and data science dimensions. One coaching expert observed the PHA “consistently excelling at the behavioural aspects of motivational interviewing and… SMART goal creation”, whereas another highlighted the data-science foundation of the PHA, “synthesizing Fitbit metrics and lab results into a baseline narrative… [to] provide a necessary mirror for the user”.

### Scaling with synthesized conversations

To complement the live evaluations that are ecologically valid but bounded by participant time, we further leveraged the 50 WEAR-ME personas and synthesized 150 multi-turn conversations (50 × 3) between persona-instructed user LLM agents and the 3 systems, and recruited a separate cohort of 20 end-user generalist evaluators and 5 health experts to rate them using the same rubrics. We observed consistent patterns with the live findings in tasks 1 and 2 (see details in Supplementary Section F.3.3).

### Relative computational cost advantage

We also compared the number of LLM calls and response time across agents. Despite its iterative orchestration, the PHA requires fewer LLM calls and responds faster than the parallel multi-agent baseline (see Methods, ‘Computational cost’ section), demonstrating that the dynamic agent assignment by the PHA delivers superior user experience with greater computational efficiency than a static parallel approach.

### Overarching summary

Across the specialist and integrated evaluations, the modular sub-agents improved their targeted capabilities, and the orchestrated PHA outperformed the single-agent and parallel multi-agent baselines. Together, these findings support a modular, collaborative architecture that emulates the collaborative and specialized nature of human expert teams to support personal health and well-being.

## Discussion

### PHA advantages and user journeys

The PHA assigns data analysis, health-domain reasoning and coaching to specialist sub-agents coordinated by an orchestrator. This modularity allows each specialist capability to be developed and evaluated independently and enables agents or base models to be replaced without redesigning the overall workflow. Personal health questions often combine quantitative interpretation, biomedical context and behavioural change guidance; the orchestrator can recruit the relevant specialists and synthesize their outputs into one response. In the integrated evaluation, the PHA received higher ratings than the baselines on complex queries from both end users and experts: differences relative to the baselines ranged from 1.6 to 15.4% (average of 4.6%) for end users and 5.7 to 39.0% (average of 17.1%) for experts. Our contribution is an architecture grounded in empirically derived user needs. We do not aim to claim that our configuration is universally optimal. By releasing the PHA implementation and evaluation benchmark, we intend this work as a reusable foundation for the community to critique, swap in different agents or base models, and build stronger PHAs.

Our analysis shows that the PHA demonstrates strong competency across all four CUJs, with particular strength over existing systems in personal health data interpretation (CUJ2) and generating wellness advice (CUJ3). The framework excels at identifying patterns in wearable data, contextualizing these findings against established health guidelines, and producing actionable, personalized recommendations. However, addressing symptom assessment (CUJ4) presents a more complex and critical challenge. Although PHA outperforms general-purpose models in providing basic symptom information, its current architecture is not designed to offer definitive medical diagnoses or replace the judgement of a qualified health-care professional. Yet simply deferring such questions to clinicians may not address the delays that often motivate people to seek artificial intelligence support^48^. A future system could instead help users to record symptoms, identify patterns, prepare questions and navigate care while avoiding diagnostic claims^1^. Such a preparatory role requires prospective validation and safeguards against premature or inaccurate suggestions. Supplementary Discussion G provides additional interpretation and future directions for each single-agent and multi-agent collaboration.

### Ethical risks and future safeguards

The PHA is a research prototype for consumer wellness, not a diagnostic or clinical triage system, and should not replace professional care. Health-facing agents nevertheless risk reinforcing disparities when their models or evaluation data underrepresent populations. Future work should incorporate fairness metrics and evaluations across devices, languages, literacy levels and demographic strata. The prototype uses in-prompt disclaimers and conservative symptom-response policies as initial safeguards. Future versions should add persistent cross-turn notices, detect high-risk queries, and either refuse them or respond with an explicit handoff to a clinician so that the system does not delay care. These mechanisms require testing for false reassurance and unnecessary escalation, with clinician review of high-risk failures. Moreover, open-web retrieval creates a related risk of conflicting or low-quality evidence, and future deployment should restrict health retrieval to curated sources such as WHO, PubMed, NICE and CDC, and display source provenance beside each recommendation. Future systems could provide layered explanations showing which data and evidence informed a response. Finally, the research harnesses sandbox generated code, but real-world deployment requires systematic code validation and standardized privacy and security controls (for example, Health Insurance Portability and Accountability Act and General Data Protection Regulation).

### Limitations and conclusion

Our evaluation provides evidence of conversational quality and short-term task efficacy, not sustained behavioural change or improvement in longitudinal health outcomes. Those outcomes require multi-session deployments that retain previous conversation context, test behavioural interventions, and measure engagement and adherence over time. Longitudinal studies should recruit people responding as themselves and test whether benefits persist with long-term use. Our current evaluation also relies partly on LLM-based autoraters, which can be sensitive to ordering, formatting, length and source. Autorater conclusions should remain cross-checked against blinded human judgements as models and prompts change. Moreover, evaluation on WEAR-ME is Fitbit centric, limiting device generalizability. In our live studies, condition-stratified participant matching on WEAR-ME was intended to increase scenario relevance but cannot eliminate perspective-taking bias. Future evaluation across devices, languages, literacy levels and demographic groups, including older adults and people with disabilities, is therefore needed, and our open benchmark provides a basis for these extensions. Finally, computational burden is a constraint. PHA averaged 6.5 LLM calls and 205.3 s per response round, compared with 3.0 calls and 36.4 s for single-agent PHIA (Methods), which may limit scalability in resource-constrained settings. Optimized orchestration, parallel execution, caching and lighter routing models may reduce this cost.

Overall, this study evaluated DS, DE and HC agents together with their orchestration across 11 benchmark tasks and more than 1,350 h of end-user and expert assessment. The openly released implementation and benchmark allow other researchers and engineers to test different agents, base models, collaboration patterns and safeguards. These results provide a basis for further research of PHAs for daily health and well-being.

## Methods

### Real-world wearable and health data

To ground our agent evaluation with real-world validity, we evaluated the applicability and robustness of the framework against the WEAR-ME study, a large-scale (*n* = 1,165) effort to investigate metabolic health^43^. The overall study design is outlined in Extended Data Fig. 9a. As part of WEAR-ME, consented participants were asked to link their Fitbit account to Google Health Studies, authorizing the collection of their wearable data for the duration of the study and for up to 3 months before they joined. Once enrolled, participants were asked to (1) complete questionnaires covering demographics, health history and personal health insights, (2) schedule a blood draw with a quest patient service centre, and (3) wear their Fitbit or Pixel Watch throughout the day and night (for at least 3 out of every 4 days). In total, WEAR-ME contains multimodal data from each participant on their questionnaire answers, Fitbit records and blood test results. More details of the dataset are described in Supplementary Section B.2. The WEAR-ME study was conducted in full compliance with privacy regulations, with the study approved by the Advarra Institutional Review Board (IRB; #Pro 00074093). All participants provided informed e-consent for their data to be used in research and publications.

Throughout our evaluation, we leveraged the WEAR-ME dataset for various evaluation tasks (see tasks DS.1, DE.3, DE.4, HC.1, HC.2, P.1, P.2 and P.3 in Fig. 1c). As introduced later in this section, we contextualized our end-user and expert evaluations on a subset cohort of WEAR-ME for real-world validity. Working with clinicians, we identified the ten most common health profiles among the participants and then randomly selected five individuals representing each of those conditions (see the specific ten profiles and persona examples in Supplementary Section B.2). These 50 user personas were used to prepare evaluation samples for our agents’ multiple evaluation tasks (for example, task 3 and task 4 for the DE agent, and task 1, task 2 and task 3 for the PHA). The construction of the evaluation cohort is depicted in Extended Data Fig. 9b.

### Choice of base language model

We used the Gemini 2 family (Flash/Pro) of models^44^ for our experiments. Gemini has demonstrated strong performance on medical tasks^8,45,46^. To demonstrate that our method is model agnostic, we further evaluated our agent architecture on other LLM families across OpenAI’s GPT and Anthropic’s Claude (see result tables in Supplementary Data). In this work, our focus was not to train a new LLM but rather to design and construct the agentic system around a given base LLM. By selecting Gemini as the base model, we could ensure that (1) there was no training data contamination, and (2) no health data from real participants would be logged by the model and used for training future models. Although existing contamination was not a main concern, as a majority of our analyses involve novel datasets that are unlikely to be part of any LLM training dataset, choosing Gemini allowed us to verify that any testing of the models did not lead to data entering a future training set. We note that our design of sub-agents and the agent teams are agnostic to the base LLM. We expect that our proposed solutions will be generalizable to other LLMs.

### User-centred design of the PHA

We adopted a user-centred approach to identify and categorize the needs of end users for a PHA. Our process integrated insights from three layers of complementary data collection: real-world user queries, targeted survey responses and expert synthesis. Together, these layers form a coherent pipeline: we began by capturing broad, naturalistic user intent through consumer health queries; we then deepened this understanding through structured survey data that elicited the goals and satisfaction of users. Finally, we conducted expert workshops to distill and organize these findings into actionable requirements.

#### Consumer health queries

We compiled and analysed consumer health queries from Google Search, Gemini and the Fitbit community forum, from January 2024 to February 2025. In addition, in October 2024 through February 2025, we launched Insights Explorer through Fitbit Labs^49^. Participation was entirely voluntary and required users to explicitly opt-in and provide in-app consent for their data to be used for research and development. As part of this consented experience, participants were able to ask health queries and receive responses from a prototype generative artificial intelligence (AI) conversational agent. We composed a set of 1,370 de-identified user queries that represent a wide range of questions regarding daily health and wellness from real-world users. Supplementary Table 1 lists some examples of these queries.

#### Fitbit Labs survey data

To enrich the query data, we also collected survey data from participants (*n* = 555) in the Insights Explorer Fitbit Labs to ask about their personal health goals and sentiments when interacting with the agent. Specifically, the survey includes questions about user needs (for example, ‘what are you hoping to achieve by using [the prototype]?’, ‘which of the following aspects of health and wellness, if any, did you explore in [the prototype]?’), user experience and satisfaction (for example, ‘indicate any aspects of [the prototype] that have/have not met your expectations’), engagement and retention (for example, ‘what was the main functionality or benefits that you continued using [the prototype] for?’) and other user characteristics (for example, previous experience with wearable sensing, AI and conversational agents). Supplementary Table 1 lists the details of these laboratory surveys.

#### Expert workshop

To complement users’ perspective, we further conducted a 1-h critical user journey workshop with experts across user experience, product, research and engineering (*n* = 14) to categorize the data collected. The workshop procedure focused on summarizing the user journey goals and the specific associated tasks. After collecting multiple experts’ perspectives, we adopted a standard thematic analysis process^50^. Two researchers independently coded the same subset of these queries and all survey data to jointly develop a codebook of hierarchical user journeys (categories and sub-categories). The same researchers then categorized the rest of the user queries into these categories and expanded the codebook as needed.

Our analysis of user needs identified four CUJs, spanning data interpretation, knowledge seeking, wellness advice and symptom assessment (see results). Satisfying these diverse requirements demands more than a single monolithic system and calls for a set of specialized, complementary capabilities.

Our agent development approach is therefore grounded in both the cognitive theory of modular cognition^51^, which posits that the mind uses specialized subsystems for different tasks, and the empirical success of human health teams, in which experts with complementary skills collaborate. Inspired by this, we deconstructed the problem space and propose a multi-agent personal health assistant that brings together three core roles, each embodied by a specialized sub-agent: data analysis, health domain expertise and behavioural coaching, working in concert to emulate a professional health support team.

### DS agent development

This agent mainly focuses on addressing user queries about personal data. Existing research has explored the ability of LLMs to answer data science questions^2,28–35^, but their capacity to interpret open-ended, personalized data inquiries and perform statistically sound data analyses remains unclear, and there is a lack of open benchmarks assessing their reliability in this area. We developed the first personal open-ended data science agent capable of analysing both personal and population-level time-series wearable data to provide numerical health insights. Our agent enables users to ask diverse, open-ended and personalized questions about their health data and provide meaningful, data-driven responses, filling a critical gap in personalized data analysis. We also open-sourced an evaluation benchmark for these capabilities. Example conversations between a user and the DS agent are shown in Supplementary Fig. 1.

We focused on a set of capabilities for the DS agent that are useful, yet tractable to evaluate^2,30,34^. These capabilities are informed by the types of user interactions and data that are expected in the context of the CUJs (presented in the results). Broadly, we define the capability of the DS agent to address queries that involve analysis of numerical data (for example, wearable streams and medical records), with the goal as obtaining statistical outcomes that align with the query intent and context surrounding those statistics, such as timeframe, type of statistics, assumptions, among others. Specifically, the capabilities of the agent are as follows.

#### Decompose an open-ended query into a structured analysis plan

For broad or ambiguous queries often posed by end users without data science expertise, the DS agent should translate them into appropriately specific, unambiguous, tractable queries and analysis plans by:

1. Identifying conceptual variables^52^, which recognizes the concepts in the question that can and should be represented in the data (for example, representing fitness as average daily steps and resting heart rate for a query such as ‘have I gotten more fit over the past few months?’).
2. Formulating the correct and exact data transformation steps to operationalize the variables across multiple tables of user data and population-level data tables. This involves the following optional steps:
  - Filtering relevant subsets of data (for example, filter for activities in the past 3 months)
  - Cleaning messy data (for example, handling missing or misrecorded wearable data)
  - Joining multiple tables (for example, joining the activities data table with the daily summary data table on the same day)
  - Aggregating data (for example, getting average steps and resting heart rate grouped by month)
3. Recognizing, highlighting or addressing missing or insufficient data to prevent unreliable conclusions.
4. Applying appropriate statistical tests and calculations on the basis of the intent of the user.

Note that given the open-ended nature of user queries, multiple interpretations and answers may be valid. Accordingly, in one single interaction, our DS agent is designed to provide at least one appropriate response, rather than an exhaustive enumeration of all possibilities. Users can have follow-up conversations to explore other options with the agent. In the case in which the data in user queries are not available, the agent might check the availability of the data with users and request data from users, if needed.

#### Compute accurate numerical results through code generation, execution and feedback

On the basis of a precisely specified analysis plan (that is, one without any ambiguity for alternative methods and models^53^), the agent should be able to generate, execute and debug code that follows the exact specifications of the analysis plan, such that it can execute bug-free code and compute accurate results.

On the basis of the delineated capabilities, the architecture of the DS agent is designed as a modular, two-stage pipeline: (1) analysis plan generation and (2) code generation with iterative execution^54^. This decomposition is critical for mitigating the risk of factual inaccuracies and logical errors common in end-to-end generation for complex data tasks^55^. It separates the semantic reasoning of ‘what to do’ from the syntactic task of ‘how to do it’.

The first stage translates an open-ended natural language query into a precise, structured analysis plan. To ground its reasoning, the planner is provided with a rich contextual summary of the available data, including table schemas, column descriptions with data types and semantic tags, and summary statistics^56^. The output is a structured natural language paragraph that explicitly outlines the conceptual variables, required data transformations (for example, filters, joins and aggregations), and the chosen statistical model, directly instantiating the components listed above.

The second stage receives this structured plan and is tasked with generating executable code. This focus on a well-defined specification allows the LLM to leverage its strong code generation capabilities^55^. The generated code is then executed in a sandboxed environment. We used an iterative refinement loop: if execution fails due to syntactic errors or runtime exceptions, the agent re-invokes the stage with the original query, plan and the error message as feedback. This self-correction cycle, which has been shown to improve robustness^57^, continues until bug-free execution is achieved and a numerical result is produced.

Figure 2a illustrates the architecture enabling these functions in the DS agent. The details of agent prompt and architecture are included in Supplementary Section C.2.

### DE agent development

This agent aims to answer any queries that are related to medical knowledge or daily symptoms. Although previous work has evaluated the medical knowledge of LLMs^36–38^, many essential skills of an ideal domain health expert are underexplored, such as interpreting data within contexts, for example, population and environmental factors, and performing summarization and reasoning across data modalities from wearables and medical records. We built a DE agent equipped with a suite of tools to address these challenges. The agent integrates the wearable data of users, medical records and contextual data to generate accurate, relevant responses to complex health inquiries. It advances the state-of-the-art LLMs by providing nuanced multi-modal interpretations that consider the broader context of personal health. Example conversations with the DE agent are shown in Supplementary Fig. 2.

We designed the DE agent to function as a specialized cognitive layer for health applications. Its purpose is not merely to retrieve information, but to synthesize, contextualize and validate it against authoritative sources and patient-specific data. By acting as a focused and reliable source of medical expertise, the DE agent aims to minimize misinformation risk and translate complex data into clear, actionable insights, serving as a critical foundation for our multi-agent design. (These capabilities were investigated in a controlled, academic context; they do not indicate real-world products that require an evaluation of complex regulatory landscapes.)

To fulfil this role, the DE agent needs to move beyond the simple query–response behaviour of standard LLMs. It should be equipped with a set of distinct yet interconnected skills that mirror the reasoning of a human clinician. This involves not only accessing factual knowledge but also applying it within the context of the specific health status and history of an individual. To formalize this multifaceted expertise, we defined and evaluated the core competencies of the agent across four foundational capabilities:

1. Accuracy of health information. The DE agent should provide accurate and reliable medical knowledge spanning diverse personal health and clinical domains, ensuring that information is consistent with expert-level understanding.
2. Diagnostic reasoning acumen. The agent can perform differential diagnostic reasoning by generating and ranking plausible diagnoses on the basis of the symptoms and relevant medical history of the user conveyed through natural conversations.
3. Contextualization and personalization efficacy. The agent should contextualize and personalize health information and advice, tailoring outputs to the specific demographic profile, pre-existing conditions and health goals of a user to ensure relevance, clarity and safety.
4. Multimodal data synthesis and reasoning. The DE agent should synthesize and reason over heterogeneous data sources, generating coherent and informative summaries from complex real-world inputs that integrate wearable data (for example, simple statistics or complex outcomes computed by the DS agent) with medical records (for example, laboratory results and medical history).

The DE agent uses a multi-step planning, reasoning and acting architecture^58^, as shown in Fig. 3a. The agent receives a user query, wearable data and medical records as input. The agent processes the query and chooses actions from a set of tools that include querying: Web Search (https://www.google.com), the NCBI (https://www.ncbi.nlm.nih.gov) application programming interface (API) and the DataCommons API (https://datacommons.org). A Python Sandbox is also available for executing APIs.

The multi-step reasoning framework enables the agent to address complex queries by interleaving reasoning with tool use. For any given query, the agent follows a reason–investigate–examine cycle: it begins with a reasoning and thinking stage, in which the agent decomposes the health query posed by the user into a series of verifiable steps, such as establishing a clinical baseline for a vital sign, finding literature to connect symptoms or planning to analyse trends in personal data. On the basis of the reasoning from the previous step, the agent chooses the most suitable investigations by selecting the appropriate tool to execute its plan—for example, querying DataCommons for population statistics or NCBI for medical studies. The agent then examines the results of the previous steps, which informs the next cycle. This iterative cycle continues until the agent has synthesized sufficient evidence to construct a comprehensive answer.

This dynamic, iterative process creates a transparent and auditable reasoning trace. By synthesizing facts from authoritative sources with the personal health data of the user, the agent ensures its final answer is not a black-box output but is instead a grounded, evidence-based conclusion. This architecture is therefore essential for fulfilling the core capabilities of the agent: providing accurate, personalized and reliable health insights. Supplementary Section D.1 details the prompt setup for the DE agent.

### HC agent development

The HC agent aims to provide personalized health and wellness advice, and to help users set up goals and motivations via conversations. Although previous studies have initiated explorations into the application of AI in personal coaching^39–41^, to our knowledge, our agent is the first to support open-ended coaching conversations across a wide range of general wellness topics. Building on coaching expert insights, we considerably enhanced the coaching skills of the agent, including motivational interviewing and personalized health recommendations. This agent adopts a modular structure to optimize the conversation flow, balancing information gathering, active listening and recommendation delivery to support a smooth and effective coaching experience. Example multi-turn conversations with the HC agent can be found in Extended Data Figs. 3 and 4.

To inform the design of our HC agent, we conducted a formative interview study with 11 health experts (6 male individuals and 5 female individuals, 24–45 years of age). Experts were selected from various domains, such as sleep, fitness and life coaching. These experts hold advanced degrees in their respective fields (with at least 5 years of professional experience) and had a deep understanding of best practices in health coaching. The semi-structured interviews, each lasting approximately 1 h, focused on eliciting expert insights regarding the critical elements of effective health coaching, particularly focusing on the interplay of conversational substances and styles. Two authors conducted thematic analysis and independently developed theme codebooks and iterated until convergence. This qualitative data provided a foundational understanding of nuanced coaching expertise, leading to the following core HC agent capabilities.

For goal and purpose identification, the HC agent needs to understand the goal and the motivation behind the goal of the user, maintain a goal-oriented conversation, and bring the user back to the main goal when they are deviating off the topic.

For active listening, the agent should provide occasional summary and paraphrasing of the state of conversation to circle back to users and ensure correct understanding and goal alignment.

For context clarification, our HC agent should collect and understand the constraints, preferences and previously attempted solutions of users.

For user empowerment, the HC agent needs to empower the user by building trust, guiding them to navigate their own paths when exploring potential solutions, and providing supportive reinforcement when they make positive progress towards their goals.

for personalized and concrete coaching recommendations, the HC agent should provide high-quality customized recommendations that are specific, measurable, attainable, relevant and time-bound (SMART), helping the user to create an actionable plan^59^.

For user feedback incorporation, the agent should be able to provide initial recommendations mid-conversation, while explicitly leaving space for user input, and subsequently updating its suggestions on the basis of that feedback.

Conventional single-prompt architectures often fail to simultaneously address the diverse coaching capabilities (HC1–HC6), struggling in particular with the demands of mixed-initiative dialogue. To overcome these limitations, we designed a modular architecture for the HC agent that embodies expert coaching principles. As illustrated in Fig. 4a, this design enables the agent to dynamically adapt its conversational flow on the basis of user input and to strategically time its recommendations.

At its core, the HC agent is designed to emulate a professional coach. To this end, we developed the personalized coaching module grounded in the principles inspired by motivational interviewing^60^, a client-centred approach that enhances motivation by exploring and resolving ambivalence. This module operationalizes key coaching behaviours, such as active listening (HC2) and user empowerment (HC4), to foster engagement. It also establishes a structured, proactive line of questioning that instructs the agent to first identify the goals of the user (HC1), then explore their context and constraints (HC3), and finally solicit feedback (HC6) before providing a recommendation (HC5).

#### Conversation flow modules: recommendation module and conclusion module

Although a single prompt can include all these aspects, our pilot testing revealed that such a prompt-based approach struggles to maintain conversational balance. Such agents tended to either offer premature recommendations before sufficient context was gathered, or become fixated on questioning, failing to transition to actionable advice. To address these failure cases, we introduce two specialized modules to manage the conversational state: the recommendation module dynamically determines the opportune moment to provide advice. It is responsible for keeping the conversation on track (HC1) and explicitly confirms with the user if enough context has been gathered before transitioning to a recommendation (HC3, HC6). Meanwhile, the conclusion module identifies when a conversation has reached a natural end point and ends the interaction gracefully with a summary, reinforcing the key takeaways (HC5).

The final architecture of the HC agent is a combination of the personalized coaching module to steer the coaching process with personalized information, and the two conversation flow modules for appropriate recommendations and conclusion to align the flow of the interaction with the queries inputs and goals of a user. More prompting details are described in Supplementary Section E.1. Overall, this modular architecture ensures that the agent robustly expresses the full range of expert coaching themes (HC1–HC6) in a dynamic, user-adaptive manner.

### PHA development: multi-agent collaboration

The three sub-agents have complementary skill sets and often need to collaborate together to provide appropriate support regarding a query by a user. A central orchestrator manages this collaboration. It receives the initial query by the user, parses it and determines which agent (or agents) is best suited to respond. It decomposes the query into sub-tasks, routes them to the appropriate sub-agents, synthesizes the responses into a cohesive, user-friendly answer, and then reflects and iterates on it before responding to users^42^. Extended Data Figs. 5–8 demonstrate a multi-turn conversation example with PHA.

Our design is grounded in both cognitive theory and empirical evidence from human collaboration. Theoretically, we drew from the concept of modular cognition, which posits that the mind comprises specialized, independent subsystems for different cognitive functions^51^. We applied this by treating each agent as a distinct cognitive module with its own knowledge and processing capabilities. Empirically, this mirrors the structure of effective human expert teams, in which members with complementary expertise collaborate to achieve a more robust outcome than any individual could alone. This modular approach not only enables a more holistic response to complex health questions but also enhances explainability. By tracing outputs back to the specific function of each agent, the decision-making of the system becomes more transparent and interpretable, an essential requirement in sensitive health contexts.

To further instantiate our design, we propose a set of four principles across capability, user experience and architecture to guide the design of our PHA.

1. Address comprehensive user needs. Our user-centred analysis has revealed the wide range of health and wellness needs of users. This calls for a comprehensive set of agent capabilities to fulfil these needs. Single-agent systems, even with rich capabilities (for example, see a previous study^2^), may not be able to cover all cases.
2. Adaptive support. We have previously identified that user queries may require different combinations of the three agents (Supplementary Table 1). A multi-agent system needs to dynamically assign agents to be adaptive towards the needs of users. This suggests that static multi-agent architectures, such as voting^23^ and debating^26^, may not fulfil the need.
3. Low user burden. When a system has access to the wearable data and medical records of users, asking users what can already be inferred from the data would not only increase user burden but also impact user trust of the intelligence of the system. For example, the system should avoid asking questions such as ‘how long did you sleep last week?’ (a typical question from the HC agent when it acts without the DS agent) when it can easily compute the data from the wearable data record (via the DS agent). The system needs to have a chain of agents for deeper reflection rather than simply a set of parallel agents for straight response, for example, voting^24,61^, to fully leverage and potentially follow up on the output from each agent.
4. Simplicity. When the capabilities of an agent are similar, simpler architecture has advantages of lower latency, less computation cost, as well as an easier pathway of maintenance and extension^62^. Although more complex, hierarchical structures can be effective for specific goals such as ensuring clinical safety^63^ or mimicking interdisciplinary care teams^27^, they may represent unnecessary complexity (for example, leading to unappealing long inference times).

These four guidelines indicate the limitations of existing multi-agent architectures and guide the implementation of the multi-agent collaboration framework of PHA.

Following theoretical foundations and empirical evidence, together with the design principles, we present our multi-agent framework of PHA, as illustrated in Fig. 5a. It consists of an orchestrator (Gemini based) that manages a sequential and iterative workflow between the three specialists agents: the DS agent for data science analysis, the DE agent for domain expertise, and the HC agent for health coaching (improved on progress tracking based on the results in the HC agent evaluation). This collaborative process involves four key steps. The implementation details can be found in Supplementary Section F.1.

1. User need understanding (PHA.1 and PHA.2). When a user query arrives, the orchestrator first performs the crucial step of user need understanding. It analyses the semantics of the query and infers the primary goal of the user. This classification aligns with the four CUJs (for example, seeking data insights and wellness advice). This ensures that the system can select the most appropriate agent (or agents) for the need of the user, addressing the principles of covering comprehensive user needs (PHA.1) and providing adaptive support (PHA.2).
2. Agent orchestration and task assignment (PHA.2 and PHA.4). On the basis of the inferred user need (or needs), the orchestrator dynamically assigns a ‘main agent’ (one of the three agents) to take the primary responsibility for the query. It then determines whether any ‘supporting agents’ are required to provide supplementary information. For instance, a query such as, ‘how can I improve my sleep based on last week’s data?’ would assign the HC agent as the main agent, supported by the DS agent to first analyse the sleep data. The orchestrator decomposes the main query into specific, targeted sub-queries for each assigned agent. Moreover, to facilitate the task assignment, we built on the four CUJs and developed a set of collaboration examples that denote agent assignment for each sub-category (see Supplementary Table 1). These examples are provided to the orchestrator for reference. This dynamic needs-based assignment fulfils the principle of adaptive support (PHA.2) while maintaining architectural simplicity (PHA.4) by avoiding a complex hierarchy.
3. Query reflection (PHA.3). Unlike parallel architectures in which agents work in isolation, PHA uses an iterative process that allows for reflection. Outputs from the main agent and the supporting agents are not sent directly to the user. Before finalizing the response, the main agent performs a ‘self-query reflection’ step. Inspired by iterative self-refinement frameworks^24,64,65^, this internal check assesses the synthesized information for coherence, completeness and accuracy, ensuring that all available data from the health data of the user is leveraged. By preventing the system from asking for data it already has (for example, ‘how many hours did you sleep?’ or ‘when did you wake up?’), this process upholds the principle of low user burden (PHA.3).
4. Memory update (PHA.1, PHA.2 and PHA.3). After each interaction turn is completed and a response is presented to the user, the orchestrator updates a persistent memory module. This memory logs key entities from the conversation, including generated insights from each agent, user-stated goals, identified barriers and preferences. This running context enables the system to maintain conversational coherence over multiple turns and personalize future interactions more effectively. The memory module supports the agent team in addressing comprehensive needs (PHA.1) over time, providing adaptive support (PHA.2) as goals evolve, and ensuring low user burden (PHA.3) by retaining context across sessions.

### Agent evaluation framework

To systematically evaluate our agent framework and identify the strengths and gaps in the design of each component, we created the evaluation framework shown in Fig. 1b. We took a mixed-methods approach and conducted a set of studies that examine a comprehensive set of factors:

1. Interaction turn: single and multi-turn interaction.
2. Data modalities: agent processing with both single-modality and multi-modality data.
3. Evaluation setup: automatic and human evaluations of conversations.
4. Human perspective: perspectives of health experts and end users on multi-turn conversations.

This multi-faceted approach provides a holistic understanding of the performance of the AI agent and is the most comprehensive evaluation of a consumer AI health agent thus far.

Specifically, our framework delineates and assesses the core capabilities of each component. The DS agent is evaluated on its two-stage process of (DS.1) generating robust analysis plans and (DS.2) translating them into accurate, executable code. The DE agent is assessed across four key competencies: (DE.1) answering common health questions, (DE.2) handling diagnostic conversations, (DE.3) personalizing answers to medical questions on the basis of context and information about the user, and (DE.4) interpreting and reasoning with multi-modal health data (wearables, laboratory results, demographics and health surveys). The capability of the HC agent for providing a personalized coaching experience is evaluated from (HC.1) its effectiveness from the perspective of the end user, and (HC.2) its fidelity to human expert coaching principles. After connecting all three sub-agents, the final multi-agent PHA system has the comprehensive set of skills to support various end-user health queries at a human health expert level. It is assessed on its holistic conversational performance from complementary viewpoints of both (P.1) end users and (P.2) experts.

### DS agent evaluation

To ensure that our DS agent produces statistically robust and numerically accurate answers, we independently evaluated the two core components in two tasks.

#### Task 1: analysis plan generation

Given an open-ended query involving data analysis (for example, ‘am I getting more fit recently?’), we measured the ability of the agent to decompose and/or expand the query into a precise and robust statistical analysis plan. The plan should align with the intent of the query and appropriately involve the analysis of personal health data.

In this paper, we focused on the data analysis on the wearable data from the WEAR-ME dataset^43^. In particular, the DS agent was given access to the following data tables for each individual user: (1) a daily summary table with daily activity and sleep wearable data, (2) an activities table in which each row represents a recorded physical activity (for example, running, biking, swimming, among others), and (3) a population summary table that provides daily wearable percentiles stratified by gender and age group. We have included the full data schema of all tables used by the DS agent in Supplementary Section C.1.

An analysis plan involves many decisions that contribute to the robustness of the final result, such as handling missing data, choosing a statistical model and operationalizing constructs^53,66,67^. Evaluating these decisions with human experts is prohibitively expensive. To scalably evaluate a wide range of analysis approaches, we developed another LLM as an autorater^68,69^ tested against human expert raters to ensure its reliability.

*Evaluation rubrics and point-detection metrics.* To enable this, we first developed a detailed analysis plan rubric in consultation with a panel of four data science experts. The rubric is designed for objective assessment across six key dimensions:

1. Timeframe: does the analysis consider a specific timeframe (for example, past month, past 6 months) when it helps to personalize and align with user intent?
2. Data transforms: does the approach correctly reference existing columns and apply any necessary data transformations, without logical errors, in a way that others can reliably reproduce?
3. Data sufficiency: does the approach reasonably and clearly assess whether there is enough data, both in terms of available rows and non-missing cells within columns, to support the intended transformations, calculations and statistical analyses?
4. Summary statistics: does the approach appropriately calculate summary statistics where helpful, and are the chosen statistics reasonable given the context and question being answered?
5. Statistical tests: does the approach appropriately apply statistical tests when helpful, with clear, reproducible methods, reasonable test choices and proper consideration of distributional assumptions?
6. Overall alignment: finally, given the evaluation of the above dimensions, does the approach align with the intent of the user?

Our final rubric consists of 26 items arranged in a conditional hierarchy. This structure means some items only apply if a preceding one is satisfied; for example, we only assessed the appropriateness of a statistical test if the initial hypotheses are sound. To quantify quality, we used a point-deduction system in which errors or omissions lower the final score. Because of this hierarchy, a maximum of 16 points can be deducted in any single evaluation. The detailed rubric development process and point system are provided in Supplementary Section C.3.1.

*Query–analysis plan benchmark.* Evaluating the quality of generated analysis plans and developing a scalable autorater requires a reliable benchmark. To this end, we created an expert-rated dataset of 141 unique query–analysis plan pairs. These pairs were generated using various LLMs (Gemini 1.5–2.0 and GPT-4o) across 100 distinct health queries (sampled from the end-user health query set collected in the user-centred design study). Ten expert data scientists (eight male individuals and two female individuals, 25–45 years of age, all with PhDs in computer science and with 5–20 years of experience) spent 47 h in total and provided 354 approach annotations based on the rubrics defined above (each query–analysis plan pair was annotated by 2–4 experts), leading to a total of 6,157 rubric item annotations. This expert-rated dataset demonstrated a moderate level of inter-rater reliability, with an average agreement of 80.2% and a Bennett’s S of 0.622 (ref. ^70^). Detailed statistics can be found in Supplementary Table 4.

*Development of a reliable LLM autorater.* Using this human-rated dataset, we developed and validated an autorater based on Gemini 2.0 and 2.5 Flash. The dataset was split into development and test sets, with the test set comprising 96 evaluations of 25 unique queries. The development set with 75 queries was then used to train the autorater (the specific process is detailed in Supplementary Section C.4). Our final autorater achieved an intraclass correlation coefficient(3,1) of 0.838 when compared with the scores assigned by human experts, indicating high reliability for scaled evaluation^68^.

We then assess the quality of the DS agent-generated analysis plans using this developed autorater, and compared them to the baseline Gemini 2.0 Flash model. The plan generation prompt templates for both the DS agent and the baseline are listed in Supplementary Section C.2.

#### Task 2: code generation and execution

Given a precisely specified analysis plan from task 1, this task evaluates the ability of the agent to translate that plan into code that is not merely executable but also functionally correct and robust. The primary goal is to measure the fidelity of the implementation to the plan and the numerical accuracy of its results, especially its ability to handle key data science challenges. This includes managing edge cases (for example, limited data and complex data transformations) and provide a correct answer to the question posed by the user.

To objectively evaluate the code, we manually converted the analysis plan into a precise function header and doc string to the agent following previous work^71^. The code was written in Python in a sandbox environment with libraries such as numpy, pandas and scipy. The details of the implementation are included in the Supplementary Section C.2.

In particular, a group of seven data scientists (five male individuals and two female individuals, 22–45 years of age, with 5–10 years of experience) were recruited for the code generation evaluation task. They were provided with 25 query–approach pairs with high ratings sampled from task 1 and were asked to formulate each of them as a coding problem with a function header and doc string (clear return output structure). Next, experts were asked to write the function implementation and associated test cases as unit tests. Going beyond simply checking for executable code, these unit tests were designed to rigorously assess the correctness and robustness of the underlying logic. They evaluated the ability of the system to handle key analysis cases, including managing limited data, performing complex data transformations, applying appropriate statistical methods and, ultimately, providing an accurate answer to the question posed by the user. In total, we collected code detailing comprehensive test cases for 173 unit tests from these 25 query–approach pairs, with the number of tests per pair varying on the basis of the complexity of the query (75 h of human effort). Examples of unit tests are shown in Supplementary Section C.3.2. Our DS agent and the base Gemini 2.0 Flash model were assessed for their code generation capabilities using these 25 test suites.

The pass rate was determined by calculating the ratio of successful test cases (across all 173 tests in 25 suites) to the total number of test cases. If code execution fails, the model will receive error messages and retry up to five times.

#### Model-agnostic check across LLMs

To probe whether the performance gains attributable to our agentic architecture are contingent on the Gemini backbone or generalizable across base LLMs, we conducted an additional ablation in which the same DS agent architecture and prompts were instantiated on top of state-of-the-art LLMs (GPT and Claude family of models). We evaluated each configuration on both DS task 1 (analysis plan generation) and DS task 2 (code generation and execution) using identical queries, rubrics, autoraters and unit-test suites as in the main experiments. For each SOTA base LLM, we have reported both (1) the base model without our agentic scaffold, and (2) the base model wrapped by our DS agent architecture. The purpose of this analysis is not to claim a new peak result on either LLM, but to demonstrate that our proposed architecture is model-agnostic and generalizes beyond the Gemini family. Detailed results are reported in the Supplementary Section C.5.3.

### DE agent evaluation

We conducted a systematic evaluation process to evaluate the four capabilities of the DE agent in four independent tasks.

#### Task 1: health knowledge and factuality

We evaluated the ability of the agent to accurately answer nuanced medical and wellness questions (DE1). The evaluation consists of a comprehensive suite of expert-level multiple-choice questions covering personal health and medical knowledge domains.

Guided by clinical relevance and our user study, we selected endocrinology, cardiology, fitness and sleep medicine as the main domains to evaluate for this task. These domains map directly to real-world daily health needs, including both high-prevalence chronic conditions (endocrinology and cardiology) and high-interest preventative wellness topics (fitness and sleep). Furthermore, they provide a scientifically robust testing ground; their complexity requires deep reasoning over case vignettes, allowing us to assess performance beyond simple fact retrieval. Although a broad medical knowledge base is essential, the foundational language model of the agent has already demonstrated strong performance on general medical benchmarks (for example, see previous articles^36,45^). Our evaluation is therefore specifically designed to assess the ability of the DE agent to apply this foundational knowledge in challenging, specialist-level domains that are of high interest to the public, rather than re-validating the established capabilities of the base model.

To this end, using board certification and coaching examination questions provides a standardized and quantitative metric for evaluating the ability of the DE agent to use its medical knowledge to reason over case studies mirroring real-world scenarios. Specifically, we took four curated multiple-choice question datasets totalling more than 2,000 test questions to assess knowledge and capabilities at answering expert-level health questions:

- Endocrinology examination: leveraging StatPearls’ ‘American Board of Internal Medicine: Endocrinology, Diabetes, & Metabolism Exam’ preparatory quizzes^72^, we selected 570 questions at random from all available levels of difficulty (173 ‘expert’, 198 ‘difficult’ and 199 ‘moderate and easy’ questions).
- Cardiology examination: we created a list of 399 Cardiology Board Certification questions using BoardVital’s ABIM-based preparatory question bank^73^. We randomly selected questions from all difficulty levels (100 ‘hard’, 199 ‘moderate’ and 100 ‘easy’ questions).
- Fitness ‘coaching’ certification examination: compilation of 542 questions by Khasentino et al.^8^ from multiple question banks that emulate examination content for the Certified Strength and Conditioning Specialists.
- Sleep medicine examination: a curated dataset of 634 multiple-choice questions from BoardVitals sleep medicine Maintenance of Certification board review question banks by Khasentino et al.^8^.

We compared our DE agent against the same backbone model, which acted as the baseline without the agentic framework.

#### Task 2: diagnostic conversations

This task evaluates the ability of the agent to perform differential diagnosis through conversational reasoning (DE2). Unlike previous work that often dealt with complex case studies^4,74,75^, we focused on the performance of the agent when presented with common symptoms that a user would typically self-report. The goal is to assess the quality and accuracy of the generated differential diagnosis on the basis of these realistic scenarios.

We conducted a large retrospective observational study to collect health case self-reports from consumers (*n* = 2,000, 18–65 years of age, IRB-approved #GH-SCD-001). The collected data were de-identified and utilized to evaluate the performance of the DE agent. Participants had experienced symptoms in one of 39 pre-defined categories (see Supplementary Section D.3.2 for the full list) within the 3 months. Participants completed an online survey detailing their previous health events, their symptom descriptions in natural language, any online information-seeking behaviour they engaged in and the ultimate diagnosis they received.

We compared our DE agent against a baseline that we re-implemented: a Gemini-based state-of-the-art DDx agent^4^. To generate predictions, we inputed all self-reported symptom descriptions into each model and prompted for the top ten most likely diagnoses (refer to Supplementary Section D.1.4 for the exact prompts). To evaluate the performance, we leveraged a separate Gemini 2.5 Pro as an autorater (prompt details in Supplementary Section D.1.4) that systematically compared the ground truth against the texts of predictions ranked by the models.

#### Model-agnostic check across LLMs

Similar to the DS agent evaluation, we also evaluated the DE agent architecture with other SOTA LLMs, GPT and Claude models, on task 1 and task 2. Detailed results are reported in the Supplementary Section D.3.3. Our results show that our agentic framework provides additional domain expertise and reasoning, resulting in better performance on diagnostic conversations and medical examinations, agnostic of the backbone LLM in use.

#### Task 3: contextualized and personalized health responses

Generic medical advice, although potentially accurate in isolation, can be inappropriate or even harmful if it fails to account for the unique health profile of an individual. For instance, encouraging a vigorous exercise plan could be beneficial for most people but may pose major risks to someone with certain cardiovascular conditions. Therefore, a critical capability of a reliable health agent is its ability to tailor responses to user-specific queries, integrating contextual information to provide personalized, trustworthy and actionable guidance. This evaluation assesses the ability of the DE agent to personalize responses to health queries involving key contextual information (DE3).

We curated a set of 50 health-related questions that are modified from the query set and designed to elicit personalized responses. To test the contextual reasoning of the models, each query was augmented with specific personal information falling into two categories:

- Demographic differences: queries included details such as age, biological sex and race or ethnicity, which critically influence health risks, disease presentation and preventative care recommendations (for example, screening schedules and risk stratification).
- Existing health conditions: queries were framed within the context of prevalent chronic conditions, including diabetes, cardiovascular disease, and impaired kidney, thyroid or liver function. Providing safe and relevant advice in these scenarios requires the agent to reason about potential contraindications and comorbidities.

We generated responses to these contextualized queries from both our DE agent and the base Gemini 2.0 Pro model. To mitigate presentation bias, all responses were programmatically normalized to a standard format. A cohort of 17 end users (25–50 years of age) were recruited and evaluated the model responses in a randomized order in which they were blinded to the model condition. In total, end users spent 13 h on the evaluation. Inspired by a previous article^76^, our evaluations used precise Boolean rubric questions, as well as side-by-side comparisons to assess dimensions of ‘personalization’, ‘relevance’, ‘credibility’ and ‘trustworthiness’. See Supplementary Table 9 for the specific evaluation rubric.

#### Task 4: multimodal personal health data reasoning

This task evaluates the ability of the agent to synthesize and reason over diverse, multimodal personal health data (wearable data and medical records) to generate comprehensive health summaries (DE4). Unlike simple information retrieval, this task demands a higher level of cognitive synthesis that mimics the ability of a clinician to connect disparate data points, identify trends and highlight potential concerns. The objective is to produce a concise and actionable overview of the health status of an individual proactively, without a specific user query or chief complaint.

To evaluate the capabilities of the agent for this task, we leveraged the following data modalities from participants from the WEAR-ME data:

- Unstructured text: for each participant, we combined all contextual and background information collected during onboarding survey as one string.
- Structured tables: this included tabular data representing wearable-derived digital measures, such as resting heart rate, heart rate variability, among other measures.
- Laboratory result documents (PDFs): given that most users will have access to an electronic document of their laboratory tests, we converted the laboratory results of real participants to PDF documents for input to the compared models.

We sampled 30 participants from our persona pools with an equal distribution across 10 health profiles. We then prompted our DE agent and the baseline Gemini model to generate a comprehensive health summary for each participant on the basis of their complete data profile. A panel of five expert clinicians (MDs, two female individuals and three male individuals, 30–60 years of age) then independently evaluated the quality of these generated summaries in a side-by-side, blinded comparison, with a total of 77 h of human effort. The clinicians used a detailed rubric to assess dimensions such as clinical relevance, accuracy of interpretation and the actionability of the summary (see Supplementary Table 10 for the full rubric).

### HC agent evaluation

To assess the capabilities of the HC agent, we conducted a comprehensive evaluation centred on real-world user interactions across various health and wellness topics. For a holistic assessment, our evaluation incorporates perspectives from both end users and coaching experts.

#### Task 1: coaching capabilities from end-user perspectives

The practical success of an LLM-based health coach hinges on the experience of the end user. An agent is effective only if users perceive it as engaging, competent and preferable to alternatives. This evaluation, therefore, investigates user sentiment and perception of the HC agent versus a baseline. We measured overall preference and engagement via agent rankings, satisfaction scores and conversation outcomes (that is, natural versus forced endings). To understand the drivers of these preferences, we also assessed user perceptions of specific coaching capabilities, conversational style and the actionability of the guidance of the agent.

We recruited 31 participants (25 male individuals and 6 female individuals, 18–64 years of age) for a 1.5-h session each (48 h of human effort total). Participants interacted with multiple conversational agents in a blinded, counterbalanced order using a Latin square design. This paper focuses on the comparison between our HC agent and a baseline agent built on the same Gemini model, prompted with a general instruction to act as a helpful personal coach. No significant difference in response time was observed between agent conditions.

To guide the interactions, we curated a set of open-ended scenarios reflecting common health concerns (see Supplementary Table 20). Participants selected the scenario that best aligned with their own personal interests to ensure a realistic coaching use case. They were instructed to treat each agent interaction as a new consultation, providing consistent background information each time (see Supplementary Section E.2.2 for detailed evaluation instructions). Each interaction began with the same initial sentence from the chosen scenario, after which participants conversed freely until reaching either a natural resolution or an ending forced by users (if the conversation was deemed unproductive).

Participants assessed each agent post-interaction and provided a final ranking at the end of the study. We adopted established metrics from psychology and agent usability, including subsets of the CUQ^77^, the WAI^78^, the CEMI^79^ and SMART goal-setting evaluations^59^. These metrics provide a comprehensive view of usability, collaborative bond, motivational techniques and goal formulation, echoing the expert-identified coaching themes (see Supplementary Table 21 for raw questions and Supplementary Table 23 for detailed aspects of these metrics). By allowing user choice in scenarios and enabling open-ended interactions, this setup is designed to capture realistic user experiences that surpass the limitations of automated evaluations.

#### Task 2: expert validation of coach design

Although end users are well-equipped to judge their personal satisfaction and engagement (task 1), they may not be able to recognize whether an agent is correctly applying evidence-based coaching techniques. A comprehensive evaluation should also validate the methodological rigour of the agent. Therefore, an expert-led validation is essential to assess the fidelity of the design of the HC agent. The primary goal of this task is to verify that the conversational behaviours of the agent are not just subjectively engaging but also objectively aligned with the intended expert-defined coaching capabilities (HC1–HC6).

We recruited three of the health experts from our formative study to evaluate the conversation transcripts collected from task 1. The experts, blind to the agent conditions, rated each conversation according to the six coaching themes (HC1–HC6), as well as the same set of CUQ, WAI, CEMI and SMART questions. We also incorporated a specialized set of AICM developed by our health coach experts, covering conversation tones (supportiveness and friendliness), style (clarification) and strategy (rapport development, information gathering for key assessment, personalized intervention and appropriate summary; see Supplementary Section E.2.3 for detailed evaluation instructions and questions). Finally, experts provided comparative rankings of the agents on motivational interviewing skills, recommendation quality and credibility.

### PHA evaluation

For a comprehensive evaluation, we compared our PHA against two baseline agent setups (with the same Gemini backbone) to demonstrate the value of our collaborative architecture.

- Baseline 1: a single-agent PHIA system. Previous work has revealed the promising capability of a single agent to conduct data analysis, generate personal health insights and provide health recommendations. We re-implemented the architecture from a previous article^2^ for a PHIA system that is capable of code generation, information retrieval, online search to analyse and interpret behavioural personal health data. This system covers the key capabilities that we have identified for the three specialist agents. Note that although this is a single-agent system, its ReAct framework can still trigger multiple LLM calls^58^. Although this design embodies architectural simplicity (+PHA.4), it may struggle to cohesively integrate the distinct skills of data science, domain expertise and health coaching within a single agent (−PHA.1, −PHA.2 and −PHA.3).
- Baseline 2: a parallel multi-agent system. For multi-agent baselines, we implemented a static architecture that adopts a voting-like strategy^24,61^: each of the three agents will respond to a query from a user in parallel, and an orchestrator will combine the responses and respond to the query. This setup leverages the capabilities of each agent (+PHA.1) and ensures a short response time with a simpler structure than PHA (+PHA.4). However, different agents are working independently with a static structure, which may not be flexible enough to handle diverse needs of users (−PHA.2). It can miss the opportunity to fully unlock the synergies among three agents and lead to a less useful response (−PHA.3).

#### Task 1: end-user multi-agent experience

After combining the three single agents into a multi-agent system, our first evaluation is to assess the ability of the PHA to conduct broad, multi-turn health-related conversations from the perspective of an end user. We aimed to measure the perceived quality of the interaction, focusing on metrics such as helpfulness, coherence, empathy and how well the agent integrates data analysis, domain knowledge and health coaching.

To ensure ecological validity, we conducted a live interactive evaluation in which end users engaged in real-time, multi-turn conversations with each of the three systems while role-playing as specific WEAR-ME personas. The role-playing ensures that the evaluation is based on real-world data collected in WEAR-ME, while keeping the natural conversation setup to measure the open-ended conversational capability of the agent.

*Persona setup.* We sampled a set of 50 representative user personas from the WEAR-ME dataset, covering ten common health profiles (five personas each), ranging from healthy individuals training for a goal to individuals managing chronic conditions such as hypertension or diabetes (examples in Supplementary Section B.2.1). Working with primary-care physicians, we developed detailed background stories and specific health needs for each persona that are aligned with their health data, so that each persona carried a coherent data profile, life context and presenting health concerns that an end user could plausibly inhabit during a live session. All three systems (the single-agent PHIA baseline, the parallel multi-agent baseline and the PHA) had access to the same persona data, ensuring that any differences in performance reflect the agentic architecture rather than data access.

*Live conversation protocol.* We recruited *n* = 45 end users (17 female participants, 25 male participants and 3 other participants (who did not identify themselves as ‘male’ or ‘female’), 21–67 years of age) across a wide range of health conditions. Each participant was mapped to 1 of the 50 WEAR-ME personas that closely matches their health profile. Participants were sampled stratified by diverse health conditions to ensure that all ten health profiles were covered. Supplementary Table 35 lists the detailed break down of the health conditions of the 45 participants across 10 profiles. Before the study, they familiarized themselves with the data profile, background story and health goals of the assigned persona, and then conducted real-time, multi-turn conversations with each of the three systems about the health situation of that persona. During the study, we intentionally inserted random response delays in the systems to control the impact of the system latency. Meanwhile, participants were explicitly instructed to exclude system latency from the evaluation consideration. Systems were presented in a counterbalanced order with a Latin square design, with participants blind to agent conditions. Each conversation continued until the participant determined the needs of the persona were addressed, or the conversation reached a natural end point, or the interaction time exceeded 15 min.

*End-user evaluation.* The evaluation process is close to the HC agent. After each conversation, participants completed a per-agent evaluation using the same rubric instruments for this task (see Supplementary Section F.2.1). The metrics include the overall quality and specific capabilities of the agent in data science, domain knowledge and health coaching (questions adopted from previous agent evaluation tasks), as well as the organic integration of these skills (see the specific questions in Supplementary Table 31 and rubrics in Supplementary Table 32). After interacting with all three systems, participants provided a comparative ranking of the three systems. Numeric end-user results are reported in the results section and in Supplementary Tables 36 and 37. This process resulted in 135 individual agent ratings (3 agents × 45 participants) and 45 comparative rankings. In total, 45 end users spent 90 h on the evaluation.

#### Task 2: expert multi-agent evaluation

Beyond the end-user perspective, this evaluation seeks to assess the technical and clinical validity of the agent’s conversations from the viewpoint of health experts. The goal is to evaluate the soundness of the data analysis, the accuracy of the health information provided, the appropriateness of the coaching strategies, the coordination across three agents, as well as the overall quality of the conversation.

*Expert evaluation.* For the expert evaluation, we used the live conversation logs produced in task 1. Three health coach experts (one female individual and two male individuals, 35–54 years of age) with professional backgrounds in health coaching and digital health (two specializing in fitness and one in sleep) reviewed these live conversations while being blind to agent conditions. Supplementary Section F.2.2 shows the detailed instruction for the expert evaluation. Although the evaluation metrics covered similar domains as the end-user task 1 (DS, DE and HC), the questions were framed from an expert standpoint, such as the clinical validity of the advice from the DE, the correctness of the analysis by the DS agent, and whether the HC agent applied evidence-based motivational interviewing appropriately and safely (see questions in Supplementary Table 33). Experts rated each agent per-conversation and, after reviewing all three systems for a given participant, provided a comparative ranking. Consistent with the live-study framing of task 1, this expert-driven evaluation provides another layer of validation for the quality and reliability of the responses from the agent under ecologically valid conditions. In total, the evaluation involves around 140 h of human effort, resulting in 405 individual agent ratings (135 scripts ×  3 experts) and 135 comparative rankings (45 participants × 3 experts).

#### Task 3: scaled synthesized dialogues

The live evaluations in task 1 and task 2 are ecologically valid but bounded by participant time, which limits the breadth of personas, query types and rater coverage they can cover. To complement them with a larger-scale evaluation, we additionally synthesized conversations between persona-instructed user models and the three systems, and recruited a separate cohort of generalist end users and health experts to rate them. We used a base Gemini 2.5 Flash model, instructed with each WEAR-ME data, background and goals of the persona, to act as the user and engage in three independent multi-turn conversations with each of the three systems, yielding 150 conversation scripts (50 personas ×  3 systems). Twenty end-user generalist evaluators (13 male individuals and 7 female individuals, 20–44 years of age) read the scripts and rated each agent using the same rubric instruments as in task 1, then provided comparative rankings (3,000 individual ratings, 1,000 comparative rankings and 500 h of human effort). Five health experts (four female individuals, one male individual, 25–64 years of age; three specializing in fitness, one in nutrition and one in sports) independently reviewed the same 150 scripts using the task 2 expert rubric, blind to agent conditions (750 individual ratings, 250 comparative rankings and 270 h of human effort). Detailed methodology and full numerical results are reported in Supplementary Section F.3.3.

#### Computational cost

Using the scaled-up, synthesized conversations in PHA task 3, we compared computational cost across the three systems by LLM call count and per-round response time. The single-agent PHIA had the lowest cost (average of 3.0 calls per round, response time of 36.4±1.6 s), followed by PHA (average of 6.5 calls per round, response time of 205.3±15.0 s) and the parallel multi-agent baseline (average of 7.0 calls per round, response time of 244.4±11.4 s). Despite the additional reflection stage, the dynamic agent task assignment by the PHA requires fewer LLM calls and responds faster than the parallel baseline.

### Statistical analysis

For per-rubric three-or-more system comparisons (for example, PHA tasks), we fit a GLMM with a log link, treating system as a fixed effect and rater (and conversation script, where applicable) as random effects. Pairwise contrasts among the systems within each rubric were obtained with Tukey’s honest significance test adjustment. Each rubric was treated as a confirmatory test of a pre-specified, conceptually distinct quality dimension before any system comparison. To verify that this per-rubric framing did not understate the overall type I error, we additionally conducted an across-rubric Holm–Bonferroni robustness check^80^; the outcome of this check has been reported in the caption of each corresponding result table. For comparative rankings, we applied a Friedman (omnibus) test followed by post-hoc pairwise Wilcoxon signed-rank tests with Holm–Bonferroni correction. Effect sizes and confidence intervals are reported alongside *P* values, where applicable. All statistical tests were two-sided. Unless otherwise noted, the error bars represent the standard error of the mean, as detailed in the corresponding tables.

### Ethics statement

The human participant research reported in this study was reviewed and approved under three protocols. The WEAR-ME study was approved by Advarra IRB #Pro 00074093. The retrospective study that collected self-reported symptom cases for the DE agent evaluation was approved by Advarra IRB #GH-SCD-001. The HC agent and PHA user studies were approved by WCG IRB #20244970. All research involving human participants was conducted in accordance with all relevant ethical guidelines and regulations, and informed consent was obtained from all participants before participation.

### Reporting summary

Further information on research design is available in the Nature Portfolio Reporting Summary linked to this article.

## Online content

Any methods, additional references, Nature Portfolio reporting summaries, source data, extended data, supplementary information, acknowledgements, peer review information; details of author contributions and competing interests; and statements of data and code availability are available at https://doi.org/10.1038/s41586-026-11161-8.

## Supplementary information

Supplementary Information Supplementary Sections A–G (A, User-Centered Design and User Journey Details; B, Evaluation Dataset Preparation; C, DS Agent Additional Results and Technical Detailsl; D, DE Agent Additional Results and Technical Details; E, HC Agent Additional Results and Technical Details; F, PHA Additional Results and Technical Details; G, Supplementary Discussion), including Supplementary Tables 1–45 and Supplementary Figs. 1–7.

Reporting Summary

Peer Review File

Supplementary Data The data underlying the graphical representations used in figures, as well as source data and rubrics for each agent.

## Data Availability

All study benchmark tasks, data and results are provided in the Supplementary Information. The multiple-choice question banks used for DE agent task 1 (factuality MCQ; StatPearls Endocrinology, BoardVitals Cardiology, Fitness/Sleep^8^) are available from their respective sources as cited. The *n* = 2,000 self-reported symptom dataset used for DE agent task 2 (diagnostic) was collected under IRB #GH-SCD-001; a de-identified release of this dataset, consistent with the terms of the original consent, is available at the same repository. The 50-persona evaluation subset of the WEAR-ME dataset that underlies multiple end-user and expert studies in the DE agent, HC agent and PHA evaluations is released under a non-restrictive research-use license at the same repository above. Researchers seeking access to the full WEAR-ME dataset (*n* = 1,165) need to complete the insulin resistance dataset access request form as requested^43^ (https://docs.google.com/forms/d/e/1FAIpQLSebcfCZMQKBua7QS9MSvqr9n-fqmfJvmU4blOHXP1WZC0NFqA/viewform).

## Code availability

We are committed to supporting independent validation of and further research on the PHA framework. We open-sourced the core PHA agentic system, including the orchestrator logic; the three specialist sub-agents (DS, DE and HC); their prompt templates, tool-use scaffolding and sandboxed code-execution harness; and all configuration and example scripts required to instantiate the system on top of a capable base LLM. The released codebase is model agnostic: although our main experiments use the Gemini family, the architecture and prompts are designed to work with any sufficiently capable base LLM, as demonstrated by the model-agnostic ablation described in the DE agent evaluation. The code will be released under an Apache 2.0 license with the in-prompt disclaimers and conservative response policies used in this research system. The additional safeguards and deployment measures described in the Discussion are future directions. The implementation of PHA is available (https://github.com/Google-Health/consumer-health-research/tree/main/personal_health_agent). Our work used the Gemini family of base models^44^, as well as additional closed-source SOTA LLMs (for example, GPT-4 or GPT-5 and Claude Opus or Sonnet) for the model-agnostic ablation. The weights of these research-level base models are proprietary to their respective developers and are not ours to release; we therefore cannot redistribute model weights.
