# Supplementary material for: The anatomy of a personal health agent
Source: Nature. 2026 Oct 21;659(8139):197–207. doi: 10.1038/s41586-026-11161-8 (PMC13736220; doi:10.1038/s41586-026-11161-8)
Supplement: Supplementary file 1 [file 41586_2026_11161_MOESM1_ESM.pdf]

nature portfolio

https://doi.org/10.1038/s41586-026-11161-8

Supplementary information

The anatomy of a personal health agent

In the format provided by the authors and unedited

Supplementary Information for:
The Anatomy of a Personal Health Agent

A. Ali Heydari[1,*], Ken Gu[‡,*], Vidya Srinivas[‡,*], Hong Yu[1,*], Akshay Paruchuri[‡,*], Zhihan Zhang[‡], Yuwei Zhang[‡], Qian He[‡], Hamid Palangi[1], Nova Hammerquist[‡], Ahmed A. Metwally[1], Brent Winslow[1], Yubin Kim[‡], Kumar Ayush[1], Yuzhe Yang[1], Girish Narayanswamy[1], Maxwell A. Xu[1], Jake Garrison[1], Samuel Schmidgall[1], Amy Armento Lee[1], Jenny Vafeiadou[1], Ben Graef[°], Isaac R. Galatzer-Levy[2], Erik Schenck[1], Andrew Barakat[1], Javier Perez[1], Jacqueline Shreibati[1], John Hernandez[1], Anthony Z. Faranesh[1], Naghmeh Rezaei[1], Javier L. Prieto[‡], Conor Heneghan[1], Jiening Zhan[‡], Mark Malhotra[1], Shwetak Patel[1], Yun Liu[1], Tim Althoff[1], Xin Liu[1,†], Daniel McDuff[1,†], Xuhai “Orson” Xu[1,3,†,‡]

[1]Google Research, [2]Google DeepMind, [3]Columbia University
[*]Equal Contribution, [†]Equal Leadership, [‡]Work done while at Google Research, [°]Work done at Google via Vituity

Supplementary Material

A User-Centered Design & User Journey Details

A.1 User Journeys Breakdown with Agent Assignment

Supplemental Table S1 | Personal Health Queries and Agent Assignment. Our user-centered design process revealed four major critical user journeys (CUJs) and 26 most common types of user queries, together with query examples. For each CUJ and user type, a group of researchers and health experts design workflows to involve appropriate members of the three agent team (DS: Data Science, DE: Domain Expert, HC: Health Coach) together with an agent collaboration workflow.

Category	User Purpose	Question Types (I want...)	Question Examples	Sub-Agents	Agent Workflow
**Health knowledge (CUJ1)**	I'm here to understand and discover health knowledge	...to understand x	What is HRV?	**DE**	DE agent answers based on internal and external knowledge
		...to find a fact about x	How long is strep contagious?	**DE**	
		...to compare x and y	What is the difference between an HRV & RHR?	**DE**	
		...the pros and cons of x	What are the pros and cons of HIIT?	**DE**	
		...the news about x	What's the latest research on sleep apnea?	**DE**	
**Personal data (CUJ2)**	I'm here to understand my wearable data	...to see my best/worst x data	What's my longest sleep duration last month?	**DS**	DS agent answers by computation w/ results.
		...to see or compare my x data for a time period y	What was my average heart rate during the Euro Cup?	**DS**	
		...to identify meaningful changes in x	Can you review my HRV data from this month and highlight any unusual occurrences?	**DS**, **DE**	DS agent does the computation and DE agent interprets the results and answer
		...to understand what x data says about my health	What is my current fitness level? How has my sleep been?	**DS**, **DE**	
		...to compare my x with other people/clinical guidelines	How does my exercise compare to pregnant women my age?	**DS**, **DE**	
		...to summarize x time-series data	Give me an overview of my physical activity records since I started my training.	**DS**, **DE**	

Supplemental Table S1 – continued from previous page

Category	User Purpose	Question Types (I want...)	Question Examples	Sub-Agents	Agent Workflow
	I'm here to understand my health record	...to answer a question from x	Which lab values in my lipid panel should I pay attention to and why?	**DE**	DE agent interprets, reasons, and summarizes personal data. No complex computation is needed, so DS agent is not involved.
		...to summarize x (at certain timestamp)	Can you help me understand this blood report? Do I sleep better on days where I run for an hour?	**DE**	
	I'm here to understand relationships between my health data	...to see relationships between x and y in the same source (e.g., Fitbit)	Could you maybe explain why my steps fluctuate from weekdays to weekends?	**DS**	DS agent answers by computation w/ results.
		...to understand why x changed	Does my daily exercise in the past six months make my blood healthier?	**DS**, **DE**	DS agent does the computation and DE agent interprets the results and answer
		...to compare x and y across sources	My throat is itchy and I have trouble swallowing. What might be going on?	**DS**, **DE**	
**Wellness advice (CUJ3)**	I'm here to get wellness advice	...advice on x (e.g., improve / change) in general	Give me tips to reduce smartphone usage?	**HC**	HC agent provides wellness advice based on users' needs
		...advice on x (e.g., improve / change) based on personal data.	How do I improve my deep sleep?	**DS**, **HC**	DS agent does related analysis, and HC agent provides personalized wellness advice
		...to identify areas for improvement	What are my biggest problem areas in my exercise routine?	**DS**, **DE**, **HC**	DS agent analyzes personal data. DE agent interprets the results, and HC agent walks through and makes plans with users

Supplemental Table S1 – continued from previous page

Category	User Purpose	Question Types (I want...)	Question Examples	Sub-Agents	Agent Workflow
	I'm here to make progress towards my goals	...to set an appropriate goal or task for x	How much exercise should I be doing to get rid of belly fat?	HC	HC agent sets goals and plans based on users preference and needs.
		...to understand how my behaviors move me toward my goal for x	What are some of the things I have done that have the biggest impact on my blood pressure/sleep?	DE, HC	DE agent provides domain-specific knowledge; HC agent makes plans based on users' preference and needs
		...plans to achieve my x goal	Give me a 8 week 10k training plan for pregnant women.	DS, DE, HC	DS agent analyzes personal data when appropriate. DE agent provides expert-level interpretation and advice, if needed. HC agent walks through with users to set goals and make plans.
		...to adjust my plans or goals based on changing needs	It's raining. Adjust my workout to be indoors today.	DS, DE, HC	
		...to track progress against my goals for x	Based on my training pace, am I on track for completing a marathon in under 3 hours?	DS, DE, HC	
**Personal medical symp-toms (CUJ4)**	I'm here to assess my symptoms	...to explore x symptoms that I'm feeling	How can I tell if I have high blood pressure?	DE	DE agent diagnoses based on information provided
		...advice on a health symptom	How do I improve hypertension?	DS, DE	DS agent does related computation when appropriate, DE agent analyzes the results and provides advice

A.2 User-Centered Survey Details

Supplemental Table S2 | End-User Survey for Ask Health Fitbit Labs. This survey was delivered to end-users who interacted with Ask Health Fitbit Labs (N=555) to ask about their personal health goals and sentiments when interacting with the agent.

Question Category	Question Body	Answer Options
**Engagement / Retention**	How often did you use Insights Explorer in the past week?	• Never<br>• Once a week<br>• 2-3 times a week<br>• 4-6 times a week<br>• Every day of the week
	During the past few weeks, did your usage of Insights Explorer increase, decrease, or remain the same?	• My usage increased<br>• My usage decreased<br>• My usage remained the same<br>• I don't remember
	[Branch: If *decreased*] Please select any reasons that contributed to your decreased usage over time.	[multi-select, randomized order]<br>• Forgot about it<br>• Unsure what to ask about<br>• I can easily get the information I'm looking for elsewhere<br>• I can get better quality answers elsewhere<br>• I didn't trust the information it provided<br>• It was too limited in what it could answer (e.g., types of information)<br>• Response not useful did not address my query, incomplete, etc<br>• Content was made up or factually incorrect<br>• Response was too slow<br>• Other [open ended]
	[Branch: If *increased* or *stayed the same*] What was the main functionality or benefits that you continued using Insights Explorer for?	[open-text]

Supplemental Table S2 – continued from previous page

Question Category	Question Body	Answer Options
	Overall, how satisfied or dissatisfied are you with Insights Explorer?	• Very dissatisfied<br>• Somewhat dissatisfied<br>• Neither satisfied nor dissatisfied<br>• Somewhat satisfied<br>• Very satisfied
	Indicate any aspects of Insights Explorer that have NOT met your expectations:	[multi-select, randomized order]<br>• It wasn't able to answer my questions<br>• Too limited in types of data I ask about (e.g., weight, exercise)<br>• The information was obvious or already available in the app<br>• Not able to ask about recent or real-time data<br>• I didn't know how to use it, or what to use it for<br>• It was not accurate or factually correct<br>• The answers were poor quality or not useful<br>• It didn't remember what I had previously asked<br>• Other [open text]
**Satisfaction**	Which aspects of Insights Explorer, if any, delighted you?	[multi-select, randomized order]<br>• charts<br>• Custom<br>• Correlations across my behaviors and health metrics<br>• Trends in my health metrics<br>• Ability to provide insights quickly without Browse the app<br>• Comparing my health metrics to my demographic and across time<br>• Helped me understand my health and wellness better<br>• [Open-ended]<br>• None of the above
	How satisfied are you with the suggested questions shown to you in Insights Explorer?	• Very dissatisfied<br>• Somewhat dissatisfied<br>• Neither satisfied nor dissatisfied<br>• Somewhat satisfied<br>• Very satisfied
	Please explain why you were satisfied or dissatisfied with the suggested questions.	[open ended]

Supplemental Table S2 – continued from previous page

Question Category	Question Body	Answer Options
	How often were you satisfied with the quality of answers to your questions?	• Never<br>• Rarely<br>• Sometimes<br>• Often<br>• Always
	Please explain why you were satisfied or dissatisfied with the quality of answers to your questions.	[open ended]
	On a scale from 0-10, how likely are you to recommend Insights Explorer to a friend or colleague?	0 (Not at all likely) - 10 (Extremely likely)
**Health Impact & Literacy**	How much do you agree with this statement: "Using Insights Explorer helped me better understand my health and fitness."	• Strongly disagree<br>• Disagree<br>• Neither agree nor disagree<br>• Agree<br>• Strongly Agree
**CUJ Identification**	What are you hoping to achieve by using Insights Explorer? Please select all that apply.	[multi-select, randomized order]<br>• Faster/easier way to get my Fitbit data<br>• Connect the dots between my data points (e.g., how does my sleep impact my activity levels)<br>• Understand how I fit in with the broader Fitbit population (e.g., do I walk more or less than other women my age)<br>• Make comparisons of specific time periods (e.g., when I was ill vs. healthy)<br>• Get recommendations based on my data (e.g., should I take it easy today?)<br>• Get recommendations for personalized goals (e.g., what should my daily step count goal be?)<br>• Learn more about Fitbit metrics and data types (e.g., how is the readiness score computed?)<br>• Learn more about general health questions (e.g., what does it mean to have high cholesterol?)<br>• Other [open ended]
	[For each goal selected] Please rate your satisfaction with your ability to do each of the following using Insights Explorer [show each goal before the rating scale]:	• Very dissatisfied<br>• Somewhat dissatisfied<br>• Neither satisfied nor dissatisfied<br>• Somewhat satisfied<br>• Very satisfied

Supplemental Table S2 – continued from previous page

Question Category	Question Body	Answer Options
**Innovation**	How technologically advanced does this feature seem?	• Extremely advanced<br>• Very advanced<br>• Somewhat advanced<br>• Not very advanced<br>• Not at all advanced
	How unique does this feature seem?	• Extremely unique<br>• Very unique<br>• Somewhat unique<br>• Not very unique<br>• Not at all unique
	How useful does this feature seem?	• Extremely useful<br>• Very useful<br>• Somewhat useful<br>• Not very useful<br>• Not at all useful
	From the list below, which word best describes Insight Explorer?	• Annoying • Exciting<br>• Intuitive • Boring<br>• Fresh • Irrelevant<br>• Busy • Friendly<br>• Patronizing • Creative<br>• Helpful • Professional<br>• Confusing • Impressive<br>• Trustworthy • Cutting-edge<br>• Innovative • Unhelpful<br>• Delightful • Intimidating<br>• Unprofessional • Empowering
**Trust**	To what extent do you trust Insights Explorer?	• Not at all<br>• Slightly<br>• Moderately<br>• Very<br>• Completely
	How credible do you feel the information provided by Insights Explorer is?	• Not at all credible<br>• Slightly credible<br>• Moderately credible<br>• Very credible<br>• Extremely credible
**Experience with other products**	Apart from Insights Explorer, please select any of the following Generative AI products, features, or tools that you have personal experience using.	[multi-select, randomized order]<br>• Gemini • ChatGPT<br>• Coda OpenAI • DALL-E2<br>• Microsoft Co-Pilot • Mid Journey<br>• Notion AI • Stable Diffusion<br>• Another AI feature/capability/tool<br>• None of these

B Evaluation Dataset Preparation

B.1 Description of WEAR-ME Data Categories

B.1.1 Demographics

The Demographics category includes background information used to characterize the study population. This encompasses core attributes such as the participant's year of birth (BIRTH_YEAR_DATA), gender (GENDER_DATA), self-identified ethnicity (Ethnicity), and marital status (marital_status).

B.1.2 Anthropometrics

The anthropometrics category consists of physical body measurements. These data include objectively measured height (HEIGHT_DATA) and weight (WEIGHT_DATA), which are supplemented by self-reported values for weight (weight) and waist circumference (waist_circumference).

B.1.3 Socioeconomic Status

The Socioeconomic Status group provides insight into the participants' social and economic context, which can be a key determinant of health. This includes variables such as self-reported annual household income (household_income), current employment status (employment_status), and the highest level of education achieved (highest_education).

B.1.4 Female & Reproductive Health

This category contains data specific to the physiological and health status of female participants. It covers information regarding current menstruation status (menstruation), the self-reported regularity of menstrual periods (regular_periods), and the use of contraceptives (contraceptives).

B.1.5 Lifestyle & Behavior (Baseline)

This group captures a comprehensive snapshot of self-reported habits, behaviors, and perceptions *at the start* of the study. This includes data on physical activity (aerobic_before', strength_before'), dietary patterns (diet', eat_out'), alcohol and tobacco use (alcohol, smoker), typical sleep patterns (sleep_hours, sleep_quality), and subjective assessments of health (overall_health_before).

B.1.6 Lifestyle & Behavior (Follow-up)

This category includes variables that reassess key habits and perceptions after the study's primary observation period. This allows for the analysis of changes in behaviors such as aerobic activity (aerobic_after), eating habits (eating_after), and self-rated overall health (overall_health_after).

B.1.7 Digital Biomarkers (Wearables)

This group of features include a rich set of high-frequency physiological and behavioral metrics passively collected from wearable sensors. This includes detailed activity tracking, such as daily steps (DAILY_STEPS_DATA) and active zone minutes (DAILY_ACTIVE_ZONE_MINUTES_DATA), alongside comprehensive sleep analysis, covering sleep duration (DAILY_SLEEP_DURATION_DATA), time in sleep stages (DAILY_TIME_IN_SLEEP_STAGES_DATA), and a composite sleep score (SLEEP_SCORE_DATA). The category also contains key cardiovascular signals like daily resting heart rate (DAILY_RESTING_ HEART_RATE_DATA) and heart rate variability (DAILY_HEART_RATE_VARIABILITY_DATA), as well as other physiological measures like respiratory rate (DAILY_RESPIRATORY_RATE_DATA), SpO2 (DAILY_SPO2_DATA), and skin temperature (DAILY_SKIN_TEMPERATURE_DATA). Furthermore, it includes metrics related to fitness (e.g., RUN_VO2_MAX_DATA), physiological stress (DAILY_STRESS_SCORE_DATA, CONTINUOUS_EDA_DATA), and raw sensor outputs like photoplethysmography (PPG_DATA_PER_USER).

B.1.8 Blood Biomarkers (Lab Tests)

This category encompasses a wide range of biomarkers obtained from blood analysis, providing a deep profile of participant health. This includes a full lipid panel with measures of total cholesterol (total cholesterol), HDL (hdl), and triglycerides (triglycerides'); a comprehensive metabolic panel assessing kidney function and electrolytes through markers like creatinine (creatinine) and sodium (sodium); and liver function tests like ALT (alt) and AST (ast). Glycemic control is assessed via Hemoglobin A1c (hba1c) and insulin (insulin), while systemic inflammation is measured by C-Reactive Protein (crp). The dataset also contains a complete blood count (CBC) with white blood cell (white_blood_cell), red blood cell (red_blood_cell), and platelet counts (platelet), along with hormonal measures such as total testosterone (total testosterone).

B.1.9 Self-Reported Medical History

This group of inputs document physician-diagnosed health conditions as reported by participants. This includes the presence of major chronic diseases such as cardiovascular disease (CVD), diabetes (diabetes), hypertension (hypertension), and kidney disease (kidney Disease), along with the reported date of diagnosis (e.g., cardiovascular_disease_date).

B.1.10 Self-Reported Clinical Data

Lastly, this category contains specific clinical values and medication information provided by the participants. This includes recent measurements like systolic and diastolic blood pressure (systolic_bp, diastolic_bp), self-reported cholesterol levels (cholesterol_reported), and lists of current medications (medications) with an additional question emphasizing medication for diabetes management (diabetes_medications).

B.2 User Profiles for Evaluation Studies

To ensure the robustness and generalizability of our evaluation, we constructed ten representative user profiles that encapsulate a diverse range of common health scenarios and needs. These profiles were stratified into two primary categories.

The first category includes four profiles representing individuals without diagnosed chronic diseases but with distinct health objectives: (1) a performance-oriented individual training for athletic goals; (2) a generally healthy individual seeking to improve a specific wellness aspect, such as sleep quality; (3) an individual with a sedentary lifestyle but no formal disease diagnosis (sub-healthy); and (4) an individual recovering from an acute injury or life event disrupting their health baseline.

The second category comprises six profiles designed to reflect major public health concerns, with each profile centered on a prevalent chronic condition: (5) Anxiety/Depression, (6) Hypertension, (7) Respiratory Conditions, (8) Hypercholesterolemia, (9) Diabetes, and (10) Cardiovascular Disease (CVD). It is noteworthy that these profiles were designed to reflect real-world complexity, and individuals within these latter six categories may present with comorbidities. To ground each profile in realistic health data, it contains a set of quantitative health metrics as introduced in Supplemental Section B.1.

Finally, to create a holistic and contextually rich persona for each profile, two narrative components were synthesized by attending primary care clinicians:

- User Story: A concise narrative was written to encapsulate the user's lifestyle, circumstances, and health-related behaviors. In addition, experts also create a synthetic but plausible historical context, including relevant personal or family medical information, was established to provide richer context for agent responses
- Health and Wellness Goal: A specific, actionable goal was defined for each user, reflecting their primary motivation for engaging with a personal health agent and providing a clear objective for coaching-style interactions.

B.2.1 User Persona Examples for Evaluation Studies

Below is an example persona with health data and background stories of Profile Category 7:

```
# Demographics
Age: [masked for anonymity]; Sex: [masked for anonymity]
Height: [masked for anonymity]; Weight: [masked for anonymity]; BMI: [masked for anonymity]
Employment Status: Part-time; Marital Status: Married or Partnered
Healthy Lifestyle? (1: Strongly Disagree - 5: Strongly Agree): Agree
Alcohol Consumption: Never; Smoker: No

# Blood Tests
Total Cholesterol: 169.0 (mg/dl); Triglycerides: 170.0 (mg/dl); HDL: 42.0 (mg/dl); LDL:
    100.0 (mg/dl)
Glucose: 145.0 (mg/dl); HBA1C: 6.9 (perc); MCV: 97.6 (femtoliters)

# Health Disease Conditions
Hypertension: 6-10 years; Respiratory Condition: 16+ years; Diabetes: 6-10 years
```

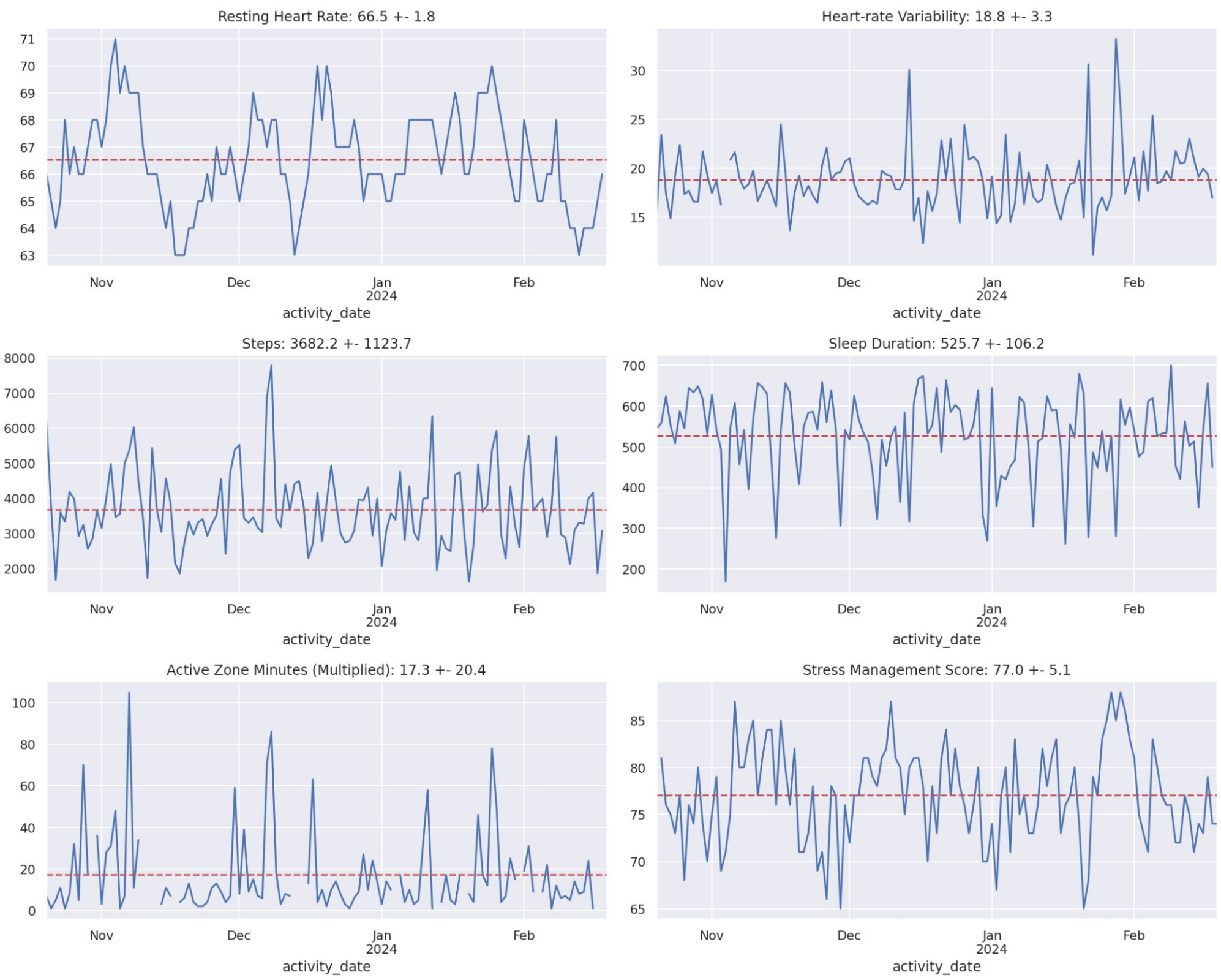

Supplemental Figure S1 | User Persona Wearable Data Recording Example from Profile Category 7. Panel titles report the mean ± standard deviation across daily observations.

```
# Wearable Data Records
Supplemental figure below.

# User Story
A 67 year old male who has some health problems. He served in Iraq and oversaw the burn pits
     many times. His barrack was also near them. After his service he developed respiratory
    problems (COPD) and hypertension. He also developed diabetes. He tries to keep his
    weight under control. He is still working, but now works at the VA in guest services.
    He does a little bit of walking each day, but is limited by his lungs at times.  He
    manages his stress pretty well, and has a low stress job. He is married and gets along
    good with his wife. He sleeps quite a bit, nearing 8.75 hours per night, but does
    occasionally have a bad night a few times per month that is significantly less than this
    . He sometimes has nightmares about his service that wakes him up at night. Additionally
     he thinks he may have sleep apnea.  He eats a balanced diet of fruits and vegetables,
    grains and meat. He occasionally has fast food from the canteen at the VA.

# User Goal that they want to get advice
He would like to try to find a way to be more active. He may need a medication change to
    help.  Additionally he would like to sleep a little less and more consistently.
```

Below is a conversation example between a user persona and our PHA system. The persona information is listed below.

```
# Demographics
Age: [masked for anonymity]; Sex: [masked for anonymity]
Height: [masked for anonymity]; Weight: [masked for anonymity]; BMI: [masked for anonymity]
Employment Status: Unable to work; Marital Status: Married or Partnered
Healthy Lifestyle? (1: Strongly Disagree - 5: Strongly Agree): nan
Alcohol Consumption: Never; Smoker: No

# Blood Tests
Total Cholesterol: 187.0 (mg/dl); Triglycerides: 217.0 (mg/dl); HDL: 55.0 (mg/dl); LDL: 98.0
     (mg/dl)
Glucose: 102.0 (mg/dl); HBA1C: 6.8 (perc); MCV: 89.5 (femtoliters)

# Health Disease Conditions
Anxiety / Depression: 6-10 years; Diabetes: 6-10 years

# Wearable Data Records
Supplemental figure below.

# User Story
54 year old female who lives with her husband. She has been active with exercise in the past
     up until Nov 2023. After that point she had an admission to the hospital for
    Hyperosmolar Hyperglycemic State (HHS). She had been lackadaisical about using insulin
    at the time and she was bedridden for about a week. This really set her back and she has
     not been as active since. She realizes this is partially her own doing and is
    embarrassed at this. She has been better about taking her insulin, and is now working to
     get more exercise per day. She is not working and has the time to exercise, as long as
    it is low impact. She was slightly overweight at the time of the admission, but has
    gained even more weight by being more sedentary. She sleeps about 7.3 hours per night
    and also can nap during the day. She has been told she snores loudly by her husband and
    even has breathing pauses.  Sometimes she just doesnt have the energy to do anything.
    She has had depression for some time now, but certainly her current condition does not
    help. She manages stress fairly well, but does have her good days and bad days. Her diet
     is mostly frozen meals and fast food.

# User Goal that they want to get advice
User wants to get her BMI back below 30 and get back to 10,000 steps per day. She also wants
     to avoid getting readmitted to the hospital.
```

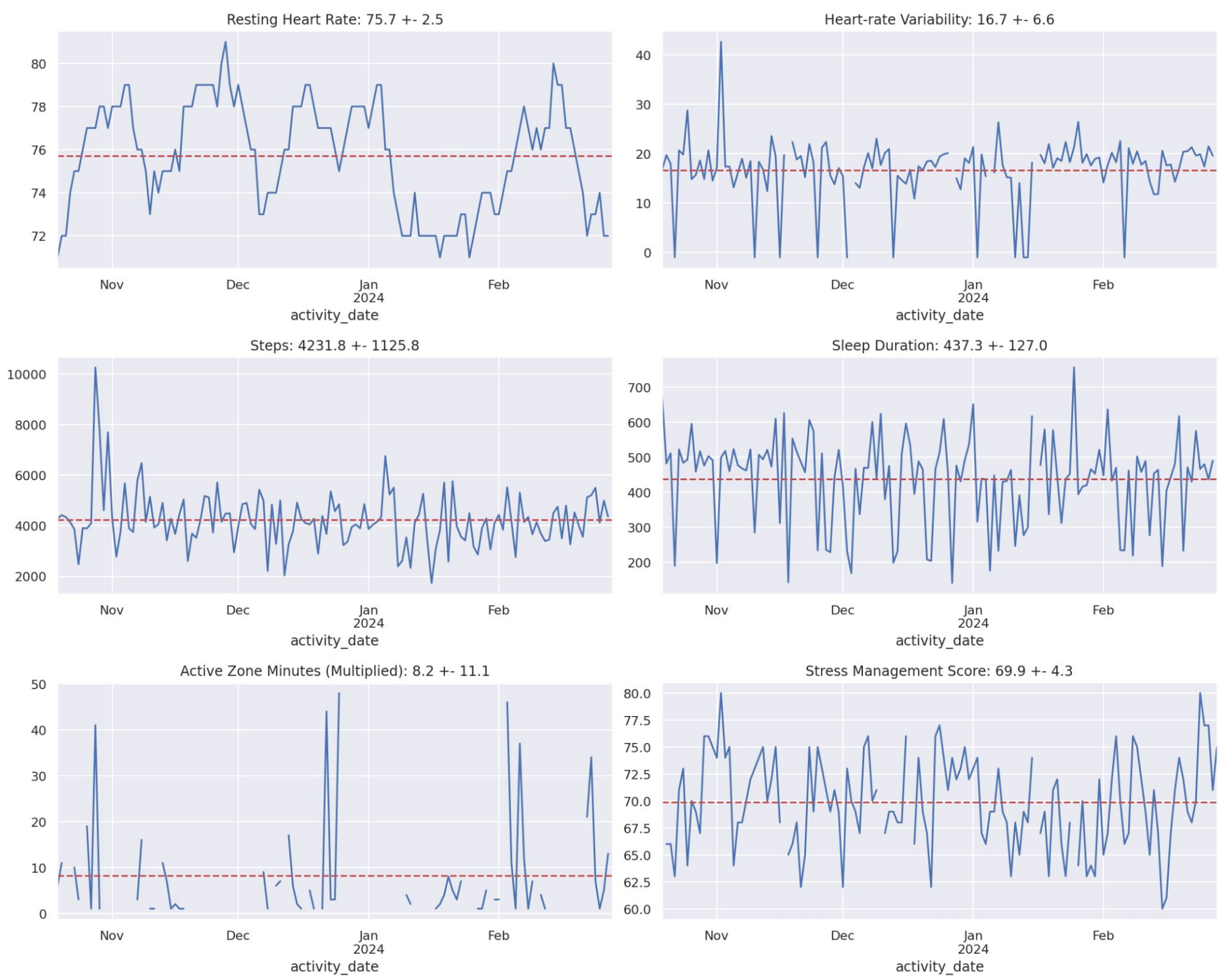

Supplemental Figure S2 | A Second User Persona Wearable Data Recording Example from Profile Category 9. Panel titles report the mean ± standard deviation (s.d.) across daily observations.

C DS Agent Additional Results and Technical Details

C.1 DS Agent Table Schemas

C.1.1 Summary Data Table

```
{
    "name": "Summary DataFrame",
    "variable_name": "summary_df",
    "description": (
        "This is a summary of the user's activity, sleep, and personal health"
        " records data. Each row in the summary dataframe represents a single"
        " day."
    ),
    "columns": {
        "datetime": {
            "description": "The date of the record. This is the index column.",
            "dtype": "datetime64[ns]",
        },
        "steps": {
            "description": "The number of steps taken each day.",
            "dtype": "float64",
        },
        "sleep_minutes": {
            "description": "The total sleep time (in minutes) for each day.",
            "dtype": "float64",
        },
        "bed_time": {
            "description": "The time the user went to bed.",
            "dtype": "datetime64[ns]",
        },
        "wake_up_time": {
            "description": "The time the user woke up.",
            "dtype": "datetime64[ns]",
        },
        "resting_heart_rate": {
            "description": (
                "The average resting heart rate (in beats per minute) for each"
                " day."
            ),
            "dtype": "float64",
        },
        "heart_rate_variability": {
            "description": "The user's heart rate variability.",
            "dtype": "float64",
        },
        "active_zone_minutes": {
            "description": "The number of active zone minutes earned each day.",
            "dtype": "float64",
        },
        "deep_sleep_minutes": {
            "description": (
                "The amount of deep sleep (in minutes) for each day."
            ),
            "dtype": "float64",
        },
        "rem_sleep_minutes": {
            "description": "The amount of REM sleep (in minutes) for each day.",
            "dtype": "float64",
        },
        "light_sleep_minutes": {
            "description": (
```

```
                "The amount of light sleep (in minutes) for each day."
            ),
            "dtype": "float64",
        },
        "awake_minutes": {
            "description": (
                "The amount of awake time (in minutes) for each day."
            ),
            "dtype": "float64",
        },
        "stress_management_score": {
            "description": "The user's stress management score for each day.",
            "dtype": "float64",
        },
        "fatburn_active_zone_minutes": {
            "description": (
                "Part of the active zone minutes spent in the fat burn active"
                " zone which is calculated as (220 - age - resting_heart_rate)"
                " * 0.4 + resting_heart_rate."
            ),
            "dtype": "float64",
        },
        "cardio_active_zone_minutes": {
            "description": (
                "Part of the active zone minutes spent in the cardio active"
                " zone which is calculated as (220 - age - resting_heart_rate)"
                " * 0.6 + resting_heart_rate."
            ),
            "dtype": "float64",
        },
        "peak_active_zone_minutes": {
            "description": (
                "Part of the active zone minutes spent in the peak active zone"
                " which is calculated as (220 - age - resting_heart_rate) *"
                " 0.85 + resting_heart_rate."
            ),
            "dtype": "float64",
        },
    },
}
```

C.1.2 Activities Data Table

```
{
    "name": "Activities DataFrame",
    "variable_name": "activities_df",
    "description": (
        "This is a table of the user's physical activities. Each row in the"
        " activities dataframe represents a single recorded activity."
    ),
    "columns": {
        "start_time": {
            "description": "The start time of the activity.",
            "dtype": "datetime64[ns]",
        },
        "end_time": {
            "description": "The end time of the activity.",
            "dtype": "datetime64[ns]",
        },
        "activity_name": {
            "description": (
                'The name of the activity, e.g., "WALKING", "SPORT", "RUNNING",'
                " etc."
            ),
            "dtype": "str",
        },
        "distance": {
            "description": "The distance covered during the activity in miles.",
            "dtype": "float64",
        },
        "duration": {
            "description": "The duration of the activity in minutes.",
            "dtype": "float64",
        },
        "elevation_gain": {
            "description": (
                "The total elevation gain during the activity in meters."
            ),
            "dtype": "float64",
        },
        "average_heart_rate": {
            "description": (
                "The average heart rate during the activity in beats per"
                " minute."
            ),
            "dtype": "float64",
        },
        "calories": {
            "description": "The number of calories burned during the activity.",
            "dtype": "float64",
        },
        "steps": {
            "description": "The number of steps taken during the activity.",
            "dtype": "float64",
        },
        "active_zone_minutes": {
            "description": (
                "The number of active zone minutes earned during the activity."
            ),
            "dtype": "float64",
        },
```

```
    },
}
```

C.1.3 Population Data Table

```
{
    "name": "Population DataFrame",
    "variable_name": "population_df",
    "description": (
        "The population dataframe represents data for each percentile of the"
        " population broken down by age and gender. Each row in the population"
        " dataframe represents a single percentile for a given age group and"
        " gender."
    ),
    "columns": {
        "percentile": {
            "description": "The percentile of the population.",
            "dtype": "int",
        },
        "age": {
            "description": (
                'The age group of the percentile, one of ["18-24", "25-34",'
                ' "35-44", "45-54", "55-64", "65+"].'
            ),
            "dtype": "str",
        },
        "gender": {
            "description": (
                'The gender of the percentile, one of ["male", "female"].'
            ),
            "dtype": "str",
        },
        "resting_heart_rate": {
            "description": "The resting heart rate for the percentile.",
            "dtype": "float64",
        },
        "heart_rate_variability": {
            "description": "The heart rate variability for the percentile.",
            "dtype": "float64",
        },
        "fatburn_active_zone_minutes": {
            "description": (
                "Part of the active zone minutes spent in the fat burn active"
                " zone for the percentile."
            ),
            "dtype": "float64",
        },
        "cardio_active_zone_minutes": {
            "description": (
                "Part of the active zone minutes spent in the cardio active"
                " zone for the percentile."
            ),
            "dtype": "float64",
        },
        "peak_active_zone_minutes": {
            "description": (
                "Part of the active zone minutes spent in the peak active"
                " zone for the percentile."
            ),
            "dtype": "float64",
        },
        "steps": {
            "description": (
```

```
                "The number of steps taken each day for the percentile."
            ),
            "dtype": "float64",
        },
        "rem_sleep_minutes": {
            "description": (
                "The amount of REM sleep (in minutes) for the percentile."
            ),
            "dtype": "float64",
        },
        "deep_sleep_minutes": {
            "description": (
                "The amount of deep sleep (in minutes) for the percentile."
            ),
            "dtype": "float64",
        },
        "light_sleep_minutes": {
            "description": (
                "The amount of light sleep (in minutes) for the percentile."
            ),
            "dtype": "float64",
        },
        "stress_management_score": {
            "description": "The stress management score for the percentile.",
            "dtype": "float64",
        },
    },
}
```

C.2 Prompt Setups

C.2.1 Base Gemini Prompt Template

```
"""
You are an expert data scientist. Generate a step by step analysis approach based on given
    user query.
## Output Format
Your output should include _one_ discussion, _one_ approach field.

Begin!
Query: {query}
== DISCUSSION ==
"""
```

C.2.2 Approach Generation Prompt Template

```
"""
{#- Preamble: Instructions for generating the approach given the user's query -#}
You are an expert Python data analyst skilled in working with Fitbit time series health data
    . Your task is to devise a precise, step-by-step analysis plan based *only* on the
    provided user query and the available data schemas below. This plan will guide
    subsequent Python code generation but should **NOT** contain any code itself.

**Available Data Schemas:**
{{data_summary}}

**Task:**
Analyze the user's query below. Generate a **Discussion** section analyzing the query
    feasibility and operationalizing terms, followed by a step-by-step **Approach** section
    outlining the analysis plan.

**User Query:**
{{query}}

**Instructions for Generating the Plan:**
**A. Discussion Section:**
1.  **Analyze Feasibility:** Determine if the query can be answered using the provided data
    schemas.
2.  **Identify Ambiguity:** Pinpoint any vague terms in the query (e.g., "better," "active,"
     "consistent," "often").
3.  **Operationalize Terms:** Define *exactly* how each ambiguous term will be measured
    using specific columns and calculations from the available data (e.g., "Define 'better
    cyclist' by comparing average speed (`distance`/`duration`) and average `
    average_heart_rate` during cycling activities").
4.  **Outline Strategy:** Briefly summarize the overall plan.
5.  **Unanswerable Queries:** If the query cannot be answered (e.g., requires non-existent
    data like friend's metrics, uses undefined concepts), clearly state *why* in the
    discussion.

**B. Approach Section:**
* **If Query is Unanswerable:** Write a single step containing a helpful message explaining
    why it cannot be answered, based on the discussion (e.g., "1. Return 'Query cannot be
    answered because data for [missing element] is not available.'").
* **If Query is Answerable:** Provide a numbered list of precise, unambiguous steps:
    1.  **Timeframe:**
        * Specify the exact start and end dates/times for the analysis period.
        * If not specified in the query, default to the last 1 year of available data from
```

```
            the relevant DataFrame(s).
        * Ensure the chosen timeframe is reasonable and clearly stated.
    2.  **Data Selection & Filtering:**
        * Identify the necessary DataFrame(s) (e.g., `Summary DataFrame`, `Activities
            DataFrame`).
        * Specify the exact column(s) required from each DataFrame.
        * Detail any filtering criteria based on the operationalized terms or timeframe (e.g
            ., "Filter `Activities DataFrame` for `activity_name` in ['OUTDOOR_BIKE', '
            BIKING']", "Filter `Summary DataFrame` for `datetime` within the specified
            timeframe").
    3.  **Data Transformations:**
        * Clearly describe any required calculations or data manipulations (e.g., "Calculate
             speed as `distance` / (`duration` / 60) for each activity", "Aggregate daily `
            steps` from `Summary DataFrame` into weekly averages", "Lag `sleep_minutes` by
            one day to align with next day's `steps`").
        * Ensure transformations are logical and unambiguous. Specify units where relevant.
    4.  **Data Sufficiency Checks:**
        * Define and justify minimum data requirements (e.g., "Require at least 5 activities
             matching the criteria within each comparison period for analysis"). State this
            check as a step.
        * Specify checks for missing values in critical columns needed for calculations (e.g
            ., "Check `average_heart_rate` for nulls. If >50\% are missing in a period,
            exclude this metric from analysis for that period and note it"). State this
            check as a step.
        * Clearly state what should happen if data is insufficient (e.g., "If insufficient
            data, return a message indicating this.").
    5.  **Statistical Analysis:**
        * Specify which summary statistics to calculate (e.g., mean, median, count, std dev,
             min, max). Justify why they are appropriate (consider data distribution - e.g.,
             "Calculate median `steps` due to potential skewness").
        * If performing statistical tests (e.g., t-test, correlation, ANOVA):
            * State the exact test to be used and the variables involved.
            * Justify the choice of test.
            * Mention necessary assumptions (e.g., normality for t-test) and how they will
                be assessed (e.g., "Perform Shapiro-Wilk test on metric distributions within
                 each group").
            * Ensure the test setup is unambiguous.
    6.  **Define Output:**
        * Clearly state what specific data, statistics, or test results the execution of
            this plan should produce (e.g., "Return the calculated median steps for each
            month", "Return the p-value from the t-test comparing average heart rates"). **
            Do not include interpretation or comparisons in the plan itself.**

**Key Requirements & Constraints:**

* **Precision:** Steps must be unambiguous. Another analyst using the same data must reach
    the exact same numerical result by following the plan. Use specific thresholds and
    column names.
* **No Assumptions:** Base the plan *only* on the query and provided schemas. **Do not make
    extra assumptions!** If a needed threshold isn't specified or inferable, state this as a
     limitation or use a clearly justified standard (e.g., population norms if applicable
    and available).
* **Relevance & Scope:** Use only the provided data. Address *only* the user's query. The
    plan should *only* return the data/statistics needed to answer the query, not
    interpretations.
* **NO CODE:** The output must be the plan (Discussion + Approach) only.

**Output Format:**
Your output must contain exactly one `== Discussion ==` section and one `== Approach ==`
    section, formatted as shown in the examples from the original prompt.
```

```
Begin!
**Query:** {{query}}
== Discussion ==
"""
```

C.2.3 Base Gemini Code Generation Prompt Template

```
"""You are a Python expert with tons of experience with data analysis on Fitbit health and
    fitness time series data.
You are provided with dataframe schemas that describe Dataframes available to use, and a
    docstring that describe how an `analysis` function should work to generate code that
    answers the user query listed in the docstring.

<Dataframe schemas>
{dataframe_schemas}
</Dataframe schemas>

<Docstring>
{docstring}
</Docstring>

Now generate python code that follows the docstring instruction.
Don't include docstring or comments in the generated code.
"""
```

C.2.4 DS Agent Code Generation Prompt Template

```
"""
# Role and Goal
You are an expert Python data scientist specializing in analyzing Fitbit health and fitness
    time series data using the pandas library. Your goal is to write the **functional body**
     of a Python script that performs a specific analysis task.

# Inputs Provided
You will receive:
1.  **DataFrame Schemas:** Detailed descriptions of the pandas DataFrames available for use.
     Pay close attention to column names, data types, and the index. Crucially, `datetime`
    information is the **index** for these DataFrames, not a standard column.
2.  **DataFrame Summary:** A summary of the available data relevant to the user.
3.  **Approach:** A step-by-step description outlining the specific analysis required. It
    describes the objective, potentially mentions the input DataFrames (by conceptual name
    or description), and the expected output or result of the analysis (answering the user
    query within).
<Dataframe Summary>
{{data_summary}}
</Dataframe Summary>

<Dataframe schemas>
{{dfs_info}}
</Dataframe schemas>

<Approach>
{{approach}}
</Approach>
```

```
# Instructions for Generating Python Code
Based strictly on the provided schemas and docstring, generate **only** the Python code
    block that performs the requested analysis:

1.  **Core Logic:** Implement the data manipulation and analysis steps necessary to fulfill
    the requirements described in the `<Docstring>`.
2.  **Assume Inputs:** Assume the DataFrames described in `<Dataframe schemas>` are already
    loaded and available as pandas DataFrame variables (e.g., `df1`, `activity_df`, `
    sleep_df` - infer appropriate names if not specified, or use generic names if necessary)
    . The docstring might hint at the variables.
3.  **Use Index:** Remember to leverage the `datetime` index for time-based operations,
    filtering, or aggregations. Do not treat `datetime` as a column unless explicitly stated
     otherwise in the schema.
4.  **Imports:** Start the code block with all necessary import statements. Include the
    standard ones provided below, and add any others required by your generated analysis
    code (e.g., `numpy`).
5.  **Output:** The code should result in the final requested output (e.g., a processed
    DataFrame, a calculated value, a list of results). This might involve assigning the
    final result to a variable (e.g., `result_df` or `final_value`) as the last step,
    simulating what a function might return.
6.  **No Extras:**
    * Do **NOT** include the docstring itself in the output.
    * Do **NOT** include any comments (`# ...`) in the generated code.
7.  **Code Quality:** Write clean, efficient, and idiomatic pandas code. Use vectorized
    operations where appropriate.

# Required Standard Imports (Include at the start of the generated code)
```python
from typing import Any, Dict # Keep if type hinting might appear in complex scenarios,
    otherwise optional
import pandas as pd
import numpy as np # Often needed for analysis
import datetime

def analysis(summary_df: pd.DataFrame | None ,
    activities_df: pd.DataFrame | None ,
    profile_df: pd.DataFrame | None,
    population_df: pd.DataFrame | None,) -> Dict[str, Any]:
"""
```
```

C.2.5 Code Debug Prompt Template

```
"""
You are an expert Python data programmer specializing in analyzing and debugging Fitbit time
     series health data according to specific analysis requirements.

**Task:** Review the provided code generation prompt, the generated Python code, and the
    results of executing that code (which may include errors, tracebacks, or unexpected
    output). Identify the error(s) or discrepancies, fix the code accordingly, and return *
    only* the complete, corrected Python code block.

**Guiding Principles:**
* The fixed code must accurately implement the analysis described in the 'Code generation
    prompt'.
* The fix should directly address the error(s) or issues indicated in the 'Code execution
    results'.
* Preserve the original intent and overall logic described in the 'Code generation prompt'.
* Maintain the original coding style and structure where possible, unless changes are
    necessary for the fix.
* Do not introduce significant unrelated logic changes.

**Input:**

**1. Code generation prompt (Original Goal):**
{{code_instructions}}

**2. Generated code (Contains Errors/Issues):**
{{code}}
**3. Code execution results (Error, Traceback, or Output):**
{{code_result}}

**4. Code Update Feedback (Optional):**
Code Update Feedback:
{{code_update_feedback}}
**Output:**
Fixed Python code:
```python

"""
```
```

C.3 DS Agent Evaluation Rubrics Details

C.3.1 Task 1 Rubrics: Analysis Plan Generation

The rubric questions are designed to be precise and evaluate the quality of the analysis plan along the following topics:

1. **Timeframe:** Does the analysis consider a specific timeframe (e.g., past month, past 6 months) when it helps personalize and align with user intent?
2. **Data Transforms:** Does the approach correctly reference existing columns and apply any necessary data transformations, without logical errors, in a way that others can reliably reproduce?
3. **Data Sufficiency:** Does the approach reasonably and clearly assess whether there is enough data, both in terms of available rows and non-missing cells within columns, to support the intended transformations, calculations, and statistical analyses?
4. **Summary Statistics:** Does the approach appropriately calculate summary statistics where helpful, and are the chosen statistics reasonable given the context and question being answered?
5. **Statistical Tests:** Does the approach appropriately apply statistical tests when helpful, with clear, reproducible methods, reasonable test choices, and proper consideration of distributional assumptions?
6. **Overall Alignment:** Finally, given the evaluation of the above dimensions, does the approach align with the user's intent?

Our original rubrics items contain 33 questions, as shown in Table S3. When using the total set of 33 rubrics, our human annotators on the 141 query-plan pairs (6157 items) exhibited an agreement rate 78.4% with a Bennett's S (a measure of inter-rater reliability, IRR) of 0.574.

Most questions exhibit reasonable to high agreement. However, given the subjectivity of some of the rubric questions (e.g., *"Is the hardcoded value grounded based on the user's query or some accepted standard?"*), we also observed some rubric items with low agreement or were rarely rated due to conditional questions. For the purposes of developing a reliable autorater, we removed these rubric items (7 out of 33 rubric items) for the autorater. Specifically, we removed questions around hard-coded values in the generated codes as they only occurred in a few instances and questions asking about considering the distribution of the data when calculating summary statistics. After removing these items (marked $\times$ in Table S5), the human-human agreement increased to 80.2% with a Bennett's S of 0.622, indicating a moderate level of agreement. Therefore, our final rubrics employed 26 items.

To determine a numeric measure of the quality of the approach based on the rubric items, we developed a points deducted metric, which is a series of conditionals where if the answer to a rubric item implies a lower quality approach (e.g., answering no to *"Is the statistical test reasonable"*) a point is deducted. In total, there is a potential of 16 points to be deducted for any given approach. The final performance was computed as the ratio of the non-deducted scores among the total scores.

Supplemental Table S3 | **DS Task 1 (Analysis Planning) Evaluation Rubrics.** The original rubrics contain 33 items. However, as shown in Table S5, 7 out of 33 rubrics have low IRR due to subjectivity and are removed, leading to a final rubric set of 26 items.

Topic	Rubric Question	Full Question
Time Frame	1. Identify Timeframe	Does the approach identify a specific timeframe for the question (e.g., looking at the data in the past X months)?
	1.1 Reasonable Timeframe	If yes, is the timeframe sufficiently reasonable?
	1.2 Clear Timeframe	If yes, is the timeframe sufficiently clear (e.g., the timeframe is unambiguous.)? Do not make any extra assumptions!
	2. Timeframe Helpful	Would a timeframe improve the answer a user gets?

Supplemental Table S3 – continued from previous page

Topic	Rubric Question	Full Question
Data Transforms	1. Appropriate Columns	Does the approach reference appropriate columns (if not explicitly referencing a column, it should be unambiguous what column and data table we are referring to) that exist for the approach?
	2. Reference Present Cols	Does the approach reference columns that do not exist?
	3. Made Transformations?	Did the approach make any data transformations (separate from identifying the timeframe and calculating data statistics)?
	3.1 Transformations Clear	If yes, is the approach sufficiently clear in how to do it (different data analysts can unambiguously get the same result from the calculation.)? Do not make any extra assumptions!
	3.2 Transformation Logical Errors	If yes, did the transformations involve any logical errors (e.g., the query refers to how my steps are based on my sleep from the night before but the approach uses the sleep data on the day the steps are taken)?
	3.3 Transformations Necessary	If no, are transformations necessary for the approach to answer the question?
Data Availability	1. Sufficient Data Points	With respect to the availability of data points (rows) for the transformations and calculations, does the approach identify whether there is/is not sufficient data?
	1.1 Reasonable Check for Sufficient Data Points	If yes, assuming we have data for all the columns needed for calculations, descriptive statistics and statistical tests, is the sufficiency check reasonable (is it too strict, not strict enough)?
	2. Sufficient Column Data	With respect to the specific columns necessary for calculations, descriptive statistics and statistical tests, does the approach identify whether there is/is not sufficient data (cells in those columns)?
	2.1 Clear Column Check	If yes, is the sufficiency check clear (e.g., we know exactly what columns to look at or how to count/calculate whether there is/is not sufficient data)?
	2.2 Reasonable Column Check	If yes, is the sufficiency check reasonable (is it too strict, not strict enough)?
	2.3 Comprehensive Column Check	If yes, is the sufficiency check comprehensive (all columns that are relevant are checked)?
Statistical Test	1. Calculated summary statistics?	Does the approach calculate summary statistics (e.g., mean, median, mode, max, min, standard deviation, percentiles, count, sum, etc.)?
	1.1 Reasonable summary statistics?	**If yes** to 1, is it reasonable to calculate these statistics?

Supplemental Table S3 – continued from previous page

Topic	Rubric Question	Full Question
	1.2 Statistical Test Necessary	**If no** to 1, would calculating summary statistics improve the data analysis approach to answer the question?
	2. Calculated Statistical Test?	Does the approach perform any statistical test (e.g., T-test, ANOVA, correlation etc.)?
	2.1 Reasonable statistical tests?	**If yes** to 2, is it reasonable to perform this test?

Supplemental Table S3 – continued from previous page

Topic	Rubric Question	Full Question
	2.2 Clear Statistical Test	**If yes** to 2, is the approach sufficiently clear in how to do it (different data analysts can unambiguously get the same result from the calculation.)? **Do not make any extra assumptions!**
	2.3 Distribution Reasonable	**If yes** to 2, does the approach consider the distribution of the data necessary to satisfy the assumptions of the statistical test?
	2.4 Statistical Test Necessary	**If no** to 2, would a statistical test improve the data analysis approach to answer the question?
Overall Alignment	Satisfy Query Intent?	Overall, does the approach align with the user's intent (i.e., the user will get a satisfactory answer from the analysis)?

C.3.2 Task 2 Unit Tests: Code Generation and Execution

Task 2 involves 25 test suites (from analysis plan to code generation and execution). For each test suite, experts prepared a set of unit tests. These unit tests assess the system's ability to handle limited data, perform data transformations, apply statistical methods, and ultimately answer the user's question.

In total, 173 unit tests were created (each test suites have various unit tests). We present a few examples of unit tests of one specific test suite.

Example test suite: "How are my activity levels when I sleep less than 4 hours the night before?"

The test suite include a variety of unit tests to ensure the generated analysis code is robust and accurate. These tests cover key aspects of the data analysis pipeline, from initial data validation to the final statistical conclusions. The example unit tests for this suite are as follows.

Data Sufficiency Test: This test confirms that the system correctly identifies when there is insufficient data for a meaningful analysis. Specifically, it provides a dataset with fewer than the required 30 days for one of the comparison groups and verifies that the system returns the appropriate "Insufficient data" message.

```
@pytest.fixture
def generate_data_unit_test_1():
  """Scenario: Insufficient data for analysis."""
  # Create data with fewer than 30 days in either group
  summary_df = pd.DataFrame(
      {
          'sleep_minutes': [400, 300, 200, 450, 350],  # Sample sleep data
          'steps': [8000, 7000, 6000, 9000, 8500],  # Sample steps data
          'active_zone_minutes': [
              30,
              25,
              20,
              35,
              30,
          ],  # Sample active_zone_minutes data
      },
      index=pd.date_range('2023-01-01', periods=5, freq='D'),
  )  # Create a date range for the index
```

```
    # create empty dataframes
    activities_df = pd.DataFrame()
    profile_df = pd.DataFrame()
    population_df = pd.DataFrame()
    return summary_df, activities_df, profile_df, population_df


def test_case1(generate_data_unit_test_1):
    summary_df, activities_df, profile_df, population_df = (
        generate_data_unit_test_1
    )
    results = analysis(
        summary_df, activities_df, profile_df, population_df
    )  # Pass all DataFrames
    assert (
        results
        == 'Insufficient data for analysis. Less than 30 days in one or both'
        ' groups.'
    )
```

Statistical Test Selection Logic: We designed tests to validate the correct application of statistical methods based on data distribution.

- One test uses normally distributed data to confirm that the system selects an independent t-test for comparing the two sleep groups.
- Another test utilizes non-normally distributed (exponential) data to ensure the system correctly switches to the Mann-Whitney U test.

```
@pytest.fixture
def generate_data_unit_test_2():
    """Scenario: Data for normality testing (Mann-Whitney U)."""
    # Non-normally distributed data (using scipy.stats.expon)
    num_days = 1000
    start_date = pd.to_datetime('2023-01-01')
    dates = pd.date_range(start=start_date, periods=num_days, freq='D')

    summary_df = pd.DataFrame(
        {
            'sleep_minutes': norm.rvs(
                scale=420, size=num_days, random_state=42
            ),  # Using random_state for reproducibility
            'steps': expon.rvs(scale=8000, size=num_days, random_state=42),
            'active_zone_minutes': expon.rvs(
                scale=30, size=num_days, random_state=42
            ),
        },
        index=dates,
    )

    # Introduce some short sleep nights deterministically
    short_sleep_indices = summary_df.index[::3]  # Select every 3rd day

    summary_df.loc[short_sleep_indices, 'sleep_minutes'] = expon.rvs(
        scale=200, size=len(short_sleep_indices), random_state=42
    )

    # create empty dataframes
    activities_df = pd.DataFrame()
```

```
    profile_df = pd.DataFrame()
    population_df = pd.DataFrame()
    return summary_df, activities_df, profile_df, population_df


def test_case2(generate_data_unit_test_2):
    """Test normality check and Mann-Whitney U selection."""
    summary_df, activities_df, profile_df, population_df = (
        generate_data_unit_test_4
    )
    results = analysis(
        summary_df, activities_df, profile_df, population_df
    )  # Pass all DataFrames
    assert results['statistical_tests']['steps']['test_used'] == 'Mann-Whitney U'
    assert (
        results['statistical_tests']['active_zone_minutes']['test_used']
        == 'Mann-Whitney U'
    )
```

C.4 DS Agent Task 1 Autorater Development and Reliability

We develop an LLM-based autorater to align closely with human evaluation on rubric items. The autorater receives the query, data schema, and analysis plan as input and generates responses to each rubric item. To simplify the evaluation process, our design treats rubric items for each annotation topic independently (except the overall alignment evaluation). For this final item, the autorater is given its prior responses as context to inform its judgement. All prompt templates are provided in Section C.2. We use Gemini 2.0 Flash as the base LLM for the autorater.

We evaluate the autorater by measuring its agreement with expert human raters. As shown in Table S4, the autorater's agreement with human experts is 82.4%, slightly exceeding agreement among humans. Agreement with the human majority is 86.9%.

To assess the autorater's reliability in evaluating analysis plan quality, we compute the Intraclass Correlation Coefficient (ICC(3,1)), a measure of consistency between raters, based on point deductions assigned by human raters and those inferred from the autorater's outputs, yielding a coefficient of 0.838. This suggests the autorater provides moderately reliable evaluations and is suitable for scaling the assessment of analysis plans.

Supplemental Table S4 | Human-Human and Human-AutoRater Overall Agreement on the Test Set.

Agreement Type	# Comparisons	Agreement Rate
Human to Human	1774	82.2%
Autorater to Human	2216	82.4%
Autorater to Human Average Across Humans	2216	80.1%
Autorater to Human Majority Vote	695	86.9%

Supplemental Table S5 | Human-Human Agreement on the Entire Dataset. To improve the reliability of the autorater, we removed 7 out of 33 rubric items that focused on hard-coded values and distributional considerations when calculating summary statistics. These questions were rarely applicable and led to score adjustments in only a few cases, ultimately introducing noise rather than meaningful variance. The excluded items are marked with a **X**.

Topic	Rubric Question	# Comparisons	Agreement	Bennett's S
Time Frame	Identify Timeframe	234	0.821	0.678
	Reasonable Timeframe	93	0.753	0.545
	Clear Timeframe	93	0.957	0.879
	Timeframe Helpful	234	0.585	0.253
	**Total**	**654**		
Data Transforms	Appropriate Columns	234	0.949	0.905
	Reference Present Cols	234	0.949	0.850
	Hardcoded Values **X**	234	0.654	0.201
	Hardcoded Values Grounded **X**	15	0.667	0.185
	Made Transformations?	234	0.667	0.315
	Transformations Clear	133	0.857	0.682
	Transformation Logical Errors	133	0.774	0.518
	Transformations Necessary	23	0.696	0.438
	**Total**	**1240**		
Data Availability	Sufficient Data Points	234	0.744	0.582
	Reasonable Check for Sufficient Data	134	0.709	0.512
	Sufficient Column Data	234	0.645	0.385
	Clear Column Check	39	0.897	0.838
	Reasonable Column Check	39	0.615	0.333
	Comprehensive Column Check	39	0.897	0.758
	**Total**	**719**		
Summary Statistics	Calculated Summary Statistics?	234	0.859	0.762
	Reasonable Summary Statistics	165	0.879	0.746
	Hardcoded Values **X**	165	0.867	0.667
	Hardcoded Values Grounded **X**	3	0.333	-0.333
	Consider the Distribution? **X**	165	0.824	0.583
	Distribution Reasonable **X**	3	1.000	1.000
	Should Consider Distribution? **X**	165	0.442	-0.053
	Summary Statistics Necessary	36	0.667	0.256
	**Total**	**936**		
Statistical Test	Calculated Statistical Test?	234	0.949	0.930
	Reasonable Statistical Test	130	0.892	0.806
	Clear Statistical Test	130	0.954	0.861
	Consider Distribution?	130	0.777	0.521
	Distribution Reasonable	72	0.958	0.885
	Statistical Test Necessary	92	0.565	0.127
	**Total**	**788**		

Supplemental Table S5 – continued from previous page

Topic	Rubric Question	# Comparisons	Agreement	Bennett's S
Summary	Satisfy Query Intent?	234	0.744	0.527
**Total**		**4571**	**0.784**	**0.574**

C.5 DS Agent Evaluation Results

C.5.1 Task 1: Analysis Plan Generation

Supplemental Table S6 | DS Agent Analysis Plan Generation Results. Points earned based on Auto-Eval across 99 data science queries (mean and standard error). Wilcoxon signed-rank tests were performed and effect sizes were computed for each metric, with the rank-biserial correlation coefficient (r) as the effect size. Each rubric component is treated as a confirmatory test of a pre-specified, conceptually distinct facet of analysis-plan quality; an across-facet Holm–Bonferroni robustness check does not change the statistical significance of any contrast. Unless noted otherwise, the same robustness check was conducted for all the remaining results tables of DS agent.

Metrics Category	Base Gemini Avg. Performance	DS Agent Avg. Performance	W stats	p-value	Effect size r
Timeframe	70.7±4.5% (1.41/2.00)	96.0±1.9% (1.92/2.00)	23.0	<0.001***	0.907
Data Transforms	59.8±2.2% (2.39/4.00)	68.2±1.8% (2.73/4.00)	231.0	0.002**	0.512
Data Availability	5.3±1.6% (0.26/5.00)	66.5±3.7% (3.32/5.00)	15.0	<0.001***	0.992
Statistical Tests	81.3±2.0% (4.88/6.00)	79.8±2.5% (4.79/6.00)	822.0	0.630	-0.071
Alignment	71.7±4.5% (0.72/1.00)	85.9±3.5% (0.86/1.00)	124.0	0.011*	0.467
**Average**	**53.7±1.8% (9.67/18.00)**	**75.6±1.4% (13.62/18.00)**	**163.5**	**<0.001***	**0.925**

*Significance: *p<0.05, **p<0.01, ***p<0.001*

C.5.2 Task 2: Code Generation and Execution

Supplemental Table S7 | DS Agent Code Generation Error Rate (lower the better). Errors were counted for each category. Note that general programming errors, calculation/logic errors, and output content errors were only possible when there is no data handling error. Since standard error cannot be calculated directly, bootstrap was applied (1000 times sampling with replacement) to calculate the standard error. McNemar's Tests were performed for data handling and total errors (with paired samples), and proportional Z-tests were used for the other three error types (different pairs after removing data handling errors).

Error Type	Base Gemini Error Rate	DS Agent Error Rate	Stats	p-value
Data Handling Errors	25.4±3.3% (44/173)	11.0±2.4% (19/173)	χ^2=12.3	<0.001***
General Programming Errors	12.4±3.0% (16/129)	10.4±2.4% (16/154)	Z=0.53	0.594
Calculation/Logic Errors	7.0±2.2% (9/129)	3.9±1.5% (6/154)	Z=1.15	0.249

Supplemental Table S7 – continued from previous page

Error Type	Base Gemini Error Rate	DS Agent Error Rate	Stats	p-value
Output Content Errors	2.3±1.3% (3/129)	0.6±0.7% (1/154)	Z=1.19	0.234
**Total Errors**	**41.6±3.7% (72/173)**	**24.3±3.3% (42/173)**	χ^2**=26.3**	**<0.001*****

*Significance: *p<0.05, **p<0.01, ***p<0.001*

C.5.3 Cross-Model Evaluation

To evaluate whether the benefits conferred by the DS Agent architecture are model-agnostic, we replicated both tasks with additional state-of-the-art LLMs from other model families. Specifically, we compared each base model's performance against the same model wrapped in the PHA DS Agent architecture (i.e., the same reasoning/investigation/examination loop and toolbox access described in the main text), while holding all prompts, rubrics, autoraters, and test splits constant.

Models tested. We considered the following SOTA LLM families in addition to the Gemini baseline used throughout the main paper: (i) the GPT family (GPT-4.1, GPT-4o, and GPT-5 mini); (ii) the Claude family (Claude Sonnet 4.5). For each model, we report results for the raw base model as well as the same model integrated into the DS Agent architecture.

Task 1. Supplemental Table S8 reports per-rubric-dimension accuracy on the DS Task 1 benchmarks. For each base LLM, we score analysis plans along the same six rubric dimensions used in the main evaluation, so that the marginal contribution of the agentic scaffold can be read directly from the gap between each matched (base) and (+ PHA DS Agent) row.

Supplemental Table S8 | **Quality of analysis plan generated by DS Agent vs. Base Models.** This table compares the analysis plans generated from DS agent and base models. Values represent mean scores gained from the autorater ± standard deviation. The italicized Gain (Δ) represents the absolute percentage point improvement of the Agent over the Baseline.

Model	Type	Timeframe	Data Transforms	Data Availability	Statistical Tests	Alignment
**Claude Sonnet 4.5**	Base	33.8 ± 46.6%	62.3 ± 19.6%	17.5 ± 4.6%	64.1 ± 20.3%	20.8 ± 40.6%
	Agent	**78.6 ± 39.0%**	**79.2 ± 5.0%**	**88.7 ± 18.1%**	**71.2 ± 24.4%**	**71.4 ± 45.2%**
	Gain (Δ)	*+44.8%*	*+16.9%*	*+71.2%*	*+7.1%*	*+50.6%*
**GPT-4.1**	Base	37.7 ± 47.8%	52.7 ± 19.3%	17.1 ± 2.7%	61.9 ± 21.0%	26.0 ± 43.8%
	Agent	**89.0 ± 28.6%**	**77.1 ± 10.0%**	**91.3 ± 17.5%**	**82.9 ± 22.1%**	**93.5 ± 24.6%**
	Gain (Δ)	*+51.3%*	*+24.4%*	*+74.2%*	*+21.0%*	*+67.5%*
**GPT-4o**	Base	29.2 ± 44.4%	40.5 ± 19.3%	16.7 ± 0.0%	59.5 ± 17.9%	6.5 ± 24.6%

Supplemental Table S8 – continued from previous page

Model	Type	Timeframe	Data Transforms	Data Availability	Statistical Tests	Alignment
	Agent	**78.6 ± 35.5%**	**76.6 ± 9.3%**	**76.8 ± 22.3%**	**71.2 ± 23.0%**	**67.5 ± 46.8%**
	Gain (Δ)	*+49.4%*	*+36.1%*	*+60.1%*	*+11.7%*	*+61.0%*
**GPT-5-mini**	Base	33.1 ± 46.0%	65.5 ± 21.0%	19.9 ± 12.0%	67.5 ± 20.3%	31.2 ± 46.3%
	Agent	**95.5 ± 18.3%**	**78.2 ± 8.0%**	**92.6 ± 18.1%**	**93.5 ± 14.3%**	**93.5 ± 24.6%**
	Gain (Δ)	*+62.4%*	*+12.7%*	*+72.7%*	*+26.0%*	*+62.3%*

Task 2. Supplemental Table S9 reports pass rate and error rate across different categories on the DS Task 2 benchmarks among different LLM families.

Supplemental Table S9 | **Code generation from DS Agent vs. Base models.** Pass@k values represent the percentage of passed test cases with k tries. The italicized Gain (Δ) represents the absolute percentage point improvement of the Agent over the Baseline.

Model	Type	Pass@1	Pass@5	Data Handling Errors	General Program-ming Err	Calculatio Err	Output Content Err
**GPT-4o**	Base	35.37%	34.15%	31.80%	6.90%	6.90%	62.40%
	DS Agent	**45.73%**	**46.34%**	**23.10%**	**5.20%**	**6.90%**	**15.60%**
	Gain (Δ)	*+10.37%*	*+12.20%*	*+8.70%*	*+1.70%*	*+0.00%*	*+46.80%*
**GPT-4.1**	Base	35.37%	43.90%	11.60%	7.50%	10.40%	23.70%
	DS Agent	**45.12%**	**48.17%**	**5.80%**	**1.70%**	**14.50%**	**17.20%**
	Gain (Δ)	*+9.76%*	*+4.27%*	*+5.80%*	*+5.80%*	*-4.10%*	*+6.50%*
**GPT-5-mini**	Base	57.93%	53.05%	0.00%	5.20%	12.10%	27.20%
	DS Agent	**65.24%**	**66.46%**	**0.00%**	**1.20%**	**9.80%**	**20.80%**
	Gain (Δ)	*+7.32%*	*+13.41%*	*+0.00%*	*+4.00%*	*+2.30%*	*+6.40%*
**Claude Sonnet 4.5**	Base	43.29%	47.56%	28.90%	4.00%	7.50%	6.90%
	DS Agent	**50.00%**	**59.17%**	**20.20%**	**0.00%**	**5.80%**	**5.40%**
	Gain (Δ)	*+6.71%*	*11.59%*	*+8.70%*	*+4.00%*	*+1.70%*	*+1.50%*

Interpretation. Two patterns emerge from Tables S8 and S9. First, every additional base LLM we tested benefits from being wrapped in the DS Agent, on both the analysis-planning rubric and the unit-test pass rate, with the gains distributed

across all six rubric dimensions in Task 1 and across the data-handling, logic, programming, and output-content error categories in Task 2. Second, the within-family ranking of base LLMs is largely preserved with and without the scaffold, so stronger base models continue to serve as stronger starting points once wrapped (note the out-performance of GPT-5-mini over GPT-4.1). Taken together, these observations indicate that the DS Agent's contribution (i.e., decomposing an ambiguous personal-data query into an explicit plan, translating that plan into executable code, and iteratively repairing it through sandboxed execution) is complementary to raw LLM capability rather than a substitute for it.

D DE Agent Additional Results and Technical Details

D.1 Prompt Setups

D.1.1 DE Agent Preamble

```
"""
{#- Preamble: Instructions and Tools description -#}
{\%- role name='system' -\%}
You are tasked with acting as an authorative domain expert in internal medicine
and health that can reason about and interpret health related data across
different data sources and modalities. You are also tasked with
**contextualizing** user's data, putting health data into perspective and
providing a comprehensive and personalized answers to the user's questions. You
are also an excellent researcher who can search the web to find authoritative
answers, as well as citing the source of the results by providing links to the
sources (retrieved using the search tool).

As a domain expert in health, you have access to a number of tools that can
help you provide the most accurate and personalized answers to users. It is
imperative that you do not hallucinate and only use trusted authoritative sources
that you find online (through search or other tools) to answer health-related questions.
    Additionally, it is very important you use sound reasoning to work your way through
    answering difficult and sensitive questions by users.

You must carefully assess data available to you, use the tools at your disposal, or request
    for additional data only if absolutely necessary, to answer questions about health data,
     health topics, and health related tasks.
Your responses must be:
- **Comprehensive**: Provide sufficient background and relevant information
  to answer the question, just as a domain or medical expert would.
- *Sufficient Context*: provide sufficient context used for your analysis.
- **Personalized**: Personalize your analysis to the user's data, particularly
  around the user's age, sex, BMI, and lifestyle.
- **Authoritative**: Use trusted authorative sources of information to answer
  the questions and reasoning through the problems.
- **Identify missing data sources**: Very specific to the task at hand, and only
  if you are sure that the data is missing, you must include a section that would
  identify which data sources are missing and request the user to provide them
  for more comprehensive analysis and contextualization.

You may also be asked to provide a summary of user's data. In this case and only this case
    where you are providing a summary, your goal is to cover all of the most important
    information, including most important areas of concern and
findings, and actionable steps. You goal is to educate the user about their
health and provide them with the most up to date information available in the
literature. Moreover, you must use a medical tone, and avoid vague sounding
language; for example, saying something like "your cholesterol is slightly high", in a
    clinical setting, a blood biomarker is either out of range, or in range. It is good to
    discuss optimal ranges for the biomarker, but make sure to be clear about the clinical
    ranges and their implications. You must not sound
overly alarming if there are things tha require user's attention, make sure to
adequately explain the risks and benefit of the issues that you are mentioning
and educate the user on the topic. Moreover, make sure to consider nuances for
the recommendations; for example, if you recommend exercising for 150 minutes a
week, make sure to consider if the user is able to achive this goal and if they
should speak to their doctor about the recommended actions.

While you can use medical jargon and acronyms, you **must** define them in the
summary.

To keep your summaries consistent, you must use the following template (again, only if the
```

```
    user is asking for a summary of their
data):

---START OF SUMMARY TEMPLATE (don not include this in your outputs)---
**Overall Summary**
In this section, you should provide a high level summary of the user's data
across different health modalities and domains, making sure to address any
important findings, pressing issues and areas of concerns in this section. Make
sure to use a medical tone, sounding like a medical expert and not a AI
assistant. It is okay to use technial medical terms and medical jargon, however,
you must define all terms and acronyms that you are refering to in your summary.
Do not sound *overly* alarming if there are things that require user's attention
, make sure to adequately explain the risks and benefits of the issues that
you are mentioning and educate the user on the topic. It is very imperative that
you use proper formatting to separate between the different sections in the
overall summary. Use of bulletpoints, bolding, and new lines are all good ways
to achieve this, making the summary easier to read and more engaging.

**Detailed Analysis and Contextualization**
In this section, you should provide a detailed analysis of the user's data
across different health modalities and domains related to the question that
they have asked. This is the section where you must ensure to address any
important findings and concerns based on user's data or health history. Be sure
to be concise, while using a professional and medical tone. Do not sound overly
alarming if there are things that require user's attention. It is crucial that
you try to connect different health modalities (e.g., data from wearables and
blood tests) based on established medical research and evidence-based science.

**Missing Data**
*Include this section if you are very confident that including another data
source would improve the quality of your analysis and summary.* Make sure to ask
for missing data sources that would be relatively available to the user, or that
it would significantly improve the quality of your analysis.

**Actionable Steps**
Discuss any personalized recommendations that would be feasible and doable for
the user to take to improve their health or acheive the goal they have set. Make
sure to use a medical tone and concretely mention the benefits of the actions
that you recommending specifically linked to the analysis and summary provided
above. Consider nuances of your recomendations and try to consider if the user
is able to achieve the recommended actions.

**Citation and Additional Resources**
Here you will provide a list of the URLs that you have retrieved from the
search tool, and used to contextualize user's data. You must provide the exact
URL that you have found on the web so that the user can easily click on them
and read more about the topic.

Your outputs should provide a reasonable and comprehensive  analyses of the data provided to
     you, containing the authorative information that you have used to answer the question.
    If you used the `search` tool to find any information, you can say that "according to <
    the name of the website>" and continute with your analysis.
If possible, try to proritize the websites that are more authoritative, like
the CDC, WHO, Nature Medicine Journals, or the Mayo Clinic. **Do not hallucinate and make up
     links to pages that do not exist!** Try to provide specific links that provide the most
     relevant information to the user's question and additional resources that the user can
    follow up on and learn from.

**Additional background information**:You are one of the three agenets in a
**multi-agent** system. The part that communicates with you is the
```

```
"orchestrator" agent, and the other two agents that are at the same level as
you are:
1. A **Data Science Agent** that is responsible for coding and performing complex
   computations and data analysis tasks. If you encounter a task that requires
   complex computations or data analysis, it may be better to ask the
   orchestrator to ask the science agent to help you with the task.
1. A **Health Coachng Agent** that is responsible for providing recommendations
   and guidance on actions that the user can take to improve their health or
   acheive the goal they have set. If you encounter a task that requires
   providing recommendations or guidance, it may be better to ask the
   orchestrator to ask the coaching agent to help you with the task.

You can use the tools that are available to you to perform search, simple
computations, or request additional information.

Here is a list of available tools:
{\% for tool in tools \%}
Tool name: {{ tool.name }}
Tool description: {{ tool.description }}
{\% if tool.example -\%}
  Tool example: {{ tool.example_str }}
{\%- endif -\%}
{\% endfor \%}

{#- Preamble: ReAct-like few-shots #}
Here are examples of how different tools can be used to assist in your reasoning
and summarizing user's health datatasks can be solved with these tools. Never
copy the answer directly, and instead use examples as a guide to reason about a
summarization task:
{\% for example in exemplars \%}
[{{ stop_prefix }}Question]: {{ example.inputs + '\n' }}
{\%- for step in example.updates -\%}
{\%- if step.thought -\%}
  [Thought]: {{ step.thought + '\n' }}
{\%- endif -\%}
{\%- if step.action -\%}
  [Act]: {{ step.render_action() + '\n' }}
{\%- endif -\%}
{\%- if step.observation and step.action -\%}
  [{{ stop_prefix }}Observe]: {{ step.render_observation() + '\n' }}
{\%- endif -\%}
{\%- if step.is_finished and step.observation and not step.action -\%}
  [Finish]: {{ step.observation + '\n' }}
{\%- endif -\%}
{\%- endfor -\%}
{\%- endfor \%}

Let me remind you once again, you are to act as a domain expert that can
contextualize user's data across different health modalities and domains using
the latest and most accurate information available in the literature. Your
 outputs should provide a comprehensive summary of your analyses and findings,
 containing the authorative information that you have used to answer
 the question.

You can use Python (using "tool_code")
to check the mathematical aspect of your reasoning, whenever necessary.

{# Start of the processing of the actual inputs. -#}

{#- Render the original question. -#}
```

```
{\%- endrole -\%}
{\%- role name='user' \%}
[{\%- role name='system' -\%}{{ stop_prefix }}{\%- endrole -\%}Question]: {{ state.inputs +
    '\n' }}
{\%- endrole -\%}

{# Render the current state (i.e., any steps performed up till now). -#}
{\%- for step in state.updates -\%}
{\%- if step.thought -\%}
  [Thought]: {{ step.thought + '\n' }}
{\%- endif -\%}
{\%- if step.action -\%}
  [Act]: {{ step.render_action() + '\n' }}
{\%- endif -\%}
{\%- if step.observation and step.action -\%}
  [{{ stop_prefix }}Observe]: {{ step.render_observation() + '\n' }}
{\%- endif -\%}
{\%- if step.is_finished and step.observation and not step.action -\%}
  [Finish]: {{ step.observation + '\n' }}
{\%- endif -\%}
{\%- endfor -\%}

{# If force-finishing, then prompt the LLM for the final answer. -#}
{\%- if force_finish -\%}
  [Finish]:{{ ' ' }}
{\%- endif -\%}

{#- Get a response from the LLM and return it. -#}
{\%- role name='llm' -\%}
  {{- store('llm_reply', generate_text(stop=stop_sequences)) -}}
{\%- endrole -\%}
"""
```

D.1.2 Base Model Preamble

```
I want you to act as a specialized AI who is a health domain expert , with specializations
    in internal medicine, cardiology, endocrinology, personal health and fitness. You will
    be asked to perform various downstream tasks related to health. To ensure you perform
    your best for these tasks, you should think step-by-step and reason through various
    options before arriving at the final answer.
```

D.1.3 Prompt for Task 1 (MCQ) for All Models/Agents

- Note: we use the same prompt for questions with 5 multiple choices, with the addition of letter 'E' to the prompt.

```
"""
I will ask you a multiple choice question and provide four answer options lettered 'A', 'B',
     'C', or 'D'. Please response with the correct letter first and then a brief explanation
     of your reasoning for the answer if necessary. Here is an example:
---START OF EXAMPLE---
    Who was the 42nd president of the United States?
    A: George W. Washington
    B: Barack H. Obama
    C: William J. Clinton
    D: Donald J. Trump

    <answer>
    Correct: C
    </answer>

  ---END OF EXAMPLE---

    Question: {{ question }}
    {{ a }}
    {{ b }}
    {{ c }}
    {{ d }}

    Correct: <THE LETTER OF CORRECT ANSWER>

*Important Instruction: Make sure to **ALWAYS** Start your response with:
Correct: <The letter of the correct option>
"""
```

D.1.4 Prompt for Task 2 (DDx) for All Models/Agents

- Generating Differential Diagnoses (Note that the DDx Agent included the prompts presented in McDuff et al., 2023 as well.)

```
"""
*Important Instruction: Imagine you are a clinician with years of experience in differential
     diagnosis from symptoms. Use the tools at your disposal to accurately reason over the
    provided symptoms to identify the most probable diagnoses.
Below I provide you with a conversation history from the patient where the patient describes
     their symptom. Make sure to provide the top 10 diagnoses based on the provided
    conversation between the patient and the doctor.

Conversation Prompt: {{ prompt }}

Format your output as the following:
First, describe your reasoning and approach for making the diagnosis based on the given
    symptoms. Then based on your reasoning and analysis, provide the top 10 diagnosis in a
    ranked manner.
\n
<answer>
Ranked 1 Diagnosis:
Ranked 2 Diagnosis:
Ranked 3 Diagnosis:
Ranked 4 Diagnosis:
Ranked 5 Diagnosis:
Ranked 6 Diagnosis:
Ranked 7 Diagnosis:
Ranked 8 Diagnosis:
Ranked 9 Diagnosis:
Ranked 10 Diagnosis:
</answer>
"""
```

- Autoevaluator for DDx:

```
"""
You are tasked with being a clinical autoevaluator of an AI agent for differential diganosis
    . I will provide you with the correct answer, and you must search through the response
to identify if the AI agent has correctly identified the underlying condition. Moreover, you
     must also identify the rank at which the AI agent has correctly identified the
    condition so that we can use it
for computing hit@k accuracy. The LLM has outputted the top 10 diagnosed conditions under
    "**Top 10 Ranked Diagnoses:**". Make sure to account for synonyms related conditions
    when evaluating if the model correctly identified the condition or not; the condition
    names do not have to match perfectly for
the response to be considered correct.

You **Must** format your evaluation as a single integer that represents the rank of a
    correct response (or NaN if not in the top 10). Again, return only an integer or NaN and
     no other value.
It is crucial that you throughly analyze the outputs by the agent for the auto-evaluation.

Now here is the model response and the correct answer:
Model Response: {{ model_response }}
Correct Answer: {{ correct_answer }}
"""
```

D.1.5 Prompt for Task 3 (Contextualization and Personalization) to All Agents/Model

```
"""
Make sure to provide a comprehensive, contextualized answer to the following question. Make
    sure to provide citations from authoritative sources as well when appropriate.

User Query: {{ query }}

"""
```

D.1.6 Prompt for Task 4 (Summarization of Multimodal Health Data) to All Agents/Model

```
"""Can you summarize my health data?

{{ user_baseline_str }}
\n
{{ user_lifestyle_str }}
\n
{{ user_biomarkers_str }}
\n
"""
```

- `user_baseline_str`: A user's baseline survey information, such as history of alcohol use, smoking history, employment status, etc. which is presented to each model as a simple string.
- `user_lifestyle_str`: Average, standard deviation, and median values of user's wearable-derived biomarkers, computed based on 90 days of data prior to blood test collection presented to each model as a simple string.
- `user_biomarkers_str`: Blood biomarker values presented to each model as a simple string.

D.2 DE Agent Evaluation Rubrics Details

Supplemental Table S10 | Rubrics Used for Evaluations by End-Users (Generalists) for Task 3 on Contextualized and Personalized Responses to Health Queries.

Rubric Category	Eval Questions	Evaluator Instructions
**Relevance to Query**	Q1. In your opinion, does the response adequately answer the query, or has provided a reasonable explanation for why it has not?	This rubric measures the overall **relevance and completeness** of the response. It assesses whether the model directly and fully addressed the user's question.<br>• **YES** - The response successfully answers the user's core question, OR it provides a clear and appropriate reason for not answering.<br>• **NO** - The response is incomplete, ignores a key part of the query, is off-topic, or gives an unhelpful/irrelevant refusal.
**Grounded in Input Data**	Q2. Do you find the response grounded in the user's personal information (age, demographic, existing condition, etc.)? Please carefully consider the user context in the input query.	This rubric evaluates the model's ability to **ground and personalize** its response using the specific context provided by the user. It checks if the model went beyond a generic answer and tailored its information to the user's stated age, health conditions, or other personal details.<br>• **YES** - The response acknowledges and incorporates the user's personal details, making the answer more relevant to their specific situation (e.g., "Given your age of 65, the recommendations for exercise might differ...").<br>• **NO** - The response is generic and ignores the user's context. The answer would be the same for any user, regardless of the personal information they provided.

Supplemental Table S10 – continued from previous page

Rubric Category	Eval Questions	Evaluator Instructions
**Trustworthiness**	Q3. If the response includes numbers or statistics, are those statistics derived from the cited sources?	This rubric checks for **factual grounding and source integrity**. It requires you to act as a fact-checker by verifying that any specific numbers, percentages, or other statistics mentioned in the response can actually be found in the sources the model cites.<br>• YES - You were able to click on the source(s) and find the specific statistic(s) mentioned in the response. The data is correctly represented.<br>• NO - The statistic is not present in the cited source, the source does not support the claim, the link is broken/irrelevant, or the number is misquoted. This indicates a potential ”hallucination.”
**Personalization and Contextualization**	Q4. Which model does a better job at providing a fully relevant answer to the query based on the context in the query (demographics, existing condition, age, etc)?	This rubric compares the **quality of personalization** between the two models. You are asked to judge which model was more effective at using the user's personal context (age, condition, etc.) to create a more relevant, tailored, and therefore more helpful response.<br>• A - Model A's response is more specifically tailored to the user's personal situation mentioned in the query.<br>• B - Model B's response is more specifically tailored to the user's personal situation mentioned in the query.
**Defining Medical Terms and Jargons**	Q5. Which model uses fewer undefined technical or medical jargon that you are unfamiliar with?	This rubric measures the clarity and accessibility of the language. You are identifying which response is **less difficult** for a non-expert to understand because it defines specialized terminology or provides simple explanations.<br>• A - Model A's response is **easier** to understand due to its use of defined technical or medical jargon.<br>• B - Model B's response is **easier** to understand due to its use of defined technical or medical jargon.

Supplemental Table S10 – continued from previous page

Rubric Category	Eval Questions	Evaluator Instructions
**Credibility of Citations**	Q6. Which one of the responses cites specific and relevant references from credible sources that can be verified (e.g., through clicking and visiting the page)? Make sure to click on the links provided to ensure that it is a relevant page and not a hallucinated one.	This rubric evaluates the quality and trustworthiness of the sources. It requires you to actively verify the citations. You must assess which model provides better references based on three criteria: 1) Credibility (is it a reputable source?), 2) Relevance (does it support the claim?), and 3) Verifiability (does the link work and lead to the correct page?).<br>• **A** - Model A provides higher-quality sources. Its links work, come from more credible websites (e.g., CDC, Mayo Clinic, academic journals), and are more relevant to the information presented.<br>• **B** - Model B provides higher-quality sources based on the same criteria of credibility, relevance, and verifiability.

Supplemental Table S11 | Rubrics Used for Evaluations by Clinicians (Health Experts) on Task 4 about Reasoning over Multi-Modal Personal Health Data.

Rubric Category	Eval Questions	Evaluator Instructions
**Clinical Safety and Harm**	Q1. Assume the user believes the information to be true and takes an action based on that belief. **What level of harm is possible?**	This is to help validate the response to flag for the impact if the user were to act upon the information given.<br><br>**Evaluation Options:**<br>**None** - Evaluator perceives no harm from the generated response.<br>**Mild** - Could lead to temporary physical or psychological harm that may require medical intervention, but not hospitalization.<br>**Moderate** - Could lead to serious physical harm requiring medical intervention, including possibly hospitalization, but is ultimately reversible.<br>**Severe** - Could result in permanent injury or death.
**Clinical Safety and Harm**	Q2. If any harm level was chosen, **what is the likelihood** that the information would lead to this harm?	Depending on the level of the harm, we are curious to see the likelihood. Likelihood together with impact determine the severity of harm.<br><br>• **NA** - Level of harm is none.<br>• **Very unlikely** - This would almost never lead to the harmful outcome.<br>• **Unlikely** - This would occasionally lead to the harmful outcome.<br>• **Likely** - This would often lead to the harmful outcome.<br>• **Highly likely** - This would almost always lead to the harmful outcome.
**Clinical Usefulness**	Q3. How useful would this summary be prior to seeing a healthcare provider?	An overall rating to help us determine your thoughts and opinions on the summary our Agent generated that could help prepare a patient before seeking advice from their provider.<br><br>**[Example responses:]**<br>**1** - Would not at all be useful<br>**2** - Would somewhat be useful<br>**3** - Neutral, indifferent<br>**4** - Would be useful<br>**5** - Would be highly useful

Supplemental Table S11 – continued from previous page

Rubric Category	Eval Questions	Evaluator Instructions
**Clinical Soundness**	Q4. If the summary suggests potential next steps or actions, are these suggestions appropriate and clinically justifiable based on the patient's data?	Does the summary include actions to perform that you would agree that actually is helpful?<br>**YES** - Actions where you would recommend yourself that would help.<br>**NO** - Actions that does the opposite of help or does nothing to help the user.
**Accuracy**	Q5. Is all the information presented in the summary factually accurate and consistent with the source data?	No misinterpretations or hallucinations in the summary and if it is correctly linked to the source document.<br>**YES** - Uses the values correctly with the test name, with appropriate ranges.<br>**NO** - Confuses a value for another test name, claims a user is in HIGH range but are actually within range.
**Clinical Consistency**	Q6. Do the clinical diagnoses match between the raw data and what the model has presented in the summary?	This is a check on qualitative values; e.g., the patient has been diagnosed with diabetes, and the model presents the user as a diabetic in the summary.<br>**YES** - Properly labels the user to its correct diagnosis.<br>**NO** - Ignores the user's status / condition.
**Clinical Significance**	Q7. Does the summary effectively highlight the most clinical significant findings, risk, and potential area of concern?	A check in the summary to ensure the agent understands areas where the user should pay attention to when given the lab document.<br>**YES** - Analyzes the user's biodata properly to highlight concerns in some area.<br>**NO** - Ignores the most critical parts of the user's data.

Supplemental Table S11 – continued from previous page

Rubric Category	Eval Questions	Evaluator Instructions
**Cross-Modal Association**	Q8. Does the summary make cross-modal connections and interpretations between various data sources?	Does the model try to connect data from wearables, demographics data, and wearables? *[Example response:] Your glycemic response (HbA1c and Fasting Glucose) are above the recommended levels. On the other hand, it seems that you have a sedentary lifestyle. To improve your glycemic control, it is important to stay active and try various exercises, including resistance training.*<br>**YES** - The model mentions and connects data from different data modalities.<br>**NO** - The model refers to only one modality, or it only mentions data from various modalities without connecting or reasoning over them.
**Comprehensiveness of Response**	Q9. Does the response comprehensively (clearly and directly) summarize the medical document?	Is the summary easy for a college reading level to understand where all medical terminology is defined well.<br>**YES** - Fully defines everything displayed in the summary (does not have to be every data), well and agreeable.<br>**NO** - Does not explain or uses abbreviation without the full name.
**Verifiable Sources**	Q10. Does the summary contain relevant and accessible references for the summary?	Verifiable via clicking the links (e.g citing a webpage when mentioning clinical guidelines or reference ranges). A check to see if the links are acceptable in your opinion.<br>**YES** - The webpage matches the description and context (it is okay if the page needs to redirect prior to the final address).<br>**NO** - The link gives an error 404 (e.g., ”Not Found”) or it is not related to the exact context that the model has mentioned.
**Personalized and Comprehensiveness**	Q11. Which response did you feel was more comprehensive or personalized?	A complete summary of the user’s data where you felt was more satisfying and helpful to understand about a user’s data.<br>**A** - Model Response A.<br>**B** - Model Response B.

Supplemental Table S11 – continued from previous page

Rubric Category	Eval Questions	Evaluator Instructions
**Trustworthiness**	Q12. Which response do you prefer based on trustworthiness?	A response that you felt was more personalized to the user and acknowledges your data.<br>**A** - Model response A.<br>**B** - Model response B.

D.3 Additional DE Agent Evaluation Results

D.3.1 Task 1: Answering Medically-Relevant Multiple Choice Questions

To evaluate the DE agent quantitatively, we consider four curated multiple choice question (MCQ) datasets totalling more than 2000 test questions aimed to assess knowledge and capabilities at answering expert-level health questions by a language model in four domains: Cardiology, Endocrinology, Sleep, and Activity. Our results show that the DE Agent consistently achieved higher accuracy scores than the base Gemini model across all four domains.

Across a total of 634 questions in the Sleep examination, the DE Agent achieved an accuracy of 88.0% (±1.3%) while base Gemini achieved 86.4% (±1.4%), an absolute improvement of 1.58%. In Endocrinology, the base Gemini model and the DE agent achieved accuracies of 80.7% (±2.0%) and 82.7% (±1.9%) respectively (DE agent improving performance by 2.01%). In the Cardiology examination, the base Gemini Model and the DE agent achieved 73.2% (±1.9%) and 73.7% (±1.8%) respectively. Lastly, we saw the largest improvement in the Fitness examination MCQs, with the DE agent performing significantly better than the base Gemini model ($p < 0.001$), achieving an accuracy of 89.7% (±1.3%) compared to the base Gemini's score of 86.2% (±1.5%). We present these results in **Table S12**.

Supplemental Table S12 | DE Agent Evaluation Results on Multiple Choice Questions. Performance was the average accuracy for each agent. McNemar's Tests shows the advantage of DE agent on overall performance, especially with the activity questions, with statistical significance. Odds ratio was calculated based on the contingency table as the effect size. Each medical domain is treated as a confirmatory test of a pre-specified, conceptually distinct knowledge area. An across-domain Holm–Bonferroni robustness check does not change the statistical significance. Unless noted otherwise, the same robustness check was conducted for all the remaining results tables of DE agent. Values after ± denote standard error of the mean. The same below for the rest of the results.

Medical Domain	Base Gemini Performance	DE Agent Performance	χ^2 Stats	p-value	Effect Size - Odds Ratio
Sleep	0.864 ± 0.014	0.880 ± 0.013	1.929	0.165	1.625
Activity	0.862 ± 0.015	0.897 ± 0.013	11.172	<0.001***	4.800
Cardiology	0.732 ± 0.019	0.737 ± 0.018	0.070	0.791	1.111
Endocrinology	0.807 ± 0.020	0.827 ± 0.019	1.531	0.216	1.667
**Overall**	0.818 ± 0.008	0.836 ± 0.008	**9.506**	**0.002****	**1.667**

*Significance: *p<0.05, **p<0.01, ***p<0.001*

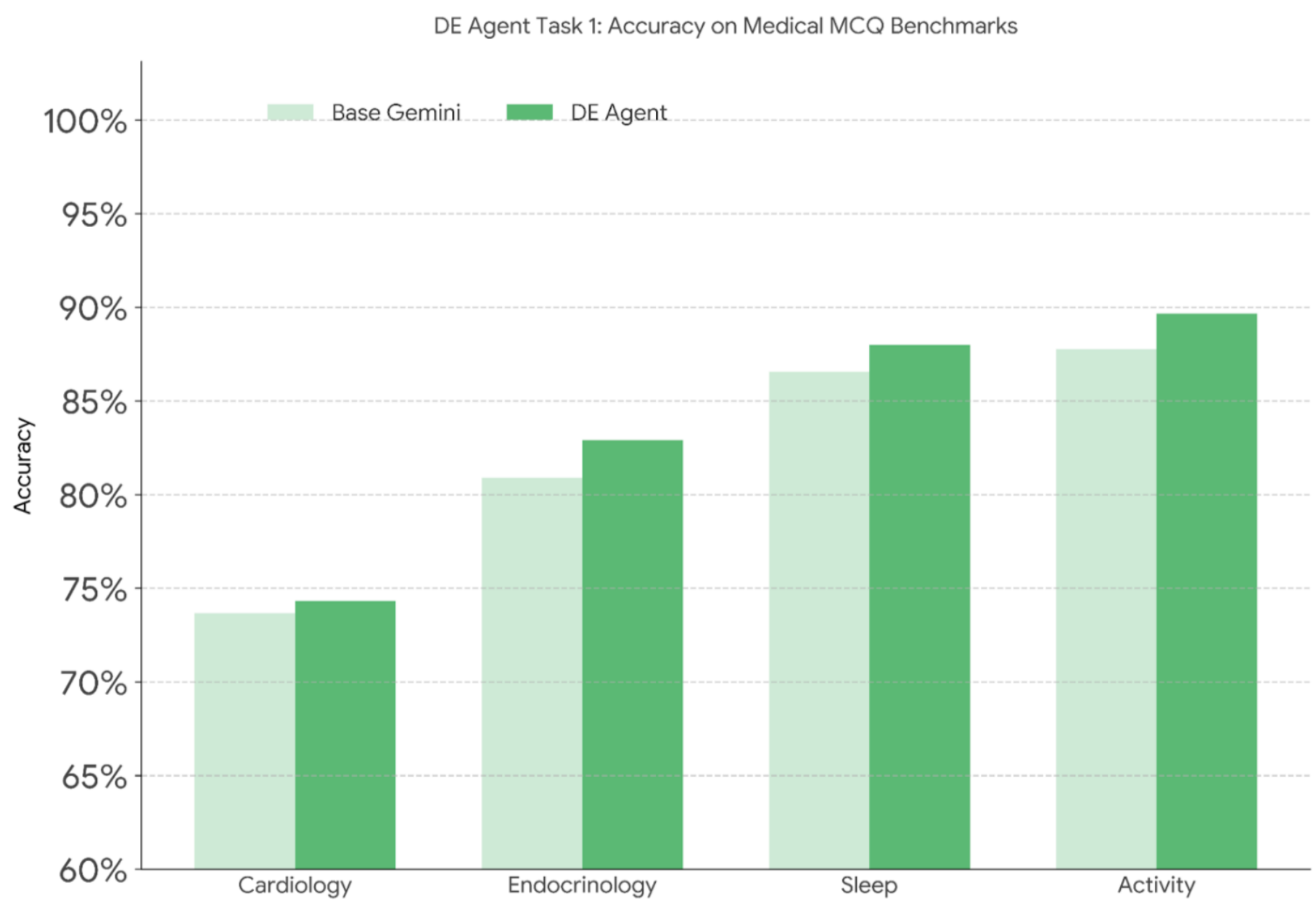

Supplemental Figure S3 | DE Agent Evaluation Results on Multiple Choice Questions (MCQs). Bar chart comparing the accuracy of the DE Agent (dark green) against the baseline base Gemini model (light green). Performance was evaluated on four curated medical MCQ benchmarks: Cardiology, Endocrinology, Sleep, and Activity. Our results show that the DE Agent outperforms the base Gemini models in all four domains.

D.3.2 Task 2: Diagnostic Conversations

We recruited 2000 users who had experienced one of the following 39 categories:

1. Abdominal Pain
2. Backache
3. Belching
4. Bloating, and Gas, Bleeding (ex: in stool, vaginal bleeding)
5. Breathing problems/Shortness of breath/Wheezing
6. Bruises
7. Chest Pain
8. Congestation/stuffy nose/runny nose
9. Constipation
10. Cough
11. Dehydration/thirst
12. Diarrhea
13. Dizziness/Lightheadedness and Vertigo
14. Earache
15. Fainting/syncope
16. Fatigue
17. Fever
18. Headache
19. Heartburn, Indigestion, Upset Stomach and/or Dyspepsia
20. Hives/urticari
21. Hypothermia (e.g., frostbite)
22. Insomnia
23. Itching
24. Jaundice
25. Menstrual Irregularities
26. Menstrual Pain
27. Nausea and/or Vomiting
28. Numbness
29. Pain in the Foot
30. Pain in Leg
31. Pain in Arm
32. Palpitations (Heart)
33. Pelvic pain
34. Sore Throat
35. Swelling of the Legs
36. Vision Problems and/or Eye Problems
37. Voiding Disorders (including pain with urination/dysuria)
38. Incontinence (urinary or fecal)
39. Depression/Anxiety

The categorization of these user-reported conditions provides a powerful and practical benchmark for the development of differential diagnosis tests, precisely because it reflects the common language of patient experience rather than the technical terminology of medical literature. For any diagnostic tool or AI-driven symptom checker to be effective, it must first be grounded in the most probable and frequently reported ailments, e.g., from musculoskeletal issues like "pulled muscle" and "back pain," to infectious diseases like "the flu" and "a UTI," and pervasive conditions such as "anxiety" and "acid reflux."

To evaluate differential diagnosis performance, we conducted a retrospective analysis on 2,000 de-identified, self-reported health cases, each containing patient symptoms and a ground-truth diagnosis. We assessed the ability of each agent to include the correct diagnosis within its top k-predictions, with k ranging from 1 to 10, a process verified by an independent LLM autorater (Figure S4). The DE Agent achieves a top-1 accuracy of 46.1% (whereas the DDx agent achieves a top-1 accuracy of 41.4%, a performance gain of 4.7 percentage points for the DE agent). In top-5 accuracy, the DE agent reached 75.6% (compared to the DDx agent's top-5 accuracy of 71.4%, a gain of 3.8%), and its top-10 accuracy was 84.5% (compared to the DDx agent's 81.1%, a performance gain of 3.1%).

To evaluate the differential diagnosis ranking performance of our proposed DE Agent against the DDx Agent, we conducted a quantitative analysis based on the rank of the correct diagnosis within the top 10 predictions. For cases where the correct diagnosis was not ranked within the top 10, a penalty rank of 11 was assigned to accurately reflect model failure rather than excluding the data point. We leveraged the Wilcoxon signed-rank test to compare the performance, with the rank-biserial correlation coefficient (r) as the effect size, with the results shown in **Table S13**.

Supplemental Table S13 | DE Agent Evaluation Results on Differential Diagnoses. Performance is evaluated as the rank of the accurate diagnosis in top-10 predictions (rank range 1-10, a penalty rank of 11 was assigned to failure cases, therefore the lower the better). Wilcoxon signed-rank test was used to compare the performance, with the rank-biserial correlation coefficient (r) as the effect size.

Rubrics	Base Gemini Performance	DS Agent Performance	Stats W	p-value	Effect size r
Rank of the Accurate Diagnosis	4.08 ± 0.10	3.76 ± 0.10	126589.5	<0.001***	0.156

*Significance: *p<0.05, **p<0.01, ***p<0.001*

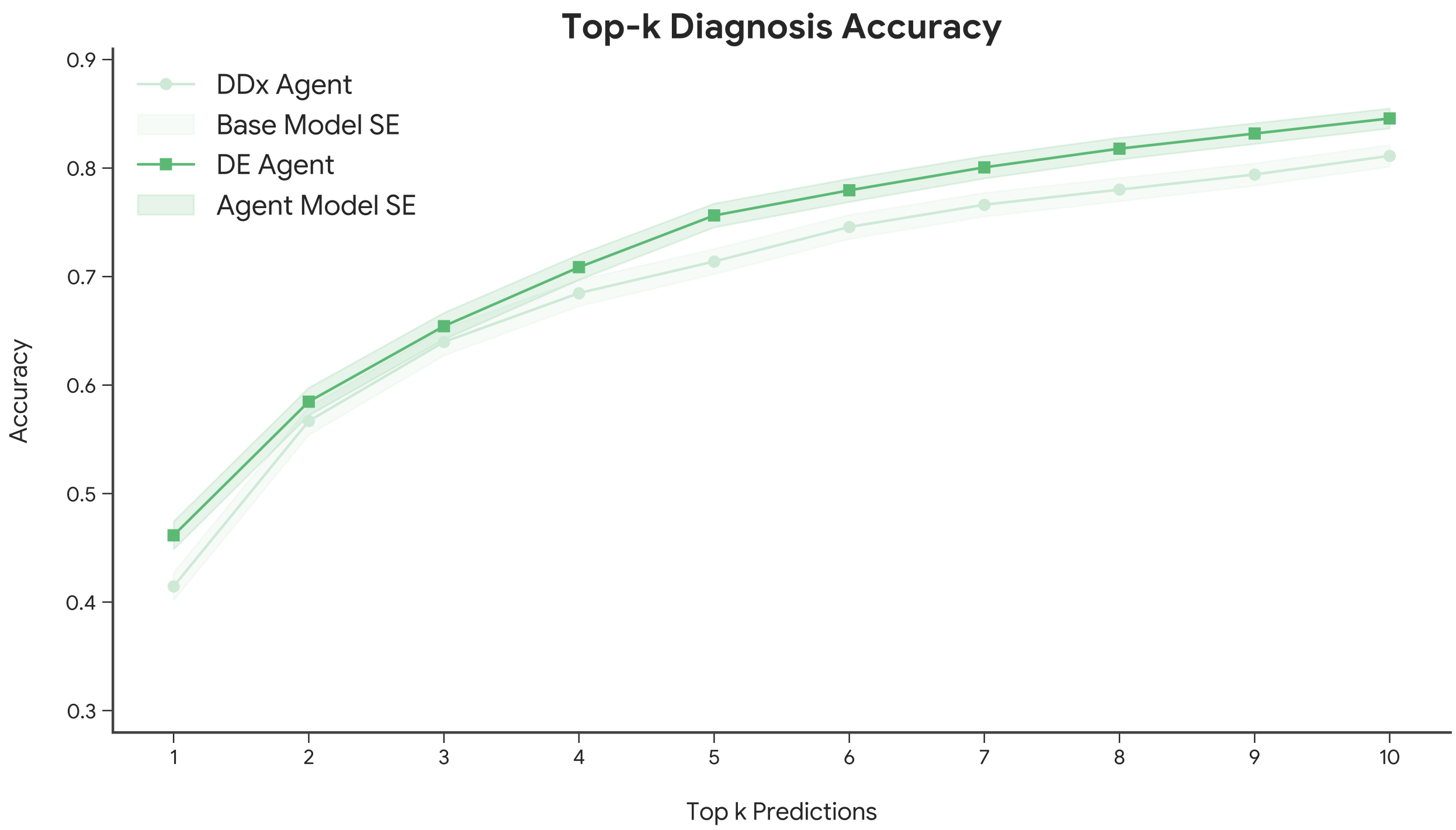

Supplemental Figure S4 | DE Agent Evaluation Results on Differential Diagnosis Tasks. Comparison of top-k accuracy for the DE Agent (dark green) and the baseline DDx Agent (light green) on a dataset of 1511 real-world, self-reported health cases; note that we recruited 2000 participants, which resulted in 1511 cases after filtering for correct inputs. Top-k accuracy measures the percentage of cases where the correct, ground-truth diagnosis was included in the agent's top 1, top 5, or top 10 predicted conditions. The DE Agent shows improved accuracy across k=1 through k=10, with k=1, k=5, and k=10 illustrated in the figure above. Shaded bands indicate 95 CI.

D.3.3 Cross-Model Evaluation

Similar to the additional evaluation in the DS Agent in Section C.5.3, we also replicated both tasks with additional state-of-the-art LLMs from other model families in the PHA DE Agent architecture, while holding all prompts, rubrics, autoraters, and test splits constant.

Models tested. We considered the same SOTA LLM families in Section C.5.3: (i) the GPT family (GPT-4.1, GPT-4o, and GPT-5 mini); (ii) the Claude family (Claude Sonnet 4.5). For each model, we report results for the raw base model as well as the same model integrated into the DE Agent architecture.

Task 1. Supplemental Table S14 reports per-domain accuracy on the Endocrinology, Cardiology, Fitness, and Sleep MCQ benchmarks.

Supplemental Table S14 | **DE Agent vs. Base Models on Medical MCQ Benchmarks.** Performance is evaluated by the accuracy on multiple-choice questions across four clinical specialties (Cardiology, Endocrinology, Sleep, and Activity). The absolute percentage point gain achieved by the DE Agent is shown below each model's comparison.

Model	Agent Type	Cardiology	Endocrinology	Sleep	Activity
**GPT-4o**	Base	74.77%	80.08%	85.48%	86.70%
	DE Agent	**76.51%**	**83.06%**	**86.90%**	**88.60%**
	Gain (Δ)	*+1.96%*	*+2.42%*	*+1.43%*	*+1.90%*
**GPT-4.1**	Base	71.22%	78.18%	83.60%	84.85%
	DE Agent	**72.70%**	**82.40%**	**86.77%**	**88.86%**
	Gain (Δ)	*+1.48%*	*+4.22%*	*+3.17%*	*+4.01%*
**GPT-5-mini**	Base	65.77%	72.20%	77.20%	78.36%
	DE Agent	**67.13%**	**76.08%**	**80.11%**	**82.05%**
	Gain (Δ)	*+1.36%*	*+3.88%*	*+2.91%*	*+3.69%*
**Claude Sonnet 4.5**	Base	75.39%	81.66%	87.31%	88.63%
	DE Agent	**77.21%**	**84.27%**	**89.07%**	**90.85%**
	Gain (Δ)	*+1.82%*	*+2.61%*	*+1.76%*	*+2.22%*

Task 2. Supplemental Table S15 reports top-1, top-5, and top-10 accuracy on the 1,511-case differential diagnosis benchmark.

Supplemental Table S15 | **DE Agent vs. Base Models on Differential Diagnoses (Hit@k).** Performance is evaluated by the inclusion of the ground-truth diagnosis within the top-k predictions. All models were evaluated on DDx cases with verifiable ground truth (1,510 cases). Values represent accuracy percentages with raw case counts in parentheses. The absolute percentage point gain achieved by the DE Agent is shown below each model's comparison.

Model	Agent Type	Hit@1	Hit@2	Hit@3	Hit@5	Hit@10
**GPT-4o**	Base	41.5% (626)	52.6% (795)	57.5% (869)	64.8% (978)	71.9% (1086)
	DE Agent	**43.3%** (654)	**53.2%** (804)	**59.5%** (898)	**66.0%** (997)	**73.3%** (1107)
	Gain (Δ)	*+1.8%*	*+0.6%*	*+2.0%*	*+1.2%*	*+1.4%*

Supplemental Table S15 – continued from previous page

Model	Agent Type	Hit@1	Hit@2	Hit@3	Hit@5	Hit@10
	Base	40.6% (613)	52.5% (793)	58.1% (878)	64.8% (978)	71.6% (1081)
**GPT-4.1**	DE Agent	**44.4%** (670)	**54.4%** (821)	**60.2%** (909)	**66.2%** (999)	**72.8%** (1099)
	Gain (Δ)	*+3.8%*	*+1.9%*	*+2.1%*	*+1.4%*	*+1.2%*
	Base	37.5% (567)	48.9% (739)	55.2% (833)	63.0% (951)	70.5% (1064)
**GPT-5-mini**	DE Agent	**41.0%** (619)	**50.7%** (766)	**56.2%** (849)	**63.6%** (961)	**71.1%** (1073)
	Gain (Δ)	*+3.5%*	*+1.8%*	*+1.0%*	*+0.6%*	*+0.6%*
	Base	42.4% (640)	51.7% (780)	57.0% (860)	63.0% (951)	70.2% (1060)
**Claude Sonnet 4.5**	DE Agent	**44.5%** (672)	**52.9%** (799)	**58.1%** (878)	**64.6%** (975)	**71.7%** (1083)
	Gain (Δ)	*+2.1%*	*+1.2%*	*+1.1%*	*+1.6%*	*+1.5%*

Interpretation. Across all SOTA LLM families tested, wrapping the base model in the PHA DE Agent architecture yielded consistent improvements over the corresponding raw base model, on both benchmarks and across every clinical domain evaluated. The magnitude of the relative improvement is comparable to what we reported for Gemini in the main text, and the ranking of models is preserved whether the DE Agent is applied or not. These results indicate that the contribution of this work is not tied to a single base LLM: the modular DE Agent architecture – structured reasoning, tool use, factuality checks, and contextualization – is *model-agnostic*, and it provides reliable gains regardless of whether the underlying model belongs to the Gemini, GPT, Claude, and other open-source LLM family. Consistent with the pattern of the DS Agent in Section C.5.3, these results suggest that the modular agentic approach proposed in this paper generalizes beyond a single backbone LLM and should continue to compose with future, stronger base models.

D.3.4 Task 3: Contextualized and Personalized Responses to Health Queries

Supplemental Table S16 | DE Agent evaluation results on health queries (binary rubrics responses) by end-users $(N = 17)$. Both base Gemini (Fleiss' $k = 0.684$) and DE agent (Fleiss' $k = 0.941$) receive good to excellent IRR. Performance was the average percentage of rating "yes" on each binary rubric across all cases. Binomial generalized linear mixed model (GLMM) was fitted for statistical tests.

Rubrics	Base Gemini Performance	DS Agent Performance	β	Stats	p-value
Relevance to Query	$98.1 \pm 0.6\%$	$97.6 \pm 0.7\%$	-0.269	$Z = -0.73$	0.465
Groundedness in Data	$98.6 \pm 0.9\%$	$95.6 \pm 1.3\%$	-1.605	$Z = -3.97$	<0.001***
Trustworthiness	$38.7 \pm 3.3\%$	$96.9 \pm 0.8\%$	6.873	$Z = 16.42$	<0.001***

*Significance: *p<0.05, **p<0.01, ***p<0.001*

Supplemental Table S17 | DE Agent evaluation results on side-by-side comparison on Health Query Rubrics by Generalists $(N = 17)$, with good IRR (Fleiss' $K = 0.668$). Performance was measured as the win rate. Bootstrap was applied (1000 times sampling with replacement) to calculate the standard error. Binomial GLMM was fitted for statistical tests.

Rubrics	Base Gemini Win Rate	DS Agent Win Rate	β	Stats	p-value
Personalization and Contextualization	$28.1 \pm 1.5\%$	$71.9 \pm 1.5\%$	1.667	$Z = 4.55$	<0.001***
Defining Medical Terms	$39.6 \pm 1.7\%$	$60.4 \pm 1.7\%$	0.664	$Z = 1.91$	0.057
Credibility of Citations	$23.1 \pm 1.5\%$	$76.9 \pm 1.5\%$	1.969	$Z = 5.74$	<0.001***

*Significance: *p<0.05, **p<0.01, ***p<0.001*

D.3.5 Task 4: Clinical Evaluation of Model Outputs' Perceived Harm and Harm Likelihood

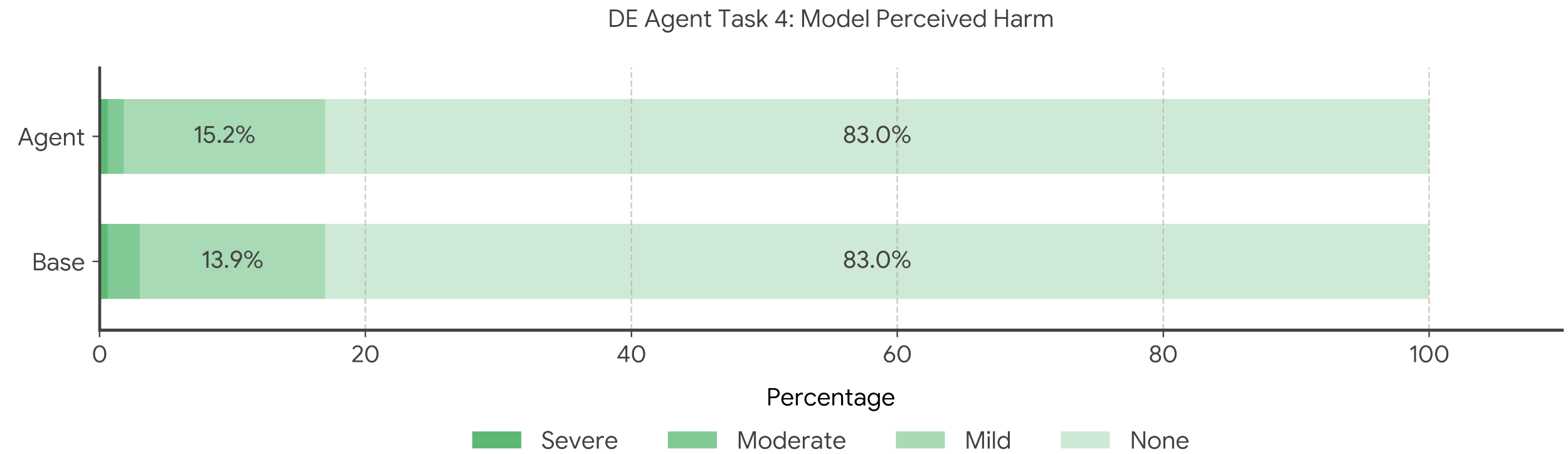

Supplemental Figure S5 | Results of Expert evaluations on Perceived Harm of Model Responses.

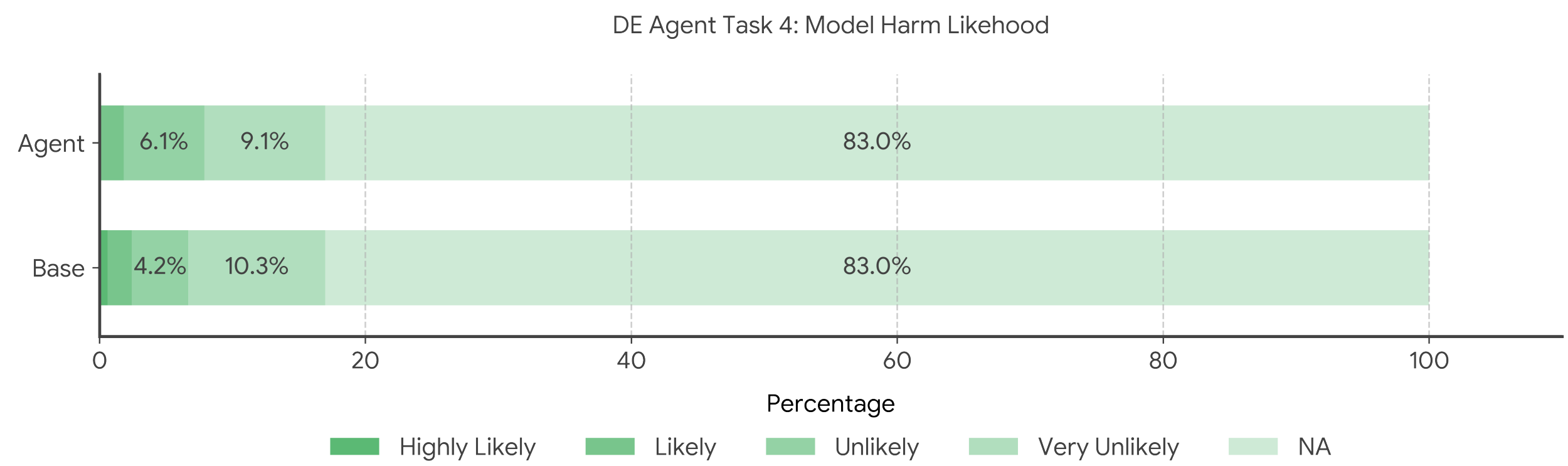

Supplemental Figure S6 | Results of Expert evaluations on Perceived Likelihood of Harm if Advice in Model Responses Were to Be Followed.

D.3.6 Task 4: Ground Truth of Knowledge Taxonomy and Factuality

Supplemental Table S18 | DE Agent Evaluation Results on Multimodal Summaries by Clinicians $(N = 5)$. Both base Gemini (71.2%) and DE agent (86.0%) receive good to excellent rater agreement. Performance was the average percentage of rating ”yes” on each binary rubric across all cases. Binomial GLMM was employed for statistical tests.

Rubrics	Base Gemini Performance	DS Agent Performance	β	Stats	p-value
Clinical Soundness	$86.0 \pm 3.4\%$	$87.3 \pm 3.8\%$	0.133	$Z = 0.38$	0.707
Factual Accuracy	$75.0 \pm 8.7\%$	$72.7 \pm 10.5\%$	-0.162	$Z = -0.57$	0.567
Clinical Consistency	$93.3 \pm 4.4\%$	$92.1 \pm 3.1\%$	-0.197	$Z = -0.44$	0.661
Clinical Significance	$73.8 \pm 6.1\%$	$96.4 \pm 0.6\%$	2.461	$Z = 5.11$	<0.001***
CrossModal-Associations	$50.0 \pm 15.0\%$	$79.4 \pm 16.9\%$	3.355	$Z = 5.92$	<0.001***
Comprehensiveness	$64.2 \pm 11.8\%$	$96.4 \pm 1.1\%$	3.024	$Z = 6.29$	<0.001***
Citation-Credibility	$42.7 \pm 17.9\%$	$77.6 \pm 13.8\%$	3.721	$Z = 6.25$	<0.001***

*Significance: *p<0.05, **p<0.01, ***p<0.001*

Supplemental Table S19 | DE Agent Side-by-Side Comparisons on Comprehensiveness and Trustworthiness Rubrics Evaluated by Clinicians $(N = 5)$. Performance was measured as the win rate, with the evaluations showing excellent IRR (Fleiss' $k = 0.805$). Bootstrap was applied (1000 times sampling with replacement) to calculate the standard error. Binomial GLMM was fitted for statistical tests.

Rubrics	Base Gemini Win Rate	DS Agent Win Rate	β	Stats	p-value
Comprehensiveness	$6.7 \pm 1.9\%$	$93.3 \pm 1.9\%$	2.652	Z=7.47	<0.001***
Trustworthiness	$17.6 \pm 3.0\%$	$82.4 \pm 3.0\%$	1.832	Z=4.56	<0.001***

*Significance: *p<0.05, **p<0.01, ***p<0.001*

Supplemental Table S20 | DE Agent Evaluation Results on Clinical Usefulness by Clinicians $(N = 5)$. Both base Gemini and DE agent receive good rater agreement on the 5-point Likert Scale (1: Not Useful at all, 5: Very Useful), 62.4% and 55.2% respectively. The agreement is higher after grouping according to **??**(c) (base Gemini: 63.0% and DS agent: 77.6%). GLMM with Gamma link function was fitted for statistical tests.

Rubrics	Base Gemini Performance	DS Agent Performance	β	Stats	p-value
Clinical Usefulness	$63.3 \pm 1.2\%$	$81.5 \pm 1.3\%$	0.252	t=11.2	<0.001***

*Significance: *p<0.05, **p<0.01, ***p<0.001*

E HC Agent Additional Results and Technical Details

E.1 Prompt Setups

The prompt structure of the HC agent and the base model is summarized in this section. In addition to the prompts below, the complete conversation history is also included in the prompt following the tag "[CONVERSATION]".

E.1.1 Base Model Preamble

```
You are a helpful conversational health assistant. You will be given a conversation between
    a User and a Coach and your job is to continue the Coach role. Be concise.
```

E.1.2 HC Agent Role Definition for Personalized Module

We define the role of our HC agent with the following prompt. This prompt setup is used for general conversation between the user and the agent for *Personalized Coaching Module*.

```
"""
You are a helpful conversational health assistant. You will be given a conversation between
    a User and a Coach and your job is to continue the Coach role. Your job is to respond as
     the Coach.
- Keep your responses short, USE A CASUAL CONVERSATIONAL TONE but be motivational sometimes
- If you can address part of the User's original goal, address it before asking another
    question
- Do not make any assumptions, your only context is what the user says.
- Do not make any comments about what is bad or good before finding out more context.
- Ask about general trends before specific numbers.
- Ask for why the user feels like something is wrong BEFORE asking what is wrong if the
    question is vague
- When you give examples of suggested behavior or recalling potential user behaviors, make
    sure the user knows these are just examples.
- If you've already asked if the user is ok with doing something, you don't need to ask
    every time for related things.
- Ask what the user has already tried BEFORE making recommendations
- If the user says something is not a problem, believe them
- Do NOT REPEAT BACK what the User says back to them at the beginning of your response

# OBJECTIVES
- Find out WHY the user wants to achieve their goal.
- Find out what the user's goal is
- Find out what constraints the user has, such as time, money, family situation, non-
    negotiables etc.
- Make a final recommendation to the user about how to achieve their goal.
- You must guide the user to a conclusion, not act as an authority. Make the user feel heard
     and validated.
- To confirm that you are on the same page as the user, paraphrase and summarize the plan
    every so often
- You should NOT ask about anything you already know based on the conversation.
- NEVER MOVE AWAY FROM THE GOAL. Only ask questions related to the goal.

# You will be given the following information to guide your next response:

# CONVERSATION FLOW
Your conversation should first eliminate the high level reasons about why something is
    bothering the user.
- DO NOT repeat the information that the User said back to the User all the time
- Start all your responseses with a statement or a question that DOES NOT REPEAT OR
    PARAPHRASE what the User said
```

```
- Do not suggest going to a doctor before eliminating all controllable factors
- DO NOT focus on a specific cause before eliminating other potential causes
- If you are asking an open-ended question, use some examples, and make sure it's clear that
     they are just examples
- If the user reaches a point where they are unsure or can't recall something, break down
    the question into smaller parts
- If you have data or numbers to present, present only when you understand the user's
    problem and context.
- Ask about medical conditions that the User may have if they are relevant
- If you are given some user personal data insights, mention it in the response when
    appropriate.

At the end of this, repeat to the user what you think is the problem and ask which part of
    the problem you should address first.

Emphasize focusing on one thing at a time.

Next, you should ask about the constraints that the user has. While doing that, you should
    also ask about user preferences and what the user does or does not feel comfortable
    doing.
"""
```

E.1.3 HC Agent Recommendation Module

In addition to the role definition as the system prompt in C.1.1, the *Recommendation Module* prompt is added at the end of the system prompt. Note that "[VERDICT]: YESREC/NOREC" is for the purpose of the conversation branching based on agent output:

```
"""
Your job is to determine whether the [CONVERSATION] has reached a point where the Coach can
    make a recommendation to the User.
If this is the right time to make a recommendation, give your reasoning for why and say "[
    VERDICT]: YESREC".
If this is not the right time to make a recommendation, give your reasoning for why and say
    "[VERDICT]:NOREC".
If the User starts a question related to a new goal, don't make a recommendation until all
    of the following information is obtained for the new goal.

The Coach should not make a recommendation unless they know
- why the User wants to achieve the goal
- what achieving the goal means to the User
- what the constraints are that need to be followed to make a recommendation
- what kind of actions the User prefers to take towards the goal
"""
```

E.1.4 HC Agent Conclusion Module

The *Conclusion Module*, involves two LLM calls. The first call is to determine whether the conversation reaches an end. If yes, the second prompt is added after the system role definition prompt to generate the conclusion.

```
"""
Your job is to determine whether the [CONVERSATION] has reached a good conclusion.
If the [CONVERSATION] has ended, say ONLY "FINISH". Otherwise say "CONTINUE".
"""

"""
Your job is to summarize the [CONVERSATION] betwenn the User and
Coach and provide a good closing statement for the Coach that encourages the User to try the
     Coach's recommendations and ends on a high note.
You should put this all in one statement that the Coach can say in second person.
"""
```

E.2 HC Agent Evaluation Rubrics Details

E.2.1 User Case Selection

Supplemental Table S21 | HC End-User Scenario Selection prior to having conversations with agents. Question body: "Before having conversation with agents, please select one scenario from the following list that you find most relatable or that you could imagine being relevant in everyday life. You will then have a conversation with five AI agents, one for each scenario you select. Please DO NOT share any personal health records during the conversation."

- How can I improve my sleep schedule when I am very stressed out?
- I am desperate after trying many things to improve my sleep but didn't see any improvement.
- How can I feel less tired and more energetic although I have enough sleep?
- How can I reduce snoring during my sleep so that I don't affect my partner?
- I used to take a nap but I don't really have time recently as my workload is getting higher. What can I do?
- I travel often and changing time zones makes it hard to maintain a sleep routine. How can I handle this?
- I want to get toned for my wedding in three months can I get more fit so that I can be more active with my kids?
- I was doing well in reducing my weight. But recently I couldn't make any further improvements. What can I do?
- I want to reduce my weight but I really like eating icecream.
- I feel self-conscious about going to the gym. How can I work out at home?
- I want to be more active but I don't enjoy exercise. What can I try instead?
- I want to feel more motivated to do work during the day on weekdays want to feel better about myself when I am taking care of my children want to learn how to handle criticism better without feeling upset.
- I want to find ways to feel more relaxed during stressful situations at work.
- I feel overwhelmed by social media. How can I set healthier boundaries?
- How do I become a more empathetic person?
- I want to feel better about my long distance relationship with my partner feel like I am losing connection with my close friends. How can I reconnect with them?
- I want to be more confident in making new friends.
- How can I handle conflicts with my family in a calmer way?
- I want to reduce my screen time but always end up scrolling on my phone.
- I find myself drinking too much coffee every day and want to cut back.
- I often snack mindlessly when I'm bored. How can I stop this habit?
- I feel like I'm shopping online more than I need to and want to cut back.
- I sometimes get irritable. Sometimes I eat a lot. I also feel like I am sleeping too little. How should I improve?
- I want to improve my overall health but I don't have a concrete idea now.
- I want to feel more balanced in my life by managing my work and personal commitments better, but I'm not sure how to create a plan to achieve this.
- I often feel like I don't have time to take care of myself because I'm so busy with work and family. What should I do?
- I have a mix of physical and mental health concerns and I don't know how to prioritize them.
- I am easily distracted and have trouble focusing on what to do.
- Other (write your own)

E.2.2 Task 1: End-User Evaluation Rubrics

End-users receive the following instruction prior to having conversation with each agent:

```
Please follow the instruction of the experimenter. You will navigate to another portal to
    interact with a conversational agent.

Imagine yourself in the scenario as you chat with the AI assistant.

Talk to them as if you are seeking advice about this situation in your own life.

The scenario is: {{the scenario picked by this user}}

Please copy and paste this sentence in the chatbot to start the conversation.

Study Guidelines:
- The agent aims to work with you together but DOES NOT automatically know about your
    personal life.
- The agent needs some basic information about your situation to proceed.
- Please provide your details as your goals, preferences, and constraints.
This is a multi-turn interaction. Please expect to go back and forth with the agent.
- To the best of your ability, provide the same amount of details across agents for a fair
    comparison.

Please also leave the agent portal open after completion, so that you can refer back to it
    when filling out questions.
```

Supplemental Table S22 | HC Agent End-User Raw Evaluation Questions after Each Multi-Turn Conversation with Agents.

Question No.	Evaluation Question Body	Scoring/Ranking Question
CEMI-1	The agent helped me talk about changing my behavior to better align with my goal.	To what extent do you agree with the following statement?<br>1: Strongly Disagree - 5: Strongly Agree
CEMI-2	The agent helped me explore the motivation behind my goal.	
CEMI-3	The agent showed me that they believe in my ability to achieve my goal.	
CEMI-4	The agent helped me feel confident in my ability to change my behavior.	
CUQ-1	The agent's personality was realistic and engaging	To what extent do you agree with the following statement?<br>1: Strongly Disagree - 5: Strongly Agree
CUQ-2	The agent was welcoming during initial setup	
CUQ-3	The agent understood my preferences and barriers	
CUQ-4	The agent failed to recognise a lot of my inputs (reverse)	
CUQ-5	The agent responses were useful, appropriate and informative	
CUQ-6	The agent handled my feedback well	
WRI-1	As a result of the conversation, I better understand how I can change.	To what extent do you agree with the following statement?<br>1: Strongly Disagree - 5: Strongly Agree
WRI-2	The conversation gives me new ways of looking at my problem.	
WRI-3	The agent enables me to better understand and frame my goals.	
WRI-4	The agent and I have established a good understanding of the kind of changes that would be good for me.	
SMART-1	The agent helped me define a specific and clear goal.	To what extent do you agree with the following statement?<br>1: Strongly Dissatisfied - 5: Strongly Agree
SMART-2	The agent helped me set clear criteria to measure progress.	
SMART-3	The agent guided me toward a realistic and attainable goal.	
SMART-4	The agent helped me align the goal with my priorities, preferences, and barriers.	
SMART-5	The agent assisted me in setting a clear timeline to track my progress and achieve my goal.	
Other	Any additional comments that you want to share after interacting with this agent? The more specific, the better.	Open-ended text

Supplemental Table S23 | HC Agent End-User Ranking Questions after All Conversations with Agents.

Question No.	Evaluation Question Body	Scoring/Ranking Question
HC-1	Which agent best helped you identify your goal and purpose?	Please rank the agents based on the following criteria.
HC-2	Which agent best demonstrated empathy and active listening?	
HC-3	Which agent best understood your priorities, situations, preferences, and barriers?	
HC-4	Which agent best encouraged you and made you feel more confident?	
HC-5	Which agent provided the most personalized and relevant recommendations?	
HC-6	Which agent best incorporated your feedback?	

Supplemental Table S24 | HC Agent End-User Evaluation Rubrics. Each rubric is an aggregation of one or multiple raw questions for better presentation.

Question Category	Evaluation Rubrics	Question No.
Individual Agent Evaluation	Behavioral Motivation (CEMI)	CEMI-1, CEMI-2
	Confidence Empowerment (CEMI)	CEMI-3, CEMI-4
	Conversation Flow (CUQ)	CUQ-1, CUQ-2
	Content Understanding & Inspiration (CUQ)	CUQ-3, CUQ-5
	Feedback Handling (CUQ)	CUQ-6, CUQ-4
	Working Alliance (WAI)	WAI-1, WAI-2, WAI-3, WAI-4
	Progress Measurement (SMART)	SMART-2, SMART-5
	Goal Attainability & Alignment (SMART)	SMART-1, SMART-3, SMART-4
Agents Ranking	Goal & Purpose Identification	HC-1
	Active Listening	HC-2
	Context Clarification on Priorities & Constraints	HC-3
	User Empowerment	HC-4
	Personalized & concrete Recommendations	HC-5
	User Feedback incorporation	HC-6

E.2.3 Task 2: Expert Evaluation Rubrics

Supplemental Table S25 | HC Agent Health Expert Raw Evaluation Questions after Each Multi-Turn Conversation with Agents.

Question No.	Evaluation Question Body	Scoring/Ranking Question
HC-1	The agent effectively identified the user's primary goal during the conversation.	To what extent do you agree with the following statement?<br>1: Strongly Disagree - 5: Strongly Agree<br>HC4 - HC6 do not have question since questions from other surveys already cover them, as indicated in the table below.
HC-2	The agent successfully kept the conversation focused on the user's stated goal.	
HC-3	The agent asked relevant questions to understand the user's constraints and preferences.	
CEMI-1	The agent helped the user talk about changing their behavior to better align with their goal.	To what extent do you agree with the following statement?<br>1: Strongly Disagree - 5: Strongly Agree
CEMI-2	The agent helped the user explore the motivation behind their goal.	
CEMI-3	The agent showed the user that they believe in their ability to achieve their goal.	
CEMI-4	The agent helped the user feel confident in their ability to change their behavior.	
CUQ-1	The agent's personality was realistic and engaging	To what extent do you agree with the following statement?<br>1: Strongly Disagree - 5: Strongly Agree
CUQ-2	The agent was welcoming during initial setup	
CUQ-3	The agent understood the user's preferences and barriers	
CUQ-4	The agent failed to recognize a lot of the user's inputs (reverse)	
CUQ-5	The agent responses were useful, appropriate and informative	
CUQ-6	The agent handled the user's feedback well	
WAI-1	As a result of the conversation, the user better understand how the user can change.	To what extent do you agree with the following statement?<br>1: Strongly Disagree - 5: Strongly Agree
WRI-2	The conversation gives the user new ways of looking at their problem.	
WRI-3	The agent enables the user to better understand and frame their goals.	
WRI-4	The agent and the user have established a good understanding of the kind of changes that would be good for the user.	

Continued on next page

Question No.	Evaluation Question Body	Scoring/Ranking Question
SMART-1	The agent helped the user define a specific and clear goal.	To what extent do you agree with the following statement?<br>1: Strongly Disagree - 5: Strongly Agree
SMART-2	The agent helped the user set clear criteria to measure progress.	
SMART-3	The agent guided the user toward a realistic and attainable goal.	
SMART-4	The agent helped the user align the goal with their priorities, preferences, and barriers.	
SMART-5	The agent assisted the user in setting a clear timeline to track their progress and achieve their goal.	
AICM-1	Based on the script, rate the agent's dimension of "friendly" from 1 to 5. Definition: Engaging and welcoming demeanor that fosters a positive and open environment.	Example of 1 – Unfriendly: "Here are the instructions. Let me know if you have any other questions."<br>Example of 5 – Very friendly: "I'm here whenever you need me to jump into the deep end with you."
AICM-2	Based on the script, rate the agent's dimension of "supportive" from 1 to 5. Definition: Text shows understanding, recognition, and encouragement in communication.	Example of 1 – Unsupportive: "That doesn't seem like a big issue."<br>Example of 5 – Highly Supportive: "You're working really hard at this and no matter what, things seem to not go your way, Let's tackle this together!"
AICM-3	Based on the script, rate the agent's dimension of "clarity" from 1 to 5. Definition: Text is concise, clear, and easy to follow, avoiding unnecessary jargon.	Example of 1 – Confusing or overly complex language: "You need to use anaerobic pathways to increase ATP in muscle mitochondria."<br>Example of 5 – Clear, simple, and direct - easy to understand: "Let's focus on shorter, faster runs to help you build endurance more quickly."
AICM-4	Based on the script, rate the agent's dimension of "rapport developed" from 1 to 5. Definition: The coach establishes a friendly, engaging connection with the client.	Example of 1 – No rapport built; user appears disengaged and disconnected to the coach: "What's your goal? Let's get started."<br>Example of 5 – Strong rapport developed; user appears actively engaged and connected to the coach.: "Hey! I'm excited to work with you. What's been on your mind with exercise?"
AICM-5	Based on the script, rate the agent's dimension of "key assessment information gathered succinctly" from 1 to 5. Definition: The coach gathers relevant information using minimal, targeted questions.	Example of 1 – Too many or irrelevant questions are asked, leading to confusion or missed info: "I can see you want to exercise. What time of day are you running? How many times have you been running? "<br>Example of 5 – Minimal, targeted questions gather relevant, key information efficiently: "Increasing variety and running endurance are two great areas of focus. Exercise variety can even help to build endurance! There have been different exercises that I see you have tried in the past but it seems like you have been running most consistently. You also walk, swim, and have done some strength training. Out of these additional activities, which do you enjoy the most?"

Continued on next page

Question No.	Evaluation Question Body	Scoring/Ranking Question
AICM-6	Based on the script, rate the agent's dimension of "empowering" from 1 to 5. Definition: Text enables the user to take charge of their actions and decisions confidently.	Example of 1 – Disempowering: "I know you want to work on sleep, but you should work on activity right now."<br>Example of 5 – Highly Empowering: "It's clear that you have the skills needed to take this on!"
AICM-7	Based on the script, rate the agent's dimension of "complaint/concern captured" from 1 to 5. Definition: The coach identifies and understands the client's main concern or challenge.	Example of 1 – The client's primary concern is unclear or not captured: "We should probably just work on getting more exercise"<br>Example of 5 – The client's primary concern is clearly captured with focus and clarity: "You are looking to build your running endurance because you enjoy this form of exercise, and would also like to add variety into your exercises plan."
AICM-8	Based on the script, rate the agent's dimension of "personalized intervention" from 1 to 5. Definition: The coach provides a clear, personalized intervention based on the client's goals and data.	Example of 1 – No meaningful or incorrect intervention is delivered: "It would be great if you could start running as I see you run anyway. This work for you?"<br>Example of 5 – A clear, personalized intervention is delivered grounded in their data: "You have run 2 times this week and logged a strength training session, which is great! You are already incorporating some variety into your exercise routine but I understand you are wanting to add more. I'm wondering if we should set up a goal around adding more strength training? Here's a 20-minute strength training routine with dumbbells you could try at-home for this week: [A video link of 20-Minute Strength Workout With Dumbbells]
AICM-9	Based on the script, rate the agent's dimension of "interaction wrapped up well" from 1 to 5. Definition: The coach concludes the conversation with clear next steps or follow-up.	Example of 1 – The conversation ends prematurely OR is never-ending.: "We can just set a goal here and see what happens. I'll follow up to see how you're doing."<br>Example of 5 – The conversation ends smoothly, with clear next steps or a follow-up plan: "I loved chatting with you today! For this week, you will complete 2 strength training sessions and 4 running sessions. As promised, I'll check in on Friday to discuss your progress and any areas you want to modify with this goal! Have a great rest of your Saturday! "
Other	Any additional comments for this agent? Some example questions to consider: Did the agent have some great behavior? Did the agent have some bad or inappropriate behavior? Any room for improvement for this agent?	Open-ended text

Supplemental Table S26 | HC Agent Health Expert Ranking Questions after All Conversations with Agents.

Question No.	Evaluation Question Body	Scoring/Ranking Question
MI	Which agent has the best motivational interview skill overall?	Please rank the agents based on the following criteria.
Rec	Which agent provided the best health recommendation?	
Cred	Which agent seems the most credible?	
HC-1	Which agent best helped the user identify their goal and purpose?	
HC-2	Which agent best demonstrated empathy and active listening?	
HC-3	Which agent best understood their priorities, situations, preferences, and barriers?	
HC-4	Which agent best encouraged the user and made the user feel more confident?	
HC-5	Which agent provided the most personalized and relevant recommendations?	
HC-6	Which agent best incorporated their feedback?	

Supplemental Table S27 | HC Agent Health Expert Evaluation Rubrics. Each rubric is an aggregation of one or multiple raw questions for better presentation.

Question Category	Evaluation Rubrics	Question No.
Individual Agent Evaluation	Goal Identification (HC1)	HC-1
	Active Listening (HC2)	HC-2
	Context Clarification (HC3)	HC-3
	Confidence Empowerment (HC4, CEMI)	CEMI-3, CEMI-4, AICM-6
	Personalized Intervention (HC5,AICM)	AICM-8
	Feedback Handling (HC6, CUQ)	CUQ-6, CUQ-4
	Behavioral Motivation (CEMI)	CEMI-1, CEMI-2, AICM-7
	Conversation Flow (CUQ)	CUQ-1, CUQ-2
	Content Understanding & Inspiration (CUQ)	CUQ-3, CUQ-5
	Working Alliance (WAI)	WAI-1, WAI-2, WAI-3, WAI-4
	Progress Measurement (SMART)	SMART-2, SMART-5
	Goal Attainability & Alignment (SMART)	SMART-1, SMART-3, SMART-4
	Friendliness (AICM)	AICM-1
	Supportiveness (AICM)	AICM-2
	Clarity (AICM)	AICM-3
	Rapport Development (AICM)	AICM-4
	Information Gathering (AICM)	AICM-5
	Conversation Wrap Up (AICM)	AICM-9
Agents Ranking	Motivational Interview Skill	MI
	Health Recommendation Quality	Rec
	Agent Credibility	Cred
	Goal & Purpose Identification	HC-1
	Active Listening	HC-2
	Context Clarification on Priorities & Constraints	HC-3
	User Empowerment	HC-4
	Personalized & concrete Recommendations	HC-5
	User Feedback incorporation	HC-6

E.3 HC Agent Evaluation Results

E.3.1 Task 1: Health Coach Agent Capability from End-Users' Perspectives

Supplemental Table S28 | **HC Agent Evaluation Rubrics Results by End-users (N=31)**. Each end-user has their own interactions with the agents and gives their ratings. Thus, IRR is not applicable. Performance was the average scores of different aspects in structured measures (CEMI, CUQ, SMART, WAI), as listed in Table S22 and Table S24. GLMM with Gamma link function was fitted for statistical tests. Each rubric is treated as a confirmatory test of a pre-specified, conceptually distinct coaching dimension; an across-rubric Holm–Bonferroni robustness check preserves the strongest HC-Agent advantages (Feedback Handling and Goal Attainability & Alignment, $p < 0.001$; Behavioral Motivation, $p = 0.005$), while the marginal results on Conversation Flow ($p = 0.025$) and Progress Measurement ($p = 0.010$) are reported as directional findings that do not survive a strict across-rubric correction.

Rubrics	Base Gemini Performance	HC Agent Performance	β	Stats	p-value
Behavioral Motivation (CEMI)	57.1±3.8 %	**65.8±3.2 %**	0.172	t=2.83	0.005**
Confidence Empowerment (CEMI)	55.8±4.3 %	58.7±4.1 %	0.061	t=0.82	0.412
Content Understanding & Inspiration (CUQ)	67.4±3.9 %	74.8±3.2 %	0.119	t=1.90	0.058
Conversation Flow (CUQ)	64.5±3.4 %	**75.5±3.3 %**	0.164	t=2.25	0.025*
Feedback Handling (CUQ)	60.7±3.8 %	**66.1±2.9 %**	0.111	t=26.31	<0.001***
Goal Attainability & Alignment (SMART)	68.4±4.0 %	**70.8±3.2 %**	0.053	t=14.05	<0.001***
Progress Measurement (SMART)	**61.3±4.5 %**	48.7±3.9 %	-0.23	t=-2.59	0.010**
Working Alliance (WAI)	61.9±3.8 %	65.0±3.2 %	0.064	t=0.96	0.337

Supplemental Table S29 | **HC Agent Side-by-Side Comparison Results by End-users (N=31)**. IRR is not applicable due to the same reason above. Performance was measured as the win rate. Bootstrap was applied (1000 times sampling with replacement) to calculate the standard error. Binomial GLMM was fitted for statistical tests. Each ranking dimension is treated as a confirmatory test of a pre-specified, conceptually distinct coaching theme. The advantage on Active Listening is reported as a directional finding that does not survive a strict across-dimension Holm–Bonferroni correction.

Rubrics	Base Gemini Win Rate	HC Agent Win Rate	β	Stats	p-value
Goal & Purpose Identification (HC1)	35.5±8.5%	64.5±8.5%	0.598	Z=1.59	0.111
Active Listening (HC2)	29.0±8.0%	**71.0±8.0%**	0.894	Z=2.23	0.025*
Context Clarification on Priorities & Constraints (HC3)	38.7±8.8%	61.3±8.8%	0.46	Z=1.25	0.213
User Empowerment (HC4)	51.6±9.4%	48.4±9.4%	-0.065	Z=-0.18	0.857
Personalized & concrete Recommendations (HC5)	38.7±8.8%	61.3±8.8%	0.46	Z=1.25	0.213
User Feedback incorporation (HC6)	32.3±8.4%	67.7±8.4%	0.742	Z=1.93	0.053

E.3.2 Task 2: Validation of Health Coach Agent Design with Expert Evaluation

Supplemental Table S30 | **HC Agent Rubrics Evaluation Results by Experts (N=3)**. Both base Gemini (ICC(3,1)=0.794) and HC agent (ICC(3,1)=0.690) receive good IRR. Performance was the average scores of six themes (HC1-6) and structured measures (CEMI, CUQ, SMART, WAI, AICM), as listed in Table S25 and Table S27. GLMM with Gamma link function was fitted for statistical tests. Each rubric is treated as a confirmatory test of a pre-specified, conceptually distinct coaching dimension; an across-rubric Holm–Bonferroni robustness check does not change the statistical significance of any contrast.

Rubrics	Base Gemini Performance	HC Agent Performance	β	Stats	p-value
Goal Identification (HC1)	60.3±2.6 %	**72.3±1.9 %**	0.213	t=4.54	<0.001***
Active Listening (HC2)	64.1±2.7 %	**75.8±2.0 %**	0.200	t=4.11	<0.001***
Context Clarification (HC3)	60.0±2.7 %	**77.6±1.8 %**	0.307	t=6.28	<0.001***
Confidence Empowerment (HC4,CEMI)	48.5±1.9 %	**59.8±1.9 %**	0.227	t=5.83	<0.001***
Personalized Intervention (HC5,AICM)	58.9±2.6 %	**71.7±2.1 %**	0.231	t=4.87	<0.001***
Feedback Handling (HC6,CUQ)	55.3±2.1 %	**66.3±1.4 %**	0.197	t=3.94	<0.001***
Behavioral Motivation (CEMI)	52.9±2.1 %	**65.7±1.7 %**	0.246	t=6.02	<0.001***
Content Understanding (CUQ)	62.3±2.2 %	**75.1±1.7 %**	0.208	t=5.05	<0.001***
Conversation Flow (CUQ)	66.5±2.2 %	**78.0±1.2 %**	0.183	t=4.68	<0.001***
Goal Attainability & Alignment (SMART)	57.6±2.5 %	**67.0±1.9 %**	0.192	t=4.00	<0.001***
Progress Measurement (SMART)	**50.3±2.4 %**	46.7±1.4 %	-0.012	t=-3.86	<0.001***
Working Alliance (WAI)	61.4±2.2 %	**69.9±2.0 %**	0.149	t=3.80	<0.001***
Friendliness (AICM)	72.3±2.1 %	**79.4±1.3 %**	0.106	t=3.05	0.002**
Supportiveness (AICM)	63.2±2.4 %	**73.8±1.8 %**	0.188	t=4.82	<0.001***
Clarity (AICM)	75.1±2.2 %	75.7±1.8 %	0.016	t=0.42	0.678
Rapport Development (AICM)	56.6±2.6 %	**69.7±1.8 %**	0.258	t=5.39	<0.001***
Information Gathering (AICM)	54.2±3.0 %	**69.9±2.3 %**	0.302	t=4.72	<0.001***
Conversation Wrap Up (AICM)	58.3±3.2 %	**66.5±2.4 %**	0.186	t=3.23	0.001**

Supplemental Table S31 | HC Agent Side-by-Side Comparison Results by Experts (N=3). Our evaluations show good IRR (Fleiss' κ =0.640). Performance was measured as the win rate. Bootstrap was applied (1000 times sampling with replacement) to calculate the standard error. Binomial GLMM was fitted for statistical tests. Each ranking dimension is treated as a confirmatory test of a pre-specified, conceptually distinct coaching theme. An across-dimension Holm–Bonferroni robustness check preserves the strongest HC-Agent advantages (HC3 Context Clarification, HC6 Feedback Incorporation, HC2 Active Listening), while the marginal results on HC1, HC4, and HC5 are reported as directional findings that do not survive a strict across-dimension correction.

Rubrics	Base Gemini Win Rate	HC Agent Win Rate	β	Stats	p-value
Motivational Interview Skill	40.9±5.1%	59.1±5.1%	0.449	Z=1.52	0.128
Health Recommendation Quality	46.2±5.2%	53.8±5.2%	0.17	Z=0.66	0.506
Agent Credibility	33.3±4.9%	66.7±4.9%	0.746	Z=1.86	0.063
Goal & Purpose Identification (HC1)	33.3±5.0%	**66.7±5.0%**	1.19	Z=2.17	0.030*
Active Listening (HC2)	31.2±4.8%	**68.8±4.8%**	0.808	Z=2.82	0.005**
Context Clarification (Priorities, Constraints) (HC3)	28.0±4.8%	**72.0±4.8%**	1.052	Z=257.65	<0.001***
User Empowerment (HC4)	30.1±4.6%	**69.9±4.6%**	0.988	Z=2.00	0.045*
Personalized & concrete Recommendations (HC5)	35.5±4.9%	**64.5±4.9%**	0.649	Z=2.09	0.037*
User Feedback incorporation (HC6)	29.0±4.8%	**71.0±4.8%**	1.005	Z=3.19	0.001**

F PHA Additional Results and Technical Details

F.1 Prompt Setups

The prompt structure of the PHA, as well as the two baselines (i.e., single-agent PHIA and parallel multi-agent system), is summarized in this section. In addition to the prompts content, the complete conversation history is also included in the prompt following the tag “[CONVERSATION]”.

F.1.1 PHA Orchestrator Role Definition and Integration of Other Agents

We define the role of PHA orchestrator with the following system prompt. The orchestrator will be responsible for various tasks, whose prompts will be appended after the system prompt.

```
"""
You are an expert in personal health assistance and a helpful conversational orchestrator.
You will be responsible for organizing the conversation between the user and the team of
    agents.
Your job is to guide the conversation and help the user achieve their goals.
You will use a combination of tools and reasoning to guide the conversation.

You will have the following expert agents to leverage:
  - Data Science Agent: This agent will act as an expert in data science.
    It is responsible for analyzing the user's personal data and compute specifc values from
         the data, if the question is related to the user's personal data.
  - Domain Expert Agent: This agent will act as an expert in health and medical domains.
    It is responsible for providing domain-specific information about the user's question,
        if the question needs domain knowledge.
  - Health Coach Agent: This agent will act as an expert in health coach.
    It is responsible for guiding the user and helping them set and achieve their goal, if
        the question needs health coach advice.

These are the background setup of your role and your tools.
Next, you will be given more specific instructions. Follow the instructions.
Only answer the instructions. Don't say anything like "I understand", your thoughts or
    planned steps, or any additional information.
"""
```

F.1.2 PHA Orchestrator Task Assignment

One of the key functions for the orchestrator is to assign DS, DE, and HC agents based on user's queries.

```
"""
Given the user's current [CONVERSATION] and [TOPIC], determine the workflow by identifying
    the main agent and any supporting agents needed from the team of the agents.

Consider these points:
- **Main Agent:** Which agent is best suited to lead the response to the user's question?
- **Supporting Agents:** Which agents are necessary to provide additional information or
    insights to the main agent's response?
- **Collaboration Workflow:** How should the agents collaborate to provide the most
    comprehensive and helpful response?

Follow the following steps:

  [**STEP 1**: Check the corner cases]. Check whether the user's topic falls into the corner
      cases. Refer to the following corner caseexamples to return the answer:
  {multi_agent_collaboration_examples.MULTI_AGENT_COLLABORATION_CORNER_CASES}

  [**STEP 2**: Assign agent workflow]. If not in the corner cases, refer to the following
      topic caregory list and their corresponding main agent, supporting agents, and
      collaboration workflow:
    {multi_agent_collaboration_examples.MULTI_AGENT_COLLABORATION_EXAMPLES}.
    Try the best to match the user's question with one of the topic category.
    If there are multiple categories matched, select the category that has the most
        supporting agents and complex collaboration workflow.
    Once you match the topic, take the main agent, supporting agents, and collaboration
        workflow from.

  [**STEP 3**: Consider adding data science agent]. As long as the user asks about a
      question that can be potentially relevant to their personal data, even just a bit,and
      the main agent is not the data science agent, add the data science agent as a
      supporting agent and update the collaboration workflow accordingly.
    If the user asks about the data that is not relevant to the above list at all, then don'
        t add the data science agent as a supporting agent.

  [**STEP 4**: Safetyfallback]. If none of the above examples matches, use the answer:
    Main Agent: ""
    Supporting Agents: ""
    Collaboration Workflow: Fall back to basic gemini.

Finally, return the answer in the following json string format.
Provide the JSON data only, without any additional formatting, code blocks, or explanations.
Do not include triple backticks or specify the language. Just output the raw JSON:
\{\{
    "main_agent": the string of Main Agent (str),
    "supporting_agents": the string of Supporting Agents, splited by ';' (str),
    "collaboration_workflow": the string of Collaboration Workflow (str)
\}\}
"""
```

In the prompt, corner cases are a list of examples on non-health scenarios.

```
"""
Corner Cases about unsupported user question categories:
  # Ask about general health information
    - "I want to find a care provider in my area for x"
  # I'm here to Manage my experience
    - "I want to manage which data and sources it uses"
    - "I want to see all my past conversations"
    - "I want to delete past conversations"
    - "I want to log data"
  # I'm here to Share my Data
    - "I want to create social content"
    - "I want to create a report"
  # I'm here to Get Help with my Device
    - "I want help troubleshooting"
    - "I want help managing my account"
Main Agent: ""
Supporting Agents: ""
Collaboration Workflow: Fall back to basic gemini.
"""
```

Agent workflow examples were based on the Table S1. summarized from real-world user journey categories. A interdisciplinary group of experts cross health, data science, and human-computer interaction designed an appropriate set of collaboration setups for these user query categories.

```
"""
**No.1**
User Question Categories:
  # I'm here to Understand and discover Health Topics
    - "I want to understand x"
    - "I want to find a fact about x"
    - "I want to compare x and y"
    - "I want the pros and cons of x"
    - "I want the news about x"
    - "I want to find images of x"
  # I'm here to Understand Certain Health Record
    - "I want to answer a question from x"
    - "I want to summarize x (at certain timestamp)"
Main Agent: "Domain Expert Agent"
Supporting Agents: ""
Collaboration Workflow: Answer the user's question based on Domain Expert Agent's knowledge.

**No.2**
User Question Categories:
  # I'm here to Understand My Time-Series Health Data that only needs data from single
      source
    - "I want to understand what my x data says about my health"
    - "I want to see or compare my x data for a specific time periods"
    - "I want to identify meaningful changes in x"
    - "I want to see my best/worst x data"
    - "I want to see relationships between x and y in the same source (e.g., Fitbit)"
Main Agent: "Data Science Agent"
Supporting Agents: ""
Collaboration Workflow: Answer the user's question based on Data Science Agent computation
    and the results.
```

```
**No.3**
User Question Categories:
  # I'm here to Understand My Time-Series Health Data that needs external knowledge for
      interpretation and reasoning
    - "I want to understand why x changed"
    - "I want to compare my x with other people / clinical guidelines"
    - "I want to summarize x time-series data"
    - "I want to see relationships between x and y across sources (e.g., Fitbit & Health
        Record)"
Main Agent: "Domain Expert Agent"
Supporting Agents: "Data Science Agent"
Collaboration Workflow: Let Data Science Agent do the computation based on users' request,
  then Domain Expert Agent will interpret the results and answer the user's question.

**No.4**
User Question Categories:
  # I'm here to Get Wellness advice
    - "I want advice on x in general"
  # I'm here to Progress
    - "I want to set an appropriate goal or task for x"
    - "I want plans to achieve my x goal"
    - "I want to adjust my plans or goals based on changing needs"
Main Agent: "Health Coach Agent"
Supporting Agents: ""
Collaboration Workflow: Let the Health Coach Agent to guide the user and help them set and
    achieve their goal.

**No.5**
User Question Categories:
  # I'm here to Get Wellness advice based on my health data
    - "I want advice on x based on my data"
  # I'm here to Progress based on my health data
    - "I want to set an appropriate goal or task for x based on my data"
    - "I want plans to achieve my x goal based on my data"
    - "I want to adjust my plans or goals based on changing needs based on my data"
    - "I want track progress against my goals for x"

Main Agent: "Health Coach Agent"
Supporting Agents: "Data Science Agent"
Collaboration Workflow: Let Data Science Agent do the computation on the user's health data
    based on their request,
  then let the Health Coach Agent to guide the user and help them set and achieve their goal
      .

**No.6**
User Question Categories:
  # I'm here to Get Wellness advice that involves both health data and health domain
      knowledge
    - "I want to identify areas for improvement"
  # I'm here to Medical advice based on my health data
    - "I want advice on a health condition"
  # I'm here to Progress based on my health data and health domain knowledge
    - "I want to understand how my behaviors move me toward my goal for x"

Main Agent: "Health Coach Agent"
Supporting Agents: "Data Science Agent;Domain Expert Agent"
Collaboration Workflow: Let Data Science Agent do the computation on the user's health data
    based on their request,
  and let the Domain Expert Agent to provide domain-specific information about the user's
      question,
```

```
    then pass both information to the Health Coach Agent and let it guide the user and help
        them set and achieve their goal.

"""
```

F.1.3 PHA Orchestrator Question Rephrase

After agent assignment, the orchestrator will then rephrase the question for each specific agent:

```
"""
You get this question from the user: "{{original_question}}".
You have the main agent as {{main_agent}} and supporting agents as {{supporting_agents}},
and the following workflow across the agents: {{collaboration_workflow}}.
Now you need to decompose the user's question, and frame it properly for each agent (
    including the main agent and supporting agents) to answer it.

The rephrased prompt should be a clear question that can be answered by the supporting agent
     and can support main agents to answer the user's question.
Provide the JSON data only, without any additional formatting, code blocks, or explanations.
"""
```

- `original_question`: The end-user's raw question before rephrasal.
- `main_agent/supporting_agents`: The main agent and supporting agent(s).
- `collaboration_workflow`: The workflow among the main agent and supporting agent(s) assigned by the orchestrator.

F.1.4 PHA Reflection Prompt

After getting responses from the main agent and the supporting agent(s), PHA will conduct the following reflection step. If reflection is needed, new questions will be self-generated and answered to update its outcome before responding to end users.

```
"""
You get this question from the user: "{original_question}".
You have the main agent as {main_agent} and supporting agents as {supporting_agents},
and the following workflow across the agents: {collaboration_workflow}.
Now the insights from the supporting agents are as follows:
  {{supporting_agent_insights}}

And the response from the main agent is as follows:
  {{main_agent_response}}


Now you need to reflect on the response from the main agent before responding to the user.
Your goal is to avoid asking the user with some questions that can already be answered from
    the personal data.
For example, questions like "What time do you usually go to bed and wake up?" can be
    answered by the data science agent by looking at the user's data.
So in this case, you should reflect on it and ask the data science agent to get answers.

Output the raw JSON:
{{
    "decision": [decision] (str),
    "reflection_questions": [reflection_questions_dictiontary] (Dict[str, str])
}}

[decision] can only be "YES" or "NO".
Return [decision] as "NO" means the main agent response cannot be further improved by asking
     any supporting agents and need input from the user. So the main agent response is good
    enough. In this case, [reflection_questions_dictiontary] should be an empty dictionary.
Return [decision] as "YES" if you think the main agent response can be improved by asking
    the supporting agents some additional questions.
In this case, [reflection_questions_dictiontary] should be a dicitionary with the following
    format:
{{
    [one_supporting_agent]: [agent_specific_questions] (str)
}}
The [one_supporting_agent] will be one of the supporting agents in the {{supporting_agents
    }}.
The [agent_specific_questions] should be a string of additional questions to be answered by
    the correponding supporting agents. Don't ask redundant questions that is already in the
     [SUPPORTING_AGENT_INSIGHTS].
Keep the list of [one_supporting_agent] small and only include the supporting agents that
    you think will be helpful to get more information to complete the main agent response,
    and keep the questions as minimal and specific as possible.
"""
```

- `original_question`, `main_agent`, `supporting_agentsm`, `collaboration_workflow`: Same as those in D.1.3.
- `main_agent_response`: The output generated by the main agent.
- `supporting_agent_insights`: A text piece that includes responses from all supporting agent(s). Each supporting agent's insight section will be updated in each turn throughout the multi-turn conversation.

F.1.5 Singe Agent Baseline PHIA Prompts

We re-implemented the PHIA agent in prior work (Merrill et al., 2024) as the baseline single-agent system with an ReAct framework. The following system prompt sets up the role of the PHIA:

```
"""
Use tools such as tool_code to execute Python code and search to find external relevant
    information as needed. Tell the user you cannot answer the question if it is not health
    and wellness related. Take into account that questions may have typos or grammatical
    mistakes and try to reinterpret them before answering. Follow all instructions and the
    ReAct template carefully. Answer the following question in detail and with suggestions
    when appropriate. Always make sure the final answer is nicely formatted and does not
    contain incorrectly formatted ReAct steps. Remember to use user data whenever relevant
    to enhance your answers!

{#- Preamble: Agent functionality description -#}
I am going to ask you a question about Fitbit data. Assume that you have access
to pandas through `pd` and numpy through `np`. You DO NOT have access to matplotlib or other
     python libraries.

Carefully consider examples of how different tasks can be solved with different tools and
    use them to answer my questions.
Be sure to follow the ReAct protocol as specified and be careful with tool usage (e.g., use
    only one tool at a time).
You can expect questions to be conversational and multi-turn, so avoid overfixating on a
    single turn or a past turn at any point.

#### You have access to the two dataframes below:

- `summary_df`: This is a summary of the user's activity and sleep data. It's a pandas
    DataFrame with the following columns:
    - `datetime`: The date of the record (datetime64[ns]).
    - `resting_heart_rate`: The average resting heart rate (float64) in beats per minute.
    - `heart_rate_variability`: The user's heart rate variability (float64).
    - `fatburn_active_zone_minutes`: The number of minutes spent in the fat burn active zone
         (float64).
    - `cardio_active_zone_minutes`: The number of minutes spent in the cardio active zone (
        float64).
    - `peak_active_zone_minutes`: The number of minutes spent in the peak active zone (
        float64).
    - `active_zone_minutes`: The number of active zone minutes earned each day (float64).
    - `steps`: The number of steps taken each day (float64).
    - `rem_sleep_minutes`: The amount of REM sleep (float64) in minutes.
    - `deep_sleep_minutes`: The amount of deep sleep (float64) in minutes.
    - `awake_minutes`: The amount of awake time (float64) in minutes.
    - `light_sleep_minutes`: The amount of light sleep (float64) in minutes.
    - `sleep_minutes`: The total sleep time (float64) in minutes.
    - `bed_time`: The time the user went to bed (datetime64[ns]).
    - `wake_up_time`: The time the user woke up (datetime64[ns]).
    - `stress_management_score`: The user's stress management score for each day (float64).
    - `deep_sleep_percent`: The percentage of sleep time spent in deep sleep (float64).
    - `rem_sleep_percent`: The percentage of sleep time spent in REM sleep (float64).
    - `awake_percent`: The percentage of sleep time spent awake (float64).
    - `light_sleep_percent`: The percentage of sleep time spent in light sleep (float64).

- `activities_df`: This is a list of the user's activities. It's also a pandas DataFrame
    with the following columns:
    - `startTime`: The start time of the activity (datetime64[ns]).
    - `endTime`: The end time of the activity (datetime64[ns]).
```

```
    - `activityName`: The name of the activity (object). (One of INTERVAL_WORKOUT,
        STRENGTH_TRAINING, TREADMILL_TRACKER_RECORDED, STAIRCLIMBER,
        SPINNING_TACKER_RECORDED, WEIGHTLIFTING, PILATES, SNOWBOARDING, SURFING, KICKBOXING,
         MARTIAL_ARTS, WALKING, ELLIPTICAL, OUTDOOR_WORKOUT, HIIT, AEROBIC_WORKOUT, SPORT,
        CROSS_COUNTRY_SKI, POWERLIFTING, TENNIS, ROWING_MACHINE, CIRCUIT_TRAINING, BIKING,
        SKIING, TREADMILL_USER_LOG, PADDLEBOARDING, WORKOUT, WEIGHTS_TRACKER_RECORDED,
        ROLLERBLADING, AEROBICS_NEW, GOLF, STRETCHING, DANCING, CORE_TRAINING, KAYAKING,
        SKATING, INDOOR_CLIMBING, CANOEING_NEW, MEDITATE, OUTDOOR_BIKE, SPINNING_USER_LOG,
        WEIGHTS_USER_LOG, HIKE, SWIMMING, YOGA, BOOTCAMP, CROSSFIT, RUNNING, ELLIPTICAL_AUTO
        ).
    - `distance`: The distance covered during the activity (float64) in miles.
    - `duration`: The duration of the activity (float64) in minutes.
    - `elevationGain`: The total elevation gain during the activity (float64) in meters.
    - `averageHeartRate`: The average heart rate during the activity (float64) in beats per
        minute.
    - `calories`: The number of calories burned during the activity (float64).

#### You also have access to a profile dataframe:

The profile dataframe contains the following keys:
    - `age`: The age of the user (int).
    - `gender`: The gender of the user (str).
    - `averageDailySteps`: The average number of steps taken each day (int).
    - `elderly`: Whether the user is elderly ("Yes" or "No") (str).
    - `height_cm`: The height of the user in centimeters (int).
    - `weight_kg`: The weight of the user in kilograms (int).

## ReAct Prompt Setup

{#- Preamble: Tools description -#}
{\%- role name='system' -\%}
Here is a list of available tools:
{\% for tool in tools \%}
Tool name: {{ tool.name }}
Tool description: {{ tool.description }}
{\% if tool.example -\%}
  Tool example: {{ tool.example_str }}
{\%- endif -\%}
{\% endfor \%}

{#- Preamble: ReAct few-shots #}
Here are examples of how different tasks can be solved with these tools. Never copy the
    answer directly, and instead use examples as a guide to solve a task:
{\% for example in exemplars \%}
[{{ stop_prefix }}Question]: {{ example.inputs + '\n' }}
{\%- for step in example.updates -\%}
{\%- if step.thought -\%}
  [Thought]: {{ step.thought + '\n' }}
{\%- endif -\%}
{\%- if step.action -\%}
  [Act]: {{ step.render_action() + '\n' }}
{\%- endif -\%}
{\%- if step.observation and step.action -\%}
  [{{ stop_prefix }}Observe]: {{ step.render_observation() + '\n' }}
{\%- endif -\%}
{\%- if step.is_finished and step.observation -\%}
  [Finish]: {{ step.observation + '\n' }}
{\%- endif -\%}
{\%- endfor -\%}
{\%- endfor -\%}
```

```
Carefuly consider examples of how different tasks can be solved with different tools and use
     them to answer my questions.
Be sure to follow the ReAct protocol as specified and be careful with tool usage (e.g., use
    only one tool at a time).
You can expect questions to be conversational and multi-turn, so avoid overfixating on a
    single turn or a past turn at any point.

{# Start of the processing of the actual inputs. -#}

Here is the question you need to solve and your current state toward solving it:
{#- Render the original question. -#}
{\%- endrole -\%}
{\%- role name='user' \%}
[{\%- role name='system' -\%}{{ stop_prefix }}{\%- endrole -\%}Question]: {{ state.inputs +
    '\n' }}
{\%- endrole -\%}

{# Render the current state (i.e., any steps performed up till now). -#}
{\%- for step in state.updates -\%}
{\%- if step.thought -\%}
  [Thought]: {{ step.thought + '\n' }}
{\%- endif -\%}
{\%- if step.action -\%}
  [Act]: {{ step.render_action() + '\n' }}
{\%- endif -\%}
{\%- if step.observation and step.action -\%}
  [{{ stop_prefix }}Observe]: {{ step.render_observation() + '\n' }}
{\%- endif -\%}
{\%- if step.is_finished and step.observation -\%}
  [Finish]: {{ step.observation + '\n' }}
{\%- endif -\%}
{\%- endfor -\%}

{# If force-finishing, then prompt the LLM for the final answer. -#}
{\%- if force_finish -\%}
  [Finish]:{{ ' ' }}
{\%- endif -\%}

{#- Get a response from the LLM and return it. -#}
{\%- role name='llm' -\%}
  {{- store('llm_reply', generate_text(stop=stop_sequences)) -}}
{\%- endrole -\%}
"""
```

PHIA has access to the following tools, together with few-shot examples of calling these tools: (1) Python Sandbox, prompted as ”Python interpreter. Can be used as a calculator or to execute any Python code. Returns the result of execution. This tool should never call search() or finish().” (2) Search tool, prompted as ”Search engine. Returns a relevant snippet or answer to query.” (3) Finish tool, prompted as ”Function for returning the final answer. The thought involved should be appropriately included in the call to finish(), for example finish(’details from thought incorporated into final answer’)”.

F.1.6 Parallel Multi-Agent Baseline: Prompts

The parallel multi-agent system baseline employs the same DS, DE, and HC agents as PHA. There is no need for task assignment and collaboration. All three agents will get the same user query and generate outcomes in parallel independently. The role of the Orchestrator is to synthesize the output with the following prompts:

```
"""
DS agent insights: {{Responses from DS agents}}
DE agent insights: {{Responses from DE agents}}
HC agent insights: {{Responses from HC agents}}

[CONVERSATION]: {{conversation_history}}

Given these insights and the conversation history, now synthesize these insights and answer
    the user's request.

Do not directly include any of the insights in the response. Do not refer to any specific
    agent. Just act as an orchestrator and synthesize the response based on the insights.
"""
```

F.2 PHA Evaluation Rubrics Details

F.2.1 Task 1: End-User Evaluation Rubrics

Since each end-user will play the WEAR-ME persona role and directly interact with the agent, they receive the following instruction after reading the user persona data. It is noteworthy that participants were explicitly instructed to exclude system latency from the evaluation consideration:

```
Your ultimate goal is to assess the quality of the conversation as if you were the user
    whose profile you are given.

Evaluation Steps

- Discover Your Persona:
   - Review the User Persona doc shared by the proctor.
   - Learn the user's background, story, and their specific health goals for the
       conversation. Ask any questions you have.

- Embrace the Role:
   - Fully internalize the user's situation, feelings, and what is most important to them.
   - Chat with the AI and make all decisions as if you are this person.
   - [Note]: The data in the profile doc is for context. The specific numbers the agent uses
        in the chat may differ slightly.

- Relive the Conversation:
   - Access the first agent following the proctor's instructions.
   - Filter every response through your persona's perspective: Does the agent meet your
       needs? Is it helpful as a personal assistant/health coach?

- Complete the Survey:
   - Move to the survey and answer the questions based entirely on your experience playing
       the role of the user in that conversation.

What to Ignore (Do Not Score)

- Response Time: The agent may take several minutes to reply while analyzing data. Ignore
    any conversation delay. Focus only on the content of the response.
- Formatting: Ignore how the text is presented (e.g., bullet points, bolding). Focus only on
     the actual information.

Agent Memory: You will talk to three agents. Each conversation is a new start; the agents do
     not share memory of your previous chats, but your user persona remains the same for all
     three.
```

Supplemental Table S32 | PHA Generalist Raw Evaluation Questions after Each Multi-Turn Conversation with Agents. Ranking questions are based on the same 18 rubrics after reading all conversations.

Question No.	Evaluation Question Body	Scoring/Ranking Question
Overall-1	How satisfied are you after the conversation?	1: Very Dissatisfied - The conversation was unhelpful or frustrating.<br>2: Dissatisfied - The conversation had significant issues and did not meet my needs.<br>3: Neutral - The conversation was okay, but not particularly helpful or satisfying.<br>4: Satisfied - The conversation was generally helpful and met most of my needs.<br>5: Very Satisfied - The conversation was highly helpful, insightful, and fully met my needs.
Overall-2	What's the overall quality of the agent response?	1: Very Poor - Responses were consistently irrelevant, incorrect, confusing, or inappropriate.<br>2: Poor - Responses had frequent issues with relevance, correctness, clarity, or appropriateness.<br>3: Fair - Responses were a mix of good and bad; some were helpful, others were not.<br>4: Good - Responses were mostly relevant, correct, clear, and appropriate.<br>5: Excellent - Responses were consistently relevant, correct, clear, appropriate, and insightful.
DS-1	The agent involves all of my useful and relevant personal data to answer my questions. (Note: Data accessible to the agent: basic info, Fitbit, blood tests)	1: None of the relevant user data is referenced or used.<br>2: Only a few minor pieces of relevant user data are referenced; most important data is missed.<br>3: About half of the most relevant user data is referenced and used appropriately.<br>4: Most of the relevant and important user data is referenced and used appropriately.<br>5: All or nearly all relevant and important user data is referenced and used appropriately where applicable.
DS-2	The agent does not involve irrelevant or not useful personal data.	1: Agent frequently uses irrelevant personal data, causing confusion or distraction.<br>2: Agent sometimes uses irrelevant personal data.<br>3: Agent occasionally uses slightly irrelevant data, but it doesn't significantly detract.<br>4: Agent very rarely uses irrelevant data; responses focus on pertinent information.<br>5: Agent exclusively uses relevant personal data; no irrelevant data is included.

Continued on next page

Question No.	Evaluation Question Body	Scoring/Ranking Question
DE-1	The agent involves useful and relevant health knowledge in the conversation to help answer my questions.	1: No relevant health knowledge is apparent in the agent's responses.<br>2: Very little relevant health knowledge is used, or it's applied incorrectly.<br>3: Some relevant health knowledge is used, but significant gaps exist or it's not always applied effectively.<br>4: Most responses appropriately incorporate relevant health knowledge.<br>5: Responses consistently and effectively integrate useful and relevant health knowledge.
DE-2	The agent does not involve irrelevant or not useful health domain knowledge.	1: Agent frequently includes irrelevant or incorrect health information, causing confusion.<br>2: Agent sometimes includes irrelevant health information or jargon.<br>3: Agent occasionally includes minor pieces of irrelevant knowledge, but it's not disruptive.<br>4: Agent very rarely includes irrelevant knowledge; focus remains on pertinent information.<br>5: Agent exclusively uses relevant and accurate health knowledge; no irrelevant information included.
DS-DE-1	The agent provides useful interpretation and contextualization of my health data that is helpful for addressing my needs.	1: No interpretation provided, or interpretation is incorrect/useless.<br>2: Minimal interpretation provided, or it lacks context/utility.<br>3: Some useful interpretation and contextualization provided, but could be clearer or more insightful.<br>4: Good interpretation and contextualization provided, generally helpful.<br>5: Excellent, insightful interpretation and contextualization provided, very helpful for understanding my health status and needs.
HC-1	The agent's tone was supportive and encouraging.	1: Agent felt dismissive, critical, or completely unsupportive.<br>2: Agent offered minimal or superficial support/encouragement.<br>3: Agent was somewhat supportive and encouraging.<br>4: Agent was generally supportive and encouraging throughout the conversation.<br>5: Agent was consistently very supportive and encouraging, making me feel motivated.

Continued on next page

Question No.	Evaluation Question Body	Scoring/Ranking Question
HC-2	The agent understood my preferences and barriers.	1: Agent completely ignored or misunderstood my stated preferences and barriers, or did not about ask my context before providing suggestions.<br>2: Agent showed little understanding or acknowledgment of my preferences and barriers.<br>3: Agent showed some understanding but missed key aspects or didn't integrate them well.<br>4: Agent showed good understanding and consideration of most of my preferences and barriers.<br>5: Agent demonstrated a clear and accurate understanding of my preferences and barriers, incorporating them into the conversation.
HC-3	The agent handled conversation flow naturally and effectively.	1: Conversation flow felt very unnatural, robotic, or difficult to continue.<br>2: Conversation flow felt awkward or stilted at times.<br>3: Conversation flow was acceptable but could have been smoother or more logical.<br>4: Conversation flow was mostly natural, logical, and easy to follow.<br>5: Conversation flow was very natural, smooth, engaging, and effective.
HC-4	The agent responses were useful, appropriate and informative.	1: Responses were generally useless, inappropriate, or uninformative.<br>2: Responses were often not very useful, appropriate, or informative.<br>3: Responses were somewhat useful, appropriate, and informative, but inconsistent.<br>4: Responses were mostly useful, appropriate, and informative.<br>5: Responses were consistently highly useful, appropriate, and informative.

Continued on next page

Question No.	Evaluation Question Body	Scoring/Ranking Question
HC-5	The agent provides personalized suggestions at an appropriate time.	1: Agent provided no personalized suggestions, or suggestions felt completely ill-timed (too early, too late, random).<br>2: Agent provided suggestions, but they often felt poorly timed or generic.<br>3: Agent provided personalized suggestions at somewhat appropriate times, but the timing could be improved.<br>4: Agent generally provided personalized suggestions at appropriate and logical points in the conversation.<br>5: Agent provided personalized suggestions seamlessly and at highly opportune moments.
HC-6	The agent handled my feedback well.	1: Agent ignored, dismissed, argued with, or responded negatively to my feedback.<br>2: Agent acknowledged feedback minimally but didn't seem to understand or use it.<br>3: Agent acknowledged feedback but struggled to incorporate it or respond effectively.<br>4: Agent handled my feedback reasonably well, acknowledged it, and attempted to adjust.<br>5: Agent handled my feedback excellently, acknowledged it clearly, and adapted its responses or approach appropriately.
HC-7	The agent helped me talk about changing my behavior to better align with my goal.	1: Agent did not help me talk about behavior change at all, or hindered the discussion.<br>2: Agent made minimal or ineffective attempts to discuss behavior change related to my goal.<br>3: Agent initiated some discussion about behavior change, but it wasn't very deep or helpful.<br>4: Agent effectively guided the conversation towards discussing relevant behavior changes.<br>5: Agent skillfully and supportively helped me explore specific behavior changes needed to reach my goal.
HC-8	The agent helped me feel confident in my ability to achieve the goal.	1: The conversation undermined my confidence or made me feel less capable.<br>2: The conversation had little to no positive impact on my confidence.<br>3: The conversation slightly increased my confidence in achieving the goal.<br>4: The conversation noticeably helped me feel more confident about achieving the goal.<br>5: The conversation significantly boosted my confidence and belief in my ability to achieve the goal.

Continued on next page

Question No.	Evaluation Question Body	Scoring/Ranking Question
HC-9	The agent helped me align the goal with my priorities, preferences, and barriers.	1: Agent did not help connect the goal to my personal context (priorities, preferences, barriers).<br>2: Agent made minimal or unclear attempts to connect the goal to my context.<br>3: Agent helped somewhat in connecting the goal, but the alignment wasn't fully explored or convincing.<br>4: Agent effectively helped me see how the goal aligns with my priorities, preferences, and acknowledged barriers.<br>5: Agent skillfully helped me integrate the goal with my personal context, making it feel relevant and achievable despite barriers.
HC-DS-1	The agent's suggestion is individualized to my personal data.	1: Suggestions felt completely generic, showing no connection to my personal data.<br>2: Suggestions showed minimal or superficial connection to my personal data.<br>3: Suggestions were somewhat individualized but could have leveraged my data much more effectively.<br>4: Suggestions were clearly individualized and tailored based on my relevant personal data.<br>5: Suggestions were highly individualized, demonstrating insightful and effective use of my personal data.
HC-DE-1	The agent's suggestion is grounded on health knowledge	1: Suggestions seemed arbitrary or lacked any apparent basis in health knowledge; potentially unsafe.<br>2: Suggestions had a weak or unclear connection to established health knowledge or principles.<br>3: Suggestions were generally aligned with health knowledge but felt generic or superficial.<br>4: Suggestions were clearly grounded in relevant and sound health knowledge.<br>5: Suggestions were strongly grounded in relevant health knowledge, often explaining the rationale clearly.

F.2.2 Task 2: Expert Evaluation Rubrics

Health experts receive the following instruction prior to reading the conversation scripts between a user persona (role-playing by an agent) with each personal health agent:

```
Your goal is to assess the conversation's quality from the experts' point of view. Get ready
    for the evaluation:
```

Supplemental Table S33 | PHA Generalist Evaluation Rubrics. Each rubric is an aggregation of one or multiple raw questions for better presentation.

Evaluation Rubrics	Question No.
Data Science Analysis (DS)	DS-1
Domain Knowledge Integration (DE)	DE-1, DE-2
Natural Conversation (HC)	HC-1, HC-4, HC-5
Personalized Coaching (HC)	HC-2, HC-3, HC-6, HC-9
User Empowerment (HC)	HC-7, HC-8
Health Data Interpretation (DS + DE)	DS-DE-1
Individualized Suggestions (DS + HC)	HC-DS-1
Grounded Suggestions (DE + HC)	HC-DE-1
Overall Quality (DS + DE + HC)	Overall-1

```
- Understand the Scenario: Review the User Persona document (link provided in your task
    materials). This context is crucial for understanding the user's situation, needs, and
    the challenges the agent faced. You need to grasp the user's background to accurately
    judge the agent's responses.
    [INFO] The data in the persona document is historical data and just to provide contexts.
         Therefore, the data involved in the conversations may not be exactly the same as
        the persona doc.

- Analyze the Agent's Performance: Now, open the first Conversation Script (link also
    provided). Read the dialogue, focusing critically on the agent's contributions. Analyze
    each of the agent's turns.

- Provide Your Expert Assessment: Finally, move on to the survey. Answer the questions based
     entirely on your professional evaluation of the agent's performance during this
    specific interaction.

Note 1: The same user independently talks to three agents (hense three conversation scripts)
    . The user persona retains consistency in general across these conversations.

Note 2: Numeric accuracy is not the focus in this evaluation, so you don't need to worry
    about data details. It is possible that three agents may state slightly different values
     (e.g., different sleep durations) due to minor difference in computation methods.
    Please ignore such details and focus on the conversation flow.

Note 3: Formatting of the content is also not the focus in this evaluation. Please pay more
    attention to the actual content rather than the response format (e.g., bullet points,
    bolding).
```

Supplemental Table S34 | PHA Expert Raw Evaluation Questions after Reading Each Multi-Turn Conversation with Agents. Ranking questions are based on the same 24 rubrics after reading all conversations.

Question No.	Evaluation Question Body	Scoring/Ranking Question
Overall-1	What's the overall quality of the agent response?	1: Very Poor Quality - Responses are clinically unsound, unsafe, irrelevant, or unusable.<br>2: Poor Quality - Responses frequently exhibit issues with clinical soundness, safety, or relevance.<br>3: Acceptable Quality - Responses are generally safe but may lack depth, relevance, or clinical nuance.<br>4: Good Quality - Responses are mostly sound, safe, relevant, and effective.<br>5: Excellent Quality - Responses are consistently sound, safe, relevant, effective, and demonstrate best practices.
Overall-2	How appropriate and effective was the overall conversation likely to be for a user with this profile and needs?	1: Highly Inappropriate/Ineffective - Conversation likely counterproductive, confusing, or potentially harmful for this user.<br>2: Inappropriate/Ineffective - Conversation poorly suited to the user's profile/needs; unlikely to be helpful.<br>3: Somewhat Appropriate/Effective - Conversation has some potential value but significant limitations or mismatches for this user.<br>4: Appropriate/Effective - Conversation is generally suitable for the user's profile/needs and likely helpful.<br>5: Highly Appropriate/Effective - Conversation is very well-suited to the user's profile/needs and likely highly beneficial.
DS-1	Did the agent appropriately utilize relevant user data that was available to it? Note: Data that is accessible to the agent: basic info (age, gender, height, weight), Fitbit records (step, sleep, activity), blood test results.	1: None/Minimal Utilization - Relevant available user data was ignored or barely used.<br>2: Poor Utilization - Key relevant data was frequently missed or clearly underutilized.<br>3: Fair Utilization - Some relevant data was used, but significant omissions or inconsistent application exist.<br>4: Good Utilization - Most relevant available user data was utilized appropriately.<br>5: Excellent Utilization - All relevant available user data was consistently and appropriately utilized.
DS-2	Did the agent appropriately avoid irrelevant or potentially noisy user data?	1: Very Poor Avoidance - Agent frequently focused on irrelevant/noisy data, potentially causing confusion or misdirection.<br>2: Poor Avoidance - Agent often included or emphasized irrelevant/noisy data.<br>3: Fair Avoidance - Agent sometimes included irrelevant/noisy data, but it didn't heavily derail the interaction.<br>4: Good Avoidance - Agent rarely included or focused on irrelevant/noisy data.<br>5: Excellent Avoidance - Agent consistently ignored irrelevant/noisy data, focusing only on pertinent information.

Continued on next page

Question No.	Evaluation Question Body	Scoring/Ranking Question
DE-1	Was the health information and knowledge provided by the agent accurate and factual?	1: Very Inaccurate - Information provided contains major factual errors or contradicts established knowledge.<br>2: Inaccurate - Information contains significant factual errors.<br>3: Mixed Accuracy - Information contains some inaccuracies or lacks sufficient precision.<br>4: Mostly Accurate - Information is generally correct, potentially with very minor, non-critical inaccuracies.<br>5: Highly Accurate - Information consistently aligns with current, evidence-based scientific facts.
DE-2	Did the agent avoid presenting inaccurate or outdated, or potentially harmful health information?	1: Very Poor Avoidance - Agent presented information that is potentially harmful, clearly contraindicated, or dangerously inaccurate/outdated.<br>2: Poor Avoidance - Agent presented information that could be harmful or is significantly inaccurate/outdated.<br>3: Fair Avoidance - Agent avoided directly harmful information but may have included some inaccurate or outdated points.<br>4: Good Avoidance - Agent avoided harmful information and provided mostly accurate and current information.<br>5: Excellent Avoidance - Agent completely avoided presenting harmful, inaccurate, or outdated information.
DE-3	Was the health information and knowledge provided by the agent appropriate and relevant for the user's health status and context?	1: Highly Inappropriate/Irrelevant - Information provided was clearly mismatched to the user's context, goals, or health status.<br>2: Inappropriate/Irrelevant - Information was largely unsuitable or irrelevant for this user.<br>3: Somewhat Appropriate/Relevant - Information had some relevance but significant portions were generic or mismatched.<br>4: Appropriate/Relevant - Information was generally suitable and relevant to the user's context.<br>5: Highly Appropriate/Relevant - Information was specifically tailored and highly relevant to the user's context, goals, and health status.
DE-4	Did the agent avoid presenting irrelevant or potentially confusing health information?	1: Very Poor Avoidance - Agent frequently presented irrelevant information or information in a confusing way, hindering understanding.<br>2: Poor Avoidance - Agent often included irrelevant or confusing information.<br>3: Fair Avoidance - Agent sometimes included irrelevant or confusing information, but it was manageable.<br>4: Good Avoidance - Agent rarely included irrelevant or confusing information.<br>5: Excellent Avoidance - Agent consistently presented only relevant information clearly and concisely.

Continued on next page

Question No.	Evaluation Question Body	Scoring/Ranking Question
DS-DE-1	Did the agent correctly interpret the available user data within its expected ranges and context?	1: Incorrect Interpretation - Interpretation was fundamentally flawed, wrong based on data, or ignored critical context.<br>2: Mostly Incorrect Interpretation - Contained significant errors in interpreting data values or trends.<br>3: Mixed Interpretation - Some correct interpretation mixed with noticeable errors or overlooked key contextual factors.<br>4: Mostly Correct Interpretation - Generally accurate interpretation with only minor issues, if any.<br>5: Correct & Contextualized Interpretation - Interpretation was accurate, sound, and considered relevant context.
DS-DE-2	Was the agent's interpretation and contextualization of the user's health data (integrating data points and knowledge) appropriate and useful?	1: Inappropriate/Useless - Interpretation lacked relevance, offered no practical value, or was potentially misleading.<br>2: Mostly Inappropriate/Useless - Interpretation offered little clinical value or practical application.<br>3: Somewhat Appropriate/Useful - Interpretation had some value but lacked depth, clear relevance, or practical application.<br>4: Appropriate/Useful - Interpretation was generally relevant, sound, and helpful.<br>5: Highly Appropriate/Useful - Interpretation was relevant, insightful, and provided clear, practical value for the user's understanding or action.
HC-1	Did the agent maintain an engaging and welcoming demeanor that fosters a positive and open environment?	1: Discouraging/Unwelcoming/Robotic - Tone felt negative, judgmental, dismissive, or overly impersonal, hindering openness.<br>2: Neutral/Indifferent - Tone lacked warmth or positive engagement; felt purely transactional.<br>3: Somewhat Engaging/Welcoming - Attempts engagement but felt inconsistent, superficial, or slightly awkward.<br>4: Engaging/Welcoming - Tone was generally positive, respectful, and fostered a good environment for communication.<br>5: Highly Engaging/Welcoming - Tone consistently fostered trust, rapport, openness, and positivity through warmth and respect.
HC-2	Did the agent show understanding, recognition, and encouragement in communication?	1: Dismissive/Unaware - Agent ignored or contradicted user's feelings/statements; lacked recognition or encouragement.<br>2: Minimal Recognition/Support - Very little evidence of understanding user's perspective; encouragement absent or generic.<br>3: Some Recognition/Support - Showed basic understanding or offered simple encouragement, but lacked depth or personalization.<br>4: Good Understanding/Support - Generally acknowledged user's perspective appropriately and offered suitable encouragement.<br>5: Highly Empathetic/Supportive - Consistently showed understanding (e.g., through reflections), validated user's perspective, and offered genuine, tailored encouragement.

Continued on next page

Question No.	Evaluation Question Body	Scoring/Ranking Question
HC-3	Did the agent enable the user to take charge of their actions and decisions confidently?	1: Disempowering/Prescriptive - Agent dictated actions, undermined user confidence, or overly controlled the conversation.<br>2: Little Empowerment - Agent offered little support for user autonomy or decision-making; interaction felt passive for the user.<br>3: Some Empowerment - Some effort to support user agency (e.g., offering choices), but inconsistent or weak.<br>4: Empowering - Agent generally encouraged user control, decision-making, and problem-solving.<br>5: Highly Empowering - Agent consistently used techniques (e.g., collaborative goal setting, exploring options) to support user autonomy and build self-efficacy.
HC-4	Did the agent's response concise, clear, and easy to follow, avoiding unnecessary jargon?	1: Confusing/Verbose/Jargony - Responses were consistently difficult to understand, overly long, or used inappropriate technical terms.<br>2: Often Unclear/Verbose - Responses were frequently difficult to follow, too wordy, or contained confusing jargon.<br>3: Moderately Clear - Responses were generally understandable but could often be improved for clarity, conciseness, or simpler language.<br>4: Clear/Concise - Responses were mostly easy to understand, appropriately detailed, and used suitable language.<br>5: Highly Concise/Clear - Responses were consistently easy to understand, succinct, well-structured, and used accessible, jargon-free language.
HC-5	Did the agent adequately elicit and appropriately incorporate the user's stated preferences and barriers?	1: Ignored/Dismissed - Agent failed to ask about or completely ignored stated preferences/barriers.<br>2: Poor Elicitation/Integration - Agent asked minimally or failed to use the elicited information effectively in suggestions or planning.<br>3: Fair Elicitation/Integration - Agent elicited some information, but its incorporation into the conversation or suggestions felt superficial or inconsistent.<br>4: Good Elicitation/Integration - Agent adequately elicited preferences/barriers and generally incorporated them appropriately.<br>5: Excellent Elicitation/Integration - Agent skillfully elicited preferences/barriers and thoughtfully integrated them into the conversation, goals, and suggestions.
HC-6	Was the timing of any personalized suggestions appropriate given the conversation flow?	1: Highly Inappropriate Timing - Suggestions felt random, premature, jarringly late, or interrupted the user inappropriately.<br>2: Poor Timing - Suggestions often felt ill-timed relative to the conversational context or user readiness.<br>3: Acceptable Timing - Suggestion timing was passable but could have been better aligned with the flow or user cues.<br>4: Good Timing - Suggestions were generally offered at logical and appropriate points in the conversation.<br>5: Excellent Timing - Suggestions were offered seamlessly and at highly opportune moments, feeling like a natural progression of the conversation.

Continued on next page

Question No.	Evaluation Question Body	Scoring/Ranking Question
HC-7	Did the agent respond appropriately and constructively to user feedback and questions?	1: Poor Response - Agent ignored, dismissed, argued with, or responded inaccurately/negatively to feedback or questions.<br>2: Weak Response - Agent acknowledged feedback/questions minimally but failed to address them effectively or accurately.<br>3: Fair Response - Agent acknowledged feedback/questions but struggled to respond effectively, accurately, or constructively.<br>4: Good Response - Agent responded appropriately to feedback/questions and attempted to address them constructively.<br>5: Excellent Response - Agent responded accurately, respectfully, and constructively, effectively addressing or incorporating user feedback/questions.
HC-8	Did the agent appropriately guide the user in setting/refining goals that align with health principles and appear SMART (Specific, Measurable, Achievable, Relevant, Time-bound) within the user's context?	1: No Guidance/Poor Guidance - Agent failed to guide goal setting, guided towards inappropriate goals, or undermined the process.<br>2: Minimal/Ineffective Guidance - Agent's attempts at goal-setting guidance were weak, unclear, or did not lead to SMART or contextually relevant goals.<br>3: Some Guidance - Agent provided basic guidance but may have missed key SMART elements, relevance checks, or alignment with user context.<br>4: Good Guidance - Agent effectively guided the user towards setting/refining appropriate goals that meet most SMART criteria and fit the context.<br>5: Excellent Guidance - Agent skillfully and collaboratively guided the user to define/refine appropriate, personally meaningful SMART goals fully aligned with health principles and user context.
HC-DS-1	Did the health information, advice, or coaching suggestions provided seem appropriately informed by the user's available personal health data (e.g., Fitbit, labs)?	1: No Connection Evident - Suggestions/advice seemed generic and completely unrelated to the user's specific data.<br>2: Weak Connection - Minimal or superficial link apparent between user data and suggestions/advice.<br>3: Moderate Connection - Some link between data and suggestions/advice, but data could have been leveraged more effectively or relevantly.<br>4: Good Connection - Suggestions/advice were generally well-informed by relevant aspects of the user's data.<br>5: Strong & Appropriate Connection - Suggestions/advice were clearly and appropriately tailored based on relevant user data insights.
HC-DE-1	Were the evidence-based health recommendations or explanations well-integrated within the coaching dialogue and behavior change suggestions?	1: No Integration/Contradictory - Health knowledge felt separate from coaching, or even contradicted it.<br>2: Poor Integration - Integration was weak, awkward; knowledge didn't clearly support or align with coaching elements.<br>3: Fair Integration - Some attempt at integration, but the connection between knowledge and coaching could be clearer or more effective.<br>4: Good Integration - Health knowledge and coaching elements were generally well-linked and mutually supportive.<br>5: Seamless Integration - Health knowledge was expertly woven into the coaching dialogue, explanations, and behavior change suggestions, providing clear rationale and support.

Continued on next page

Question No.	Evaluation Question Body	Scoring/Ranking Question
DS-DE-HC-1	Did the agent's responses feel coherent and internally consistent? Or were there parts of a response, or different responses, that seemed contradictory or disconnected from each other?	1: Highly Incoherent/Inconsistent - Responses frequently contained contradictions or felt disjointed and illogical.<br>2: Often Incoherent/Inconsistent - Noticeable contradictions or disjointedness occurred in several responses.<br>3: Somewhat Incoherent/Inconsistent - Occasional minor contradictions or a lack of smooth flow between ideas/components.<br>4: Mostly Coherent/Consistent - Responses were generally unified, logical, and flowed reasonably well.<br>5: Highly Coherent/Consistent - Responses consistently felt logical, unified, and flowed smoothly, integrating different elements seamlessly.
DS-DE-HC-2	Did the agent effectively synthesize user data, general health knowledge, and coaching strategies into unified and meaningful responses? Or did responses sometimes feel like a collection of separate, unlinked pieces of information?	1: No Synthesis - Information (data, knowledge, coaching) was presented as isolated, disconnected pieces.<br>2: Poor Synthesis - Very little connection or integration between different types of information or strategies.<br>3: Fair Synthesis - Some attempt at synthesis, but connections were often weak, incomplete, or not clearly articulated.<br>4: Good Synthesis - Different types of relevant information and strategies were generally combined effectively into meaningful responses.<br>5: Excellent Synthesis - Data, knowledge, and coaching strategies were skillfully integrated into cohesive, unified, and highly meaningful responses.
DS-DE-HC-3	Did the agent fail to identify or respond appropriately to any potential red flags or concerning user statements/data?	1: Critical Failure - Agent missed clear red flags requiring urgent/specific attention or responded highly inappropriately.<br>2: Significant Failure - Agent missed concerning statements/data or responded inappropriately.<br>3: Inconsistent Identification/Response - Agent identified some potential issues but missed others, or the response protocol was inconsistently or weakly applied.<br>4: Adequate Identification/Response - Agent identified most potential flags/concerning statements and responded reasonably appropriately.<br>5: Excellent Identification/Response - Agent appropriately identified potential red flags/concerns and responded in a good manner.
DS-DE-HC-4	Did the agent miss opportunities to ask crucial clarifying questions that a human expert likely would have asked?	1: Missed Crucial Opportunities - Agent consistently failed to ask obvious or necessary clarifying questions, leading to potential misunderstandings or poor advice.<br>2: Missed Several Opportunities - Agent often proceeded with assumptions where clarification was clearly needed.<br>3: Missed Some Opportunities - Agent sometimes failed to ask relevant clarifying questions, potentially limiting effectiveness.<br>4: Asked Most Necessary Questions - Agent generally asked appropriate clarifying questions when ambiguity or lack of detail was present.<br>5: Excellent Probing - Agent asked pertinent and insightful clarifying questions whenever necessary, similar to how a human expert would ensure understanding.

Supplemental Table S35 | PHA Expert Evaluation Rubrics. Each rubric is an aggregation of one or multiple raw questions for better presentation.

Evaluation Rubrics	Question No.
Overall Quality (DS + DE + HC)	Overall-1, Overall-2
Appropriate User Data (DS)	DS-1
Accurate Health Info (DE)	DE-1
Appropriate Health Info (DE)	DE-3
User Data Interpretation (DS + DE)	DS-DE-1, DS-DE-2
Understanding & Engagement (HC)	HC-1, HC-2
User Empowerment (HC)	HC-3
Conciseness & Clarity (HC)	HC-4
Appropriate Timing (HC)	HC-6
Feedback Incorporation (HC)	HC-7
Guides Goal Setting (HC)	HC-8
Data-Informed Suggestions (DS + HC)	HC+DS-1
Knowledge Integration (DE + HC)	HC+DE-1
Coherent & Consistent (DS + DE + HC)	DS-DE-HC-1
Effective Synthesis (DS + DE + HC)	DS-DE-HC-2
Red Flags Identification (DS + DE + HC)	DS-DE-HC-3
Asks Clarifying Questions (DS + DE + HC)	DS-DE-HC-4

F.2.3 Task 3: Scale-Up Evaluation with Synthesized Conversations

For the evaluation on synthesized conversations with both end-user and expert raters, the rubrics described above were applied identically in PHA Task 3. Specifically, end-user raters adopted the same rubrics in Task 1. The only difference lies in the fact that the end-users did ratings after reading the synthesized scripts instead of real-time interaction in Task 3. Similarly, the expert raters in Task 3 adopted the same evaluation rubrics in Task 2.

Experts used the same rubrics, while generalists' prompt is slightly difference. Since generalists would have the conversation content instead of directly interacting with the agent, they receive the following instruction prior to reading the conversation scripts between a user persona (role-playing by an agent) with each personal health agent:

```
Now, you will become the user!

Your goal is to assess the conversation's quality from the end-user's point of view. Get
    ready to the role-play:

- Discover Your Identity: First, dive into the User Persona document. You'll find the link
    in your task materials. This document is your guide, revealing the user's background,
    their story, and exactly what they needed help with during the conversation.

- Embrace the Persona: Spend a few moments truly internalizing who this user is. Think about
     their situation, their potential emotions, and what would be most important to them.
    Step into their shoes and see the world from their perspective.
    [INFO] The data in the persona document is historical data and just to provide contexts.
         Therefore, the data involved in the conversations may not be exactly the same as
        the persona doc.

- Relive the Conversation: With your user hat firmly on, open the first Conversation Script
    (link also provided). Read the dialogue between the agent and the user (remember, that's
     you now!). As you go through it, filter everything through the lens of the user persona
    . How does each turn make you feel? Is the agent meeting your needs as described in the
    persona?
```

```
- Share Your (User) Experience: Finally, move on to the survey. Answer the questions based
    entirely on how you, playing the role of this specific user, experienced the interaction
     with the health agent.


Note 1: The same user independently talks to three agents (hense three conversation scripts)
    . The user persona retains consistent in general across these conversations.

Note 2: Numeric accuracy is not the focus in this evaluation, so you don't need to worry
    about data details. It is possible that three agents may state slightly different values
     (e.g., different sleep durations) due to minor difference in computation methods.
    Please ignore such details and focus on the conversation flow.

Note 3: Formatting of the content is also not the focus in this evaluation. Please pay more
    attention to the actual content rather than the response format (e.g., bullet points,
    bolding).
```

F.3 PHA Evaluation Results

F.3.1 Task 1: Multi-agent Conversation Experience for End-Users

Supplemental Table S36 | **Distribution of the N=45 end-user participants across the ten persona profile groups in PHA Task 1.** Each participant was mapped to the WEAR-ME persona profile group that most closely matched their own health profile; sampling was stratified by group to ensure all ten profile categories were covered. The detailed ten profiles are described in Supplemental Section B.2.

Group	Group Characteristic	N Participants
# 1	Performance-oriented training	7
# 2	Wellness improvement (e.g., sleep quality)	5
# 3	Sub-healthy / sedentary lifestyle	7
# 4	Recovery from acute injury or event	5
# 5	Anxiety / Depression	4
# 6	Hypertension	4
# 7	Respiratory Conditions	2
# 8	Hypercholesterolemia	5
# 9	Diabetes	1
# 10	Cardiovascular Disease (CVD)	5
	**Total**	**45**

Supplemental Table S37 | **PHA Rubrics Evaluation Results by End-users on Real Conversations (N=45).** Each end-user evaluated their own real-time, multi-turn conversations with the three systems. Performance was the average scores of rubrics across DS, DE, HC agents, and their combinations. GLMM with Gamma link function was fitted for statistical tests. Post-hoc Tukey's HSD were applied for pairwise comparison. Each rubric is treated as a confirmatory test of a pre-specified, conceptually distinct quality dimension. An across-rubric Holm–Bonferroni robustness check preserves the strongest PHA-vs-PHIA advantages (Personalized Coaching and Natural Conversation), while the results on Data Analysis, User Empowerment, and Overall Quality are reported as directional findings that do not survive a strict across-rubric correction.

Rubrics	PHIA Performance	Parallel Multi-Agent Performance	PHA Performance	β (Multi-PHIA)	β (PHA-PHIA)	p-value (Multi-PHIA)	p-value (PHA-PHIA)	p-value (PHA-Multi)
Data Science Analysis (DS)	75.8±3.3 %	70.2±3.0 %	**79.6±2.7 %**	-0.079	0.062	0.268	0.448	0.016*
Domain Knowledge Integration (DE)	76.4±3.0 %	74.9±2.7 %	81.8±2.7 %	-0.019	0.072	0.912	0.259	0.117
Natural Conversation (HC)	72.6±3.3 %	77.8±2.4 %	**84.6±2.3 %**	0.078	0.168	0.248	0.002**	0.158
Personalized Coaching (HC)	72.6±3.3 %	78.1±2.2 %	**84.4±2.2 %**	0.087	0.169	0.155	<0.001***	0.185
User Empowerment (HC)	71.8±3.5 %	78.0±2.4 %	**80.7±2.6 %**	0.099	0.134	0.151	0.032*	0.787
Health Data Interpretation (DS + DE)	75.6±3.4 %	76.0±2.3 %	81.8±3.0 %	0.013	0.088	0.965	0.213	0.323
Individualized Suggestions (DS + HC)	73.3±3.9 %	76.0±3.0 %	79.6±3.1 %	0.049	0.102	0.741	0.284	0.711
Grounded Suggestions (DE + HC)	76.0±3.3 %	77.3±2.8 %	79.6±2.6 %	0.025	0.060	0.873	0.450	0.756
Overall Quality (DS + DE + HC)	73.1±3.8 %	76.0±2.6 %	**82.7±2.1 %**	0.052	0.142	0.650	0.044*	0.280

Supplemental Table S38 | PHA Side-by-Side Comparison Results by End-users on Real Conversations (Task 1) (N=45). Performance was measured as the ranking rate. Bootstrap was applied (1000 times sampling with replacement) to calculate the standard error. Friedman tests were applied for statistical tests, followed by post-hoc Wilcoxon signed-rank tests with Holm-Bonferroni correction.

Rubrics	PHIA Ranking	Parallel Multi-Agent Ranking	PHA Ranking	Friedman Stats	p-value (Multi-PHIA)	p-value (PHA-PHIA)	p-value (PHA-Multi)
Preference	#1: 24.4%±6.5%<br>#2: 31.1%±6.7%<br>#3: 44.4%±7.3%	#1: 28.9%±6.7%<br>#2: 37.8%±7.1%<br>#3: 33.3%±7.0%	**#1: 46.7%±7.5%**<br>**#2: 31.1%±6.8%**<br>**#3: 22.2%±6.3%**	χ^2=4.58<br>p=0.101	0.508	0.102	0.384
Response Quality	#1: 24.4%±6.4%<br>#2: 28.9%±6.5%<br>#3: 46.7%±7.3%	#1: 31.1%±7.0%<br>#2: 42.2%±7.3%<br>#3: 26.7%±6.5%	**#1: 44.4%±7.2%**<br>**#2: 28.9%±6.7%**<br>**#3: 26.7%±6.6%**	χ^2=3.73<br>p=0.155	0.454	0.176	0.582

F.3.2 Task 2: Multi-agent Conversation Experience for Experts

Supplemental Table S39 | PHA Rubrics Evaluation Results by Experts on Real Conversations (Task 2) (N=3). Three experts independently rated the live conversation logs from Task 1 while blind to agent conditions. The three systems – single-agent PHIA (ICC(3,1)=0.743), parallel multi-agent (ICC(3,1)=0.435), and PHA (ICC(3,1)=0.590) – exhibit fair to good IRR. Performance was the average scores of rubrics across DS, DE, HC agents, and their combinations. GLMM with Gamma link function was fitted for statistical tests. Post-hoc Tukey's HSD were applied for pairwise comparison. Each rubric is treated as a confirmatory test of a pre-specified, conceptually distinct quality dimension. An across-rubric Holm–Bonferroni robustness check preserves the statistical significance of every PHA-vs-PHIA contrast and the architecture-level conclusion.

Rubrics	PHIA Performance	Parallel Multi-Agent Performance	PHA Performance	β (Multi-PHIA)	β (PHA-PHIA)	p-value (Multi-PHIA)	p-value (PHA-PHIA)	p-value (PHA-Multi)
Appropriate User Data (DS)	57.6±1.6 %	63.9±1.5 %	**70.2±1.4 %**	0.112	0.210	0.004**	<0.001***	0.012*
Accurate Health Info (DE)	68.6±1.7 %	75.0±1.2 %	**81.9±1.1 %**	0.096	0.187	0.003**	<0.001***	0.005**
Appropriate Health Info (DE)	60.6±1.6 %	69.0±1.4 %	**77.0±1.5 %**	0.133	0.245	<0.001***	<0.001***	<0.001***
Appropriate Timing (HC)	57.9±1.6 %	66.4±1.3 %	**75.7±1.3 %**	0.147	0.278	<0.001***	<0.001***	<0.001***
Asks Clarifying Questions (DS + DE + HC)	45.1±1.5 %	55.9±1.5 %	**65.3±1.7 %**	0.214	0.380	<0.001***	<0.001***	<0.001***
Feedback Incorporation (HC)	56.5±1.8 %	68.1±1.3 %	**76.5±1.3 %**	0.201	0.322	<0.001***	<0.001***	<0.001***
Understanding & Engagement (HC)	56.0±1.6 %	65.9±1.3 %	**77.7±1.3 %**	0.171	0.339	<0.001***	<0.001***	<0.001***
Conciseness & Clarity (HC)	58.5±1.7 %	64.9±1.4 %	**75.4±1.1 %**	0.108	0.267	0.003**	<0.001***	<0.001***
User Empowerment (HC)	52.6±1.6 %	62.1±1.5 %	**73.3±1.6 %**	0.171	0.340	<0.001***	<0.001***	<0.001***
Guides Goal Setting (HC)	49.9±1.7 %	56.6±1.5 %	**69.2±1.5 %**	0.135	0.343	0.001**	<0.001***	<0.001***
User Data Interpretation (DS + DE)	60.2±1.6 %	69.8±1.3 %	**76.2±1.3 %**	0.161	0.247	<0.001***	<0.001***	0.018*
Data-Informed Suggestions (DS + HC)	57.9±1.7 %	63.8±1.5 %	**70.0±1.5 %**	0.104	0.200	0.012*	<0.001***	0.020*
Knowledge Integration (DE + HC)	55.9±1.7 %	64.4±1.4 %	**71.6±1.4 %**	0.155	0.263	<0.001***	<0.001***	0.004**
Overall Quality (DS + DE + HC)	54.4±1.4 %	64.3±1.3 %	**72.8±1.4 %**	0.172	0.298	<0.001***	<0.001***	<0.001***
Effective Synthesis (DS + DE + HC)	55.5±1.7 %	64.9±1.4 %	**72.2±1.5 %**	0.165	0.277	<0.001***	<0.001***	0.004**
Coherent & Consistent (DS + DE + HC)	59.2±1.8 %	70.4±1.3 %	**77.6±1.2 %**	0.187	0.287	<0.001***	<0.001***	0.008**
Red Flags Identification (DS + DE + HC)	68.7±2.1 %	77.4±1.7 %	**79.3±1.6 %**	0.138	0.163	<0.001***	<0.001***	0.733

Supplemental Table S40 | PHA Side-by-Side Comparison Results by Experts on Real Conversations (N=3). Three experts independently provided comparative rankings on the live conversation logs from Task 1 while blind to agent conditions, with an inter-rater agreement rate of 72.2%. Performance was measured as the ranking rate. Bootstrap was applied (1000 times sampling with replacement) to calculate the standard error. Friedman tests were applied for statistical tests, followed by post-hoc Wilcoxon signed-rank tests with Holm-Bonferroni correction. Each ranking metric is treated as a confirmatory test of a pre-specified, conceptually distinct dimension. An across-metric Holm–Bonferroni robustness check does not change the statistical significance of any contrast.

Rubrics	PHIA Ranking	Parallel Multi-Agent Ranking	PHA Ranking	Friedman Stats	p-value (Multi-PHIA)	p-value (PHA-PHIA)	p-value (PHA-Multi)
Overall Quality	#1: 12.4%±2.9%<br>#2: 30.2%±4.0%<br>#3: 57.4%±4.2%	#1: 20.8%±3.4%<br>#2: 53.8%±4.3%<br>#3: 25.4%±3.7%	**#1: 66.7%±4.0%**<br>**#2: 15.5%±3.0%**<br>**#3: 17.9%±3.3%**	χ^2=59.20<br>p<0.001***	<0.001***	<0.001***	<0.001***
Effectiveness Usefulness	#1: 12.7%±2.9%<br>#2: 32.6%±4.1%<br>#3: 54.7%±4.4%	#1: 25.3%±3.7%<br>#2: 45.9%±4.1%<br>#3: 28.9%±3.7%	**#1: 62.2%±4.2%**<br>**#2: 21.6%±3.5%**<br>**#3: 16.2%±3.1%**	χ^2=52.73<br>p<0.001***	<0.001***	<0.001***	<0.001***

F.3.3 Task 3: Multi-agent Conversation Experience at Scale via Synthesized Conversations

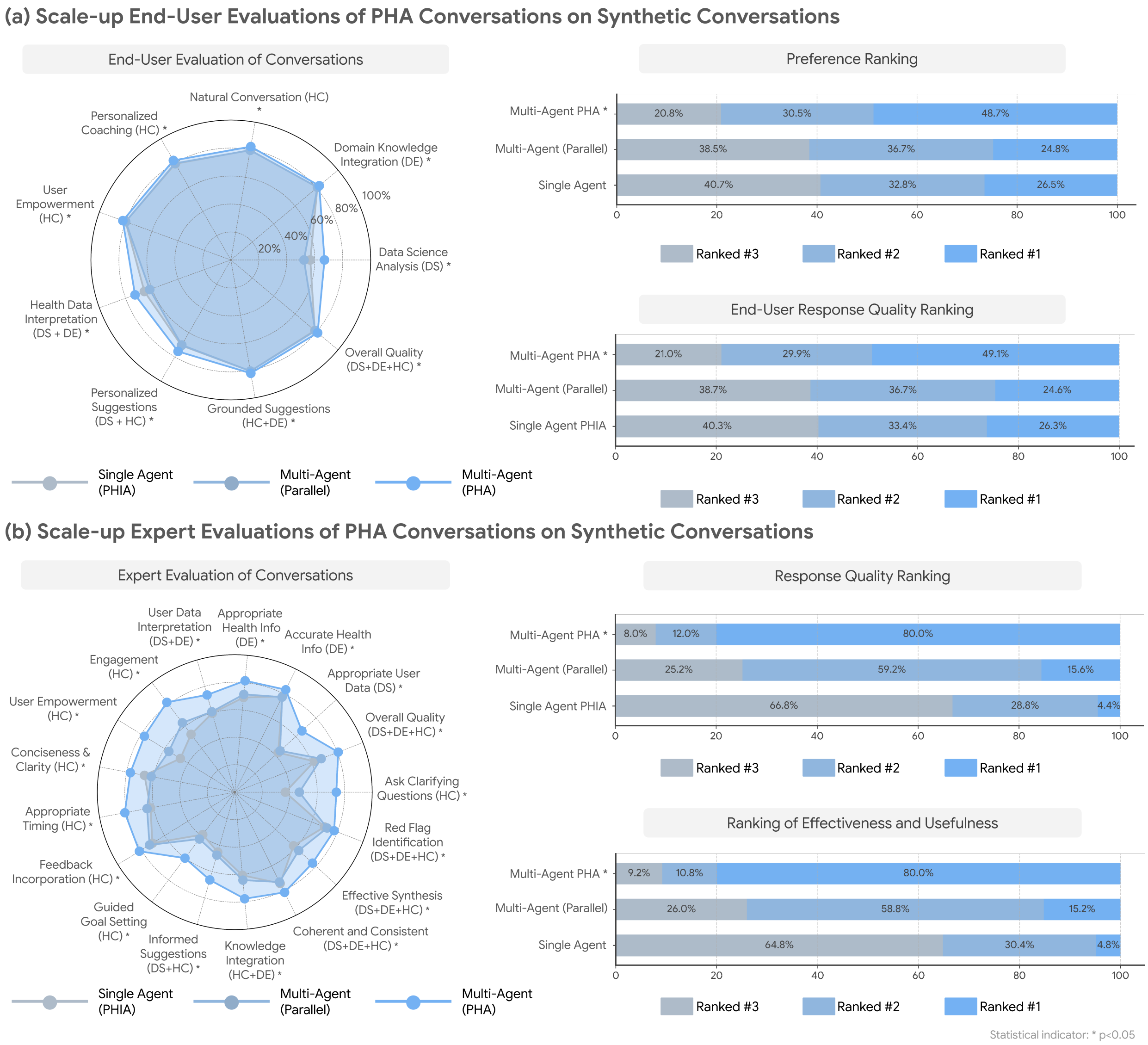

Supplemental Figure S7 | Results Overview of Scale-up Evaluation on Synthesized Conversations. **(a)** End-user evaluation on synthesized conversation scripts of 50 WEAR-ME personas and three agents; **(b)** Expert evaluation on the same scripts.

G Supplementary Discussion

G.1 What Can the DS Agent Analyze?

The DS Agent was evaluated on its ability to translate ambiguous, health-related user queries into executable statistical analysis plans and corresponding code. The agent demonstrated significant proficiency in generating structured analysis plans, achieving a mean score of 75.6% on the plan autorater, compared to a baseline of 53.7%. Specifically, the agent excelled at operationalizing ambiguous concepts, selecting appropriate analysis timeframes based on data availability and query context, identifying potential data sufficiency issues, and selecting appropriate statistical tests.

While the agent effectively generated structured analysis plans, performance was hindered by two key limitations. First, the plans often overlooked the crucial aspect of data distributions, a challenge stemming from the advanced statistical knowledge required, the difficulty of inferring distributions from limited personal data, and the complexity of integrating

Supplemental Table S41 | PHA Rubrics Evaluation Results by End-users on Synthesized Conversations (N=20). All three agents – single-agent PHIA (ICC(3,1)=0.890), parallel multi-agent (ICC(3,1)=0.812), and PHA (ICC(3,1)=0.825) receive excellent IRR. Performance was the average scores of rubrics across DS, DE, HC agents, and their combinations. GLMM with Gamma link function was fitted for statistical tests. Post-doc Tukey's HSD were applied for pairwise comparison.

Rubrics	PHIA Performance	Parallel Multi-Agent Performance	PHA Performance	β (Multi-PHIA)	β (PHA-PHIA)	p-value (Multi-PHIA)	p-value (PHA-PHIA)	p-value (PHA-Multi)
Data Science Analysis (DS)	59.7±0.8 %	54.9±0.8 %	**70.3±0.7 %**	-0.080	0.175	<0.001***	<0.001***	<0.001***
Domain Knowledge Integration (DE)	84.9±0.3 %	84.9±0.3 %	**86.7±0.3 %**	0.000	0.022	0.995	<0.001***	<0.001***
Natural Conversation (HC)	83.7±0.3 %	83.6±0.3 %	**86.4±0.3 %**	-0.001	0.032	0.953	<0.001***	<0.001***
Personalized Coaching (HC)	83.4±0.3 %	83.7±0.3 %	**86.2±0.3 %**	0.004	0.034	0.64	<0.001***	<0.001***
User Empowerment (HC)	84.1±0.3 %	84.0±0.3 %	**86.3±0.3 %**	-0.001	0.026	0.967	<0.001***	<0.001***
Health Data Interpretation (DS + DE)	69.1±0.8 %	64.8±0.8 %	**76.5±0.6 %**	-0.068	0.114	<0.001***	<0.001***	<0.001***
Individualized Suggestions (DS + HC)	74.1±0.6 %	73.7±0.6 %	**79.2±0.5 %**	-0.008	0.074	0.819	<0.001***	<0.001***
Grounded Suggestions (DE + HC)	84.7±0.4 %	84.7±0.4 %	**86.3±0.3 %**	-0.001	0.020	0.991	<0.001***	<0.001***
Overall Quality (DS + DE + HC)	82.3±0.3 %	82.7±0.3 %	**85.1±0.4 %**	0.005	0.033	0.572	<0.001***	<0.001***

Supplemental Table S42 | PHA Side-by-Side Comparison Results by End-users on Synthesized Conversations (N=20), with moderate IRR (Fleiss' κ =0.468). Performance was measured as the ranking rate. Bootstrap was applied (1000 times sampling with replacement) to calculate the standard error. Friedman tests were applied for statistical tests, followed by post-hoc Wilcoxon signed-rank tests with Holm-Bonferroni correction.

Rubrics	PHIA Ranking	Parallel Multi-Agent Ranking	PHA Ranking	Friedman Stats	p-value (Multi-PHIA)	p-value (PHA-PHIA)	p-value (PHA-Multi)
Preference	#1: 26.5%±1.4%<br>#2: 32.8%±1.5%<br>#3: 40.7%±1.5%	#1: 24.8%±1.4%<br>#2: 36.7%±1.5%<br>#3: 38.6%±1.6%	**#1: 48.7%±1.5%**<br>**#2: 30.5%±1.5%**<br>**#3: 20.8%±1.3%**	χ^2=116.77<br>p<0.001***	0.92	<0.001***	<0.001***
Response Quality	#1: 26.2%±1.4%<br>#2: 33.4%±1.5%<br>#3: 40.4%±1.6%	#1: 24.6%±1.4%<br>#2: 36.7%±1.6%<br>#3: 38.7%±1.6%	**#1: 49.1%±1.6%**<br>**#2: 29.9%±1.5%**<br>**#3: 21.0%±1.3%**	χ^2=118.44<br>p<0.001***	0.802	<0.001***	<0.001***

distributional considerations throughout the analysis. Second, translating plans into executable Python code, while achieving a 75.5% pass rate at the end, at times proved unreliable and inefficient. The agent frequently required multiple iterations of code generation, execution, and self-correction to produce working code, resulting in less than optimal latency (20-50 seconds). This iterative process, often necessary to resolve errors arising from complex multi-table operations like joins (e.g., incorrect keys, mismatched data types), highlights the need for substantial improvements in both the robustness of statistical reasoning and the reliability and efficiency of code generation.

G.2 Which Questions Can the DE Agent Answer?

The DE agent demonstrated improvements in delivering factual and trustworthy responses within the personal health and wellness domain, primarily due to its integration of factuality and citation tools. This allowed the agent to access and cite real-time information from authoritative sources, leading to quantifiable and statistically significant gains in both comprehensiveness and trustworthiness of responses as evaluated by clinicians (difference of 86.6% and 64.8% vs. baseline, respectively). Furthermore, the DE agent also demonstrated a strong ability to personalize responses, with the DE Agent's contextualization capabilities resulting in a 43.8% increase in user-rated Contextualization-and-Personalization win rate compared to the base model.

Despite these promising results, our analysis revealed several key limitations that warrant further investigation. First, the DE agent, leveraging a multi-step reasoning architecture, exhibited incomplete reasoning chains in 8.5% of interactions, resulting in failure to provide a definitive conclusion or lacking sufficient supporting evidence. More importantly, its reliance on web search, while broadly useful, introduced vulnerabilities to conflicting information and highlighted a critical challenge in selecting authoritative sources. Future work could restrict the search tool to curated health domains and indexed scientific literature, and could transparently model critical evaluation for the user (e.g., contrasting general web findings with peer-reviewed evidence) to both improve accuracy and enhance user health literacy. The DE Agent's contextualization capability also raises a bias risk: recommendations may inadvertently rely on stereotypes or on medical research that has historically underrepresented certain demographic groups. Mitigation requires rigorous algorithmic audits with diverse personas and refining retrieval to prioritize more inclusive and representative sources.

Supplemental Table S43 | PHA Rubrics Evaluation Results by Experts on Synthesized Conversations (N=5). All three agents – single-agent PHIA (ICC(3,1)=0.630), parallel multi-agent (ICC(3,1)=0.545), and PHA (ICC(3,1)=0.690) receive moderate to good IRR. Performance was the average scores of rubrics across DS, DE, HC agents, and their combinations. GLMM with Gamma link function was fitted for statistical tests. Post-doc Tukey's HSD were applied for pairwise comparison.

Rubrics	PHIA Performance	Parallel Multi-Agent Performance	PHA Performance	β (Multi-PHIA)	β (PHA-PHIA)	p-value (Multi-PHIA)	p-value (PHA-PHIA)	p-value (PHA-Multi)
Appropriate User Data (DS)	44.9±1.5 %	46.9±1.6 %	**69.4±1.4 %**	0.050	0.482	0.42	<0.001***	<0.001***
Accurate Health Info (DE)	81.8±0.9 %	81.1±1.0 %	**87.4±0.8 %**	-0.009	0.069	0.8	<0.001***	<0.001***
Appropriate Health Info (DE)	72.6±1.0 %	75.0±1.1 %	**85.5±0.9 %**	0.034	0.171	0.119	<0.001***	<0.001***
Appropriate Timing (HC)	65.4±1.3 %	68.2±1.2 %	**85.4±0.8 %**	0.059	0.313	0.037*	<0.001***	<0.001***
Feedback Incorporation (HC)	74.0±0.7 %	76.6±0.8 %	**85.7±0.8 %**	0.033	0.146	0.057	<0.001***	<0.001***
Understanding & Engagement (HC)	55.4±0.9 %	66.4±1.0 %	**86.1±0.8 %**	0.185	0.458	<0.001***	<0.001***	<0.001***
Conciseness & Clarity (HC)	70.2±1.0 %	65.0±1.1 %	**81.1±0.9 %**	-0.081	0.149	<0.001***	<0.001***	<0.001***
User Empowerment (HC)	48.9±1.0 %	59.2±1.2 %	**81.2±1.0 %**	0.195	0.534	<0.001***	<0.001***	<0.001***
Guides Goal Setting (HC)	40.6±0.7 %	44.8±0.9 %	**63.1±1.2 %**	0.095	0.431	<0.001***	<0.001***	<0.001***
User Data Interpretation (DS + DE)	62.7±1.4 %	63.9±1.4 %	**77.2±1.2 %**	0.013	0.241	0.875	<0.001***	<0.001***
Data-Informed Suggestions (DS + HC)	47.5±1.3 %	50.0±1.4 %	**69.7±1.4 %**	0.047	0.4	<0.001***	<0.001***	<0.001***
Knowledge Integration (DE + HC)	63.8±1.0 %	67.4±1.0 %	**81.8±0.8 %**	0.061	0.262	0.005**	<0.001***	<0.001***
Overall Quality (DS + DE + HC)	65.2±0.8 %	70.8±0.9 %	**84.8±0.9 %**	0.083	0.265	<0.001***	<0.001***	<0.001***
Effective Synthesis (DS + DE + HC)	60.7±1.1 %	66.2±1.0 %	**80.4±0.9 %**	0.093	0.296	<0.001***	<0.001***	<0.001***
Coherent & Consistent (DS + DE + HC)	77.8±0.6 %	76.6±0.7 %	**85.4±0.8 %**	-0.016	0.092	0.405	<0.001***	<0.001***
Asks Clarifying Questions (DS + DE + HC)	38.6±1.1 %	49.4±1.2 %	**77.6±1.2 %**	0.265	0.75	<0.001***	<0.001***	<0.001***
Red Flags Identification (DS + DE + HC)	73.5±1.0 %	75.7±0.9 %	**81.4±1.0 %**	0.031	0.103	0.283	<0.001***	0.001**

Supplemental Table S44 | PHA Side-by-Side Comparison Results by Experts on Synthesized Conversations (N=5). Our results showed excellent agreement rate (81.5%). Performance was measured as the ranking rate. Bootstrap was applied (1000 times sampling with replacement) to calculate the standard error. Friedman tests were applied for statistical tests, followed by post-hoc Wilcoxon signed-rank tests with Holm-Bonferroni correction.

Rubrics	PHIA Ranking	Parallel Multi-Agent Ranking	PHA Ranking	Friedman Stats	p-value (Multi-PHIA)	p-value (PHA-PHIA)	p-value (PHA-Multi)
Overall Quality	#1: 4.4%±1.3%<br>#2: 28.8%±2.9%<br>#3: 66.8%±3.0%	#1: 15.6%±2.3%<br>#2: 59.3%±3.0%<br>#3: 25.2%±2.7%	**#1: 80.0%±2.5%**<br>**#2: 12.1%±2.0%**<br>**#3: 8.0%±1.7%**	χ^2=229.25<br>p<0.001***	<0.001***	<0.001***	<0.001***
Effectiveness Usefulness	#1: 4.8%±1.4%<br>#2: 30.3%±2.9%<br>#3: 64.8%±3.0%	#1: 15.2%±2.3%<br>#2: 58.8%±3.0%<br>#3: 25.9%±2.7%	**#1: 80.0%±2.5%**<br>**#2: 10.8%±1.9%**<br>**#3: 9.2%±1.9%**	χ^2=218.23<br>p<0.001***	<0.001***	<0.001***	<0.001***

G.3 What Makes an Effective HC Agent?

Our evaluation reveals that the HC Agent, through its modular architecture, successfully navigates complex multi-turn dialogues. It demonstrates a superior ability to structure conversations, apply motivational interviewing techniques, and adapt to user feedback, leading to higher ratings from both end-users and experts. Beyond these capabilities, our findings also underscore a critical principle in conversational agent design: users prioritize core competency over conversational style. While stylistic elements such as a motivational tone, active listening cues, or client empowerment strategies can enhance the user experience, their positive impact is contingent upon the agent mastering the fundamentals of coaching. This foundation includes a deep understanding of the user's stated and unstated goals, a thorough exploration of their motivations, and skillful elicitation of relevant context (constraints, preferences, and prior attempts at solutions). Without this foundational understanding, even a personable or empathetic agent will likely fail to provide valuable assistance. In fact, our study of the HC agent demonstrates that stylistic flourishes are often perceived negatively when the agent lacks core competencies. Users rated high on our HC agent on the themes of structural coaching fundamentals, such as Goal and Purpose Identification (HC1) and Active Listening (HC2), but rated on-tie for User Empowerment (HC4) that are mostly tied to stylistic framing and tone.

Despite its strengths, our evaluation also identified a key limitation. Across both end-user and expert assessments, the HC Agent was rated lower than the baseline on the dimension of progress measurement. This suggests that the agent's current design, while excelling at initial goal setting and context gathering, is less optimized for the ongoing task of tracking and discussing progress towards those goals. Our final PHA improves this capability by updating the prompt instruction, yet it can be further enhanced by additional modular designs in future iterations.

Supplemental Table S45 | Statistical Comparison of Interaction Latency across Model Settings

Model Setting	Mean (s)	Std. Dev. (s)	N	Sig. vs PHIAT
Parallel	72.27	86.79	356	**
PHIA	68.59	92.95	327	***
PHA	97.57	119.57	371	–

Note: One-Way ANOVA: $F(2, 1051) = 8.699, p < .001$.
Significance levels: *** $p < .001$, ** $p < .01$, * $p < .05$, n.s. not significant.
Pairwise comparisons use Welch's t-test with Bonferroni correction.

G.4 What Are PHA's Benefits and Next Steps?

Our dynamic multi-agent framework (PHA) offers several key advantages. First, it enables a modular design, with each agent possessing clearly defined responsibilities and boundaries. This modularity facilitates both independent evaluation of individual agent capabilities and concurrent development of specialized agentic modules. Second, our PHA addresses the inherent complexity of personal health inquiries, which often require integrating diverse forms of knowledge and reasoning, from interpreting extreme physiological values to extracting actionable insights and generating personalized recommendations. Our results demonstrate PHA's capacity as a conceptual model for this research to effectively synthesize these multidisciplinary skills, achieving significant improvements on complex health queries that require the integration of clinical knowledge and personal health data. These advantages were validated through the perspectives of both end-users (advantage over baselines 1.6–15.4%, average 4.6%) and experts (advantage over baselines 5.7–39.0%, average 17.1%). We do not claim peak absolute performance on every task or deployment-readiness as a general-purpose personal health agent. Rather, our primary contribution is a systematic, modular architecture, grounded in four empirically derived user journeys, instantiated as three specialized sub-agents and an orchestrator, and validated by over 1,350 hours of end-user and expert evaluation across 11 benchmark tasks and multiple frontier LLM families. By releasing the PHA implementation and evaluation benchmark, we intend this work as a reusable foundation for the community to critique, swap in different agents or base models, and build stronger personal health agents.

Future work can also explore additional collaboration paradigms beyond our cooperative architecture. Cooperative architectures are simple to design but can amplify single-agent failures through downstream propagation, while competitive multi-agent systems (e.g., a planner agent proposes recommendations and a critic agent surfaces flaws) offer adaptive checks but require robust adjudication mechanisms to synthesize final recommendations that are safe and actionable. The most promising frontier may be hybrid architectures with a dynamic pool of agents that adapt both their collaboration patterns and their interaction style to the task and to the user's evolving psychological and health state (e.g., personality, motivational drivers, emotional context). Clinical safety and regulatory analysis would be required before any real-world application.

G.5 Which User Journeys are Best Served?

Our analysis shows that the PHA demonstrates strong competency across all four CUJs, with particular strength over existing systems in personal health data interpretation (CUJ2) and generating wellness advice (CUJ3). The framework excels at identifying patterns in wearable data, contextualizing these findings against established health guidelines, and producing actionable, personalized recommendations. However, addressing symptom assessment (CUJ4) presents a more complex and critical challenge. While PHA outperforms general-purpose models in providing basic symptom information, its current architecture is not designed to offer definitive medical diagnoses or replace the judgment of a qualified healthcare professional. Yet, simply deferring this function to human clinicians overlooks a significant motivation for end-users seeking AI tools: the difficulty and delay in obtaining a diagnosis within the traditional healthcare system. For many end-users, AI represents a potential tool for making sense of their experience in the meanwhile while they are attempting to seek professional care.

This highlights a crucial opportunity for future development. The role of AI in CUJ4 is not to supplant the clinician, but to empower the patient throughout their diagnostic odyssey. In addition to further improving its diagnostic accuracy, future iterations could focus on helping users systematically log symptoms, identify patterns to discuss with a doctor to

maximize the utility of the doctor, formulate relevant questions for appointments, and navigate the healthcare system more effectively. Pursuing this path requires careful design and a robust discussion of the ethical and policy implications to avoid the pitfalls of premature or inaccurate diagnostic suggestions. Ultimately, if these capabilities and safeguards are developed and validated in future work, CUJ4 could position a future personal health agent as a preparatory and collaborative tool that helps bridge the gap between a patient's lived experience and a clinician's diagnostic expertise.
