# Supplementary material for: The anatomy of a personal health agent
Source: Nature. 2026 Oct 21;659(8139):197–207. doi: 10.1038/s41586-026-11161-8 (PMC13736220; doi:10.1038/s41586-026-11161-8)
Supplement: Supplementary file 2 [file 41586_2026_11161_MOESM2_ESM.pdf]

nature portfolio

Corresponding author(s): A. Ali Heydari (aliheydari@google.com)
Orson Xu (orson@google.com)

Last updated by author(s): Aug 24, 2026

Reporting Summary

Nature Portfolio wishes to improve the reproducibility of the work that we publish. This form provides structure for consistency and transparency in reporting. For further information on Nature Portfolio policies, see our Editorial Policies and the Editorial Policy Checklist.

Statistics

For all statistical analyses, confirm that the following items are present in the figure legend, table legend, main text, or Methods section.

n/a	Confirmed	
☐	☒	The exact sample size (*n*) for each experimental group/condition, given as a discrete number and unit of measurement
☐	☒	A statement on whether measurements were taken from distinct samples or whether the same sample was measured repeatedly
☐	☒	The statistical test(s) used AND whether they are one- or two-sided<br>*Only common tests should be described solely by name; describe more complex techniques in the Methods section.*
☐	☒	A description of all covariates tested
☐	☒	A description of any assumptions or corrections, such as tests of normality and adjustment for multiple comparisons
☐	☒	A full description of the statistical parameters including central tendency (e.g. means) or other basic estimates (e.g. regression coefficient) AND variation (e.g. standard deviation) or associated estimates of uncertainty (e.g. confidence intervals)
☐	☒	For null hypothesis testing, the test statistic (e.g. F, t, r) with confidence intervals, effect sizes, degrees of freedom and P value noted<br>*Give P values as exact values whenever suitable.*
☒	☐	For Bayesian analysis, information on the choice of priors and Markov chain Monte Carlo settings
☐	☒	For hierarchical and complex designs, identification of the appropriate level for tests and full reporting of outcomes
☐	☒	Estimates of effect sizes (e.g. Cohen's *d*, Pearson's *r*), indicating how they were calculated

Our web collection on statistics for biologists contains articles on many of the points above.

Software and code

Policy information about availability of computer code

Data collection	Data collection was conducted through Google's Human Research Lab via IRB-approved studies (IRB Protocol: #20244970). User interaction data was collected via the Insights Explorer feature on Fitbit Labs. All other data used were from published sources, with references included in our manuscript.
Data analysis	Python (3.13), NumPy (2.4.6), Pandas (2.3.3), Seaborn (0.13.2), Matplotlib (3.10.6), R (4.5.0). AI-driven analyses utilized the Gemini family, along with comparative models (GPT-4/GPT-4o/GPT-5-mini, Claude Sonnet 4.5). The Personal Health Agent (PHA) multi-agent framework codebase, including orchestrator logic and sub-agent prompting, is open-sourced under an Apache 2.0 license.

For manuscripts utilizing custom algorithms or software that are central to the research but not yet described in published literature, software must be made available to editors and reviewers. We strongly encourage code deposition in a community repository (e.g. GitHub). See the Nature Portfolio guidelines for submitting code & software for further information.

Data

Policy information about availability of data

All manuscripts must include a data availability statement. This statement should provide the following information, where applicable:

- Accession codes, unique identifiers, or web links for publicly available datasets
- A description of any restrictions on data availability
- For clinical datasets or third party data, please ensure that the statement adheres to our policy

All study benchmark tasks, data, and results are available through our public GitHub repository(https://github.com/Google-Health/consumer-health-research/tree/main/personal_health_agent). The multiple-choice question banks used for DE Agent Task 1 are available from their respective sources as cited (StatPearls Endocrinology, BoardVitals Cardiology, Khasentino et al. 2025). The self-reported symptom dataset used for DE Agent Task 2 was collected under IRB #GH-SCD-001; a de-identified release of this dataset, consistent with original consent terms, is available at the same repository. The 50-persona evaluation subset of the WEAR-ME dataset underlying the end-user and expert studies is released under a non-restrictive research-use license at the repository. Researchers seeking access to the full WEAR-ME dataset (N=1,165) must complete the Insulin Resistance Dataset Access Request Form (https://docs.google.com/forms/d/e/1FAIpQLSebcfCZMQKBua7QS9MSvqr9n-fqmfJvmU4blOHXP1WZC0NFqA/viewform) as instructed by Metwally et al. (Nature, 2026).

Research involving human participants, their data, or biological material

Policy information about studies with human participants or human data. See also policy information about sex, gender (identity/presentation), and sexual orientation and race, ethnicity and racism.

Reporting on sex and gender	Self-reported gender was used in sample processing for persona generation and matching with real participants to ensure a representative cohort (e.g., spanning both male and female users in user studies and persona profiles).
Reporting on race, ethnicity, or other socially relevant groupings	Self-reported demographic information, including race/ethnicity, age, and existing health conditions, was utilized to capture contextualization and personalization performance across different groups (e.g., DE Agent Task 3).
Population characteristics	We provide detailed population characteristics (e.g., age, gender, employment status, health conditions) for various participants and personas in the corresponding sections and supplementary tables (e.g., Supplemental Table S35) of the manuscript. The profiles covered individuals ranging from performance-oriented athletes to those managing chronic conditions (hypertension, diabetes, CVD).
Recruitment	Participants were recruited through Google's Human Research Lab, Fitbit Labs (voluntary opt-in), and specific expert networks (for clinicians, data scientists, and health coaches). Complete recruitment details and inclusion criteria for both end-users and expert raters are provided in the Methods section.
Ethics oversight	The WEAR-ME study was approved by the Advarra Institutional Review Board (IRB, #Pro 00074093). The self-reported symptom dataset was collected under IRB #GH-SCD-001. Human evaluations and live interactions were conducted through Google's Human Research Lab via IRB-approved studies (IRB Protocol: #20244970).

Note that full information on the approval of the study protocol must also be provided in the manuscript.

Field-specific reporting

Please select the one below that is the best fit for your research. If you are not sure, read the appropriate sections before making your selection.

☐ Life sciences ☒ Behavioural & social sciences ☐ Ecological, evolutionary & environmental sciences

For a reference copy of the document with all sections, see nature.com/documents/nr-reporting-summary-flat.pdf

Behavioural & social sciences study design

All studies must disclose on these points even when the disclosure is negative.

Study description	A mixed-methods study evaluating the Personal Health Agent (PHA) framework. It involves (1) user-centered design via surveys (N=555) and expert workshops (N=14); and (2) empirical evaluation of agent capabilities across 11 benchmark tasks using both automated metrics and extensive human evaluation. Human evaluations involved single-turn Q&A, comprehensive health summaries, multi-turn coaching, and full multi-agent conversations, assessed quantitatively and qualitatively by both end-users and domain experts. Additionally, a retrospective observational study on diagnostic conversations (N=2,000) was conducted.
Research sample	The sample encompasses diverse adult end-users (consumers) and subject-matter experts. End-user cohorts included: 555 Fitbit Labs survey participants, 17 users for DE Agent Q&A, 2,000 consumers for the diagnostic study, 31 users for HC Agent evaluation, 45 users for live PHA evaluation, and 20 users for synthesized PHA evaluation. End-users spanned ages 18–67 with diverse health profiles. Expert cohorts included: 14 UX/engineering experts, 10 data scientists for analysis planning, 7 data scientists for code generation, 5 clinicians for DE summaries, 3 health coaches for HC evaluation, and 8 experts for PHA evaluations. Experts held advanced degrees with at least 5 years of professional experience.

Sampling strategy	For the live PHA evaluation, 45 end-users were sampled and stratified by diverse health conditions to ensure comprehensive coverage across 10 specific health profiles (matched to 50 WEAR-ME personas). For all human evaluations, while no statistical methods were used to pre-determine sample size, the number of participants were chosen to be comparable to or exceed those in contemporary HCI/AI health agent literature to provide sufficient statistical power for detecting differences in subjective ratings using generalized linear mixed models (GLMMs) and non-parametric tests.
Data collection	Data was collected via digital logs of agent-user interactions, side-by-side preference rankings, and structured questionnaires (Chatbot Usability Questionnaire, Working Alliance Inventory, Client Evaluation of Motivational Interviewing, SMART goal setting, and AI Coaching Metrics). Clinical diagnostic data was collected via an online survey capturing prior health events and symptoms. Interactions in live human evaluations were conducted via a custom online chat interface.
Timing	Consumer queries were collected from January 2024 to February 2025. Insights Explorer Fitbit Labs data was collected from October 2024 to February 2025. Retrospective symptom data was based on events occurring within the prior 3 months of the survey. Human evaluation studies were conducted concurrently with manuscript preparation.
Data exclusions	These exclusion criteria were pre-established prior to data collection and analysis. For WEAR-ME data, quality control criteria were applied, excluding users with abnormally high BMI (BMI > 65), as well as short or unreliable wearing periods or high missing data rates to ensure validity.
Non-participation	Participation in all surveys and lab studies was entirely voluntary (e.g., Insights Explorer required explicit opt-in and in-app consent). Non-participation did not affect the core analyses as the required sample targets for the evaluations were successfully met.
Randomization	In comparative human evaluations, agent conditions (baseline vs. proposed framework) were presented to participants in a randomized, counterbalanced order using a Latin-square design. Participants and expert raters were strictly blinded to the agent condition to mitigate bias. Order of presentation was controlled to avoid sequence effects.

Reporting for specific materials, systems and methods

We require information from authors about some types of materials, experimental systems and methods used in many studies. Here, indicate whether each material, system or method listed is relevant to your study. If you are not sure if a list item applies to your research, read the appropriate section before selecting a response.

Materials & experimental systems

n/a	Involved in the study	
☒	☐	Antibodies
☒	☐	Eukaryotic cell lines
☒	☐	Palaeontology and archaeology
☒	☐	Animals and other organisms
☒	☐	Clinical data
☒	☐	Dual use research of concern
☒	☐	Plants

Methods

n/a	Involved in the study	
☒	☐	ChIP-seq
☒	☐	Flow cytometry
☒	☐	MRI-based neuroimaging

Plants

Seed stocks	N/A
Novel plant genotypes	N/A
Authentication	N/A
