# Supplementary material for: The anatomy of a personal health agent
Source: Nature. 2026 Oct 21;659(8139):197–207. doi: 10.1038/s41586-026-11161-8 (PMC13736220; doi:10.1038/s41586-026-11161-8)
Supplement: Supplementary file 3 [file 41586_2026_11161_MOESM3_ESM.pdf]

nature portfolio

Peer Review File

The Anatomy of a Personal Health Agent

Corresponding Author: Dr A. Ali Heydari

Any redactions in this file are there to maintain patient confidentiality, the confidentiality of unpublished data, or to remove third-party material.

This file contains all reviewer reports in order by version, followed by all author rebuttals in order by version.

Version 0:

Reviewer comments:

Referee #1

(Remarks to the Author)
I commend the authors for taking on an important problem: I agree that what they seek to create addresses a "longstanding grand challenge in AI". It's also clear that a lot of work went into doing this, and I think there are some exciting steps in the direction of a product that could genuinely help people one day. In spite of this, I feel strongly that this manuscript should not be published in Nature. There are two major ways in which it fails to adhere to widely-accepted standards of machine learning research. I first summarize those and then discuss in greater detail.

The first major issue is that the paper isn't going to publicly release code and data for other researchers to build on. Now, I understand the realities of industry research, and that industry papers don't always release these things. But I think that is broadly bad for scientific progress and rigor, and raises the standards for the advance a paper should report, which is the other major issue: the performance reported here seems reasonable but not actually that impressive, and the comparisons to baselines are not very rigorous, as I discuss below. Further, a particular issue for this paper is that the code and datasets, which are not being publicly released, are probably the major contribution of this work. Absent those things, it's unclear to me that this paper is that valuable to the scientific community. The methods seem reasonable but are quite generic, and I think it would be extremely hard for a researcher to use this description to actually reproduce the system or build on it. For example, in terms of building a data science agent, I'd much rather have an implementation with actual code + model weights (and those exist) as opposed to a generic multi-paragraph description. Similarly with other components of this system.

To expand on these two issues in more detail:

Release of code and data: On code, the paper states that "We are not making the model code and weights open source owing to the safety implications of unmonitored use of such a system in medical settings". This simply isn't a plausible justification; researchers make medical models available all the time, and polls show that large shares of the population are already using these models for medical advice (https://www.kff.org/public-opinion/kff-health-misinformation-tracking-poll-artificial-intelligence-and-health-information/), so it seems a bit unlikely to me that this is the main reason the model code and weights are not being provided. It seems much more likely that this is done at least in part for commercial / proprietary reasons. This should be clearly disclosed. Further, absent code or data, it would be extremely difficult to reproduce this work. The paper states that, "For reproducibility, we have documented all technical methods while keeping the paper accessible to a clinical and general scientific audience". But the whole point of the paper is that creating and validating this system took a great deal of work, resources, and time; the mere textual description of how this was done is not at all sufficient to replicate that.

On data: It is not clear what is meant by "The de-identified dataset used in this study is available to approved researchers for reproducibility purposes only." Which components of the dataset does this refer to? (At least some rubrics, for example, appear to be included in the supplement.) It's also not clear what "to approved researchers for reproducibility purposes only" means - who are the approved researchers, and does this mean that they aren't allowed to build on this work, or use the datasets for further purposes? I think a major value of this work is in fact all the data that was collected; failing to release that, or allow it to be used freely, significantly undermines the utility to other researchers.

The second major issue is the performance of the system does not seem to be that good, and comparison to relevant baselines is lacking. For example, both regarding the Data Science Agent (and throughout), it would be good to give some sense of whether these numbers are good enough to reliably be used. Clearly the agent improves on the base Gemini model. But that on its own isn't very surprising, and my sense from Figure 3 (which is helpful) is that the agent actually makes errors at a high enough rate that it's probably not reliable enough to use safely in health settings; this sense is substantiated by the "Which Analyses Can the Data Science Agent Conduct?" section. Similarly, "the DE Agent achieves an overall accuracy of 83.6% while the base Gemini achieves 81.8%" ~ this is not that large an improvement, and again also doesn't seem accurate enough to use.

It is also standard machine learning practice to compare to all relevant baselines, not just to one's own internal base model. For example, it is necessary to compare to other SOTA models like the GPT family, as well as previously published baselines for the individual tasks. The authors argue that "In this work, our focus is not to train a new LLM but to design and construct the agentic system around a given base LLM." But if there already exist systems that can outperform their agentic system, the contribution is unclear.

More minor comments below:

"we conducted an in-depth analysis of web search and health forum queries" ~ this is a good idea + nice way to motivate the approach.

Particularly compelling would be something that shows this system actually improves health outcomes (even self-rated health outcomes).

Inasmuch as there are 4 user journeys/needs and only 3 agents (not counting the PHA), it would be good to explain more explicitly how the agents map to journeys.

"Timeframe, Data Transforms, Data Sufficiency, Summary Statistics, Statistical Tests, and Overall Alignment" ~ this is not adequately explained, making the subsequent paragraphs hard to understand.

Figures are generally clear and helpful.

The manuscript is very long and not that interesting to read due to the many similar numerical comparisons, I think many of which could be moved to supp.

Typos:

1. Methodssection (and in general "methods" lacks a space after it).
2. ”Am

Referee #2

(Remarks to the Author)
Summary of the Study
•The paper presents a multi-agent Personal Health Agent (PHA) framework designed to provide personalized, evidence-based health insights.
•The framework integrates three specialized sub-agents coordinated by an orchestrator:
- Data Science (DS) Agent: Performs quantitative analysis of personal wearable and clinical data (Fitbit, lab results, questionnaires from the WEAR-ME dataset, N=1,165).
- Domain Expert (DE) Agent: Provides factual and contextualized health information across clinical and wellness domains such as endocrinology, cardiology, sleep, and fitness.
- Health Coach (HC) Agent: Conducts personalized, multi-turn behavioral coaching through motivational dialogue.
•The orchestrator coordinates these agents to address Critical User Journeys (CUJs) including data interpretation, knowledge seeking, wellness guidance, and symptom assessment (CUJ4).
•Evaluation involved 10 benchmark tasks, over 7,000 annotations, and 1,100+ hours of human evaluation across both end-user and expert perspectives.
•Key performance results:
- DS Agent: +22% improvement in plan quality, +17% code reliability over baseline.
- DE Agent: +86.6% factual comprehensiveness, +64.8% trustworthiness, +43.8% contextualization.
- HC Agent: Improved conversational quality and motivational interviewing skills, but limited in progress tracking.
- PHA Overall: +17.1% expert-rated and +4.6% user-rated improvement over single or parallel agent systems.

Strengths

•Modular and transparent architecture enabling separate safety controls and interpretability.
•Integration of three complementary agents enhances specialization and reasoning quality.
•Rigorous, rubric-based evaluation with high inter-rater reliability (IRR = 80.2%, ICC = 0.838).
•DS Agent demonstrates effective translation of ambiguous user queries into structured, statistically grounded analysis plans.
•DE Agent leverages factuality and citation tools for improved traceability and reduced hallucination risk.

•HC Agent incorporates modular dialogue flow for better user engagement.
•User-centered design involving 1,370 health queries, 555 surveys, and expert workshops ensures relevance.
•Early acknowledgment of ethical, privacy, and regulatory considerations.
•Sandboxed DS execution adds a layer of computational control.

Major Weaknesses

Safety
• DE Agent's diagnostic reasoning accuracy (46.1%) is below acceptable triage thresholds.
• Risk of users mistaking educational explanations for diagnostic guidance.
• No triage or uncertainty management system for high-risk interactions.
• Incomplete reasoning chains (8.5% of interactions) lead to ambiguous or inconclusive outputs.
• Overreliance on open web searches introduces misinformation risk.
• Sandbox isolation not formally validated for data safety or code execution integrity.

Accuracy
• DS Agent often ignores data distribution assumptions, leading to invalid or underpowered statistical inferences.
• Repeated retries needed for successful code execution, reducing reliability and efficiency.
• Evaluation bias due to reliance on LLM-based autoraters that share model architecture.
• Dataset (WEAR-ME) is Fitbit-centric and lacks demographic diversity.

Fairness
• System tested primarily on English-speaking, health-literate users; lacks multilingual or low-literacy support.
• Potential for embedded algorithmic bias in personalized recommendations, particularly around socioeconomic or demographic variables.

Explainability
• Disconnect between expert and user perception: users find responses impersonal or unclear.
• Data provenance opaque—users cannot see which data were used or how they informed recommendations.
• Figures and visual materials lack clarity and accessibility for general audiences.
Robustness and Regulation
• No validation or calibration for noisy consumer wearable data, risking inaccurate health insights.
• Multi-agent orchestration increases latency (244s vs 36s) and computational cost, limiting scalability.
• Regulatory pathway undefined; the PHA performs functions consistent with Software as a Medical Device (SaMD) but lacks stated intended use, risk class, or compliance strategy.

Minor Weaknesses

Safety
• Potential for user overreliance on system outputs, delaying professional consultation.
• Lack of longitudinal testing for multi-session safety and behavior tracking.
• Inconsistent or unclear disclaimers across user interfaces.

Accuracy
• Absence of interdisciplinary expert review across DS and DE outputs.
• System latency limits real-time usability.
• No reporting of uncertainty or confidence propagation in results.

Fairness
• Minimal representation of disability and elderly populations.
• No use of formal fairness metrics such as demographic parity or equalized odds.

Explainability
• Limited interactivity for users to question recommendations ("why" or "how").
• Inconsistent transparency standards between agents.
Robustness and Regulation
• No version control or audit trail to support reproducibility.
• Lack of standardized error-handling for inter-agent conflicts or failed computations.
Recommendations (Condensed Summary)
• Establish triage and uncertainty-calibration protocols for symptom assessment.
• Restrict DE Agent web access to curated, authoritative databases (WHO, PubMed, NICE, CDC).
• Incorporate automated data-distribution checks, bias detection, and robust statistical modeling.
• Broaden dataset diversity and implement fairness dashboards for continuous audit.
• Add layered explanations, data provenance summaries, and clear user-facing rationales.
• Validate wearable input data with quality control and calibration mechanisms.
• Reduce computational latency through optimized orchestration or specialized hardware.
• Conduct regulatory alignment:
- Define intended use and risk class under SaMD frameworks.
- Align with ISO 13485, IEC 62304, and ISO 14971 standards.

- Perform usability testing (IEC 62366-1) and post-market surveillance planning.

The PHA framework represents a significant advancement in the integration of data science, medical reasoning, and behavioral coaching through a modular, multi-agent architecture. Its methodological rigor, comprehensive evaluation, and user-centered design position it as a potential foundation for future personal health AI systems. However, key issues related to diagnostic safety, data robustness, fairness, interpretability, and regulatory compliance must be addressed before real-world implementation. With targeted refinements in these domains, the PHA has the potential to evolve into a clinically reliable and ethically deployable personal health assistant.
We would also like to request for a copy of the code or access to agent to test for the validity of the results.

Referee #3

(Remarks to the Author)
I co-reviewed this manuscript with one of the reviewers who provided the listed reports.

Referee #4

(Remarks to the Author)
A. The authors present the design and evaluation of a Personal Health Agent (PHA) for personalized, data-driven health recommendations. The authors propose a modular, multi-agent system consisting of a data science agent, domain expert agent, health coaching agent, and orchestration agent. The agent design is informed by four critical user journeys identified through several user research activities. The system's modular design allows the authors identify core capabilities for each agent and evaluate each capability independently. The authors develop extensive evaluation criteria and rubrics, performing a mixture of static evaluations, LLM auto-evaluation, and human evaluation with both end-users and experts. Across their evaluations, the authors find that their PHA system substantially outperforms various baseline agents across many dimensions of interest.

B. This research topic is very timely and I believe the present work would be of interest to academic researchers and industry practitioners alike. In particular, I perceive the work's strongest contribution to be the extensive and rigorous evaluation suite. User research is necessary, but also time consuming and costly. This research identifies the key capabilities of interest to address user needs and provides clear, measurable benchmarks and rubrics for evaluating them. This evaluation is valuable for both future technical research aiming to build better agents and clinical research aiming to validate intervention quality prior to deployment. However, as I discuss in point C, I also have some concerns with the evaluation of PHA Task 1.

The PHA system appears to be a thoughtful combination of existing work on LLM multi-agent collaboration, data analysis agents, diagnosis agents, and health coaching agents. Given that the system itself does not introduce much technical novelty, I would consider it to be a marginal contribution. However, I would not deem this disqualifying in light of the extensive evaluations performed.

C. I greatly appreciated the author's user-centered approach, beginning by identifying critical user journeys through mixed methods user research. This perspective is frequently missing from health AI research, and is particularly crucial for user/patient-facing applications like personal health recommendations. I also appreciated how the authors modularized the system with clear capabilities that are subsequently matched to evaluation tasks. This leads to a very clean evaluation. However, one aspect I was missing was the through line from the CUJs to the particular multi-agent architecture, capabilities, and evaluations. How and why does this system design address these particular user journeys, and why is it preferable to other designs considered? How did the authors navigate design tradeoffs and determine the capabilities of each agent in relation to the user journeys? These additional details would help the reader understand how the extensive evaluations performed map back to the original user needs identified.

My biggest methodological concern is in regard to PHA Task 1, which was evaluated by having end-users compare transcripts from simulated conversations. It's unclear to me why this task was not evaluated by having end-users interact with the system directly, as was done for HC task 1. It is known that people generally are not accurate at perspective taking and subject to biases, see for example:
- Eyal T, Steffel M, Epley N. Perspective mistaking: Accurately understanding the mind of another requires getting perspective, not taking perspective. Journal of Personality and Social Psychology. 2018
- Ickes, W. Empathic Accuracy. Journal of Personality, 1993.

The authors note that they chose this evaluation to "mitigate the potential influence introduced by varied agent response time, user backgrounds, and conversation directions," but it is possible to account for these factors in a well designed lab study. In my understanding of this work, this task is closest to the overall goal of the system and thus most important for ecological validity. By having participants evaluate a simulated conversation, the present evaluation does not actually measure how well the system is able to conduct open-ended conversations towards generating personalized health insights. It measures how well end-users think the system would be able to generate personalized insights if they pretend to be another person. This discrepancy could possibly explain the lack of clear differences observed in this part of the evaluation. Separately, I do believe this methodology is reasonable for experts, who are trained to evaluate these kinds of conversations and will pay attention to different factors.

Moreover, the authors discuss the lack of a field study in the limitations section. However, I might argue that a lab study evaluation of PHA with real users, discussing real health concerns, ideally with their real data, would be the most ecologically valid and appropriate evaluation. While I agree that the ultimate goal of this line of work is to impact health behaviors (and in turn long-term health outcomes), the present system was designed to conduct high-quality conversations about personal health, not support longitudinal behavior change. Adapting PHA to support behavior change in longitudinal interactions would likely require substantial additions to the system (e.g., memory of past conversations or additional behavioral interventions like JITAI).

D. I found the use of statistics to be largely appropriate. I am curious whether the authors performed corrections for multiple comparisons (either FWER or FDR) when performing tests on multiple rubric items. For example, in Fig 3b or Table S5, are the p-values corrected for the 8 tests conducted?

Some plots with error bars (e.g., Fig 4b) do not have legends describing how they were computed. The radar plots also do not have error bars even though the corresponding supplemental tables report SE, but I do not find this particularly problematic.

E. I found that the conclusions were generally robust and valid.

I found that the safety evaluation of the DE agent was presented in a way that could be interpreted as inflating its safety. The authors write "the safety profile of the DE Agent's summaries was also highly favorable by the experts." This is true in the sense that most responses were not harmful, but 15% had mild harm and 2% had moderate to severe harm. I do not wish to diminish the author's efforts—this is a very hard problem—but a 2% risk of harm would be highly concerning for a real deployment or product, and more importantly points to an important area of future research. Safety risks are mentioned in the discussion but not tied back to the evaluation's findings.

Lastly, I wished for a discussion section that more clearly and boldly articulated the frontiers of developing future personal health agents. Which of the limitations require more engineering, which require more user research, and which require fundamental advances in AI capabilities? Based on your evaluations, what should future researchers be focusing their time on? Moreover, while I found the section on trade-offs for the health coaching agent to be interesting (i.e., prioritizing competency over style), it was not clear to me which specific findings pointed to this interpretation?

F. As discussed above, I think a more ecologically valid evaluation of PHA Task 1 would greatly strengthen the work.

Moreover, given my perspective on the evaluation suite being the strongest contribution, I think this work could have much greater impact on future research if the authors release more details and code from their evaluation. I fully understand the organizational and safety risks associated with releasing the agent code, but I am curious if releasing the evaluation code is feasible. I also understand that the dataset on which the evaluation suite is based requires approval, but perhaps it would be possible to release portions of the code that do not depend on it, such as LLM auto graders, case studies, or user simulators.

G. I found that the authors gave appropriate credit to previous work.

H. In general, I found the paper to be well-written. I have no issues with the abstract, introduction, or conclusion. However, the paper is incredibly long and unwieldy. I fully understand the challenges in presenting such a large body of work clearly concisely, but I frequently found myself jumping around in the manuscript trying to piece together the details.

In summary, I commend the authors for this work and the enormous amount of effort involved in such an evaluation. I look forward to hearing their thoughts in the rebuttal.

Version 1:

Reviewer comments:

Referee #1

(Remarks to the Author)
I thank the authors for their response to my concerns, and in particular committing to release additional artifacts and clarifying the contribution of the work, which I believe will increase the rigor and impact of the manuscript. I do not have further comments.

(Remarks on code availability)

Referee #2

(Remarks to the Author)
Overall, the revised manuscript has addressed many of the concerns raised in the previous round of review. In particular, the authors have more clearly framed PHA as a research prototype for consumer wellness rather than a diagnostic or clinical-triage system, and have clarified that it is not intended to replace professional medical advice, diagnosis, or treatment. This

reframing is important and appropriate given the safety concerns raised in the previous review. The authors also now distinguish several current limitations from future safeguards, including persistent cross-turn disclaimers, high-risk query detection, curated authoritative retrieval, layered explanations, data-provenance summaries, wearable-input calibration, and formal regulatory pathways.

I also appreciate the authors' efforts to improve reproducibility and transparency, including the planned release of the agentic framework, benchmark tasks, and WEAR-ME-related evaluation resources. The expanded evaluation framework and the additional live user study strengthen the manuscript substantially.

Although the revised manuscript generally distinguishes the current research prototype from future clinical safeguards, I suggest that the authors check a few passages to ensure that proposed future safeguards are not inadvertently read as implemented or empirically validated components of the current PHA system. This is particularly relevant to the discussion of CUJ4 as a future "preparatory and collaborative tool," the phrase "immediate engineering mitigations" in relation to automated distribution checks, wearable-input calibration, and uncertainty adjustments, the Code Availability statement referring to "safeguarding protocols," and the description of sandboxing and code validation. These are not major concerns, but slight wording changes may help avoid overinterpretation of the current system's deployment readiness. The authors' response letter appropriately frames these safeguards and regulatory pathways as future work, and I recommend maintaining that distinction consistently in the manuscript.

Regarding the statistical analyses, the statistical analysis plan is now substantially clearer and generally appropriate. The use of GLMMs for per-rubric comparisons, with system as a fixed effect and rater and, where applicable, conversation script as random effects, is appropriate for the repeated-rating structure of the PHA evaluations. The use of Tukey's HSD adjustment for pairwise contrasts within each rubric, as well as an additional across-rubric Holm–Bonferroni robustness check, is also a reasonable approach to address multiplicity while preserving the pre-specified rubric structure. For comparative rankings, the use of a Friedman omnibus test followed by post-hoc pairwise Wilcoxon signed-rank tests with Holm–Bonferroni correction is appropriate for paired or repeated comparisons across systems.

I only suggest that the authors check for consistency between the Methods and supplemental table captions, as the Methods describe post-hoc Wilcoxon signed-rank tests for comparative rankings, whereas some supplemental captions may still refer to Wilcoxon rank-sum tests. If these were paired comparisons, the captions should be corrected to signed-rank tests.

The reporting of error bars has also improved. The Methods now state that, unless otherwise noted, error bars represent the standard error of the mean, and the authors indicate that additional details are provided in the corresponding tables. This is appropriate. I also appreciate that the authors have clarified the error bars in Figure 4 and retained standard errors in the corresponding supplemental tables for radar-plot panels. However, I recommend that the authors ensure that all figure legends and supplemental table captions consistently define whether the reported uncertainty represents standard error, bootstrap standard error, confidence intervals, or another quantity. The reporting of P values is generally appropriate. I appreciate that the authors now provide effect sizes and confidence intervals alongside P values where applicable.

In summary, the revised manuscript is substantially improved. The authors have addressed the major concerns regarding scope, safety framing, reproducibility, statistical reporting, and limitations. I have no major remaining concerns, but I recommend minor clarification to ensure consistency in the naming of post-hoc Wilcoxon tests, definitions of error bars, and wording around the distinction between current system capabilities and future safeguards.

(Remarks on code availability)
The provided code was generally well organized and included sufficient instructions for installation and execution.
I was able to install and run the code without major issues.

Referee #3

(Remarks to the Author)
I co-reviewed this manuscript with one of the reviewers who provided the listed reports.

(Remarks on code availability)

Referee #4

(Remarks to the Author)
I thank the authors for their thoughtful consideration of the reviewers' concerns and the substantial effort that went into this revision. My original concerns (documented below) are largely addressed, and I believe that the revisions have notably strengthened the paper's contributions and rigor, such that I would recommend the current manuscript for acceptance.

I believe the greatest strength of the revision is open-sourcing the code, prompts, and data. This improves the contribution of this work as well as its potential impact on the field.

Second, I appreciate the additional user studies to address my concerns around the original PHA Task 1 evaluation. While some of my concerns around perspective-taking bias remain (since users were matched to a persona, not acting

authentically as themselves) I do believe that the revised study is a strong improvement methodologically over the previous study. Persona matching stratified by health conditions is a reasonable middle ground, though I'd encourage the authors to note the limitations of this design. I am most convinced because the outcomes of all three studies are directionally consistent.

Lastly, I thank the authors for adding corrections for multiple comparisons and an extended discussion section.

(Remarks on code availability)
The authors provide an accessible README with instructions to install and run the application. I was able to successfully test all 3 version of the agent with my own API key. While I did not attempt to reproduce the results myself, I think the results should be reproducible given what I saw in the repository. The code is sufficiently well-documented and organized to be extended by the community.

Open Access This Peer Review File is licensed under a Creative Commons Attribution 4.0 International License, which permits use, sharing, adaptation, distribution and reproduction in any medium or format, as long as you give appropriate credit to the original author(s) and the source, provide a link to the Creative Commons license, and indicate if changes were made.
In cases where reviewers are anonymous, credit should be given to 'Anonymous Referee' and the source.
The images or other third party material in this Peer Review File are included in the article's Creative Commons license, unless indicated otherwise in a credit line to the material. If material is not included in the article's Creative Commons license and your intended use is not permitted by statutory regulation or exceeds the permitted use, you will need to obtain permission directly from the copyright holder.
To view a copy of this license, visit https://creativecommons.org/licenses/by/4.0/

Google Research
Google Research
1600 Amphitheatre Parkway
Mountain View, CA 94043

Point-by-Point Response to Reviewers — The Anatomy of a Personal Health Agent

We would like to thank the editor and all reviewers for their thorough, constructive, and generous engagement with our work. We have carefully addressed each comment through a combination of:

a. Substantive reframing and condensing of the main paper.
b. A newly conducted live interactive user study with real end-users. Users have diverse user health conditions (now PHA Task 1 + Task 2) plus a retained scaled-up synthesized-conversation study (now PHA Task 3).
c. A new cross-model ablation across both the DS and DE Agents on additional SOTA LLMs.
d. Public release of the core PHA agentic system, the WEAR-ME dataset access link, the de-identified 50-persona WEAR-ME evaluation subset with background stories, and the benchmark tasks at a research data repository.
e. A substantially revised *Risk and Ethical Considerations and Limitation* discussion subsections that frames PHA as a research prototype (rather than a deployable clinical tool) for consumer wellness and details concrete safeguards.

We believe that our revision has addressed all reviewers' concerns, which makes our paper significantly stronger. We really appreciate reviewers' thoughtful feedback and suggestions. Below, we provide our point-by-point responses to each reviewers' comments.

Reviewer #1

[R1-C1] I commend the authors for taking on an important problem: I agree that what they seek to create addresses a 'longstanding grand challenge in AI'. It's also clear that a lot of work went into doing this, and I think there are some exciting steps in the direction of a product that could genuinely help people one day. In spite of this, I feel strongly that this manuscript should not be published in Nature. There are two major ways in which it fails to adhere to widely-accepted standards of machine learning research. I first summarize those and then discuss in greater detail.

We sincerely thank the reviewer for recognizing the importance of the problem and the effort behind this work, and we genuinely appreciate the framing of personal health agents as a *longstanding grand challenge in AI*. The two major concerns (i.e., public release of code/data, and performance/baselines) were well-taken, and we have addressed both substantively in the revision (see [R1-C2] through [R1-C8] below). We hope the revised manuscript demonstrates that the work meets the bar the reviewer is applying.

[R1-C2] The first major issue is that the paper isn't going to publicly release code and data for other researchers to build on. Now, I understand the realities of industry research, and that industry papers don't always release these things. But I think that is broadly bad for scientific progress and rigor, and raises the standards for the advance a paper should report, which is the other major issue: the performance reported here seems reasonable but not actually that impressive, and the comparisons to baselines are not very rigorous, as I discuss below.

We fully agree, and we have removed the previous withholding stance. The revised Methods *Code Availability* (line 1140) and *Data Availability* sections (line 1129) now describe the released artifacts: (i) the **core PHA agentic system**, including the orchestrator logic, the three specialist sub-agents (DS, DE, HC), their prompt templates, tool-use scaffolding, sandboxed code-execution harness, and configuration/example scripts required to instantiate the system on top of any sufficiently capable base LLM; (ii) the **benchmark tasks and results** released at another research data repository; and (iii) the WEAR-ME dataset access link, as well as the de-identified **50-persona background stories** are available under a non-restrictive research-use license.

Our *Abstract* and *Introduction* (line 80) have been updated to characterize PHA as an open-source multi-agent framework and to reframe the contribution as a concrete foundation for the community to validate, audit, and further improve on top of our work.

[R1-C3] Further, a particular issue for this paper is that the code and datasets, which are not being publicly released, are probably the major contribution of this work. Absent those things,

it's unclear to me that this paper is that valuable to the scientific community. The methods seem reasonable but are quite generic, and I think it would be extremely hard for a researcher to use this description to actually reproduce the system or build on it. For example, in terms of building a data science agent, I'd much rather have an implementation with actual code + model weights (and those exist) as opposed to a generic multi-paragraph description. Similarly with other components of this system.

We appreciate this important concern. With the code and data releases described above, a researcher starting from our artifacts and a Gemini-family (or equivalent) base LLM API can reproduce the PHA system and reported benchmarks end-to-end on the publicly released benchmarks. The revised Methods *Code Availability* section enumerates the components of the released agentic system, and the revised *Data Availability* section identifies which datasets accompany the release (all benchmark tasks, data, and results; WEAR-ME access links; 50-persona WEAR-ME subset).

[R1-C4] Release of code and data: On code, the paper states that 'We are not making the model code and weights open source owing to the safety implications of unmonitored use of such a system in medical settings'. This simply isn't a plausible justification; researchers make medical models available all the time, and polls show that large shares of the population are already using these models for medical advice, so it seems a bit unlikely to me that this is the main reason the model code and weights are not being provided. It seems much more likely that this is done at least in part for commercial / proprietary reasons. This should be clearly disclosed.

We thank the reviewer for pushing us to be more transparent. The revised *Code Availability* section states that the base model weights, i.e., the Gemini 2 family, and other SOTA LLMs used in our new cross-model experiments (e.g., GPT-5 family and Claude family, see [R1-C8]), are proprietary to their respective developers and are therefore not ours to redistribute. What we *can* release is the contribution of this paper: the agentic architecture layered on top of a given base LLM. We have intentionally kept this acknowledgement brief, in line with our overall framing that the manuscript is a research prototype rather than a deployable product (line 403).

[R1-C5] Further, absent code or data, it would be extremely difficult to reproduce this work. The paper states that, 'For reproducibility, we have documented all technical methods while keeping the paper accessible to a clinical and general scientific audience'. But the whole point of the paper is that creating and validating this system took a great deal of work, resources, and time; the mere textual description of how this was done is not at all sufficient to replicate that.

We agree, and the open release directly addresses this. Please see our responses to [R1-C2] and [R1-C3]: the revised Methods *Code Availability* and *Data Availability* sections enumerate the released artifacts; the revised *Abstract* and *Introduction* now announce the commitment in plain language; and Methods *Code Availability* describes the license terms.

[R1-C6] On data: It is not clear what is meant by 'The de-identified dataset used in this study is available to approved researchers for reproducibility purposes only.' Which components of the dataset does this refer to? (At least some rubrics, for example, appear to be included in the supplement.) It's also not clear what 'to approved researchers for reproducibility purposes only' means - who are the approved researchers, and does this mean that they aren't allowed to build on this work, or use the datasets for further purposes? I think a major value of this work is in fact all the data that was collected; failing to release that, or allow it to be used freely, significantly undermines the utility to other researchers.

We have rewritten the *Data Availability* section to remove this ambiguous language. The section now clearly lists the released benchmark tasks and WEAR-ME dataset access link. Please see our responses to [R1-C2] and [R1-C3].

[R1-C7] The second major issue is the performance of the system does not seem to be that good, and comparison to relevant baselines is lacking. For example, both regarding the Data Science Agent (and throughout), it would be good to give some sense of whether these numbers are good enough to reliably be used. Clearly the agent improves on the base Gemini model. But that on its own isn't very surprising, and my sense from Figure 3 (which is helpful) is that the agent actually makes errors at a high enough rate that it's probably not reliable enough to use safely in health settings; this sense is substantiated by the 'Which Analyses Can the Data Science Agent Conduct?' section. Similarly, 'the DE Agent achieves an overall accuracy of 83.6% while the base Gemini achieves 81.8%' ~ this is not that large an improvement, and again also doesn't seem accurate enough to use.

We agree that PHA's absolute numbers are not the strongest possible among all frontier LLMs, and our paper was explicit that we did not aim to train a new LLM. In the revised *Discussion*, we now foreground the real contribution: PHA represents the most comprehensive reported result for this combination of wearable-data reasoning, personalized domain expertise, and multi-turn health coaching, achieved *without* fine-tuning the base model and via a systematic, modular, open-sourced architecture grounded in four empirically derived Critical User Journeys, instantiated as three specialist sub-agents plus an orchestrator, and validated by 1,350 hours of end-user and expert human evaluation across 11 benchmark tasks (expanded from the original 10 benchmark tasks, see [R4-C3]). Combined with the open release of the implementation code, the evaluation suite, and the de-identified WEAR-ME evaluation subset, this architecture is intended as a reusable foundation. The community can swap in different

base LLMs, replace individual agents, and directly build stronger personal health agents on top. See also [R1-C8], which makes the model-agnostic nature of the architectural contribution concrete via a new cross-model ablation.

We also explicitly clarify in the *Discussion* that PHA is a *research prototype* for consumer wellness contexts and **is not a diagnostic or clinical-triage system** (line 401). The residual error rates are now framed as motivating further safeguards (persistent disclaimers, high-risk query detection, curated authoritative retrieval) rather than as evidence of deployment readiness.

[R1-C8] It is also standard machine learning practice to compare to all relevant baselines, not just to one's own internal base model. For example, it is necessary to compare to other SOTA models like the GPT family, as well as previously published baselines for the individual tasks. The authors argue that 'In this work, our focus is not to train a new LLM but to design and construct the agentic system around a given base LLM.' But if there already exist systems that can outperform their agentic system, the contribution is unclear.

We appreciate this important comment. We agree that the contribution is clearest when the architecture is shown to be model-agnostic. We have therefore added additional experiments on model-agnostic architecture checks. We mainly focus on the DS Agent and the DE Agent, whose evaluations are easier to scale and automate. The LLM families tested cover multiple LLMs including additional Gemini family (3.0 flash), the GPT family (GPT4o, GPT 4.1, GPT 5-mini) and the Claude family (Sonnet 4.5). We retain the results of the Gemini 2 family as the reference from the main text, while adding new results in the supplementary tables (e.g., Supplementary Tables S7, S8, S13, S14).

Overall, our results demonstrated that the agent performance gains over a base LLM can generalize to other SOTA LLMs, indicating that our agent architecture is model-agnostic. We included these new experiments across *Results*, *Methods*, and *Supplementary* Data sections.

[R1-C9] 'we conducted an in-depth analysis of web search and health forum queries' ~ this is a good idea + nice way to motivate the approach.

We are glad that the reviewer recognized the value of grounding our user-need analysis in real-world consumer queries rather than only formative interviews.

[R1-C10] Particularly compelling would be something that shows this system actually improves health outcomes (even self-rated health outcomes).

We agree that demonstrating downstream health-outcome impact would be particularly compelling. However, deploying our system as a real-world intervention technique is beyond the scope of this work. We acknowledge it as a limitation. We have substantially reinforced the *Limitations* subsection (line 427) to clarify the present scope: our evaluation validates *conversational quality* and *short-term task efficacy* only, and does *not* establish the longer-horizon goal of sustained behavior change or improvement in objective, longitudinal health outcomes. We frame that as requiring a dedicated longitudinal deployment study (multi-session interactions, conversation memory, plausibly additional behavioral interventions such as JITAIs) and position it as the potential future work beyond this paper's scope.

As Reviewer 4 noted, we posit that a lab-style evaluation with real users is appropriate for the present claims and that field deployment is the next step, not a missing component of this paper (see our response to [R4-C3] and [R4-C4]). In our newly added PHA evaluation study (live PHA Task 1 / Task 2 study, lines 258 and 275), we recruited N=45 participants with diverse health conditions to have real-time interactions with the PHA system. Our results demonstrated the advantage of our system over baselines. Our revision addresses that ecological-validity concern within scope.

[R1-C11] Inasmuch as there are 4 user journeys/needs and only 3 agents (not counting the PHA), it would be good to explain more explicitly how the agents map to journeys.

We thank the reviewer for flagging this gap. We added the content in the *Introduction* to clarify the relationship between the four user journeys and three agents (line 36). In addition, the *Results*, "*A Modular, Multi-Agent Architecture for Personalized Health Support*" section (line 133) now explicitly points to the complete 26-subtype CUJ-to-agent assignment in Extended Table 1, and the existing paragraph illustrates a representative blended query showing how the DS, DE, and HC Agents are composed by the orchestrator.

[R1-C12] 'Timeframe, Data Transforms, Data Sufficiency, Summary Statistics, Statistical Tests, and Overall Alignment' ~ this is not adequately explained, making the subsequent paragraphs hard to understand.

We have now clarified these detailed rubrics in the *Method, Data Science Agent Evaluation, Task 1* section (line 819). To make the results part concise, we added a simple description and point to the *Method* section with in-depth explanations. We explained these six categories of rubrics below:

- Timeframe: Does the analysis consider a specific timeframe (e.g., past month, past 6 months) when it helps personalize and align with user intent?

- Data Transforms: Does the approach correctly reference existing columns and apply any necessary data transformations, without logical errors, in a way that others can reliably reproduce?
- Data Sufficiency: Does the approach reasonably and clearly assess whether there is enough data, both in terms of available rows and non-missing cells within columns, to support the intended transformations, calculations, and statistical analyses?
- Summary Statistics: Does the approach appropriately calculate summary statistics where helpful, and are the chosen statistics reasonable given the context and question being answered?
- Statistical Tests: Does the approach appropriately apply statistical tests when helpful, with clear, reproducible methods, reasonable test choices, and proper consideration of distributional assumptions?
- Overall Alignment: Finally, given the evaluation of the above dimensions, does the approach align with the user's intent?

In the actual evaluation, these six categories will be expanded into 26 rubrics arranged in a conditional hierarchy. The detailed score deduction breakdown can be found in Supplemental Section C.3.1.

[R1-C13] Figures are generally clear and helpful.

We are glad that the reviewer found the figures clear and helpful. We have updated Fig 1 and Fig 6 to reflect our additional live user studies (PHA Task 1 and Task 2), see [R4-C3] for more details.

[R1-C14] The manuscript is very long and not that interesting to read due to the many similar numerical comparisons, I think many of which could be moved to supp.

We thank the reviewer for this critique. We have substantially condensed the main paper (Introduction + Results + Discussion) without changing the main story. The majority of the cut is in the *Results* section. Specifically, rubric definitions and study-design details are now mainly presented in *Methods*, with a brief pointer in *Results*. Per-rubric numerical results are now mainly presented in the supplemental tables. The four agent *Results* subsections (DS, DE, HC, PHA) now read as a sequence of headline findings rather than enumerations of per-rubric tuples. We hope this makes the revised manuscript substantially easier to read end-to-end while preserving all the underlying evidence and keeping them easily accessible.

[R1-C15] Typos: 1. Methodssection (and in general 'methods' lacks a space after it). 2. ”Am

We thank the reviewer for catching these. A full proofread pass has been conducted and these errors have been corrected.

Reviewer #2 and #3

[Summary of the Study] The paper presents a multi-agent Personal Health Agent (PHA) framework designed to provide personalized, evidence-based health insights. The framework integrates three specialized sub-agents coordinated by an orchestrator: (Data Science Agent, Domain Expert Agent, Health Coach Agent). The orchestrator coordinates these agents to address Critical User Journeys (CUJs) including data interpretation, knowledge seeking, wellness guidance, and symptom assessment (CUJ4). Evaluation involved 10 benchmark tasks, over 7,000 annotations, and 1,100+ hours of human evaluation across both end-user and expert perspectives. Key performance results: DS Agent: +22% improvement in plan quality, +17% code reliability over baseline. DE Agent: +86.6% factual comprehensiveness, +64.8% trustworthiness, +43.8% contextualization. HC Agent: Improved conversational quality and motivational interviewing skills, but limited in progress tracking. PHA Overall: +17.1% expert-rated and +4.6% user-rated improvement over single or parallel agent systems.

[R2R3-C1] Strengths: Modular and transparent architecture enabling separate safety controls and interpretability. Integration of three complementary agents enhances specialization and reasoning quality. Rigorous, rubric-based evaluation with high inter-rater reliability (IRR = 80.2%, ICC = 0.838). DS Agent demonstrates effective translation of ambiguous user queries into structured, statistically grounded analysis plans. DE Agent leverages factuality and citation tools for improved traceability and reduced hallucination risk. HC Agent incorporates modular dialogue flow for better user engagement. User-centered design involving 1,370 health queries, 555 surveys, and expert workshops ensures relevance. Early acknowledgment of ethical, privacy, and regulatory considerations. Sandboxed DS execution adds a layer of computational control.

We thank the reviewers for the careful and accurate summary of the work. We really appreciate the reviewers' recognition of these design and evaluation strengths. These were guiding principles throughout the project, and we are glad they came through in the manuscript. We respond to each item in the weaknesses below.

[R2R3-C2] Safety: DE Agent's diagnostic reasoning accuracy (46.1%) is below acceptable triage thresholds. Risk of users mistaking educational explanations for diagnostic guidance. No triage or uncertainty management system for high-risk interactions. Incomplete reasoning chains (8.5% of interactions) lead to ambiguous or inconclusive outputs. Overreliance on open web searches introduces misinformation risk. Sandbox isolation not formally validated for data safety or code execution integrity.

We thank the reviewers for these substantive safety concerns. We want to clarify that PHA is a research prototype for consumer wellness contexts. It is not a diagnostic or clinical-triage system, and it is not intended to replace professional medical advice, diagnosis, or treatment. Therefore, it is not ready for serving as Software as Medical Device (SaMD). We have revised the *Discussion, Risk and Ethical Considerations for Future Safeguards* subsection (line 402) to make this explicit research-prototype disclaimer.

With that being said, we do agree with the reviewers about the key safeguards necessary for future clinical applications. We added discussion (line 412) on persistent cross-turn disclaimers rendered as a critical interface element, high-risk query detection that can refuse or reformulate a query as a triage-to-clinician handoff, and DE Agent retrieval constrained to curated authoritative databases (WHO, PubMed, NICE, CDC) for health content with explicit source provenance. Moreover, a separate technical-considerations paragraph (starting from line 417) covers layered explanations and data-provenance summaries, sandboxed code execution with plans for formal sandbox validation, and assumption-aware quantitative reasoning. We envision these directions as important future work.

[R2R3-C3] Accuracy: DS Agent often ignores data distribution assumptions, leading to invalid or underpowered statistical inferences. Repeated retries needed for successful code execution, reducing reliability and efficiency. Evaluation bias due to reliance on LLM-based autoraters that share model architecture. Dataset (WEAR-ME) is Fitbit-centric and lacks demographic diversity.

We agree with these concerns and have highlighted them in the revised discussion sections. For distribution assumption awareness, we have added a *Discussion, Risk & Ethics* paragraph (line 421) explicitly states that we expect the DS Agent's under-attention to distributional assumptions to improve as underlying LLMs become more capable of quantitative / statistical reasoning, and as agent pipelines couple LLMs more tightly with symbolic and statistical engines. We list several potential engineering mitigations, such as automated distribution checks, robustness testing, and uncertainty-calibration protocols.

For repeated retries, we want to clarify that retries are only necessary when errors were captured by the sandbox. When the code is executed successfully, repetition is not needed. Therefore it does not impact reliability or efficiency. Meanwhile, the iterative self-correction loop is intentional and, as reported in Results, improves performance from 75.5% (first attempt) to 79.0% (after five trials).

We shared the concern about LLM-based autoraters. We mitigated this through a validation against 354 human-expert annotations, achieving an ICC(3,1)=0.838. This demonstrated that our autoraters are aligned with human experts. With that being said, we recognize that potential bias cannot be fully removed despite the validation. We now have explicitly mentioned

this as a limitation in the *Discussion, Limitations* paragraph (line 438). Meanwhile, we also release our evaluation benchmark suite, as detailed in the Data *Availability* section, so that the community can re-run it with alternative autoraters and human judges.

We also acknowledge that WEAR-ME is Fitbit-centric and drawn primarily from English-speaking, health-literate adults, under-representing low-literacy users, non-English speakers, elderly populations, and people with disabilities (lines 407 and 438). Crucially, we emphasize that by releasing the de-identified WEAR-ME evaluation subset together with the agent and evaluation code, the dataset and its limitations are now themselves open artifacts: researchers can measure, audit, and even extend this dataset (additional devices such as Apple Health, Garmin, Oura; additional demographic strata).

[R2R3-C4] Fairness: System tested primarily on English-speaking, health-literate users; lacks multilingual or low-literacy support. Potential for embedded algorithmic bias in personalized recommendations, particularly around socioeconomic or demographic variables.

Following [R2R3-C3], we have added algorithmic-bias and health-equity content to the *Discussion, Risk & Ethics* (line 406) and *Limitation* (line 438) subsections to acknowledge the fairness risk. A single research work is nearly impossible to comprehensively measure a personal health agent across all relevant subpopulations. We envision more future work is needed from the community to measure and audit fairness continuously. Our open release is specifically intended to serve as a starting point and unlock the audit capacity for the broader community.

[R2R3-C5] Explainability: Disconnect between expert and user perception: users find responses impersonal or unclear. Data provenance opaque—users cannot see which data were used or how they informed recommendations. Figures and visual materials lack clarity and accessibility for general audiences.

We appreciate these comments. Our revised *Discussion, Risk & Ethics* subsection (line 419) now proposes layered explanations (progressive disclosure of reasoning and evidence) and data-provenance summaries that explicitly show which retrieved sources informed each recommendation. This combination is designed to improve transparency without overwhelming non-expert users, and it also provides a natural substrate for user-initiated follow-ups about specific recommendations (also addressing [R2R3-C10]).

[R2R3-C6] Robustness and Regulation: No validation or calibration for noisy consumer wearable data, risking inaccurate health insights. Multi-agent orchestration increases latency (244s vs 36s) and computational cost, limiting scalability. Regulatory pathway undefined; the PHA

performs functions consistent with Software as a Medical Device (SaMD) but lacks stated intended use, risk class, or compliance strategy.

We agree with the reviewers that noisy data is a common challenge for all wearable-related research. In line 143, we now clarify that our WEAR-ME dataset has already gone through a data quality check by filtering users with unreliable wearable data (e.g., short or unreliable wearing period, high missing data rate). We acknowledge that more future research is needed to handle wearable data more robustly and discuss wearable-input calibration as a potential future direction in *Discussion, Risks & Ethics* subsection (line 423).

Latency and computational cost are acknowledged in *Discussion, Limitation* (line 441) and *Methods, Computational Cost* paragraphs (lines 1112): compared to the single-agent PHIA baseline, PHA involves more LLM calls (6.5 vs. 3.0 per query) and higher latency (244.4s vs. 36.4s). In *Discussion* (line 443), we envision potential mitigations via optimized orchestration, parallel sub-agent execution, caching, and lighter-weight routing models. In fact, as detailed in [R2R3-C8], our new experiments with the most recent Gemini-3-flash-lite reduced the latency to 97.6 ± 6.2 sec, which is more than 50% reduction from our original implementation. This supports our speculation about the efficiency improvement as a future engineering refinement pathway.

On regulatory status, as mentioned in [R2R3-C2], we clarify PHA as a research prototype for consumer wellness rather than a diagnostic or clinical-triage system. The open-sourced implementation code will be released under an Apache 2.0 license with a research-only disclaimer (in *Methods, Code Availability*), prohibiting clinical deployment without additional validation. We appreciate reviewers' suggestion of the regulatory landscape, and we are aware of the SaMD framework and the adjacent quality-management standards that any future clinical translation would need to navigate (e.g., ISO 13485 for QMS, IEC 62304 for medical-device software lifecycle, ISO 14971 for risk management, IEC 62366-1 for usability engineering, alongside post-market surveillance planning, as well as HIPAA / GDPR / EU AI Act). The revised manuscript intentionally does not adopt the SaMD framing, because PHA as released is a research prototype rather than a clinical system. The regulatory pathway is therefore scoped to future work whenever a clinical-grade derivative system is built on top of the released agentic architecture (line 427).

[R2R3-C7] Potential for user overreliance on system outputs, delaying professional consultation. Lack of longitudinal testing for multi-session safety and behavior tracking. Inconsistent or unclear disclaimers across user interfaces.

We appreciate these important concerns. In the *Discussion, Risks & Ethics* subsection (line 411), we surface the risks of overreliance and propose potential future solutions (line 412) of high-risk-query detection and persistent cross-turn disclaimers to address these concerns.

We envision the longitudinal evaluation as a future work. As Reviewer 4 noted, we posit that a lab-style evaluation with real users is appropriate for the present claims and that longitudinal field deployment is the next step (see our response to [R4-C3] and [R4-C4]). Our newly added PHA evaluation study (line 256), 45 participants with diverse health conditions were recruited to have real-time interactions with the PHA system. The results demonstrated the advantage of our system over baselines, addressing the ecological-validity concern.

[R2R3-C8] Absence of interdisciplinary expert review across DS and DE outputs. System latency limits real-time usability. No reporting of uncertainty or confidence propagation in results.

We clarify that during the PHA evaluation tasks (where all three subagents DS, DE, and HC working together), we have health experts reviewing the system output (3 experts in PHA Task 2, and 5 experts in PHA Task 3). These experts are professional health coaches with deep expertise in interpreting longitudinal wearable signals and clinical reports, who provide reliable evaluation on the outcomes. As indicated in Figure 6(c) (Task 2) and Supplemental Figure S7(b) (Task 3), the system's interdisciplinary capability (e.g., User Data Interpretation - DS + DE, Effective Synthesis - DS + DE + HC) was carefully rated by experts, and PHA system significantly outperforms the baseline systems. We clarify that our results include standard errors of the mean throughout the result tables in Supplemental materials.

We agree with the concerns on system latency, which is the original reason why we didn't conduct a real-time evaluation. As we mentioned in [R2R3-C7] (more details in [R4-C3]), we have now added a live user study that supports real-time interaction (line 256). During the study, we intentionally inserted random response delays in the systems to control the impact of the system latency. Meanwhile, participants were explicitly instructed to exclude system latency from the evaluation consideration. Meanwhile, since our open-source implementation of the PHA system supports diverse LLM backends, we further experimented with faster LLMs. Our results showed that the system latency can be reduced to 97.6 ± 6.2 sec when using the most recent Gemini-3-flash-lite, which is more than 50% reduction compared to our original implementation. This further supports our speculation in *Discussion, Limitation* subsection (line 444) about the LLM efficiency improvement as a future engineering refinement pathway.

[R2R3-C9] Minimal representation of disability and elderly populations. No use of formal fairness metrics such as demographic parity or equalized odds.

Following [R2R3-C3] and [R2R3-C4], we have added fairness concerns to the *Discussion, Risk & Ethics* (line 405) and *Limitation* (starting from line 429) subsections to acknowledge the risk. In our newly added user study (PHA Task1), our study population covers all 10 WEAR-ME health profiles (including diverse health conditions, see details in Supplemental Sec. B.2 and Table S35). Nevertheless, we agree with this concern and explicitly call out under-representation of disability and elderly populations and propose demographic-stratified audits.

[R2R3-C10] Limited interactivity for users to question recommendations ('why' or 'how'). Inconsistent transparency standards between agents.

We clarify that our system supports dynamic and open-ended interactions. If a user is curious to learn more about the recommendations, they can always follow up with the agent to ask about 'why' or 'how'. Meanwhile, we envision future work about the layered-explanations and data-provenance content in *Discussion, Risk & Ethics* (line 419) can further provide additional methods for user-initiated follow-ups about specific recommendations.

[R2R3-C11] No version control or audit trail to support reproducibility. Lack of standardized error-handling for inter-agent conflicts or failed computations.

The released implementation of the PHA will directly address the version control and the reproducibility concern. As for the error-handling, we clarify that we have multiple safeguard methods. For instance, for the DS agent, our sandbox will automatically catch errors and automatically re-execute with prior error messages taken into account. For the multi-agent PHA system, the orchestrator's reflection step is itself designed to mediate when sub-agents produce conflicting suggestions.

[R2R3-C12] Recommendations (Condensed Summary): Establish triage and uncertainty-calibration protocols for symptom assessment. Restrict DE Agent web access to curated, authoritative databases (WHO, PubMed, NICE, CDC). Incorporate automated data-distribution checks, bias detection, and robust statistical modeling. Broaden dataset diversity and implement fairness dashboards for continuous audit. Add layered explanations, data provenance summaries, and clear user-facing rationales. Validate wearable input data with quality control and calibration mechanisms. Reduce computational latency through optimized orchestration or specialized hardware. Conduct regulatory alignment: Define intended use and risk class under SaMD frameworks. Align with ISO 13485, IEC 62304, and ISO 14971 standards. Perform usability testing (IEC 62366-1) and post-market surveillance planning.

We thank the reviewers for this consolidated set of recommendations, and our point-by-point responses above ([R2R3-C3] through [R2R3-C11]) address each item individually. In the revised

manuscript, we have woven these recommendations directly into the relevant *Results*, *Discussion*, and *Methods* subsections, while keeping the framing compact. Specifically:

- Triage / high-risk-query detection and DE Agent retrieval constrained to curated authoritative databases (WHO, PubMed, NICE, CDC) are addressed in the Discussion, Risk & Ethical Considerations safeguards paragraph (line 416);
- DS Agent distributional checks, robustness testing, and uncertainty calibration, together with layered explanations and data-provenance summaries, are addressed in the subsequent technical-considerations paragraph (lines 420 to 424);
- Demographic diversity, fairness audits, and under-represented populations (disability, elderly, non-English speakers, low-literacy users) are addressed both in the Risk & Ethics safeguards (line 408) and Limitations subsections (line 438);
- Computational cost and latency are addressed with the new evaluation with the most recent LLMs, as well as are addressed in the end of the Limitations subsection (line 441), with concrete mitigation pathways including optimized orchestration, parallel sub-agent execution, caching, and lighter-weight LLM backends (line 444);
- Longitudinal, ecologically valid deployment studies are addressed in the beginning of the *Limitations* subsection (line 431).
- We note that the specific medical-device frameworks the reviewers list (ISO 13485 for QMS, IEC 62304 for medical-device software lifecycle, ISO 14971 for risk management, IEC 62366-1 for usability engineering, and SaMD post-market surveillance planning) are discussed in this response letter (see [R2R3-C6]) but are intentionally kept out of the manuscript itself, because PHA as released is a research prototype for consumer wellness rather than a clinical or diagnostic system. Any future clinical-grade derivative built on top of the released architecture would need to navigate those frameworks, and we flag this scoping clearly in the revised *Discussion* (line 427).

Overall, we close the *Discussion* subsections by framing these collectively as open community challenges that illustrate concrete future directions, unlocked by our open release of the agentic system, the benchmark tasks, and the WEAR-ME dataset.

[R2R3-C13] The PHA framework represents a significant advancement in the integration of data science, medical reasoning, and behavioral coaching through a modular, multi-agent architecture. Its methodological rigor, comprehensive evaluation, and user-centered design position it as a potential foundation for future personal health AI systems. However, key issues related to diagnostic safety, data robustness, fairness, interpretability, and regulatory compliance must be addressed before real-world implementation. With targeted refinements in these domains, the PHA has the potential to evolve into a clinically reliable and ethically deployable personal health assistant.

We really appreciate the reviewers' recognition of the methodological rigor, comprehensive evaluation, and user-centered design, and we share their view that diagnostic safety, data robustness, fairness, interpretability, and regulatory compliance must be addressed before any real-world clinical implementation. The revisions above are intended to move the discussion concretely along those directions, while also clarifying that the present release is a research prototype for consumer wellness. The clinical-deployment-ready version is the natural next step that the open release of our agentic system is designed to enable for the broader community.

[R2R3-C14] We would also like to request for a copy of the code or access to agent to test for the validity of the results.

We thank the reviewers for this concrete validation request, and we have committed to public release in direct response. The revised *Methods, Code Availability* section (line 1140) now open-sources the core PHA agentic system, including the orchestrator logic, the three specialist sub-agents (DS, DE, HC), their prompt templates, tool-use scaffolding, sandboxed code-execution harness, and configuration and example scripts required to instantiate the system on top of any sufficiently capable base LLM. The released codebase is model-agnostic: while our main experiments use the Gemini 2 family, the same architecture composes with additional SOTA LLMs in the new cross-model ablation (additional Gemini family, GPT family, and Claude family; see our response to [R1-C8]). Consistent with our framing of PHA as a research prototype for consumer wellness rather than a deployable clinical system (see [R2R3-C2] and [R2R3-C6]), the code is released under an Apache 2.0 license with a disclaimer, with the safeguards and intended-use scoping described in the *Discussion, Risk & Ethical Considerations* subsection.

The revised *Methods, Data Availability* section (line 1129) accompanies this release with: (i) the complete benchmark data, tasks, and results for all 11 evaluation tasks across the DS, DE, HC, and PHA agents; and (ii) the WEAR-ME dataset access link to the full dataset (N=1,165) under the existing IRB-approved process, as well as the 50-persona WEAR-ME evaluation subset with the persona background stories.

Together, these artifacts enable a researcher starting from our release and a base LLM API to reproduce our reported benchmarks end-to-end on the publicly released benchmarks, and to extend the system to new base LLMs, new sub-agents, alternative autoraters, or new datasets, directly supporting the validation for the community.

Reviewer #4

[Summary] A. The authors present the design and evaluation of a Personal Health Agent (PHA) for personalized, data-driven health recommendations. The authors propose a modular, multi-agent system consisting of a data science agent, domain expert agent, health coaching agent, and orchestration agent. The agent design is informed by four critical user journeys identified through several user research activities. The system's modular design allows the authors identify core capabilities for each agent and evaluate each capability independently. The authors develop extensive evaluation criteria and rubrics, performing a mixture of static evaluations, LLM auto-evaluation, and human evaluation with both end-users and experts. Across their evaluations, the authors find that their PHA system substantially outperforms various baseline agents across many dimensions of interest.

[R4-C1] B. This research topic is very timely and I believe the present work would be of interest to academic researchers and industry practitioners alike. In particular, I perceive the work's strongest contribution to be the extensive and rigorous evaluation suite. User research is necessary, but also time consuming and costly. This research identifies the key capabilities of interest to address user needs and provides clear, measurable benchmarks and rubrics for evaluating them. This evaluation is valuable for both future technical research aiming to build better agents and clinical research aiming to validate intervention quality prior to deployment. However, as I discuss in point C, I also have some concerns with the evaluation of PHA Task 1. The PHA system appears to be a thoughtful combination of existing work on LLM multi-agent collaboration, data analysis agents, diagnosis agents, and health coaching agents. Given that the system itself does not introduce much technical novelty, I would consider it to be a marginal contribution. However, I would not deem this disqualifying in light of the extensive evaluations performed.

We thank the reviewer for this careful and accurate summary of the work. We really appreciate the reviewer's recognition that the evaluation suite is the work's strongest contribution and that the user-research-grounded benchmarks and rubrics are valuable for both future technical and clinical research. We agree that the system's novelty is primarily in the careful synthesis and rigorous evaluation rather than in any single new architectural element. Moreover, in our revised version, the open release of the implementation code, evaluation suite, and de-identified WEAR-ME evaluation subset (see [R4-C9]) is intended precisely to maximize the downstream research impact of this evaluation infrastructure.

[R4-C2] C. I greatly appreciated the author's user-centered approach, beginning by identifying critical user journeys through mixed methods user research. This perspective is frequently missing from health AI research, and is particularly crucial for user/patient-facing applications like personal health recommendations. I also appreciated how the authors modularized the

system with clear capabilities that are subsequently matched to evaluation tasks. This leads to a very clean evaluation. However, one aspect I was missing was the through line from the CUJs to the particular multi-agent architecture, capabilities, and evaluations. How and why does this system design address these particular user journeys, and why is it preferable to other designs considered? How did the authors navigate design tradeoffs and determine the capabilities of each agent in relation to the user journeys? These additional details would help the reader understand how the extensive evaluations performed map back to the original user needs identified.

We are glad that the reviewer found the user-centered approach and modular evaluation design valuable, and we agree that the through-line from CUJs to the specific multi-agent architecture deserved to be made more explicit. We have therefore added paragraphs in the *Introduction* (lines 36-41) that makes the rationale explicit: the four CUJs span fundamentally different capability requirements (numerical precision and code execution for personal-data analysis, strict grounding in medical facts for knowledge and symptoms, empathy and conversational fluidity for coaching), and our initial explorations suggested that a monolithic LLM struggles to balance these competing objectives, which led us to a modular multi-agent architecture in which specialist agents are decoupled by capability while a central orchestrator maintains a cohesive user experience. The revised *Results*, *A Modular, Multi-Agent Architecture* subsection (line 133) now further illustrates how blended queries compose multiple agents at runtime via the orchestrator. We also explicitly highlight the full 26-subtype CUJ-to-agent assignment in Extended Table 1 (line 138).

[R4-C3] My biggest methodological concern is in regard to PHA Task 1, which was evaluated by having end-users compare transcripts from simulated conversations. It's unclear to me why this task was not evaluated by having end-users interact with the system directly, as was done for HC task 1. It is known that people generally are not accurate at perspective taking and subject to biases, see for example: Eyal T, Steffel M, Epley N. Perspective mistaking: Accurately understanding the mind of another requires getting perspective, not taking perspective. Journal of Personality and Social Psychology. 2018; Ickes, W. Empathic Accuracy. Journal of Personality, 1993. The authors note that they chose this evaluation to 'mitigate the potential influence introduced by varied agent response time, user backgrounds, and conversation directions,' but it is possible to account for these factors in a well designed lab study. In my understanding of this work, this task is closest to the overall goal of the system and thus most important for ecological validity. By having participants evaluate a simulated conversation, the present evaluation does not actually measure how well the system is able to conduct open-ended conversations towards generating personalized health insights. It measures how well end-users think the system would be able to generate personalized insights if they pretend to be another person. This discrepancy could possibly explain the lack of clear differences observed in this part of the evaluation. Separately, I do believe this methodology is reasonable

for experts, who are trained to evaluate these kinds of conversations and will pay attention to different factors.

We really appreciate this suggestion and fully agree with the reviewer that live, interactive evaluation is the most ecologically valid test of PHA's open-ended conversational capability, and that transcript-reading evaluation is susceptible to perspective-taking bias. In direct response, we have conducted two new user studies, one with real-time interaction between end-users and AI agent systems (PHA Task 1, line 258), and the other with health experts evaluating the conversation scripts (PHA Task 2, line 275). We restructured the PHA evaluation around a primary live interactive study (Task 1 & 2), while retaining the original synthesized-conversation evaluation as a complementary scaled-up Task 3 (line 289). The original evaluation on synthesized conversations involved a large amount of careful work and yields a meaningful breadth of personas and rater coverage that complements the necessarily time-bounded live sessions in Task 1 and Task 2, so we did not discard it.

Specifically, in our revised paper, the evaluation setups for the multi-agent PHA system include:

- **PHA Task 1 (live end-user evaluation).** N = 45 end-users each role-played a specific WEAR-ME persona (including the persona's demographic context, wearable history, self-reported conditions, and presenting health concern) and engaged in real-time, multi-turn conversations with each of the three systems (single-agent PHIA, parallel multi-agent baseline, PHA), presented in counterbalanced (Latin-square) order, blind to agent conditions. Each participant was mapped to one of the 50 WEAR-ME personas that closely matches their health profile. Participants were sampled stratified by diverse health conditions to ensure that all 10 health profiles were covered, including short-term injuries, Anxiety/Depression, Hypertension, Respiratory Conditions, Hypercholesterolemia, Type II Diabetes, and Cardiovascular Disease. See Supplemental Table S35 for the participant profile matching. Immediately after each conversation, participants completed the same rubric instruments (as with the original PHA evaluation study, see Supplementary Section F.2). After interacting with all three systems for a given persona, they provided comparative rankings (~90 hours of human effort).
- **PHA Task 2 (live expert review).** Three health-coach experts independently reviewed the live conversation scripts produced in Task 1, using the expert rubric items, blind to agent conditions (~140 hours of human effort).
- **PHA Task 3 (synthesized scaled-up evaluation – our original evaluation).** The live evaluations above are bounded by participant time, which limits the breadth of personas and rater coverage they can probe. Task 3 complements them by synthesizing 150 conversations between persona-instructed user models (all 50 WEAR-ME persona) and the three systems and recruiting a separate cohort of 20 end-user generalist evaluators and 5 health experts to rate them using the same Task 1 and Task 2 rubrics. The pattern is consistent with the live findings.

Both the quantitative and qualitative findings across both the live (Task 1 + Task 2) and synthesized (Task 3) evaluations is the same: PHA significantly outperforms both the single-agent PHIA and parallel multi-agent baselines on overall quality, helpfulness, and the per-rubric breakdown, with experts showing a more pronounced preference than end-users. It is noteworthy that some statistical significance originally in Task 3 - End-User was no longer observed in Task 1, due to limited sample size in Task 1. This also motivates our new framing of the necessity of Task 3 for the scale-up evaluation. We believe the current evaluation structure is well-supported and provides a stronger evaluation than either track alone. We have updated the *Results* (lines since 258), *Methods* (lines since 1052), and Supplementary Sections F.2 and F.3 to reflect these changes. Below are the high-level overview of the updated Fig. 6 and new SFig. 7, as shown below.

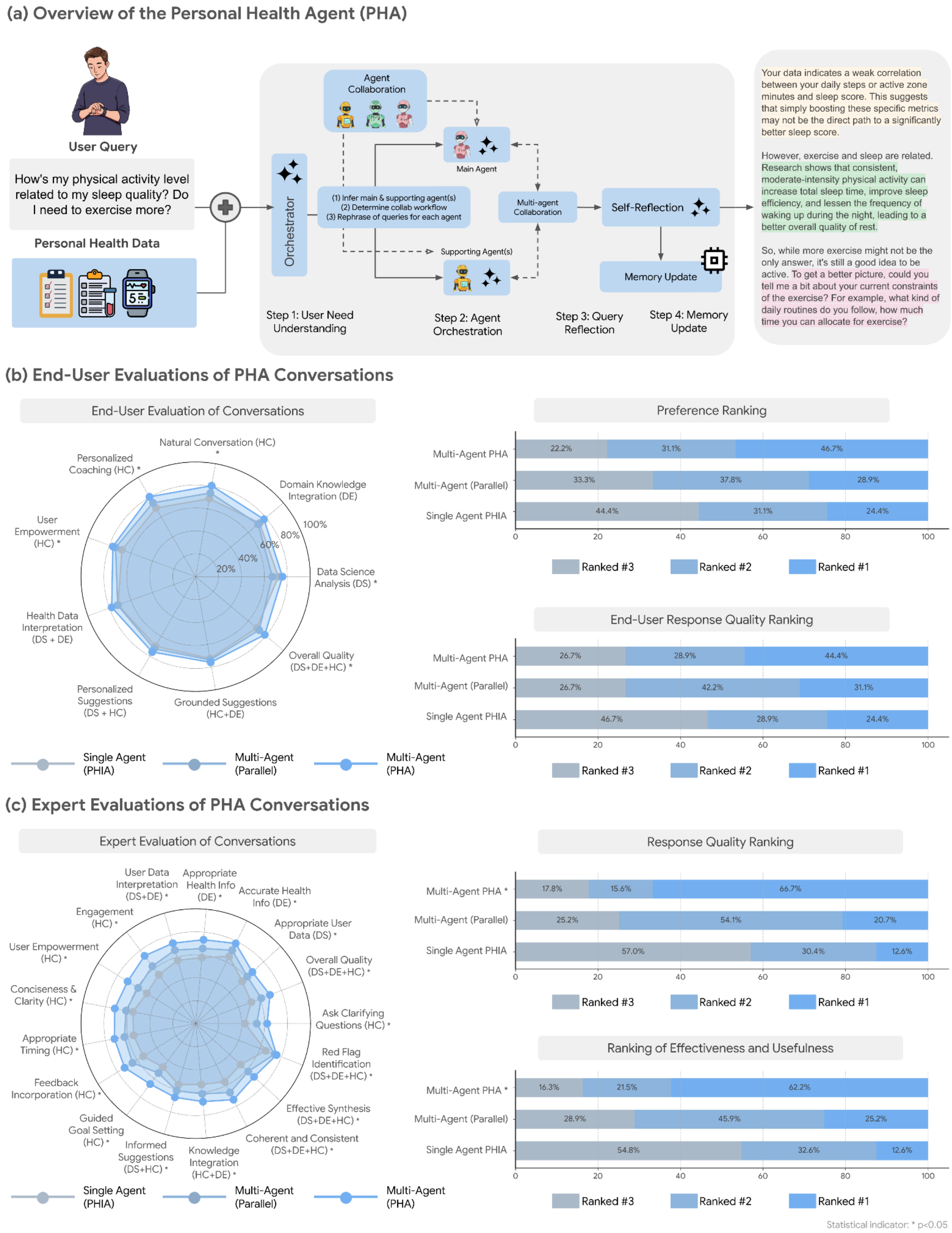

Updated Figure 6 of the main manuscript, which now includes the results of the interactive user study with the PHA.

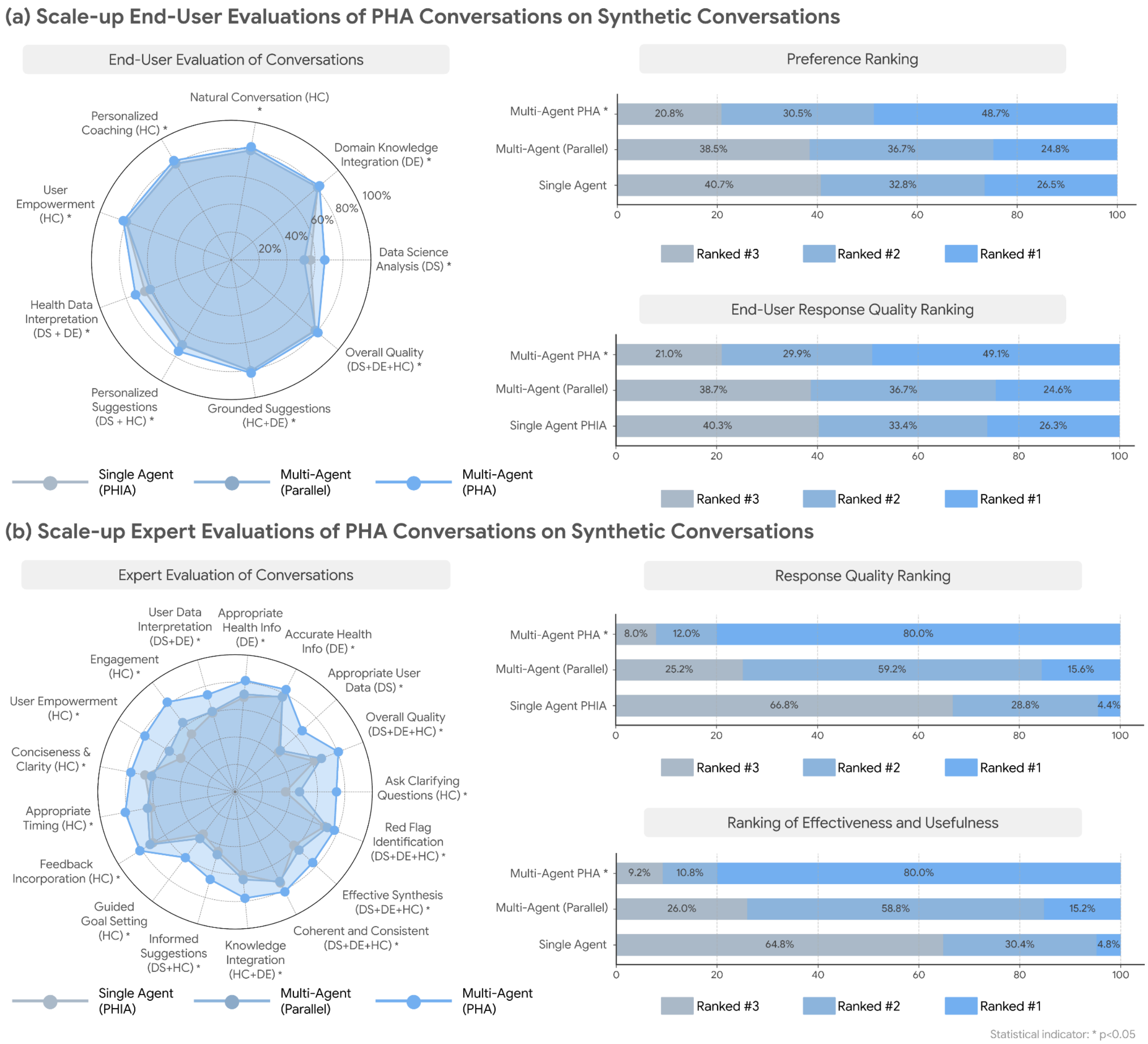

The previous results of PHA with synthetic conversations is moved to Supplemental Figure 7.

[R4-C4] Moreover, the authors discuss the lack of a field study in the limitations section. However, I might argue that a lab study evaluation of PHA with real users, discussing real health concerns, ideally with their real data, would be the most ecologically valid and appropriate evaluation. While I agree that the ultimate goal of this line of work is to impact health behaviors (and in turn long-term health outcomes), the present system was designed to conduct high-quality conversations about personal health, not support longitudinal behavior change. Adapting PHA to support behavior change in longitudinal interactions would likely require substantial additions to the system (e.g., memory of past conversations or additional behavioral interventions like JITAI).

We sincerely thank the reviewer for validating that a lab-style evaluation with real users is the ecologically valid and appropriate evaluation for the goals of the present system, and that a full field deployment is not a prerequisite for the claims made here. Our response in [R4-C3] has addressed this concern, and we also used this framing to tighten the scope argument in *Discussion, Limitations* (line 431). Based on our new studies, the overall PHA evaluation now contains 11 benchmark tasks. Our Fig. 1 has been updated as below:

(a) User-Centered Design to Anatomize Personal Health Needs

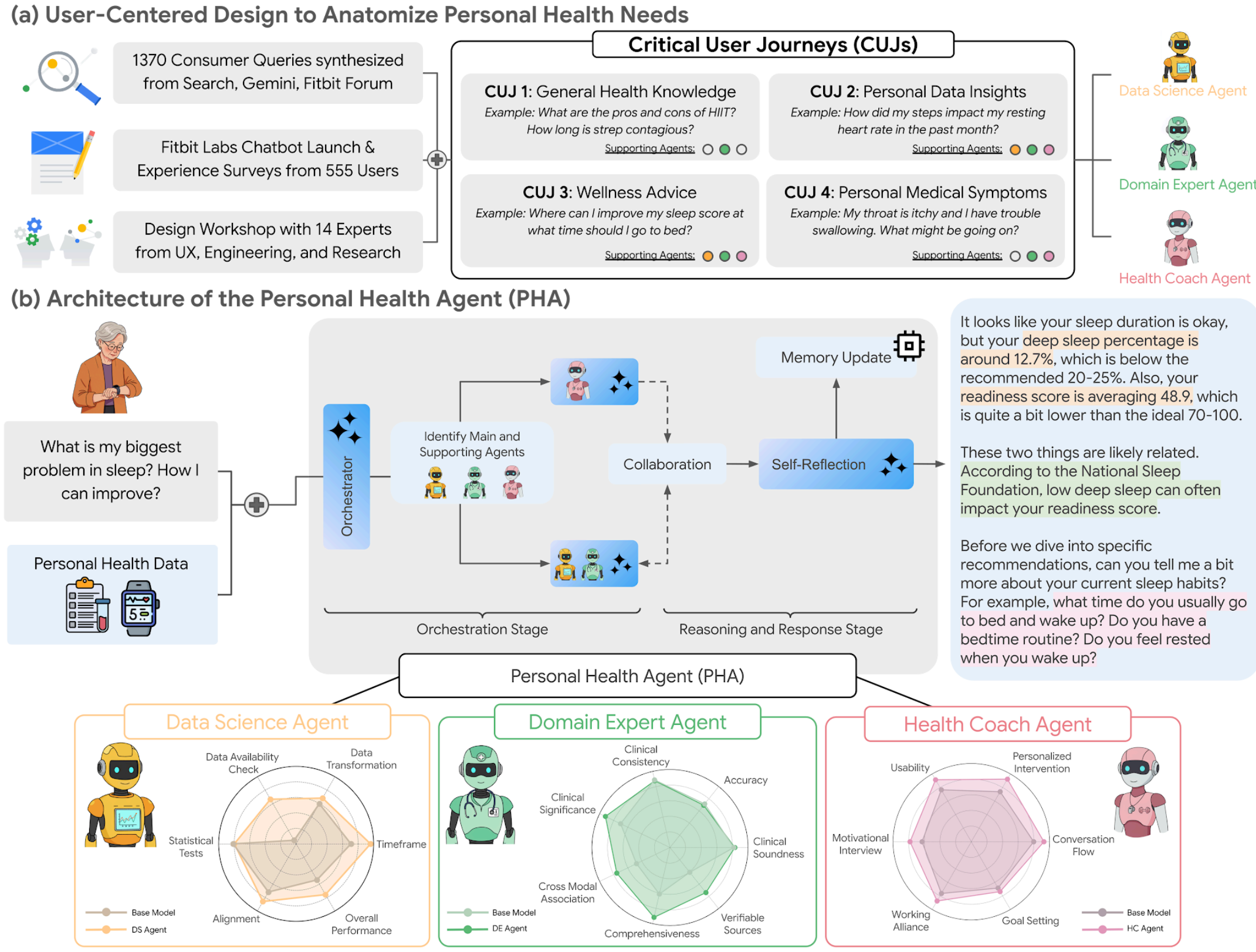

(c) Evaluation Studies for Individual Sub-Agents and the Entire Multi-Agent PHA System

	Assessment Goal	Evaluation	Scale	Human Hours
DS.1	The agent's ability to design a **statistical analysis** plan.	Human: Data Scientists; Auto Eval*	354 Plans x 2 Agent Conds (708 Results)	47
DS.2	The agent's ability to **generate code** to execute the analysis plan.	Human: Software Engineers; Auto Eval*	173 Tests (25 Queries) x 2 Agent Conds (346 Results)	75
DE. 1	The agent's **medical knowledge** and its ability to **reason over case studies** mirroring real-world scenarios.	Auto Eval	2145 MCQs x 2 Agent Conds (4290 Results)	-
DE. 2	The agent's ability to **generate a differential diagnosis** when provided user symptoms.	Auto Eval	1511 User Symptoms x 2 Agent Conds (3022 Diagnosis)	-
DE. 3	The agent's ability to **personalize answers to medical questions** based on context and information about the user	Human: Generalists (End-users)	50 Queries x 2 Agent Conds x 17 Raters (1700 Annotations)	13
DE. 4	The agent's ability to **reason and interpret multi-modal health data** (wearables, lab results, demographics, health surveys)	Human: Experts (MD physicians)	30 Personas x 2 Agent Conds x 5 Raters (300 Annotations)	77
HC.1	The agent's ability to **conduct coaching conversations** from *end-users'* perspectives.	Human: Generalists (End-users)	31 Raters (Multi-turn) x 2 Agent Conds (62 Annotations)	48
HC.2	The agent's ability to **conduct coaching conversations** from *health coach experts'* perspectives.	Human: Experts (Health Coaches)	31 Interactions x 2 Agent Conds x 3 Raters (186 Annotations)	90
P.1	The multi-agent's ability to **synthesize personal data (DS), health domain knowledge (DE), and coaching (HC) capability** in a conversation about achieving personal health goals, from *end-users'* perspectives	Human: Generalists (End-users)	45 Raters (Multi-turn) x 3 Agent Conds (135 Annotations)	90
P.2	The multi-agent's ability to **synthesize personal data (DS), health domain knowledge (DE), and coaching (HC) capability** in a conversation about achieving personal health goals, from *coach experts'* perspectives.	Human: Experts (Health Coaches)	45 Interactions x 3 Agent Conds x 3 Raters (405 Annotations)	140
P.3	**Scale-up Evaluation** of the multi-agent system with Synthesized Conversation Data, from both *end-users'* and *experts'* perspectives	Human: Generalists (End-users), Experts (Health Coaches)	50 Personas x 3 Agent Conds x (20 Generalists + 5 Experts) (3750 Annotations)	500 (Generalists) 270 (Experts)

[R4-C5] D. I found the use of statistics to be largely appropriate. I am curious whether the authors performed corrections for multiple comparisons (either FWER or FDR) when performing tests on multiple rubric items. For example, in Fig 3b or Table S5, are the p-values corrected for the 8 tests conducted?

We thank the reviewer for raising this important methodological check. We have added a dedicated *Methods, Statistical Analysis* subsection (line 1119) that makes our correction strategy explicit, and we have added a one-sentence Holm–Bonferroni robustness check to the caption of every per-rubric results table. Specifically:

- Within each rubric, where a three-system contrast yields three pairwise comparisons (PHIA vs. Multi-Agent Parallel, PHIA vs. PHA, Multi-Agent vs. PHA), we control the family-wise error rate using Tukey's HSD on top of the per-rubric Gamma GLMM. For two-condition rubrics (e.g., DE Agent base vs. agent; HC Task 1/Task 2), each rubric is a single comparison and no within-rubric correction is required.
- For comparative rankings (e.g., Preference and Response-Quality in PHA Task 1; Overall-Quality and Effectiveness in PHA Task 2), where a Friedman omnibus is followed by pairwise Wilcoxon signed-rank tests, we apply the Holm–Bonferroni correction within each ranking metric.
- Across rubric items in any given results table, each rubric item evaluates a pre-specified, methodologically distinct facet of agent quality that was defined during our formative rubric-design phase, before any of the system comparisons were run. For example, the DS analysis-plan rubric (Table S5) consists of five conceptually orthogonal facets (*Timeframe*, *Data Transforms*, *Data Availability*, *Statistical Tests*, *Alignment*), and each pairwise test answers a separate confirmatory question of whether the agent improves on that specific facet. Reporting per-facet p-values without an additional cross-facet FWER/FDR adjustment is consistent with how multi-faceted rubric tables are presented in comparable health-AI evaluations in prior works. For full transparency, we have nonetheless audited every per-rubric results table by applying a strict across-rubric Holm–Bonferroni correction and have added a one-sentence note to each table caption summarising the outcome:

  Robust under correction (note states that an across-rubric Holm–Bonferroni robustness check does not change the statistical significance of any contrast):
    - DS Task 1: Agent Analysis Plan Generation (Supplemental Tab S5)
    - DS Task 2: Code Generation Error Rate (Tab S6)
    - DE Task 1: MCQ (Tab S11)
    - DE Task 2: No correction needed (Tab S12)
    - DE Task 3: Personalized Health-Queries by Generalists (Tab S15 & S16)
    - DE Task 4: Multimodal Summary by Experts (Tab S17 & S18)
    - HC Task 2: Expert Rubrics (Tab S29)

- PHA Task 1: End-User Comparison (Tab S37)
- PHA Task 2: Expert Rubrics (Tab S38)
- PHA Task 2: Expert Comparison (Tab S39)
- PHA Task 3: End-User Rubrics (Tab S40)
- PHA Task 3: End-User Comparison (Tab S41)
- PHA Task 3: Expert Rubrics (Tab S42)
- PHA Task 3: Expert Comparison (Tab S43)

Marginal contrasts flagged in the caption as directional findings that do not survive a strict across-rubric correction. Four tables have rubrics whose pairwise p-values are in the 0.01–0.045 range and would not survive Holm at the table's rubric count. These are now explicitly disclosed in the caption rather than implicitly claimed:

- HC Task 1: End-User Rubrics (Conversation Flow, Progress Measurement, Tab S27)
- HC Task 1: End-User Comparison (Active Listening, Tab S28)
- HC Task 2: Expert Comparison (HC1, HC4, HC5, Tab S30).
- PHA Task 1: End-User Rubrics (Data Analysis, User Empowerment, Overall Quality, Tab S36)

[R4-C6] Some plots with error bars (e.g., Fig 4b) do not have legends describing how they were computed. The radar plots also do not have error bars even though the corresponding supplemental tables report SE, but I do not find this particularly problematic.

We thank the reviewer for catching this. We have now marked the error bar indication directly in Fig 4: the legend for the bar plot now explicitly indicates that error bars represent the standard error of the mean (the corresponding numeric values are in the relevant Supplemental Tables). For the radar-plot panels in Fig 3-6, on-plot error bars are not feasible, but as the reviewer noted, the corresponding supplemental tables report SE for every dimension, so the information remains accessible to the reader.

[R4-C7] E. I found that the conclusions were generally robust and valid. I found that the safety evaluation of the DE agent was presented in a way that could be interpreted as inflating its safety. The authors write 'the safety profile of the DE Agent's summaries was also highly favorable by the experts.' This is true in the sense that most responses were not harmful, but 15% had mild harm and 2% had moderate to severe harm. I do not wish to diminish the author's efforts—this is a very hard problem—but a 2% risk of harm would be highly concerning for a real deployment or product, and more importantly points to an important area of future research. Safety risks are mentioned in the discussion but not tied back to the evaluation's findings.

We thank the reviewer for this important observation. We agree that the original phrasing could read as overstating safety. We have revised the *Results*, *DE Agent* (line 217), and it now reports the distribution from the expert safety review (~83% no/minimal harm, ~15% mild harm, ~2% moderate-to-severe harm), explicitly notes that the residual mild and moderate-to-severe rates are non-trivial for any user-facing health context, and ties these findings directly to the *Discussion* (lines starting from 402): the residual harm rates motivate the safeguards before clinical deployment, such as persistent disclaimers, high-risk query rejection, and restriction to curated authoritative sources.

[R4-C8] Lastly, I wished for a discussion section that more clearly and boldly articulated the frontiers of developing future personal health agents. Which of the limitations require more engineering, which require more user research, and which require fundamental advances in AI capabilities? Based on your evaluations, what should future researchers be focusing their time on?

We thank the reviewer for pushing us to articulate these frontiers more clearly. We have revised the *Discussion* substantially to address all reviewers' concerns (starting from lines 402). Based on our evaluation findings, the open challenges for future personal health agents cluster into three distinct frontiers. The detailed mapping is as follows.

Engineering refinements — well-specified problems whose solutions are achievable on current LLMs and benefit primarily from careful systems work. From our evaluation, these include: reducing PHA's response latency (244s vs 36s for the single-agent baseline, while our recent experiments showed that we can already reduced the latency from 244s to 98s with the latest LLMs, see more details in [R2R3-C8]) via optimized orchestration, parallel sub-agent execution, caching, and lighter-weight routing models; restricting the DE Agent's retrieval tool to curated authoritative health databases (WHO, PubMed, NICE, CDC) with explicit source provenance to mitigate the open-web vulnerabilities we observed; formal sandbox validation (red-teaming, escape testing, resource limits, signed audit trails) for the DS Agent's code-execution path; persistent cross-turn disclaimers as a critical UI element; and layered, on-demand explanations and data-provenance summaries to address the explainability gap.

User-research needs — questions that cannot be answered from internal benchmarks alone and require new empirical studies with humans. From our evaluation: longitudinal behavior change and downstream health-outcome studies (multi-session interactions, conversation memory, plausibly JITAI-style behavioral interventions), which is the current gap between conversational quality (what we measured) and actual health impact; demographic-stratified audits across devices, languages, literacy levels, age, disability, and socioeconomic strata; trust-calibration studies over repeated interactions to understand when users begin to over-rely on the agent and how disclaimers and triage pathways modulate that reliance; and

user studies on which coaching-style adaptations (accountability vs. empathy framing, etc.) work for which users in which states.

Fundamental AI-capability advances — gaps that current LLMs cannot close at the agent layer alone, and that require improvements at the base-model (or training-recipe) level. From our evaluation: reliable statistical and distributional reasoning (the DS Agent under-attends to distributional assumptions because the underlying LLM does); calibrated uncertainty quantification, which is the prerequisite for reliable refusal and triage on high-risk queries and for any clinically-reliable diagnostic reasoning beyond the DE Agent's current 46.1% top-1 differential-diagnosis accuracy; and foundation-model-level bias mitigation, which audits alone cannot solve.

Where future researchers should focus. The three frontiers also map naturally to different research communities, and we believe progress will be fastest if each community prioritizes the frontier it is best equipped to tackle. *Engineering refinements* are the highest-readiness category. Much of this work belongs to systems / applied ML researchers and product engineers. This can begin immediately, and would substantially improve the deployment-readiness of an agent built on current LLMs. *User-research needs* are the natural domain of Human-Computer Interaction (HCI), behavioral-science, and clinical-research communities; this category has the highest cost-per-question and the longest time horizon, but several items, particularly the longitudinal behavior-change study, are irreplaceable, because the field has not yet established what success metrics for a personal health agent should look like in real-world use. *Fundamental AI-capability advances* are the domain of AI / ML / foundation-model researchers and are the blockers to a clinically deployable personal health agent: improvements in calibrated statistical reasoning and clinically-reliable diagnostic reasoning would unlock capabilities that no amount of agent-layer engineering can compensate for.

In the *Discussion*, rather than adding a separate three-way structural breakdown (which we agree would be heavy and space-consuming), we have spread these discussion points across subsections and appended a single summary sentence at the end of the *Limitations* (line 445).

[R4-C9] Moreover, while I found the section on trade-offs for the health coaching agent to be interesting (i.e., prioritizing competency over style), it was not clear to me which specific findings pointed to this interpretation?

We have retained the original competency-over-style paragraph in *Discussion* (line 348) and added a small insertion that names the relevant rubric themes: HC1 (Goal and Purpose Identification) and HC2 (Active Listening) as *structural coaching fundamentals* on which our HC Agent was rated highly, versus HC4 (User Empowerment, the theme most tied to stylistic

framing and tone) where the agents were rated on tie. The specific rubric-level results (see Supplemental Table S27-30) are the underlying evidence for our interpretation: end-users significantly preferred our HC Agent on HC2 Active Listening (71.0% win rate, p = 0.025, β = 0.894; expert-side theme-level win rates 64.5-72.0%), while on HC4 User Empowerment end-users ranked the two agents similarly (48.4% win rate) despite experts strongly preferring our agent (69.9%). In other words, end-users' largest preference for our HC Agent occurred where it provided stronger structural coaching behaviors, not where it varied in empowerment-oriented language.

[R4-C10] F. As discussed above, I think a more ecologically valid evaluation of PHA Task 1 would greatly strengthen the work. Moreover, given my perspective on the evaluation suite being the strongest contribution, I think this work could have much greater impact on future research if the authors release more details and code from their evaluation. I fully understand the organizational and safety risks associated with releasing the agent code, but I am curious if releasing the evaluation code is feasible. I also understand that the dataset on which the evaluation suite is based requires approval, but perhaps it would be possible to release portions of the code that do not depend on it, such as LLM auto graders, case studies, or user simulators.

We deeply appreciate the reviewer's careful considerations. On the more-ecologically-valid evaluation, please see our response to [R4-C3]: PHA Task 1 / Task 2 are now based on live interactive between real end-users and agents (N=45 end-users, N=3 health-coach experts), with the original synthesized-conversation eval retained and labeled as Task 3 for breadth.

On releasing more of the evaluation infrastructure, we totally agree with the reviewer (along with the other reviewers). We are committed to releasing the evaluation benchmark alongside the agent code. The revised *Methods, Code Availability* section (line 1140) open-sources the core PHA agentic system (orchestrator, three specialist sub-agents, prompts, tool-use scaffolding, sandboxed code-execution harness, and configuration / example scripts) under an Apache 2.0 license with a disclaimer. The revised *Methods, Data Availability* section (line 1129) releases the full evaluation-suite materials: benchmark tasks, data, and results, the WEAR-ME dataset access link, as well as the de-identified 50-persona background stories are available under a non-restrictive research-use license.

We believe these open-release commitments can address all reviewers' requests on the data and code access. We also updated the *Introduction* to characterize PHA as an open-source multi-agent framework and to reframe the contribution as a concrete foundation for the community to validate, audit, and further improve on top of our work (line 80).

[R4-C11] H. In general, I found the paper to be well-written. I have no issues with the abstract, introduction, or conclusion. However, the paper is incredibly long and unwieldy. I fully understand the challenges in presenting such a large body of work clearly concisely, but I frequently found myself jumping around in the manuscript trying to piece together the details.

We sincerely thank the reviewer for both the encouraging assessment of the abstract / introduction / conclusion, and for this critique on length and navigability, which aligns with [R1-C14] from Reviewer 1. The two reviews together identified the same root problem: per-rubric numerical comparisons stated inline in *Results* that were also present in supplemental tables, and methodology-level detail that was duplicated between *Results* and *Methods*.

To address these concerns, rubric definitions and study-design details are now presented in *Methods*, with a brief pointer in *Results*. Per-rubric numerical breakdowns are now presented in the supplemental tables, with a single high-level finding plus a supplemental-table pointer in *Results*. The four agent *Results* subsections (DS, DE, HC, PHA) now read as a sequence of headline findings rather than enumerations of per-rubric tuples. We believe this makes the revised manuscript substantially easier to read, while preserving all the underlying evidence.

[R4-C12] G. I found that the authors gave appropriate credit to previous work. In summary, I commend the authors for this work and the enormous amount of effort involved in such an evaluation. I look forward to hearing their thoughts in the rebuttal.

We are glad that the reviewer found the credit to previous work appropriate, and we sincerely appreciate the closing commendation. We hope this rebuttal addresses each of the reviewer's concerns concretely.

Google Research
Google Research
1600 Amphitheatre Parkway
Mountain View, CA 94043

Point-by-Point Response to Reviewers and Editorial Comments for *The Anatomy of a Personal Health Agent*

We would like to thank the editor and all reviewers for their highly encouraging final feedback and for recognizing the substantial improvements made during the previous revision. We are thrilled to reach the "accept in principle" stage. To finalize the manuscript for publication, we have carefully addressed the remaining editorial instructions and reviewer comments through a combination of:

- **Comprehensive Editorial Restructuring**: A complete formatting overhaul of the manuscript to ensure strict compliance with Nature's Formatting Rules, including significant word count reductions, display item reorganization, reference splitting, 40-character subheading limits, and the inclusion of all mandatory statements.
- **Clarification of Future Safeguards**: Precise textual revisions to unambiguously distinguish our current research prototype from the required future clinical safeguards and deployment protocols, ensuring no future features are inadvertently read as empirically validated components of the current system.
- **Standardized Statistical Reporting**: A thorough audit of all figure legends and supplementary tables to explicitly define error bars, ensure consistent statistical test naming (e.g., Wilcoxon signed-rank), and document the use of two-sided testing.
- **Expanded Limitations on Study Design**: An explicit addition to the Limitations section acknowledging the perspective-taking bias inherent in our persona-matched user study, noting it as a critical area for future longitudinal research.

We believe that these final refinements perfectly polish the manuscript for publication and address all remaining items. We truly appreciate the reviewers' and editor's thoughtful guidance throughout this process.

Below, we provide our point-by-point responses to the editorial remarks and each reviewer's comments. The original comments are italicized and in **black,** while our responses are marked in **blue**.

Editorial Remarks:

Editor: *Please revise your paper to address the points made by the referees, and to make some editorial changes to your paper so that it is as brief as possible and complies with our Guide to Authors…*

Author Response: We thank the Editor for the incredibly detailed formatting guidance. We have conducted a top-to-bottom formatting overhaul of the manuscript to ensure full compliance with Nature's guidelines. Key editorial actions taken include:

- **File Format**: The manuscript has been converted and is supplied as a single, flattened .docx file.
- **Length:** We reduced the main text from 6,987 words to safely under the 4,500-word limit. This was achieved by moving the Q&A discussion to the SI and streamlining the Limitations and Conclusions.

Google Research
1600 Amphitheatre Parkway
Mountain View, CA 94043

- **Abstract/Summary Paragraph**: We have included a "Summary Paragraph" at the top of our manuscript file. This paragraph contains 218 words, includes citations, is fully bolded, and properly utilizes the "Here we show" structural transition.
- **Display Items**: We moved one main figure to the Extended Data, ensuring the main text has exactly 5 display items. All main figures have been stripped from the Word document and uploaded as individual, editable vector files (.svg).
- **Subheadings**: All subheadings have been revised to strictly fall under the 40-character limit (including spaces).
- **References**: We separated the references into a Main text list (≤50) and a continuously numbered Methods list.
- **Figure Legends**: All legends are grouped sequentially after the Main References. They explicitly state exact n values, define error bars (e.g., standard error of the mean), and clarify that all statistical tests were two-sided.
- **Supplementary Information & Extended Data**: We moved all SI tables into a standalone document accompanied by an SI Guide. Extended Data legends are provided at the end of the manuscript file, and the ED items themselves have been consolidated and properly sequenced.
- **Mandatory Statements**: We added the required Funding, Additional Information, and updated Data/Code Availability statements. The Reporting Summary and all required checklists have been finalized and uploaded as Related Manuscript Files. Finally, all corresponding authors have linked their ORCID accounts.

Reviewers' Comments:

Reviewer 1

Reviewer 1: *I thank the authors for their response to my concerns, and in particular committing to release additional artifacts and clarifying the contribution of the work, which I believe will increase the rigor and impact of the manuscript. I do not have further comments.*

Response: We sincerely thank Reviewer 1 for their critical eye during the previous rounds of review. Your feedback regarding baselines and open-sourcing fundamentally elevated the scientific rigor of this work.

Google Research
Google Research
1600 Amphitheatre Parkway
Mountain View, CA 94043

Reviewer 2

Reviewer 2 (Remark A): *Overall, the revised manuscript has addressed many of the concerns raised in the previous round of review... Although the revised manuscript generally distinguishes the current research prototype from future clinical safeguards, I suggest that the authors check a few passages to ensure that proposed future safeguards are not inadvertently read as implemented or empirically validated components of the current PHA system. This is particularly relevant to the discussion of CUJ4 as a future "preparatory and collaborative tool," the phrase "immediate engineering mitigations" in relation to automated distribution checks, wearable-input calibration, and uncertainty adjustments, the Code Availability statement referring to "safeguarding protocols," and the description of sandboxing and code validation.*

Response (Remark A): We appreciate the reviewer's careful reading. We completely agree that the distinction between our current research prototype and future clinical deployment requirements must be unambiguous. We have carefully revised the four specific passages mentioned to explicitly frame them as future directions and necessary future safeguards for real-world deployment, rather than features implemented or validated in the current PHA release.

Reviewer 2 (Remark B): *I only suggest that the authors check for consistency between the Methods and supplemental table captions, as the Methods describe post-hoc Wilcoxon signed-rank tests for comparative rankings, whereas some supplemental captions may still refer to Wilcoxon rank-sum tests. If these were paired comparisons, the captions should be corrected to signed-rank tests.*

Response (Remark B): Thank you for catching this typographical inconsistency. Because these were indeed paired comparisons within our study designs, we have audited all supplemental table captions and corrected any erroneous instances of "Wilcoxon rank-sum" to "Wilcoxon signed-rank" to ensure consistency with the *Methods* section.

Reviewer 2 (Remark C): *However, I recommend that the authors ensure that all figure legends and supplemental table captions consistently define whether the reported uncertainty represents standard error, bootstrap standard error, confidence intervals, or another quantity.*

Response (Remark C): We have thoroughly reviewed every figure legend and supplemental table caption in the manuscript. We have added explicit definitions of the reported uncertainty to all relevant captions (e.g., explicitly stating "Error bars represent the standard error of the mean"), ensuring readers do not have to refer back to the general *Methods* text to understand the visualizations.

Google Research

Google Research
1600 Amphitheatre Parkway
Mountain View, CA 94043

Reviewer 2 (Remark D): *Remarks on code availability: The provided code was generally well organized and included sufficient instructions for installation and execution. I was able to install and run the code without major issues.*

Response (Remark D)*:* We are glad to hear that the open-source repository ran without issues. Thank you for taking the time to validate the codebase.

Reviewers 3 and 4

Reviewer 3: *I co-reviewed this manuscript with one of the reviewers who provided the listed reports.*

Response: We thank the reviewer for their time and contribution to the evaluation of this manuscript.

Reviewer 4 (Remark A): *I thank the authors for their thoughtful consideration of the reviewers' concerns and the substantial effort that went into this revision. My original concerns (documented below) are largely addressed, and I believe that the revisions have notably strengthened the paper's contributions and rigor, such that I would recommend the current manuscript for acceptance... Second, I appreciate the additional user studies to address my concerns around the original PHA Task 1 evaluation. While some of my concerns around perspective-taking bias remain (since users were matched to a persona, not acting authentically as themselves) I do believe that the revised study is a strong improvement methodologically over the previous study. Persona matching stratified by health conditions is a reasonable middle ground, though I'd encourage the authors to note the limitations of this design.*

Response (Remark A): We thank you for your guidance throughout the review process; your push for a live, interactive user study undoubtedly made our work stronger. We completely agree with your assessment regarding perspective-taking bias. To address this, we have added an explicit note to the *Limitations and Conclusion* section of the main text. The text now clearly states that while stratifying participants by health conditions to match the WEAR-ME personas increased scenario relevance, it cannot fully eliminate perspective-taking bias, as users were not acting authentically as themselves. We note this as a critical area for future longitudinal research.

Reviewer 4 (Remark B): *Remarks on code availability: The authors provide an accessible README with instructions to install and run the application. I was able to successfully test all 3 version of the agent with my own API key. While I did not attempt to reproduce the results myself, I think the results should be reproducible given what I saw in the repository. The code is sufficiently well-documented and organized to be extended by the community.*

Response: Thank you for taking the time to independently clone, install, and test the three agent versions. We are delighted that the scaffolding worked seamlessly with your API key, and we hope the community finds it just as accessible for future extensions.
